# Merging Organocatalysis with 1,2-Boronate Rearrangement: A Lewis Base-Catalyzed Asymmetric Multicomponent Reaction

**DOI:** 10.1021/jacs.4c11113

**Published:** 2024-09-24

**Authors:** Hong-Cheng Shen, Varinder K. Aggarwal

**Affiliations:** School of Chemistry, University of Bristol, Cantock’s Close, Bristol, BS8 1TS, U.K.

## Abstract

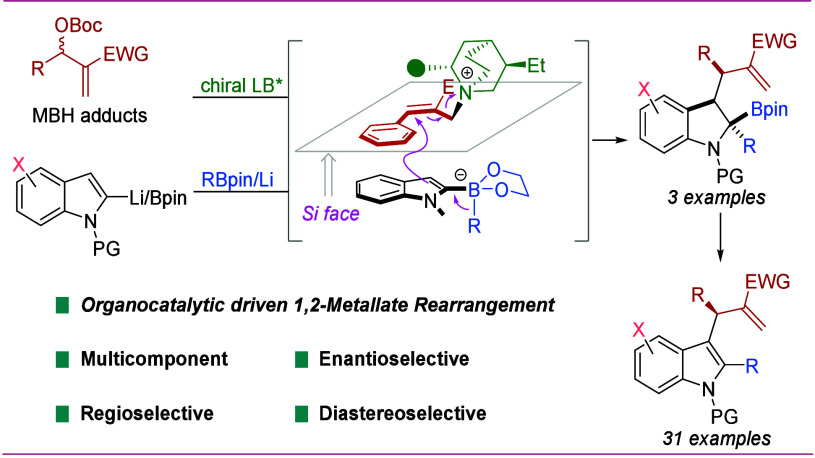

Catalytic asymmetric multicomponent 1,2-boronate rearrangements provide a practical approach for synthesizing highly valuable enantioenriched boronic esters. When applied to alkenyl or heteroaryl boronates, these reactions have relied mainly on transition-metal catalysis. Herein, we present an organocatalytic, Lewis base-catalyzed asymmetric multicomponent 1,2-boronate rearrangement, involving indoles, boronic esters, and Morita–Baylis–Hillman carbonates, leading to enantioenriched, highly substituted indole and indoline derivatives. Using cinchona alkaloid-based catalysts, high selectivity has been achieved, enabling expansion of the chemical space around pharmaceutically relevant indole and indoline derivatives.

Multicomponent reactions (MCRs) provide a simple and practical strategy for the direct synthesis of diverse and structurally distinct compounds from basic feedstocks.^1^ The compound libraries derived from such reactions have proven invaluable as chemical probes, natural product mimics, and potential leads in drug discovery.^2^ Of particular utility are the asymmetric multicomponent reactions (AMCRs) catalyzed by either organocatalysis or metal catalysis.^3^ Within this field, the catalytic asymmetric multicomponent 1,2-boronate rearrangements,^4^ leading to highly valuable enantioenriched boronic esters, are especially attractive.^5^

Morken pioneered the catalytic asymmetric multicomponent 1,2-boronate rearrangement catalyzed by transition metals in 2016.^6^ Their group demonstrated that enantioselective 1,2-boronate rearrangements of alkenyl-boronates could be achieved with π-acidic late transition-metal complexes, such as Pd^II^ and Ni^II^, proceeding via an inner-sphere metal-induced migration pathway (pathway a, Scheme 1a).^7^ Related reactions involving alkenylboronates (or indoleboronates) with other electrophilic metal complexes, including π-allyl–Pd,^8^ π-allyl–Ir,^9^ and allenylidene–Cu,^10^ reported by Ready, Brown, and Gao, are believed to occur through an outer-sphere pathway (pathway b, Scheme 1a). These reactions result in the asymmetric difunctionalization of C=C π-bonds. More recently, our group extended the π-allyl–Ir-induced asymmetric multicomponent 1,2-boronate rearrangement to the difunctionalization of activated σ-bonds in ring-strained bicyclo[1.1.0]butyl boronate complexes.^11^ Song and co-workers employed the aryl–Ni complex to induce 1,2-boronate rearrangement of alkynyl boronate complexes, leading to the synthesis of axially chiral compounds.^12^

**Scheme 1:**
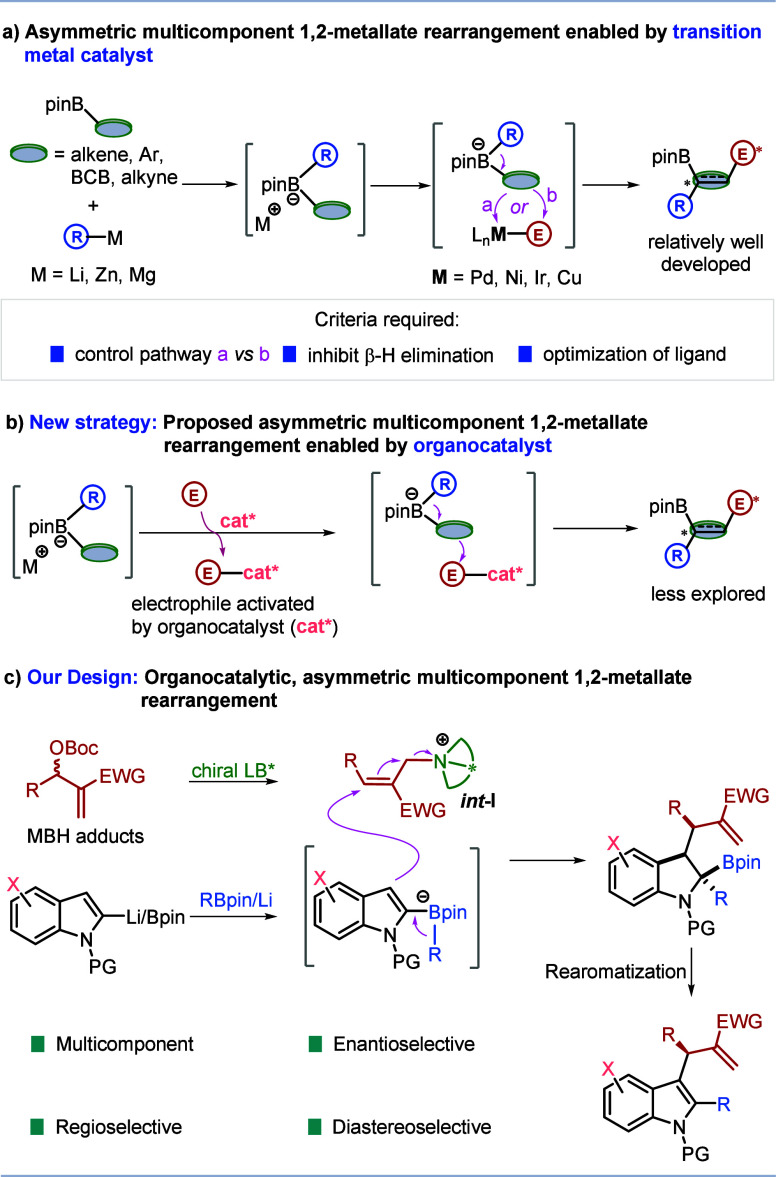
Asymmetric Multicomponent 1,2-Metallate Rearrangements and Our Reaction Design

As an alternative to transition-metal catalysis, we considered merging the fields of organocatalysis^13^ with 1,2-boronate rearrangements as this could offer new opportunities in asymmetric multicomponent reactions.^14,15^ In our design, we considered employing an organocatalyst to enhance the electrophilicity of one of the components, which would ultimately trigger a 1,2-boronate rearrangement (Scheme 1b). We were particularly attracted to the cationic intermediates *int***-I** (Scheme 1c) formed from Morita–Baylis–Hillman (MBH) carbonates^16,17^ and Lewis base catalysts^18^ as they had been shown to react with a broad range of nucleophiles.^19^ For successful enantioselective variants of this type of electrophile-nucleophile reaction, it is critical that the nucleophile displays nucleophilicity lower than that of the chiral Lewis base catalyst.^18^ This criterion aligns well with boronate complexes, which are known for their relatively weak nucleophilicity.^20^ Herein, we report our success in the development of a novel Lewis base-catalyzed asymmetric three-component coupling reaction of indoles, boronic esters, and MBH carbonates. This AMCR affords enantioenriched indoline derivatives featuring three contiguous vicinal chiral centers, including one quaternary chiral center. Enantioenriched indole and indoline derivatives are prevalent in natural products and bioactive molecules, and our method provides access to them with high enantio- and diastereoselectivity from basic feedstocks.^21^

We first investigated the reaction of MBH carbonate **3** with indole boronate complex **2a**, derived from *N*-Me-indole **1** and phenylboronic ester (Table 1). Typically, the boronic ester product **4** was oxidized to the corresponding highly substituted indole **5**, although the isolation of product **4** was also possible. We were pleased to find that the reaction was successful with chiral (DHQ)_2_AQN (**LB1**, 10 mol%) as the Lewis base catalyst in a mixture of THF-toluene to give indole **5** in 46% GC yield with 86:14 rr (rr = **5a**:**5a’**) and 94:6 er (entry 1). Without the Lewis base catalyst, only linear product **5a’** was obtained (entry 2). Other chiral Lewis base catalysts **LB2**-**LB5** were tested, and they gave the desired product but with lower regio- and enantioselectivity (entries 3–6). Solvent was found to have a considerable effect on the reaction (entries 7–11). Interestingly, toluene led to excellent reactivity and regioselectivity but poor enantioselectivity (entry 7), while THF provided the opposite results [high enantioselectivity with poor yield and poor regioselectivity (entry 8)] and a 1:1 mixture gave moderate reactivity, good regioselectivity, and high enantioselectivity (entry 1). Evaluation of different solvent mixtures with toluene showed that a mixture of 1,4-dioxane and toluene was optimal (entry 11). Finally, it was found that the combination of a higher concentration (0.2 M) and lower temperature (10 °C) was ideal, generating product **5** in 73% isolated yield with 98:2 rr and 96:4 er (entries 12–13). Notably, control experiments employing Pd- or Ir-catalysis^8,9^ for the reaction of indole boronate complex **2a** with MBH carbonate **3** gave only linear product **5a’** (see Supporting Information, Table S8), the same results as without the metal catalyst, suggesting that neither Pd nor Ir were participating in the reaction. These observations demonstrated the importance of Lewis base catalysis for enhancing and controlling both the reactivity and the selectivity in our reactions.

**Table 1:**
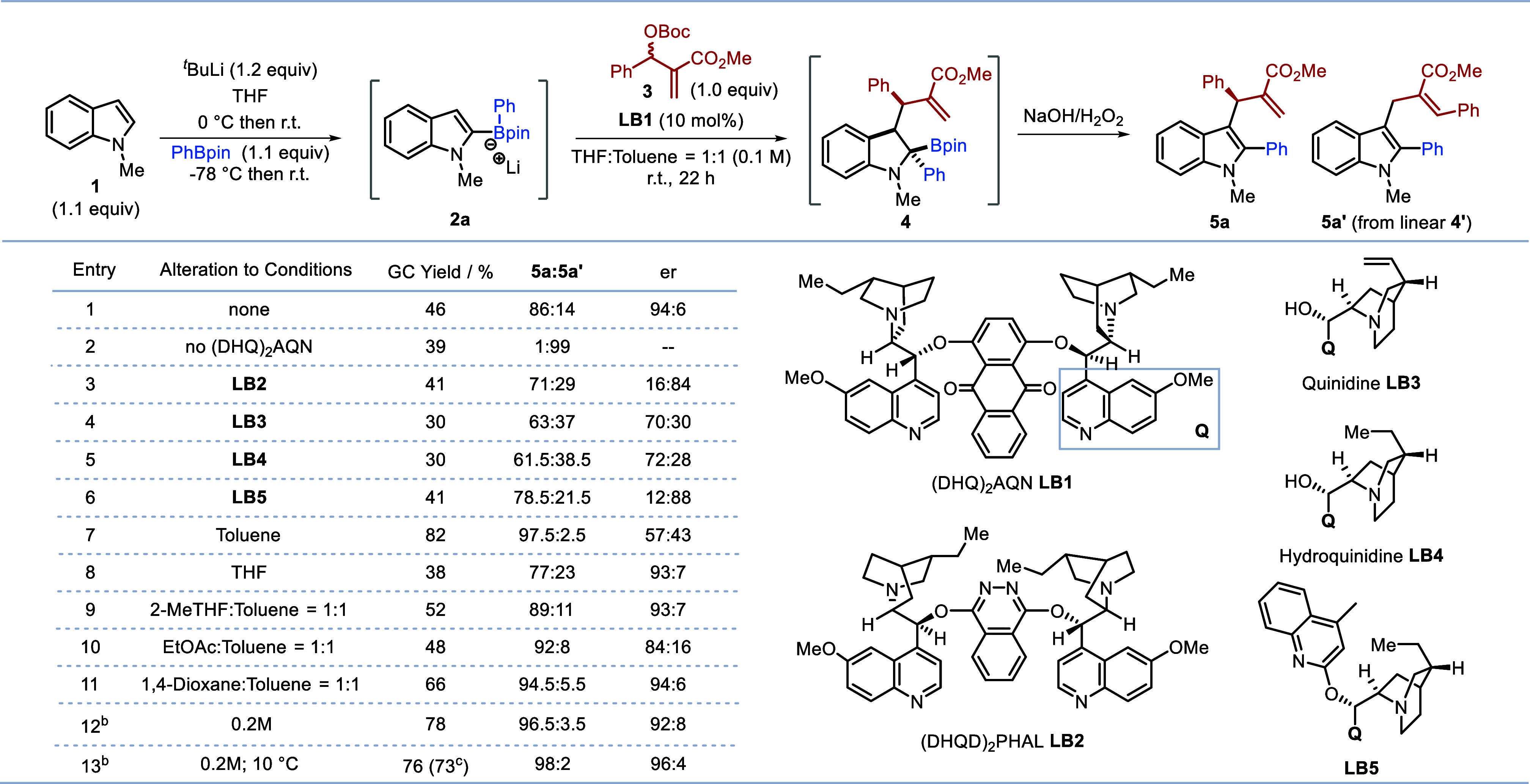
Reaction optimization^a^

a Reactions conducted on 0.2 mmol scale as shown in scheme; er was determined by HPLC analysis, rr was determined by GC analysis.

b 1,4-Dioxane:toluene = 1:1 as solvent.

c Isolated yield.

Having established the optimal conditions, we embarked on investigating the generality of the reaction (Scheme 2, top). We first evaluated the scope, with respect to the boronic ester. The reaction accommodated various substituents on the phenyl group, including both electron-donating and electron-withdrawing groups at the *para-*, *ortho-*, and *meta*- positions. This resulted in the formation of the corresponding 1,2,3-trisubstituted indole products **5b**-**5g** with yields ranging from 32%–77%, 90:10–98:2 rr, and up to 96:4 er. Of note, a sterically demanding substituent such as *o*-Me (**5f**) required a longer reaction time to achieve a good yield. Boronates derived from other aromatic rings, including naphthalene, thiophene, and indole, were also investigated, leading to the corresponding products (**5h**-**5j**) in good yields. Additionally, alkenyl boronic esters worked equally well, affording the indole products (**5k**-**5l**). Primary alkyl boronic esters (**5m**-**5n**) were also employed, giving good to excellent regio- and enantioselectivity, albeit in lower yield. However, neither secondary nor tertiary boronic esters were successful.^8a,10a,14^

**Scheme 2:**
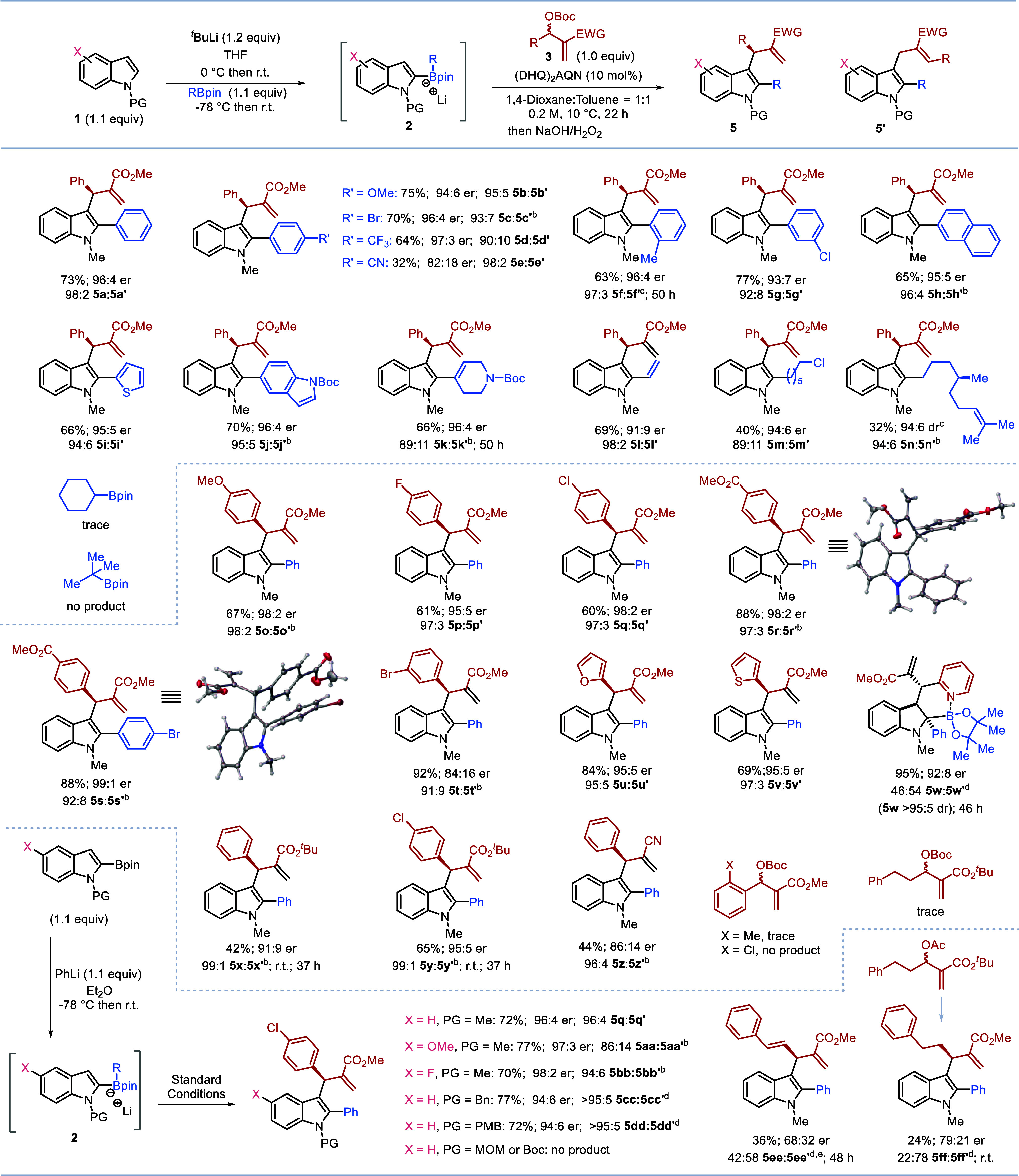
Substrate Scope Reactions conducted on 0.2 mmol scale as shown in scheme; yields are of isolated products; er was determined by HPLC analysis. The regioselectivity was determined by GC analysis. Determined by LC analysis. Determined by HPLC analysis. Determined by NMR analysis. 2.0 equiv of MBH carbonate was used.

**Scheme 3:**
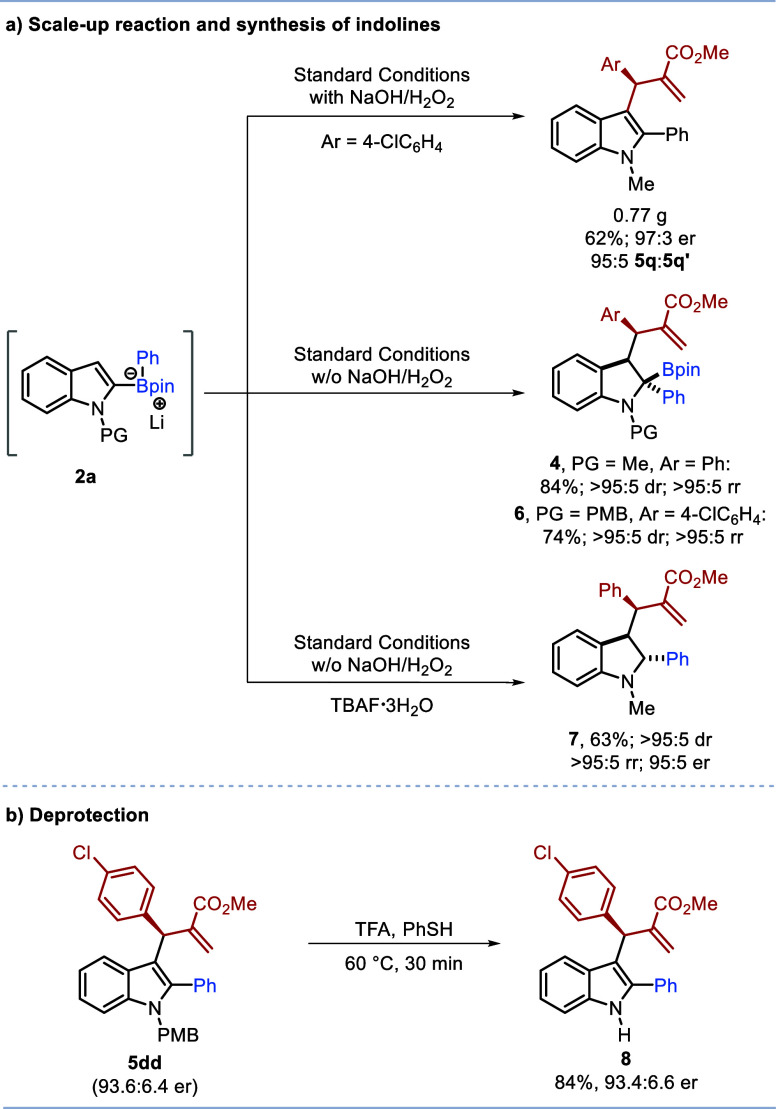
(a) Scale up and Synthesis of Indolines (b) Deprotection to Indole Nitrogen

We further expanded the reaction scope to various other racemic MBH carbonates (Scheme 2, center). We found that electron-rich and -deficient substitutions at the *para* position as well as *meta*-substituted aryl-MBH carbonates yielded good to excellent results with high levels of selectivity (**5o**-**5t**). However, sterically demanding *ortho*-substituted (*o*-Me and *o*-Cl) aryl-MBH carbonates were unreactive. Since *ortho* substituted aromatics are tolerated in related reactions with different nucleophiles,^19^ the poor reactivity observed must be because of the lower nucleophilicity of the indole boronate complexes. Heteroaromatics were also successfully employed, including thiophene- and furan-substituted MBH carbonates, leading to desired products **5u**-**5v** in good yields and selectivity. Interestingly, the pyridine-substituted carbonate furnished a highly substituted indoline product **5w** in 95% yield with 92:8 er and 46:54 branched/linear. The product **5w** was stable to mild oxidation and easy to isolate, and NMR analysis showed strong coordination of the pyridine nitrogen to boron (^11^B NMR δ = 7.9 ppm). Finally, MBH adducts derived from other activated alkenes (EWG = CO_2_^*t*^Bu, CN) were readily converted to the corresponding products **5x**-**5z**, again with good selectivity. We also observed that bulkier esters resulted in slower reactions but higher regioselectivities (**5x** vs **5a**, **5y** vs **5q**). The absolute configurations of enantioenriched products **5r** and **5s** were determined by X-ray crystallographic analysis.

**Scheme 4:**
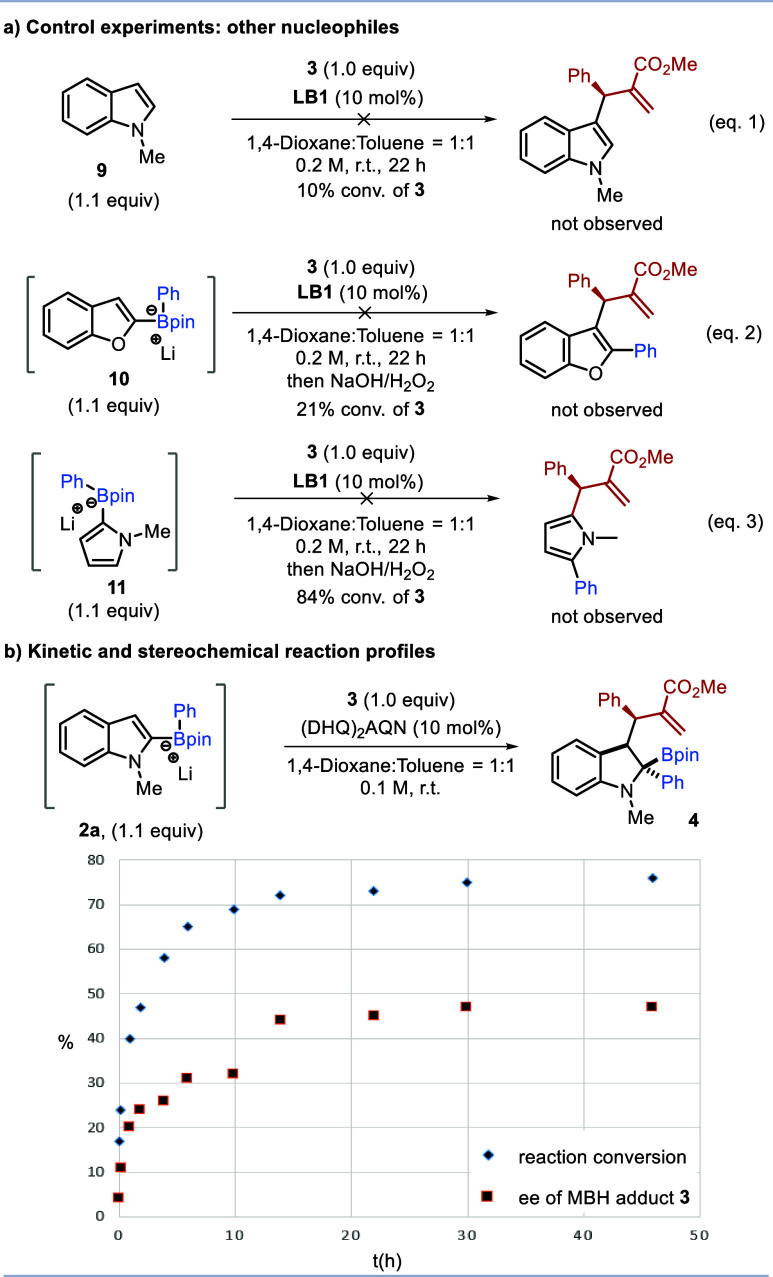
Mechanistic Investigations

Indole boronate complexes can also be accessed through the addition of organolithium reagents to indole boronic esters.^22^ We adopted this approach to further investigate the substitution effects on the indole moiety (Scheme 2, bottom). The boronate obtained from the reaction of phenyl lithium and 2-Bpin indole furnished product **5q** with comparable yield and selectivity compared to that derived from the reaction of indole lithium with phenyl boronic ester. This asymmetric multicomponent reaction also works well with other substituted indole boronic esters. Both 6-OMe and 6-F indole boronic esters provided products **5aa** and **5bb**, respectively, in good yields with high regio- and enantioselectivity. Four additional *N*-PG-indole boronic esters were evaluated in our reaction. The *N*-Bn-indole and *N*-PMB-indole boronic esters successfully yielded the desired products **5cc** and **5dd** in high yield with high selectivity. In contrast, the *N*-MOM-indole and *N*-Boc-indole boronic esters were unsuccessful. The alkenyl-MBH carbonate yielded product **5ee** in 36% yield with moderate enantioselectivity but poor regioselectivity. Finally, alkyl-MBH carbonates were explored, and while the Boc derivative was unsuccessful, the MBH acetate delivered product **5ff** in 24% yield, with moderate enantioselectivity but poor regioselectivity.^23^

The asymmetric multicomponent reaction could be scaled up (Scheme 3a) giving a similar yield of product **5q** (62%, 0.77 g) with similar selectivity (97:3 er and 95:5 rr). Furthermore, if oxidation is omitted, the highly substituted indoline products **4** and **6** could be obtained in high yield with excellent diastereo- and regioselectivity. Indeed, the very high diastereoselectivity of **4**, **5w**, and **6** suggests that this reaction most likely proceeds via a concerted 1,2-boronate migration/indole addition pathway (vide infra). In addition, if the product of the reaction is directly treated with TBAF, protodeboronation occurs to form enantioenriched indoline **7** with very high diastereo-, regio-, and enantioselectivity. The relative stereochemistry of indoline product **5w** was determined by NOESY (see Supporting Information for details), and **4**, **6**, and **7** are assigned by analogy. Finally, we were able to remove the PMB protecting group in product **5dd** using trifluoroacetic acid (TFA) in the presence of thiophenol (Scheme 3b) to give the unprotected indole **8**.^24^

We then performed a series of experiments to gain mechanistic insight into this asymmetric multicomponent reaction (Scheme 4). First, alternative nucleophiles were tested to examine which features were required for reactivity. These included N-Me-indole **9** without an attached boronate, replacing the indole with benzofuran boronate **10** and replacing the indole with N-Me-pyrrole boronate **11**.^25^ However, none of these nucleophiles yielded the desired products, indicating that both the indole and the boronate complexes are required to provide sufficient nucleophilicity to react with the Lewis-base-enhanced MBH electrophiles (Scheme 4a). We monitored the kinetic and stereochemical profiles of the reaction (Scheme 4b). The reaction exhibited an initial fast rate where the matched enantiomer of MBH carbonate was consumed approximately twice as fast as the mismatched enantiomer. Interestingly, after ∼70% conversion, the enantiomeric excess of recovered MBH carbonate **3** stabilized at around 50% ee. These observations suggest that, although this asymmetric multicomponent reaction is enantioconvergent, a match/mismatch effect still exists between the enantiomers of racemic MBH carbonate and the chiral Lewis base.^23c^

Based on prior research^19,26^ and our own observations, a proposed catalytic cycle for this reaction is illustrated in Scheme 5. The S_N_2’ addition of chiral Lewis base catalyst **LB1** to the MBH carbonate **3** generates the chiral cationic intermediate *int***-1** with *E* geometry.^26^ According to the established model,^19c^ as a result of π–π stacking between the quinoline ring and the (red) aromatic ring, the *Re* face is blocked and the nucleophile adds from the *Si* face, leading to enantiomer **4** as observed. The alkyl- and alkenyl-MBH adducts gave much lower ee values, presumably because of their reduced π–π interactions, consistent with this model. This model fits other examples using different nucleophiles and accounts for the experimentally determined enantioselectivities.^27^ To rationalize the diastereoselectivity, we propose that the nucleophile can approach in two different orientations: ***TS-1*** and ***TS-2***. It is proposed that ***TS-1*** is favored over ***TS-2*** since it benefits from stabilizing π–π interactions between the electron-rich indole and the Ph group as well as electrostatic interactions^28^ between the boronate anion and the ammonium cation, both of which are much reduced in ***TS-2***. Concerted *anti* addition of the migrating Ph group and displacement of the ammonium salt in an S_N_2’ manner then leads to the major diastereoisomer **4**. Finally, the linear product is believed to come from the direct S_N_2’ addition of **2a** to **3** (Table 1, entry 2), but it could also arise from an S_N_2 addition of **2a** to *int***-1**.

**Scheme 5:**
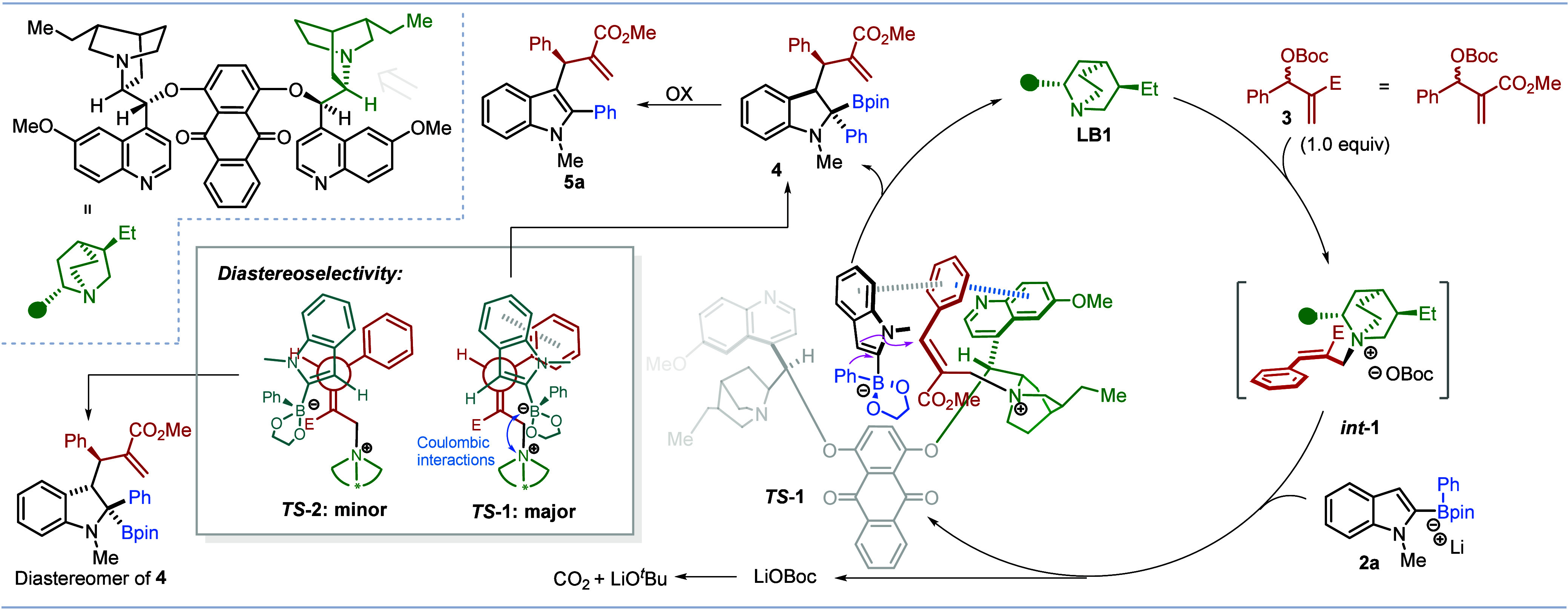
Catalytic Cycle

In conclusion, through merging the fields of organocatalysis with 1,2-metallate rearrangements, we have developed a novel asymmetric multicomponent 1,2-boronate rearrangement involving indole, boronic ester, and MBH carbonate, enabled by a chiral Lewis base catalyst. This approach provides a powerful method for the synthesis of highly substituted enantioenriched indole and indoline derivatives, expanding the chemical space of these important entities.

## Supporting Information Available

Experimental procedures and characterization data for new compounds (PDF)
