# Supplementary material for: Merging Organocatalysis with 1,2-Boronate Rearrangement: A Lewis Base-Catalyzed Asymmetric Multicomponent Reaction
Source: J Am Chem Soc. 2024 Sep 24;146(40):27305–11. doi: 10.1021/jacs.4c11113 (PMC11467900; doi:10.1021/jacs.4c11113)

Supplementary Information for

Merging Organocatalysis with 1,2-Boronate Rearrangement: a Lewis Base-Catalyzed Asymmetric Multicomponent Reaction

Hong-Cheng Shen, Varinder K. Aggarwal*

School of Chemistry, University of Bristol, Cantock's Close, Bristol, BS8 1TS, U.K.
Correspondence to: v.aggarwal@bristol.ac.uk

Table of Contents

1. Materials and general methods 4
1.1. Glassware, solvents and reagents 4
1.2. Instrumentation 4
1.3. Naming of compounds 5
2. Experimental data 6
2.1. Reaction optimization 6
2.2 Structure of product 5w 11
2.3. General procedures 13
2.3.1. General procedure A: synthesis of 5a-5z 13
2.3.2. General procedure B: synthesis of 5aa-5ff 14
2.3.3. General procedure C: synthesis of 4 and 5w 15
2.3.4. General procedure D: synthesis of 7 15
2.3.5. Procedure E: scale up reaction. 16
2.3.6. Procedure F: kinetic and stereochemical reaction profiles. 17
2.3.7. Procedure G: deprotection of 5dd. 18
2.4. Synthesis of starting materials 18
2.4.1. Synthesis of MBH adducts 18
2.4.2. Synthesis of noncommercially available boronic esters used in this project. 18
2.5. Characterization data for products. 19
methyl (*R*)-2-((1-methyl-2-phenyl-1H-indol-3-yl)(phenyl)methyl)acrylate (5a) 19
methyl 2-((*S*)-((2*S*,3*S*)-1-methyl-2-phenyl-2-(4,4,5,5-tetramethyl-1,3,2-dioxaborolan-2-yl)indolin-3-yl)(phenyl)methyl)acrylate (4) 19
methyl (*Z*)-2-((1-methyl-2-phenyl-1H-indol-3-yl)methyl)-3-phenylacrylate (5a') 20
methyl (*R*)-2-((2-(4-methoxyphenyl)-1-methyl-1H-indol-3-yl)(phenyl)methyl)acrylate (5b) 21
methyl (*R*)-2-((2-(4-bromophenyl)-1-methyl-1H-indol-3-yl)(phenyl)methyl)acrylate (5c) 22
Methyl (*R*)-2-((1-methyl-2-(4-(trifluoromethyl)phenyl)-1H-indol-3-yl)(phenyl)methyl) acrylate (5d) 22
methyl (*R*)-2-((2-(4-cyanophenyl)-1-methyl-1H-indol-3-yl)(phenyl)methyl)acrylate (5e) 24
methyl (*R*)-2-((1-methyl-2-(o-tolyl)-1H-indol-3-yl)(phenyl)methyl)acrylatehenyl (5f) 25
methyl (*R*)-2-((2-(3-chlorophenyl)-1-methyl-1H-indol-3-yl)(phenyl)methyl)acrylate (5g) 26
methyl (*R*)-2-((1-methyl-2-(naphthalen-2-yl)-1H-indol-3-yl)(phenyl)methyl)acrylate (5h) 27
methyl (*R*)-2-((1-methyl-2-(thiophen-2-yl)-1H-indol-3-yl)(phenyl)methyl)acrylate (5i) 28
tert-butyl (*R*)-3-(2-(methoxycarbonyl)-1-phenylallyl)-1-methyl-1H,1'H-[2,5'-biindole]-1'-carboxylate (5j) 28
tert-butyl (*R*)-4-(3-(2-(methoxycarbonyl)-1-phenylallyl)-1-methyl-1H-indol-2-yl)-3,6-dihydropyridine-1(2H)-carboxylate (5k) 29
methyl (*R*)-2-((1-methyl-2-vinyl-1H-indol-3-yl)(phenyl)methyl)acrylate (5l) 31
methyl (*R*)-2-((2-(6-chlorohexyl)-1-methyl-1H-indol-3-yl)(phenyl)methyl)acrylate (5m) 32
methyl 2-((*R*)-(2-((*R*)-4,8-dimethylnon-7-en-1-yl)-1-methyl-1H-indol-3-yl)(phenyl) methyl)acrylate (5n) 32
methyl (*R*)-2-((4-methoxyphenyl)(1-methyl-2-phenyl-1H-indol-3-yl)methyl)acrylate (5o) 34
methyl (*R*)-2-((4-fluorophenyl)(1-methyl-2-phenyl-1H-indol-3-yl)methyl)acrylate (5p) 35
methyl (*R*)-2-((4-chlorophenyl)(1-methyl-2-phenyl-1H-indol-3-yl)methyl)acrylate (5q) 36

methyl (*R*)-4-(2-(methoxycarbonyl)-1-(1-methyl-2-phenyl-1H-indol-3-yl)allyl)benzoate (5r) ...36
methyl (*R*)-4-(1-(2-(4-bromophenyl)-1-methyl-1H-indol-3-yl)-2-(methoxycarbonyl)allyl) benzoate (5s)...37
methyl (*R*)-2-((3-bromophenyl)(1-methyl-2-phenyl-1H-indol-3-yl)methyl)acrylate (5t)...39
methyl (*R*)-2-(furan-2-yl(1-methyl-2-phenyl-1H-indol-3-yl)methyl)acrylate (5u)...39
methyl (*R*)-2-((1-methyl-2-phenyl-1H-indol-3-yl)(thiophen-2-yl)methyl)acrylate (5v) ...40
methyl 2-((5a*S*, 12*R*)-4',4',5,5',5'-pentamethyl-5a-phenyl-5,5a,12,12a-tetrahydro-6l4,7l4-spiro[pyrido[1',2':1,6][1,2]azaborinino[3,4-b]indole-6,2'-[1,3,2]dioxaborolan]-12-yl)acrylate (5w) ...41
tert-butyl (*R*)-2-((1-methyl-2-phenyl-1H-indol-3-yl)(phenyl)methyl)acrylate (5x)...42
tert-butyl (*R*)-2-((4-chlorophenyl)(1-methyl-2-phenyl-1H-indol-3-yl)methyl)acrylate (5y) ...43
(*R*)-2-((1-methyl-2-phenyl-1H-indol-3-yl)(phenyl)methyl)acrylonitrile (5z)...44
methyl (*R*)-2-((4-chlorophenyl)(5-methoxy-1-methyl-2-phenyl-1H-indol-3-yl)methyl) ...45
acrylate (5aa)...45
methyl (*R*)-2-((4-chlorophenyl)(5-fluoro-1-methyl-2-phenyl-1H-indol-3-yl)methyl) acrylate (5bb) ...46
methyl (*R*)-2-((1-benzyl-2-phenyl-1H-indol-3-yl)(4-chlorophenyl)methyl)acrylate (5cc)...47
methyl (*R*)-2-((4-chlorophenyl)(1-(4-methoxybenzyl)-2-phenyl-1H-indol-3-yl)methyl)acrylate (5dd)...48
methyl (*R*,*E*)-3-(1-methyl-2-phenyl-1H-indol-3-yl)-2-methylene-5-phenylpent-4-enoate (5ee)..49
methyl (*R*)-3-(1-methyl-2-phenyl-1H-indol-3-yl)-2-methylene-5-phenylpentanoate (5ff)...50
methyl 2-((*S*)-(4-chlorophenyl)((2*R*,3*R*)-1-(4-methoxybenzyl)-2-phenyl-2-(4,4,5,5-tetramethyl-1,3,2-dioxaborolan-2-yl)indolin-3-yl)methyl)acrylate (6) ...51
methyl 2-((*S*)-((2*S*,3*S*)-1-methyl-2-phenylindolin-3-yl)(phenyl)methyl)acrylate (7) ...52
methyl (*R*)-2-((4-chlorophenyl)(2-phenyl-1H-indol-3-yl)methyl)acrylate (8) ...53
methyl 2-((1-methyl-1H-pyrrol-2-yl)(phenyl)methyl)acrylate (12)...54
2.6. Crystallography...55
3. Reference...57
4. NMR Spectra...58

1. MATERIALS AND GENERAL METHODS

1.1. Glassware, solvents and reagents

All manipulations were performed with oven-dried (130 °C for a minimum of 12 h) or flame-dried glassware using standard Schlenk techniques under an atmosphere of nitrogen, unless otherwise stated.

All anhydrous solvents were commercially supplied or dried using an Anhydrous Engineering alumina column drying system (dichloromethane, toluene, diethyl ether, and tetrahydrofuran). Reagents were purchased from commercial sources and used as received. All organolithium reagents were titrated against *N*-benzylbenzamide.[1]

1.2. Instrumentation

Thin layer chromatography (TLC) was performed using Merck Kieselgel 60 F254 fluorescent treated silica, which was visualized under UV light, or by staining with aqueous basic potassium permanganate followed by heating, or Hanessian's stain (CAM stain) followed by heating, or *p*-anisaldehyde solution followed by heating, as stated.

Flash column chromatography (FCC) was carried out using Sigma-Aldrich silica gel (60 Å, 230-400 mesh, 40-63 µm), Biotage Isolera™ flash purification system or boric acid impregnated silica gel.[2] In cases where automated column chromatography was employed the solvent gradient and flow rate are indicated.

NMR spectra were recorded at various field strengths, as indicated, using Bruker 400 MHz, Varian VNMR 400 MHz, Bruker Cryo 500 MHz or Bruker Cryo 600 MHz for ^{1}H, and ^{13}C acquisitions. All NMR spectra were recorder at 25 °C unless otherwise stated. Chemical shifts (δ) are reported in parts per million (ppm) and referenced to $CDCl_3$ (^{1}H: 7.26 ppm; ^{13}C: 77.16 ppm). Coupling constants (J) are given in Hertz (Hz) and refer to apparent multiplicities (s = singlet, d = doublet, t = triplet, q = quartet, quin = quintet, hex = hextet, h = heptet, m = multiplet, brs = broad signal, dd = doublet of doublets, etc.). The ^{1}H NMR spectra are reported as follows: chemical shift (multiplicity, coupling constants, number of protons).

HPLC analyses were performed on Agilent 1100 system with Daicel Chiralpak columns.

High resolution mass spectra (HRMS) were recorded on a Bruker Daltonics MicrOTOF II by Electrospray Ionisation (ESI); a Thermo Scientific QExactive by Electron Ionisation (EI); a Thermo Scientific Orbitrap Elite by ESI or Atmospheric Pressure Chemical Ionisation (APCI); or a Bruker UltrafleXtreme by Matrix-assisted Laser Desorption/Ionisation (MALDI).

IR spectra were recorded neat as a thin film on a Perkin Elmer Spectrum One FT-IR. Selected absorption maxima (ν_{max}) are reported in wavenumbers (cm^{-1}).

Gas chromatography–mass spectrometry (**GC-MS**) was recorded on an Agilent 6890 Series GC and 5973 detectors using a HP-5MS UI column (15 m × 0.25 mm × 0.25 μm).

1.3. Naming of compounds

Compound names are those generated by ChemDraw Professional 20.0 software (PerkinElmer), following the IUPAC nomenclature.

2. EXPERIMENTAL DATA

2.1. Reaction optimization

Table S1: Background reaction.

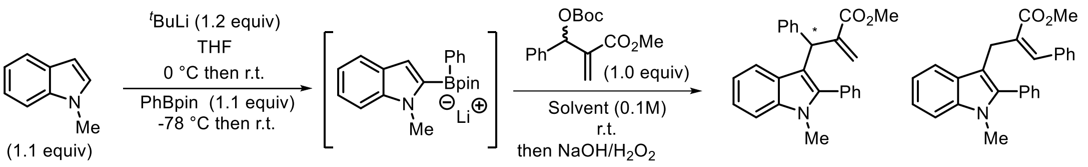

Entry	Solvent	GC Yield/%[a]	rr[a]
1	THF	31	1:99
2	Toluene	21	1:99
3	THF/Toluene = 1/1	39	1:99
4	THF/Toluene = 1/1; 0 °C	N. R.	--
5	THF/Toluene = 1/1; 0 °C; $(DHQ)_2AQN$	3	--

a: Determined by GC

Table S2: Solvent.

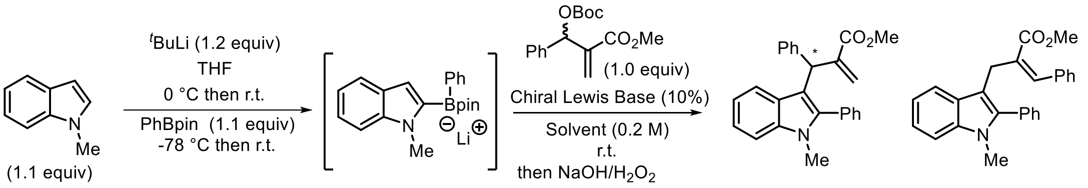

Entry	Chiral Lewis Base	Solvent	GC Yield/%[a]	rr[a]	er[b]
1	$(DHQ)_2Pyr$	EtOAC	41	92.5:7.5	36:64
2	$(DHQ)_2Pyr$	1,4-dioxane	40	92.5:7.5	19:81
3	$(DHQ)_2Pyr$	DCM	69	97.5:2.5	40:60
4	$(DHQ)_2Pyr$	Toluene	85	99:1	62:38
5	$(DHQ)_2Pyr$	THF	41	77:23	22:78
6	$(DHQ)_2Pyr$	Et_2O/Toluene =1/1	77	97.5:2.5	44:56

a: Determined by GC; b: Determined by HPLC;

Observation: 1) The indole boronate complex is insoluble in reaction where Et_2O I used as a solvent. 2) The melting point of 1,4-dioxane is 11.8 ℃. Reactions using 1,4-dioxane as solvent at low temperatures need to be mixed with other solvents.

Table S3: Lewis base catalysts with toluene.

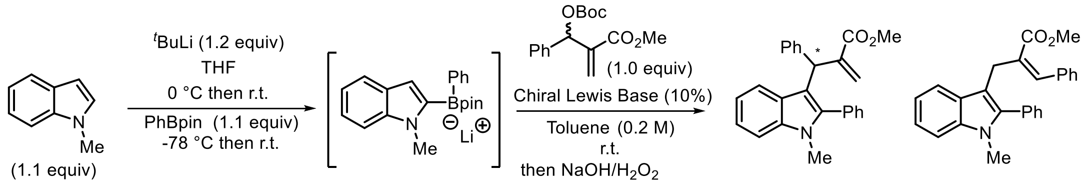

Entry	Chiral Lewis Base	Solvent	GC Yield/%[a]	rr[a]	er[b]
1	$(DHQ)_2$PHAL	Toluene	28	96.5:3.5	75:25
2	$(DHQ)_2$Pyr	Toluene	85	99:1	62:38
3	$(DHQ)_2$AQN	Toluene	82	97.5:2.5	43:57
4	$(DHQ)_2$Pyr	Toluene (0.1 M)	86	99:1	62:38
5	$(DHQ)_2$Pyr	Toluene (- 20 °C)	66	>99:1	63:37
6	$(DHQ)_2$AQN	Toluene (- 20 °C)	26	98.5:1.5	10:90

a: Determined by GC; b: Determined by HPLC;

Table S4: Other Lewis base catalysts.

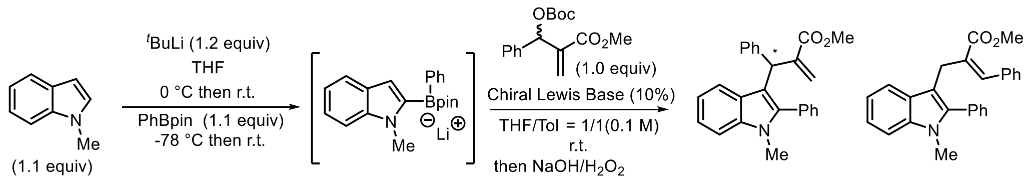

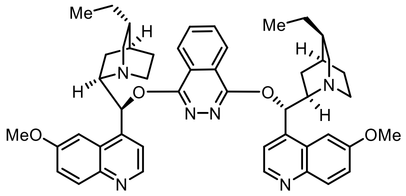

$(DHQD)_2$PHAL
41%; 71:29 b:l; 84:16 er

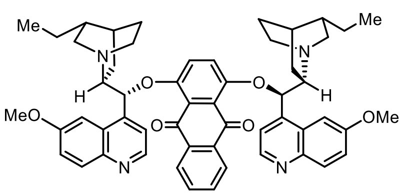

$(DHQ)_2$AQN
46%; 86:14 b:l; 6:94 er

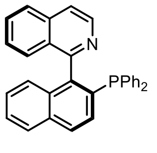

22%; 1:99 b:l; -- er

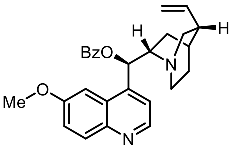

Bz-Quinine
30%; 19:81 b:l; 47:53 er

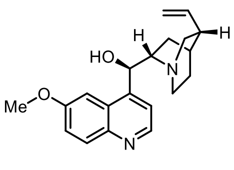

Quinine
31%; 50:50 b:l; 88:12 er

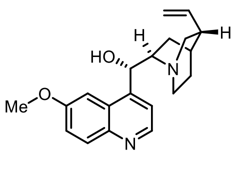

Quinidine
30%; 63:37 b:l; 30:70 er

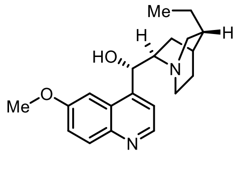

Hydroquinidine
30%; 61.5:38.5 b:l; 28:72 er

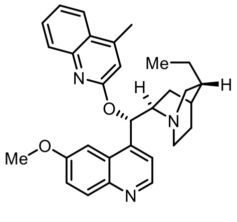

DHQD
41%; 78.5:21.5 b:l; 88:12 er

Table S5: Other parameters.

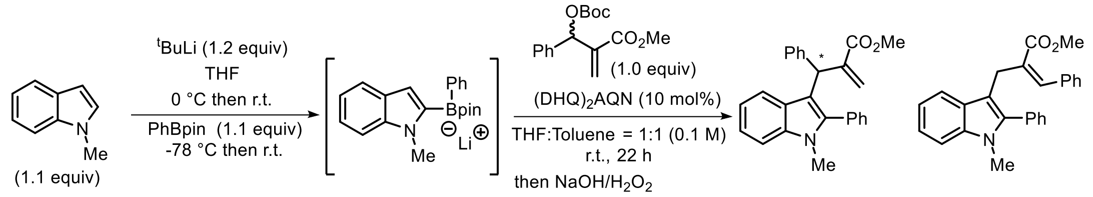

Entry	Conditions	GC Yield/%[a]	rr[a]	er[b]
1	none	46	86:14	94:6
2	PG = Ac	42	62:38	95:5
3	^{t}BuOMe:Toluene = 1:1	50	95:5	52:48
4	2-MeTHF:Toluene = 1:1	52	89:11	93:7
5	EtOAc:Toluene = 1:1	48	92:8	84:16
6	1,4-dioxane:Toluene = 1:1	66	94.5:5.5	94:6
7	1,4-dioxane:Toluene = 3:1	72	94.5:5.5	93:7
8	1,4-dioxane:Toluene = 1:3	37	94:6	85:15
9	1,4-dioxane:Toluene = 1:1; 0.2M	78	96.5:3.5	92:8

a: Determined by GC; b: Determined by HPLC

Table S6 Synthesis of *ent*-**5a**

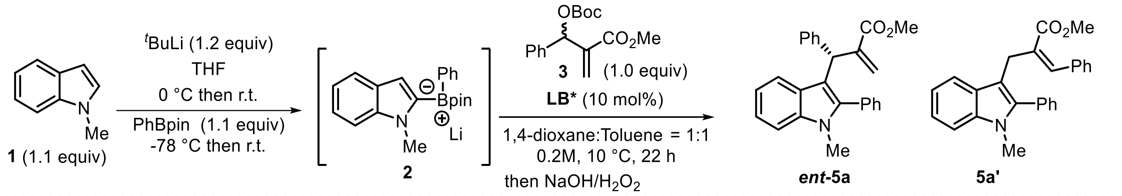

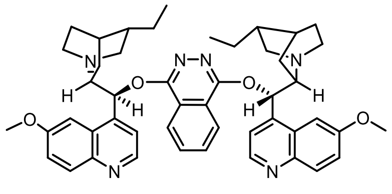

$(DHQD)_2PHAL$

72%, 92.5:7.5 *ent*-**5a**:**5a'**
52:48 er of *ent*-**5a**

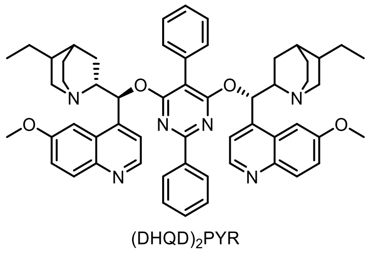

$(DHQD)_2PYR$

67%, 95:5 *ent*-**5a**:**5a'**
79:21 er of *ent*-**5a**

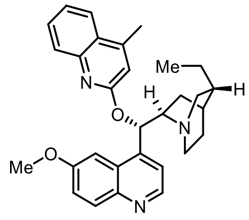

67%, 91:9 *ent*-**5a**:**5a'**
69:31 er of *ent*-**5a**

Isolated yields were given; rr was determined by GC analysis; er was determined by HPLC analysis.

Table S7: Unsuccessful photoredox functionalization.[4]

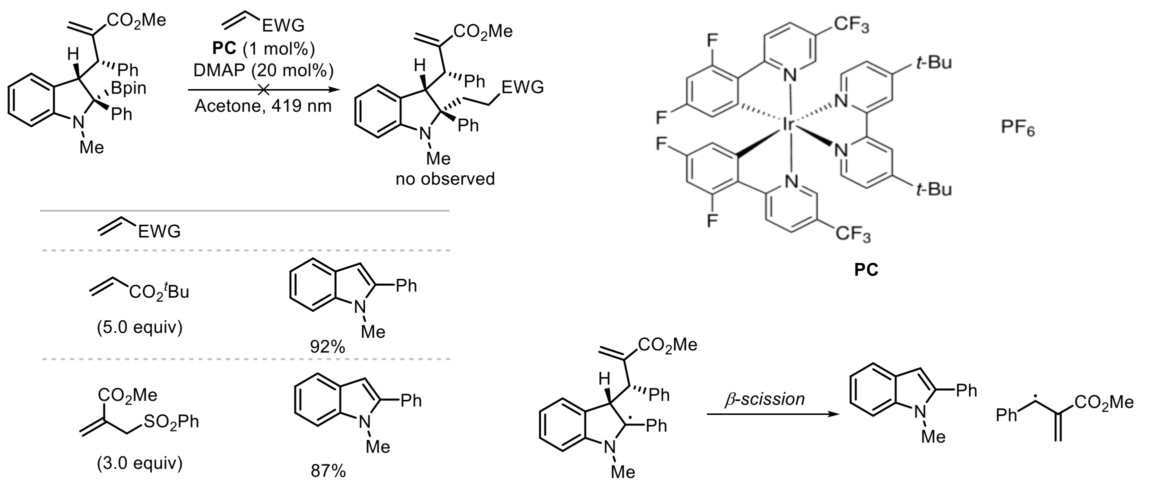

Table S8: Control Experiments: Pd and Ir catalysis.

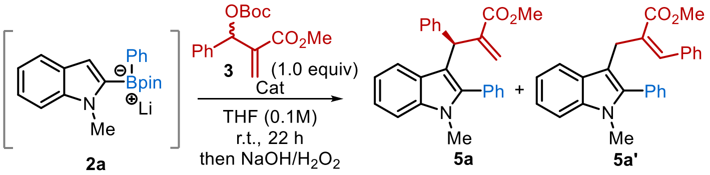

Conditions	5a:5a'	Yield
**LB1** (10 mol%), 1,4-dioxane:Toluene = 1:1, 0.2M, 10 °C	98:2	73% isolated yield
Cat = $PdCl_2$ (10 mol%), *rac*-BINAP (12 mol%)	1:99	36% NMR yield
Cat = $[Ir(cod)Cl]_2$ (2.5 mol%), (*S*)-(P, olefin)-**L*** (10 mol%)	1:99	33% NMR yield
without catalyst	1:99	33% isolated yield

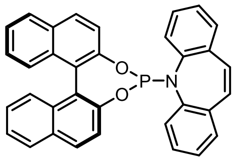

(*S*)-(P, olefin)-**L***

Control experiments employing Pd- or Ir-catalysis for the reaction of indole boronate complex **2a** with MBH carbonate **3** gave only linear product **5a'**, the same as with no catalyst, suggesting that neither Pd- nor Ir were participating species. These observations demonstrated the importance of Lewis base catalysis for enhancing and controlling reactivity and selectivity in our reactions.

Table S9: Substrates with OAc as leaving group.

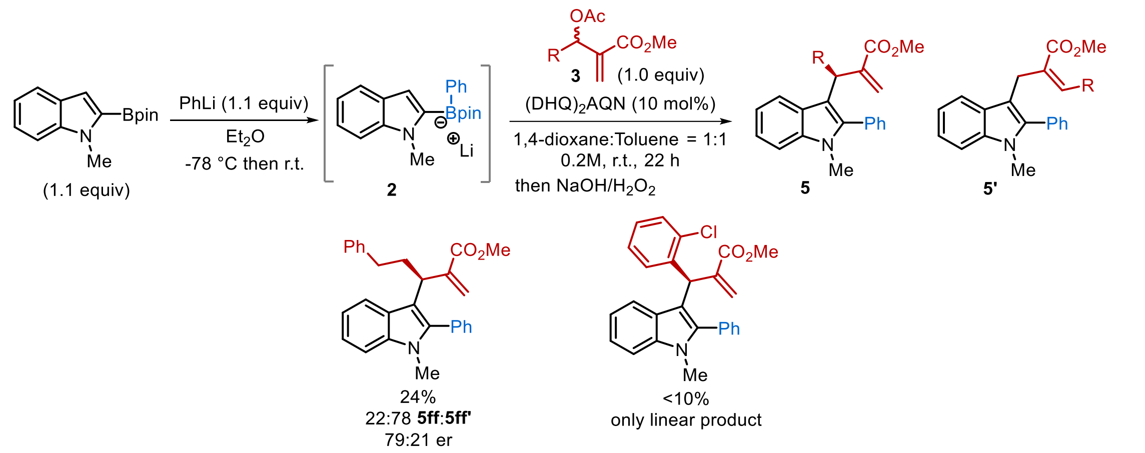

2.2 Structure of product 5w

^{11}Boron NMR of **5w**

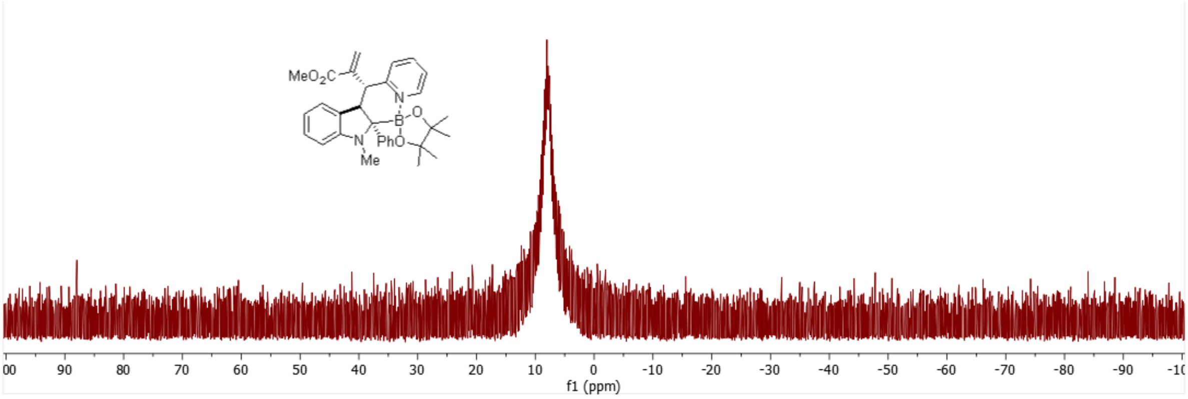

H, H-COSY of **5w**

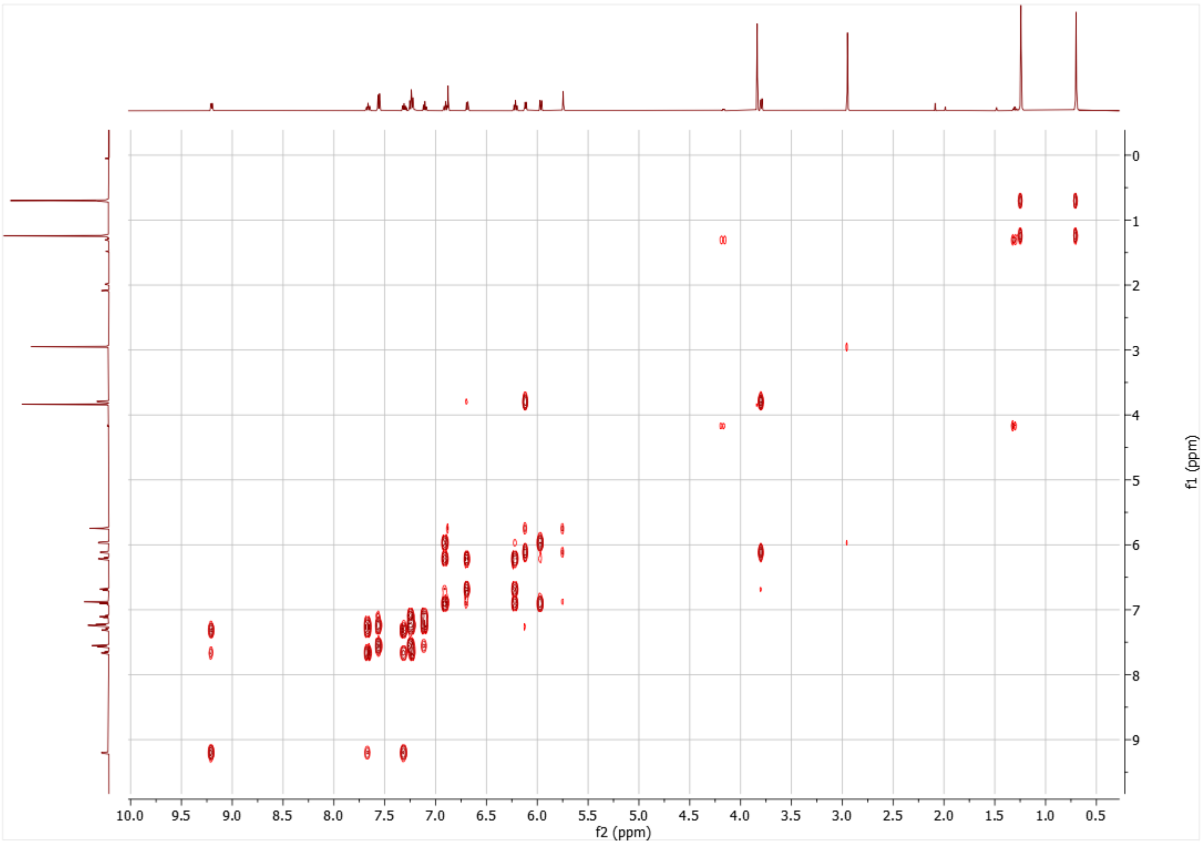

1H NMR assignment of **5w**

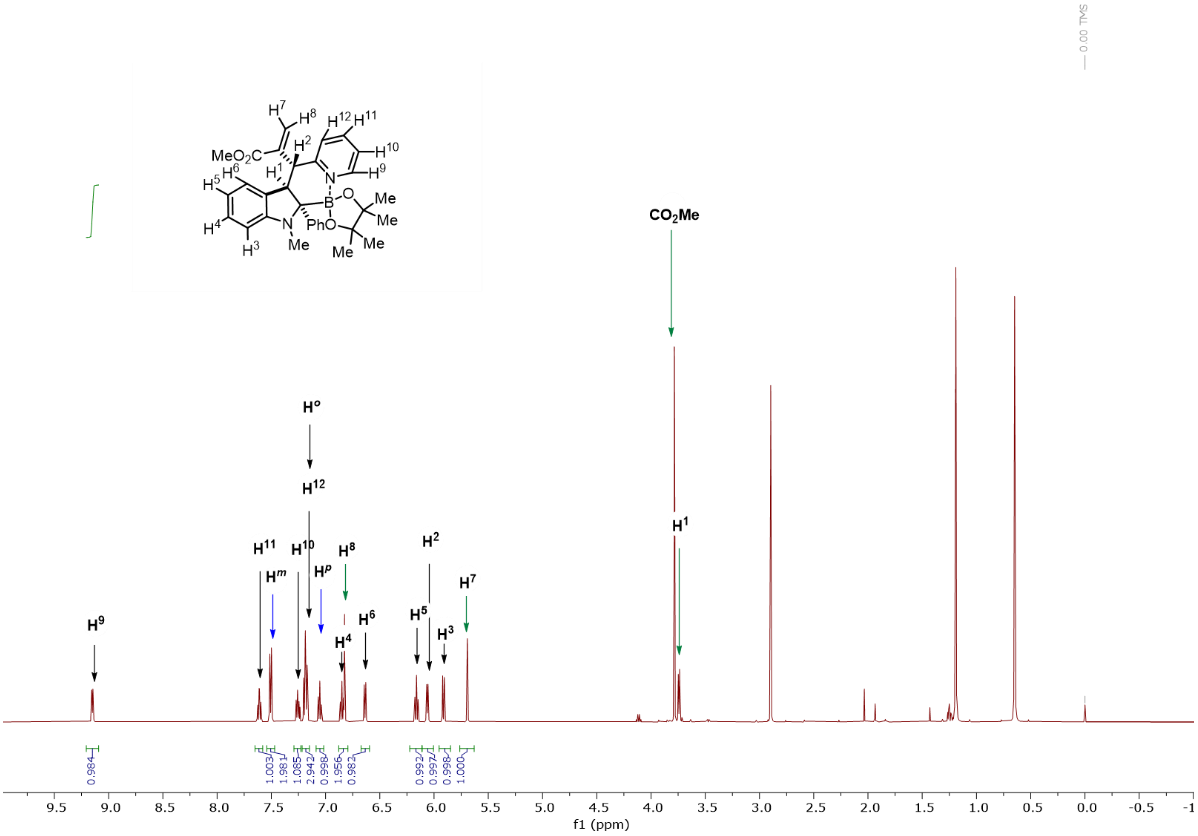

2D NOE of **5w**

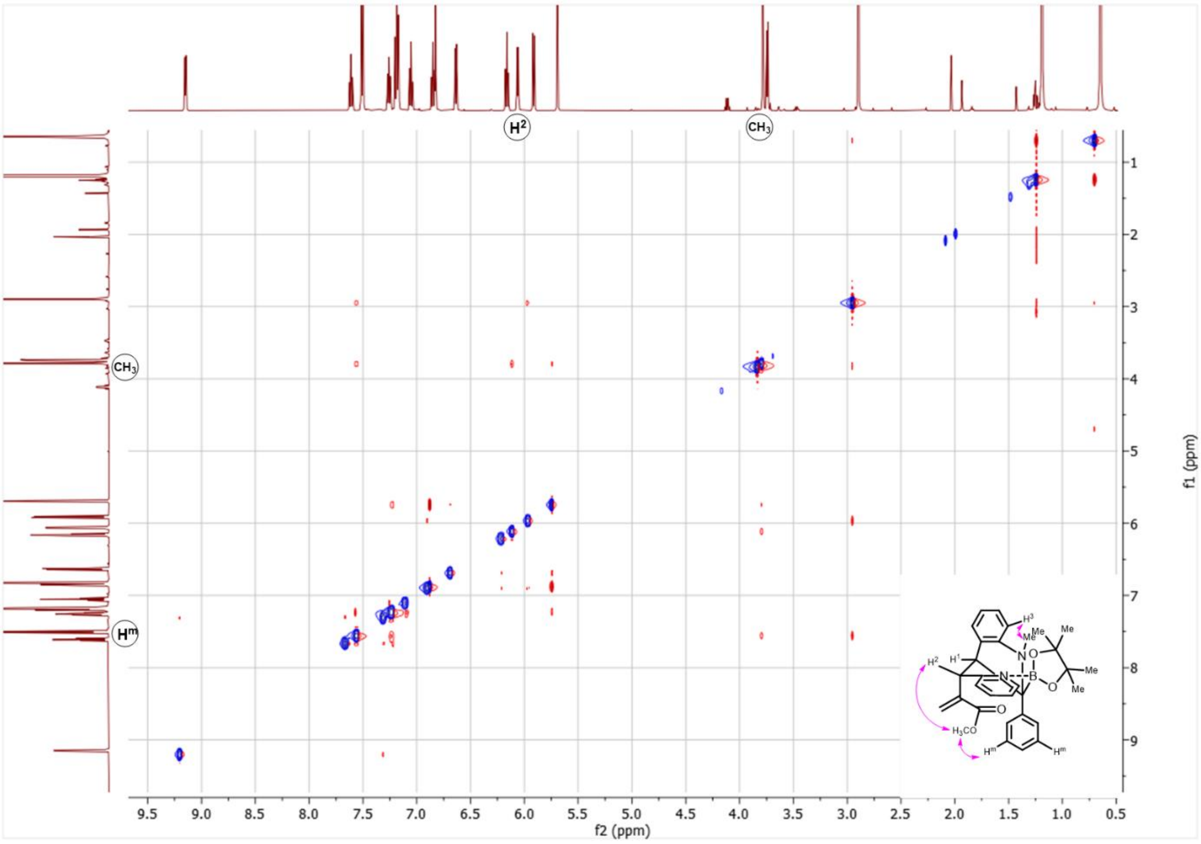

2.3. General procedures

2.3.1. General procedure A: synthesis of 5a-5z

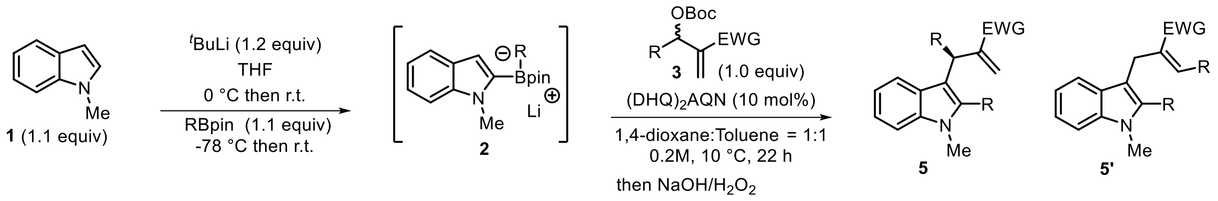

tert-Butyl lithium (in pentane, 0.24 mmol, 1.2 equiv) was added dropwise to a 20 mL Schlenk tube containing solution of N-Me-indole **1** (0.44 mL of 0.5M **1** in THF was used, 0.22 mmol, 1.10 equiv) in anhydrous THF (1 mL) at 0 °C (ice bath). The reaction mixture was stirred at 0 °C for 5 min. Subsequently, the reaction was removed from the cooling bath and warmed to ambient temperature. After stirring for 1 hour, the reaction mixture was cooled to -78 °C (dry ice/acetone bath). A solution of boronic ester (0.22 mmol, 1.1 equiv) in anhydrous THF was added dropwise. The reaction mixture was stirred at -78 °C for 5 min. Subsequently, the reaction was removed from the cooling bath and warmed to ambient temperature. After stirring for 30 min, the solvent was removed under high vacuum and refilled with N_2. Next, MBH carbonate **3** (0.2 mmol, 1.0 equiv) and $(DHQ)_2$AQN (17.2mg, 0.02 mmol, 10 mol%) were added quickly under a positive pressure of nitrogen. After backfilling with N_2 3 times, a 1 mL mixture of 1,4-dioxane and toluene (1/1 v/v) was added. The reaction flask was sealed and stirred at 10 °C for 22 h. After cooling to 0 °C, the resulting solution was diluted with THF (1.0 mL). The reaction solution was treated with NaOH (3 M aqueous solution, 1 mL) followed by the dropwise addition of H_2O_2 (30% aqueous solution, 0.5 mL) at 0 °C. It was allowed to stir at room temperature for 1-3 h, and then quenched with saturated aqueous $Na_2S_2O_3$ (1 mL) at 0 °C. After stirring for 10 min at ambient temperature, the mixture was extracted with Et_2O (3 x 10 mL). The combined organic layers were dried using Na_2SO_4, filtered, and concentrated in vacuo. The residue was purified by flash column chromatography on silica gel to afford the desired product.

Observations: 1) When the ArOH byproduct, from the oxidation of ArBpin does not separate well from the desired product, triethylamine was added to the solvent eluent to improve

separation during flash column chromatography. 2) Prepared TLC plate was used to separate the two regiomers, using a solvent mixture of 20% DCM in toluene.

2.3.2. General procedure B: synthesis of 5aa-5ff

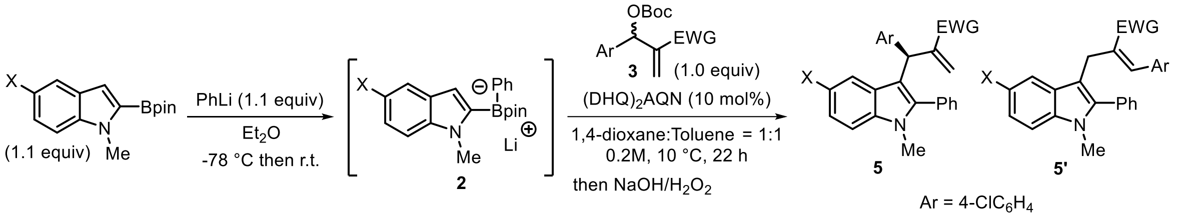

Phenyl lithium (in dibutyl ether, 0.22 mmol, 1.1 equiv) was added dropwise to a 20 mL Schlenk tube containing solution of indole boronic ester (0.22 mmol, 1.10 equiv) in anhydrous Et_2O (1 mL) at -78 °C (dry ice/acetone bath). Subsequently, the reaction was removed from the cooling bath and warmed to ambient temperature. After stirring for 30 min, the solvent was removed under high vacuum and refilled with N_2. Next, MBH carbonate **3** (65.2 mg, 0.2 mmol, 1.0 equiv) and $(DHQ)_2AQN$ (17.2mg, 0.02 mmol, 10 mol%) were added quickly under a positive pressure of nitrogen. The reaction flask was sealed, evacuated, and refilled with N_2 3 times. A 1 mL mixture of 1,4-dioxane and toluene (1/1 v/v) was then added. The reaction was stirred at 10 °C for 22 h. After cooling to 0 °C, the resulting solution was diluted with THF (1.0 mL). The reaction mixture was treated with NaOH (3 M aqueous solution, 1 mL) followed by the dropwise addition of H_2O_2 (30% aqueous solution, 0.5 mL) at 0 °C. It was allowed to stir at ambient temperature for 1-3 h, and then quenched with saturated aqueous $Na_2S_2O_3$ (1 mL) at 0 °C. After stirring for 10 min at ambient temperature, the mixture was extracted with Et_2O (3 x 10 mL). The combined organic layers were dried using Na_2SO_4, filtered, and concentrated in vacuo. The residue was purified by flash column chromatography on silica gel to afford the desired product.

Observation: In certain cases, a solvent change is required for successful oxidation. For these examples, the reaction mixture is passed through a silica gel (2 cm) filter, washed with Et_2O (3 x 10 mL), and concentrated in vacuo. The oxidation is then performed using $NaOH/H_2O_2$ in THF. This procedure applies to examples such as **5cc** and **5dd**.

2.3.3. General procedure C: synthesis of 4 and 5w

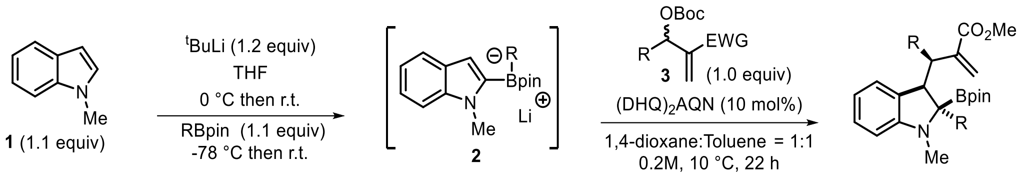

tert-Butyl lithium (in pentane, 0.24 mmol, 1.2 equiv) was added dropwise to a 20 mL Schlenk tube containing a solution of N-Me-indole **1** (0.44 mL of 0.5M **1** in THF was used, 0.22 mmol, 1.10 equiv) in anhydrous THF (1 mL) at 0 °C (ice bath). The reaction mixture was stirred at 0 °C for 5 min. Subsequently, the reaction was removed from the cooling bath and warmed to ambient temperature. After stirring for 1 hour, the reaction mixture was cooled to -78 °C (dry ice/acetone bath). A solution of boronic ester (0.22 mmol, 1.1 equiv, 0.5 M in THF) was added dropwise. The reaction mixture was stirred at -78 °C for 5 min. Subsequently, the reaction was removed from the cooling bath and warmed to ambient temperature. After stirring for 30 min, the solvent was removed under high vacuum and refilled with N_2. Next, MBH carbonate **3** (0.2 mmol, 1.0 equiv) and $(DHQ)_2AQN$ (17.2 mg, 0.02 mmol, 10 mol%) were added quickly under a positive pressure of nitrogen. After backfilling with N_2 3 times, a 1 mL mixture of 1,4-dioxane and toluene (1/1 v/v) was added. The reaction flask was sealed and stirred at 10 °C for 22 h. The resulting mixture was warmed to room temperature, filtered through a silica plug eluting with diethyl ether, and concentrated under reduced pressure. The crude product was purified by flash column chromatography on silica gel to afford the desired product.

2.3.4. General procedure D: synthesis of 7

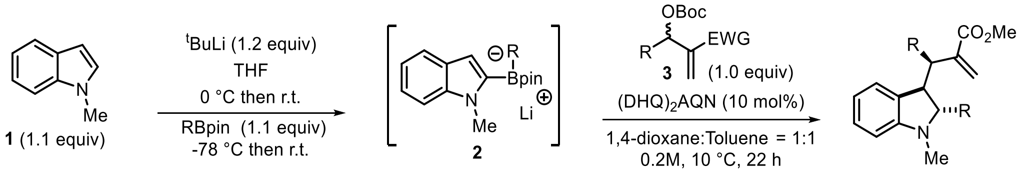

tert-Butyl lithium (in pentane, 0.24 mmol, 1.2 equiv) was added dropwise to a 20 mL Schlenk tube containing a solution of N-Me-indole **1** (0.44 mL of 0.5M **1** in THF was used, 0.22 mmol, 1.10 equiv) in anhydrous THF (1 mL) at 0 °C (ice bath). The reaction mixture was stirred at

0 °C for 5 min. Subsequently, the reaction was removed from the cooling bath and warmed to ambient temperature. After stirring for 1 hour, the reaction mixture was cooled to -78 °C (dry ice/acetone bath). A solution of boronic ester (0.22 mmol, 1.1 equiv, 0.5 M in THF) was added dropwise. The reaction mixture was stirred at -78 °C for 5 min. Subsequently, the reaction was removed from the cooling bath and warmed to ambient temperature. After stirring for 30 min, the solvent was removed under high vacuum and refilled with N_2. Next, MBH carbonate **3** (0.2 mmol, 1.0 equiv) and $(DHQ)_2AQN$ (17.2mg, 0.02 mmol, 10 mol%) were added quickly under a positive pressure of nitrogen. After backing with N_2 3 times, a 1 mL mixture of 1,4-dioxane and toluene (1/1 v/v) was added. The reaction flask was sealed and stirred at 10 °C for 22 h. The resulting mixture was moved into a glovebox, and TBAF·$3H_2O$ (95 mg, 3.0 mmol, 15 equiv) and 1 mL THF were added. The reaction flask was sealed, moved out of the glovebox, and stirred at 55 °C for 14 h. The resulting mixture was cooled to room temperature, filtered through a silica plug, eluting with diethyl ether, and concentrated under reduced pressure. The crude product was purified by flash column chromatography on silica gel to afford the desired product.

2.3.5. Procedure E: scale up reaction.

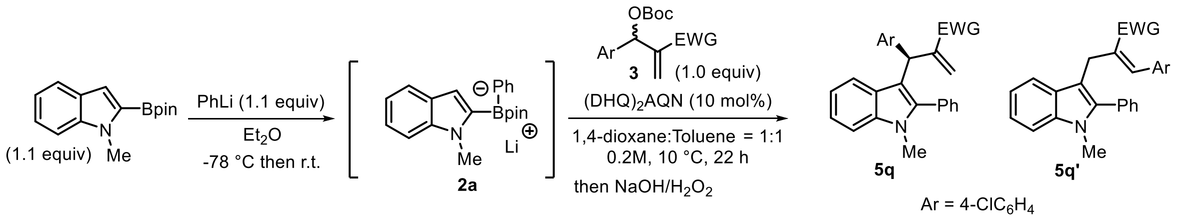

Phenyl lithium (in dibutyl ether, 3.3 mmol, 1.10 equiv) was added dropwise to a 20 mL Schlenk tube containing a solution of indole boronic ester (0.85g, 3.3 mmol, 1.10 equiv) in anhydrous Et_2O (15 mL) at -78 °C (dry ice acetone bath). Subsequently, the reaction was removed from the cooling bath and warmed to ambient temperature. After stirring for 30 min, the solvent was removed under high vacuum and refilled with N_2. Next, MBH carbonate **3** (1.09 mg, 3.0 mmol, 1.0 equiv) and $(DHQ)_2AQN$ (258 mg, 0.3 mmol, 10 mol%) were added quickly under a positive pressure of nitrogen. The reaction flask was sealed, vacuum and refilled with N_2 3 times, 15 mL mixture of 1,4-dioxane and toluene (1/1 v/v) was then added. The reaction was stirred at

10 °C for 22 h. After cooling to 0 °C, the resulting solution was diluted with THF (15 mL). The reaction mixture was treated with NaOH (3 M aqueous solution, 15 mL) followed by the dropwise addition of H_2O_2 (30% aqueous solution, 6 mL) at 0 °C. The reaction mixture was allowed to stir at room temperature for 3 h, and then quenched with saturated aqueous $Na_2S_2O_3$ (6 mL) at 0 °C. After stirring for 10 min at ambient temperature, the reaction mixture was extracted with Et_2O (3 x 50 mL). The combined organic layers were dried using Na_2SO_4, filtered, and concentrated in vacuo. The residue was purified by flash column chromatography (Hex:Et_2O:TEA = 300:15:10) on silica gel to afford the desired product.

2.3.6. Procedure F: kinetic and stereochemical reaction profiles.

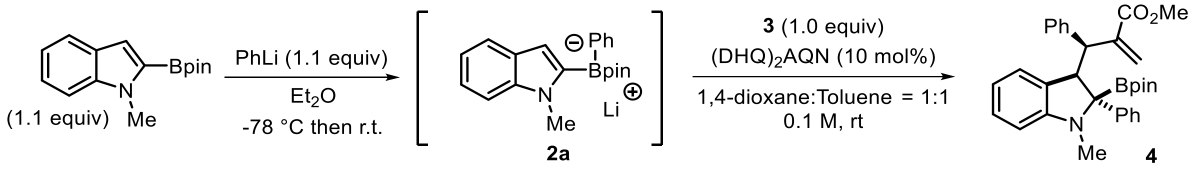

Phenyl lithium (in dibutyl ether, 0.88 mmol, 1.10 equiv) was added dropwise to a 20 mL Schlenk tube containing a solution of indole boronic ester (226.4 mg, 0.88 mmol, 1.10 equiv) in anhydrous Et_2O (5 mL) at -78 °C (dry ice acetone bath). Subsequently, the reaction was removed from the cooling bath and warmed to ambient temperature. After stirring for 30 min, the solvent was removed under high vacuum and refilled with N_2. Next, MBH carbonate **3** (234 mg, 0.8 mmol, 1.0 equiv) and $(DHQ)_2AQN$ (68.8 mg, 0.08 mmol, 10 mol%) was added. The reaction flask was sealed, evacuated and refilled with N_2 3 times. An 8 mL mixture of 1,4-dioxane and toluene (1/1 v/v) and hexadecane (38.8 mg, 50 uL, internal standard) was then added. The reaction was stirred at ambient temperature, and periodically monitored by GC analysis.

GC analysis sample preparation: Using a syringe, a 0.2 mL aliquot was taken at each interval. This was filtered through a silica plug eluting with Et_2O, concentrated in vacuo, and redissolved in ethyl acetate in a GC vial.

2.3.7. Procedure G: deprotection of 5dd.

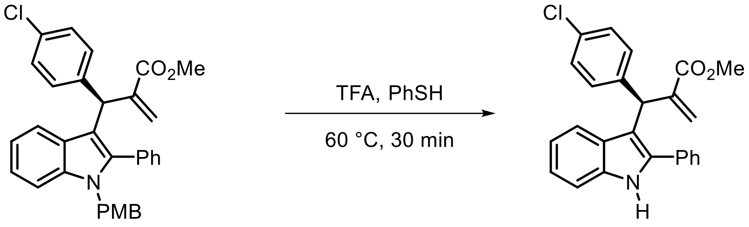

5dd (93.6:6.4 er) → **8** 84%, 93.4:6.6 er

A vial was charged with a mixture of **5dd** (60 mg, 0.115 mmol, 1.00 equiv.), thiophenol (0.130 mL, 1.150 mmol, 10.0 equiv.), and trifluoroacetic acid (1.8 mL) and stirred at 60 °C for 30 min. The solvent was removed in vacuo and the resulting residue dissolved in CH_2Cl_2 (10 mL). The solution was transferred to a separatory funnel and vigorously shaken with 1 N HCl (3 mL). The CH_2Cl_2 was removed, and the aqueous solution was extracted with CH_2Cl_2 (3×10 mL). The combined organic layers were dried using Na_2SO_4, filtered, and concentrated in vacuo. The residue was purified by flash column chromatography (Hex:EtOAc = 5:1) on silica gel to afford the desired product **8** (38.7 mg, 0.097 mmol, 84%).

2.4. Synthesis of starting materials

2.4.1. Synthesis of MBH adducts.

All racemic allylic carbonates were synthesized according by previously reported methods.[2-3]

2.4.2. Synthesis of noncommercially available boronic esters used in this project.

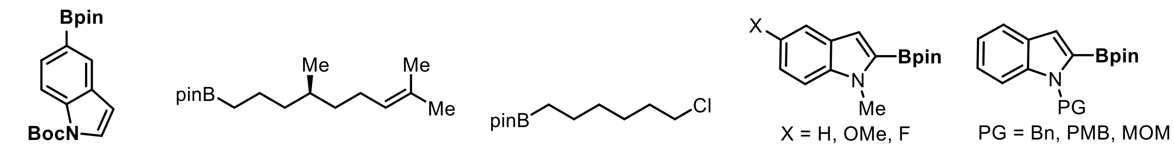

J. Med. Chem. **2012**, *55*, 2437. — *Org. Lett.* **2021**, *23*, 4300–4304 — *Tetrahedron* **2004**, *60*, 10695–10700 — *Chem. Commun.* **2015**, *51*, 6508−6511 — *Chem. Commun.* **2015**, *51*, 6508−6511

2.5. Characterization data for products.

methyl (*R*)-2-((1-methyl-2-phenyl-1H-indol-3-yl)(phenyl)methyl)acrylate (5a)

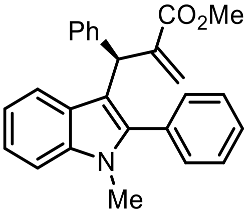

Prepared following **General procedure A**. Purification by flash column chromatography (10% Et_2O in *n*-hexane) gave the title compound (55.4 mg, 73%, 96:4 er, 98:2 rr).

^{1}H NMR (400 MHz, $CDCl_3$) δ 7.50 – 7.39 (m, 4H), 7.38 – 7.27 (m, 3H), 7.26 – 7.08 (m, 6H), 7.01 (ddd, *J* = 8.1, 7.0, 1.1 Hz, 1H), 6.41 (t, *J* = 1.3 Hz, 1H), 5.53 (t, *J* = 1.6 Hz, 1H), 5.40 (s, 1H), 3.59 (s, 3H), 3.58 (s, 3H) ppm. **^{13}C NMR** (126 MHz, $CDCl_3$) δ 167.7, 143.5, 142.8, 139.1, 137.4, 131.9, 130.8, 128.7, 128.5, 128.3, 127.3, 126.1, 121.5, 120.8, 119.4, 112.3, 109.50, 52.0, 44.5, 31.0 ppm. **Specific rotation** $[\alpha]_D^{23}$ = +61 (c = 1.2, CH_2Cl_2). **HRMS** (ESI) m/z calculated for $C_{26}H_{24}NO_2$ $[M+H]^+$, 382.1802, found: 382.1783. **IR (neat)** 3057, 2949, 1719, 1626, 1132, 737, 700 cm^{-1}. **HPLC conditions**: Chiral column IC, hexane: isopropanol = 97:3, flow rate = 1.0 mL/min, wavelength = 254 nm, tR = 7.3 min for major isomer, tR = 6.9 min for minor isomer.

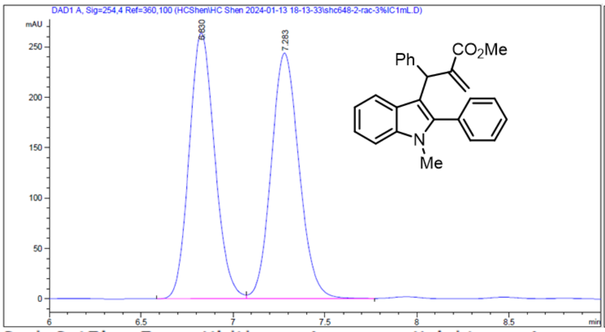

Peak #	RetTime [min]	Type	Width [min]	Area [mAU*s]	Height [mAU]	Area %
1	6.830	BV	0.1505	2545.27710	263.87143	49.8168
2	7.283	VB	0.1647	2563.99951	243.88641	50.1832

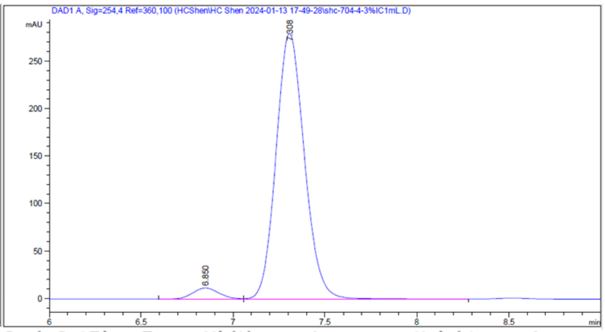

Peak #	RetTime [min]	Type	Width [min]	Area [mAU*s]	Height [mAU]	Area %
1	6.850	BV	0.1527	114.37309	11.62927	3.6743
2	7.308	VB	0.1670	2998.37305	279.75919	96.3257

methyl 2-((*S*)-((2*S*,3*S*)-1-methyl-2-phenyl-2-(4,4,5,5-tetramethyl-1,3,2-dioxaborolan-2-

yl)indolin-3-yl)(phenyl)methyl)acrylate (4)

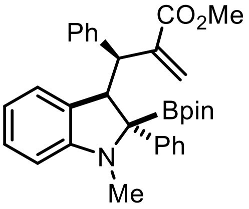

Prepared following **General procedure C**. Purification by flash column chromatography (10% Et_2O in *n*-hexane) gave the title compound (85.2 mg, 84%, >95:5 rr). Average yield over 2 reactions.

^{1}H NMR (400 MHz, CD_2Cl_2) δ 7.25-7.21 (m, 4H), 7.21 – 7.13 (m, 4H), 7.08 – 7.00 (m, 2H), 6.98 (td, *J* = 7.6, 1.3 Hz, 1H), 6.45 (s, 1H), 6.33 (dd, *J* = 7.8, 1.0 Hz, 1H), 6.10 (td, *J* = 7.4, 1.0 Hz, 1H), 5.93 (d, *J* = 1.0 Hz, 1H), 5.43 (dt, *J* = 7.3, 1.2 Hz, 1H), 4.48 (dd, *J* = 11.5, 0.9 Hz, 1H), 3.85 (d, *J* = 11.5 Hz, 1H), 3.54 (s, 3H), 2.79 (s, 3H), 1.29 (s, 6H), 1.25 (s, 6H) ppm. **^{13}C NMR** (126 MHz, CD_2Cl_2) δ 166.8, 152.5, 145.3, 144.0, 142.1, 130.2, 128.6, 128.3, 128.2, 128.2, 128.1, 126.9, 126.6, 126.4, 125.2, 115.6, 104.0, 84.7, 58.9, 51.8, 49.5, 32.5, 26.2, 24.3 ppm. **HRMS** (ESI) m/z calculated for $C_{31}H_{36}BN_2O_4$ $[M+H]^+$, 511.2738, found: 511.2763. **IR (neat)** 2980, 1720, 1626,1599,1488, 1141, 852, 745, 702 cm^{-1}.

methyl (*Z*)-2-((1-methyl-2-phenyl-1H-indol-3-yl)methyl)-3-phenylacrylate (5a')

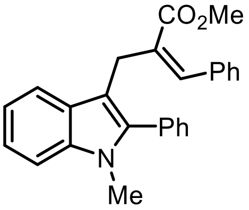

Prepared following **General procedure A**. THF:toluene = 1:1 as solvent, without catalyst. Purification by flash column chromatography (10% Et_2O in *n*-hexane) gave the title compound (34.9 mg, 33%).

^{1}H NMR (400 MHz, $CDCl_3$) δ 7.68 (s, 1H), 7.58-7.56 (m, 1H), 7.49 – 7.35 (m, 7H), 7.38 – 7.25 (m, 4H), 7.24-7.22 (m, 1H), 7.09-7.04 (m, 1H), 4.07 (d, *J* = 1.1 Hz, 2H), 3.61 (s, 3H), 3.54 (s, 3H) ppm. **^{13}C NMR** (126 MHz, $CDCl_3$) δ 168.9, 139.2, 138.4, 137.1, 135.7, 132.3, 132.0,

131.0, 129.8, 128.5, 128.4, 128.3, 128.2, 127.6, 121.5, 119.7, 119.3, 109.5, 109.3, 51.9, 30.9, 24.4 ppm. **HRMS** (ESI) m/z calculated for $C_{26}H_{24}NO_2$ $[M+H]^+$, 382.1802, found: 382.1784. **IR (neat)** 3054, 2947, 1712, 1681, 1467, 1230, 1090, 926, 741, 701 cm^{-1}.

methyl (*R*)-2-((2-(4-methoxyphenyl)-1-methyl-1H-indol-3-yl)(phenyl)methyl)acrylate (5b)

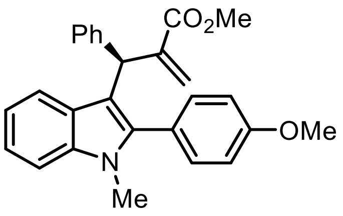

Prepared following **General procedure A**. Purification by flash column chromatography (10% Et_2O in *n*-hexane) gave the title compound (61.3 mg, 75%, 94:6 er, 95:5 rr).

^{1}H NMR (500 MHz, $CDCl_3$) δ 7.47 (d, *J* = 8.1, 1H), 7.33 (d, *J* = 8.3, 1H), 7.27 – 7.18 (m, 7H), 7.17-7.13 (m, 1H), 7.06 – 6.96 (m, 3H), 6.43 (t, *J* = 1.3 Hz, 1H), 5.55 (t, *J* = 1.6 Hz, 1H), 5.42 (s, 1H), 3.88 (s, 3H), 3.61 (s, 3H), 3.58 (s, 3H) ppm. **^{13}C NMR** (126 MHz, $CDCl_3$) δ 167.7, 159.7, 143.6, 142.9, 139.0, 137.3, 132.0, 128.7, 128.3, 127.3, 127.1, 126.1, 124.1, 121.3, 120.7, 119.3, 113.9, 112.1, 109.4, 55.4, 51.9, 44.6, 30.9 ppm. **Specific rotation** $[\alpha]_D^{23}$ = +53 (c = 1.2, CH_2Cl_2). **HRMS** (ESI) m/z calculated for $C_{27}H_{26}NO_3$ $[M+H]^+$, 412.1907, found: 412.1888. **IR (neat)** 2950, 1720, 1611, 1504, 1466, 1249, 1135, 838, 745, 703 cm^{-1}. **HPLC conditions**: Chiral column IA, hexane: isopropanol = 99:1, flow rate = 0.5 mL/min, wavelength = 254 nm, tR = 20.8 min for major isomer, tR = 19.6 min for minor isomer.

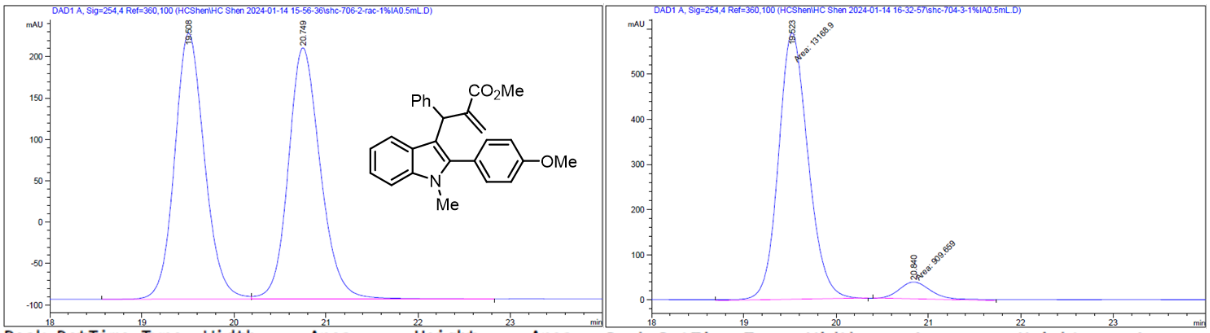

Peak #	RetTime [min]	Type	Width [min]	Area [mAU*s]	Height [mAU]	Area %
1	19.508	BV	0.3465	7235.28320	321.57480	49.6341
2	20.749	VB	0.3721	7341.97266	303.67636	50.3659

Peak #	RetTime [min]	Type	Width [min]	Area [mAU*s]	Height [mAU]	Area %
1	19.523	MM	0.3720	1.31689e4	590.03680	93.5387
2	20.840	MM	0.4055	909.65857	37.39238	6.4613

methyl (*R*)-2-((2-(4-bromophenyl)-1-methyl-1H-indol-3-yl)(phenyl)methyl)acrylate (5c)

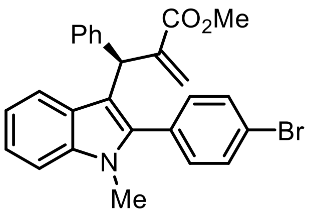

Prepared following **General procedure A**. Purification by flash column chromatography (10% Et_2O in *n*-hexane) gave the title compound (64.5 mg, 70%, 96:4 er, 93:7 rr).

^{1}H NMR (500 MHz, $CDCl_3$) δ 7.59 – 7.58 (m, 2H), 7.50 (d, *J* = 8.1, 1H), 7.37 – 7.31 (m, 1H), 7.24 – 7.14 (m, 8H), 7.06-7.03 (m, 1H), 6.43 (t, *J* = 1.3 Hz, 1H), 5.52 (t, *J* = 1.5 Hz, 1H), 5.38 (s, 1H), 3.60 (s, 3H), 3.57 (s, 3H) ppm. **^{13}C NMR** (126 MHz, $CDCl_3$) δ 167.6, 143.4, 142.5, 137.8, 137.5, 132.4, 131.7, 130.9, 128.8, 128.3, 127.2, 127.2, 126.3, 122.9, 121.8, 120.9, 119.6, 112.6, 109.6, 52.0, 44.6, 31.0 ppm. **Specific rotation** $[\alpha]_D^{23}$ = +39 (c = 1.2, CH_2Cl_2). **HRMS** (ESI) m/z calculated for $C_{26}H_{23}BrNO_2$ $[M+H]^+$, 460.0907, found: 460.0917. **IR (neat)** 2949, 1721, 1626, 1465, 1246, 1134, 1011, 832, 741, 702 cm^{-1}. **HPLC conditions**: Chiral column IA, hexane: isopropanol = 99:1, flow rate = 0.5 mL/min, wavelength = 254 nm, tR = 13.1 min for major isomer, tR = 13.7 min for minor isomer.

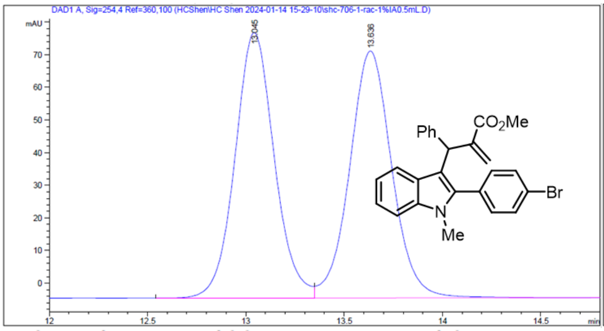

Peak #	RetTime [min]	Type	Width [min]	Area [mAU*s]	Height [mAU]	Area %
1	13.045	BV	0.2117	1128.21265	81.40012	49.9048
2	13.636	VB	0.2285	1132.51904	75.72210	50.0952

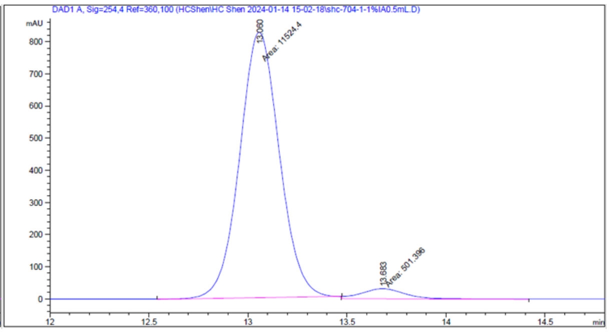

Peak #	RetTime [min]	Type	Width [min]	Area [mAU*s]	Height [mAU]	Area %
1	13.060	MM	0.2325	1.15244e4	826.24200	95.8306
2	13.683	MM	0.2674	501.39648	31.24792	4.1694

**Methyl (*R*)-2-((1-methyl-2-(4-(trifluoromethyl)phenyl)-1H-indol-3-yl)(phenyl)methyl)

acrylate (5d)

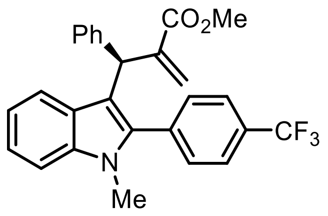

Prepared following **General procedure A**. Purification by flash column chromatography (10% Et_2O in *n*-hexane) gave the title compound (64.1 mg, 64%, 97:3 er, 90:10 rr).

^{1}H NMR (400 MHz, $CDCl_3$) δ 7.69 (d, *J* = 8.0 Hz, 1H), 7.51 (d, *J* = 8.0 Hz, 1H), 7.42 (d, *J* = 7.8 Hz, 1H), 7.35 (d, *J* = 8.2 Hz, 1H), 7.27 – 7.10 (m, 6H), 7.07-7.03 (m, 1H), 6.42 (s, 1H), 5.51 (s, 1H), 5.38 (s, 1H), 3.59 (s, 3H), 3.57 (s, 3H) ppm. **^{13}C NMR** (126 MHz, $CDCl_3$) δ 167.7, 143.4, 142.3, 137.7, 137.5, 135.8, 131.2, 130.4 (q, *J* = 32.4 Hz), 128.8, 128.4, 127.3, 127.2, 126.4, 125.4 (q, *J* = 3.8 Hz), 122.1, 121.0, 119.8, 113.0, 109.7, 52.0, 44.5, 31.1 ppm. **Specific rotation** $[\alpha]_D^{23}$ = +77 (c = 0.8, CH_2Cl_2). **HRMS** (ESI) m/z calculated for $C_{27}H_{23}F_3NO_2$ $[M+H]^+$, 450.1675, found: 450.1653. **IR (neat)** 2922, 1720, 1618, 1466, 1321, 1124, 849, 739, 702 cm^{-1}. **HPLC conditions**: Chiral column IA, hexane: isopropanol = 99:1, flow rate = 0.5 mL/min, wavelength = 254 nm, tR = 11.0 min for major isomer, tR = 11.6 min for minor isomer.

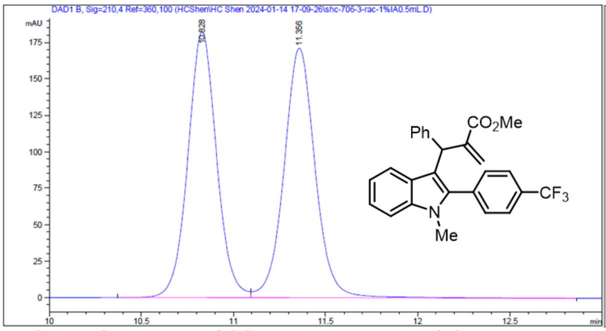

Peak #	RetTime [min]	Type	Width [min]	Area [mAU*s]	Height [mAU]	Area %
1	10.828	BV	0.1663	1973.69568	182.33331	49.4226
2	11.356	VB	0.1795	2019.80896	171.40485	50.5774

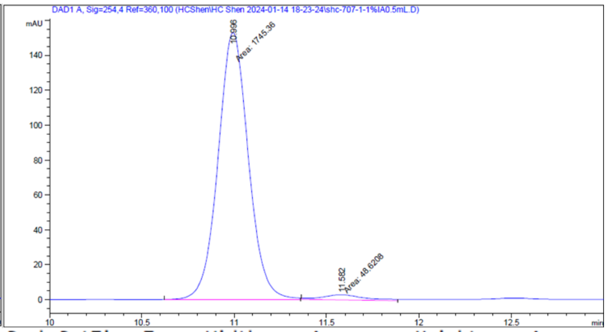

Peak #	RetTime [min]	Type	Width [min]	Area [mAU*s]	Height [mAU]	Area %
1	10.996	MM	0.1901	1745.36035	153.02264	97.2898
2	11.582	MM	0.2689	48.62083	3.01333	2.7102

methyl (*R*)-2-((2-(4-cyanophenyl)-1-methyl-1H-indol-3-yl)(phenyl)methyl)acrylate (5e)

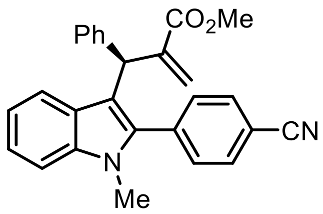

Prepared following **General procedure A**. Purification by flash column chromatography (10% Et_2O in *n*-hexane) gave the title compound (26.0 mg, 32%, 82:18 er, 98:2 rr).

^{1}H NMR (500 MHz, $CDCl_3$) δ 7.72 (d, J = 8.6 Hz, 2H), 7.52 (d, J = 8.1 Hz, 1H), 7.42 (d, J = 7.8 Hz, 2H), 7.38 – 7.32 (m, 1H), 7.29 – 7.23 (m, 1H), 7.26 – 7.11 (m, 5H), 7.08-7.04 (m, 1H), 6.42 (t, J = 1.3 Hz, 1H), 5.50 (t, J = 1.5 Hz, 1H), 5.37 (s, 1H), 3.59 (s, 3H), 3.58 (s, 3H). ppm. **^{13}C NMR** (126 MHz, $CDCl_3$) 167.5, 143.3, 142.1, 137.9, 136.9, 136.9, 132.2, 131.5, 128.8, 128.4, 127.4, 127.2, 126.5, 122.4, 121.1, 119.9, 118.8, 113.4, 112.1, 109.7, 52.1, 44.5, 31.2 ppm. **Specific rotation** $[\alpha]_D^{23}$ = +28 (c = 1.0, CH_2Cl_2). **HRMS** (ESI) m/z calculated for $C_{27}H_{23}N_2O_2$ $[M+H]^+$, 407.1754, found: 407.1756. **IR (neat)** 2970, 2227, 1720, 1609, 1455, 1365, 1217, 848, 747, 704 cm^{-1}. **HPLC conditions**: Chiral column IA, hexane: isopropanol = 98:2, flow rate = 1.0 mL/min, wavelength = 230 nm, tR = 19.2 min for major isomer, tR = 18.1 min for minor isomer.

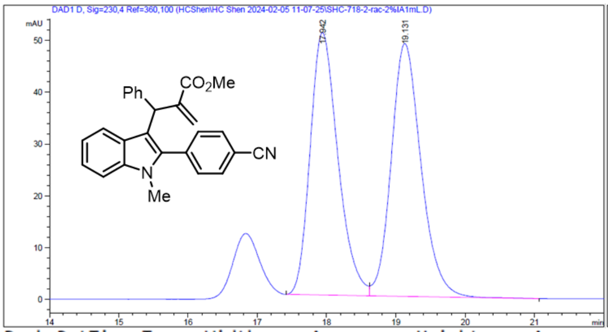

Peak #	RetTime [min]	Type	Width [min]	Area [mAU*s]	Height [mAU]	Area %
1	17.942	BV	0.4271	1412.07227	50.67032	49.1492
2	19.131	VB	0.4552	1460.96240	48.81120	50.8508

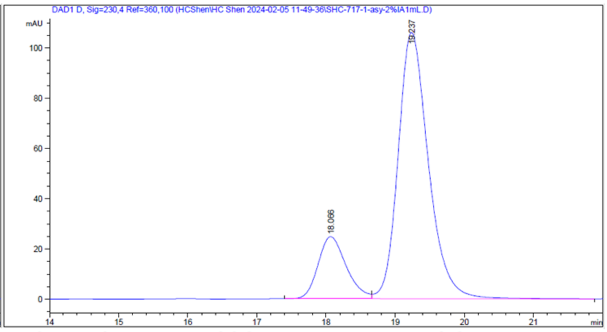

Peak #	RetTime [min]	Type	Width [min]	Area [mAU*s]	Height [mAU]	Area %
1	18.066	BV	0.4338	698.49335	24.70675	17.8337
2	19.237	VB	0.4626	3218.20898	105.89412	82.1663

methyl (*R*)-2-((1-methyl-2-(o-tolyl)-1H-indol-3-yl)(phenyl)methyl)acrylatehenyl (5f)

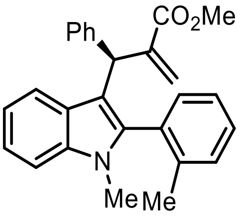

Prepared following **General procedure A**. Purification by flash column chromatography (10% Et_2O in *n*-hexane) gave the title compound (49.7 mg, 63%, 96:4 er, 97:3 rr).

^{1}H NMR (500 MHz, $CDCl_3$) δ 7.53 (d, *J* = 7.9 Hz, 1H), 7.34 (d, *J* = 7.8 Hz, 2H), 7.32 – 7.26 (m, 1H), 7.25 – 7.13 (m, 6H), 7.13-7.10 (m, 1H), 7.08 – 7.01 (m, 2H), 6.38 (t, *J* = 1.4 Hz, 1H), 5.55 (t, *J* = 1.6 Hz, 1H), 5.24 (s, 1H), 3.58 (s, 3H), 3.43 (s, 3H), 2.07 (s, 3H) ppm. **^{13}C NMR** (126 MHz, $CDCl_3$) δ 167.8, 143.2, 142.9, 139.1, 138.8, 137.2, 131.6, 131.4, 130.1, 129.1, 128.7, 128.2, 127.3, 127.1, 126.0, 125.7, 121.2, 120.7, 119.3, 112.2, 109.4, 51.9, 44.7, 30.3, 19.9 ppm. **Specific rotation** $[\alpha]_D^{23}$ = +136 (c = 1.3, CH_2Cl_2). **HRMS** (ESI) m/z calculated for $C_{27}H_{26}NO_2$ $[M+H]^+$, 396.1958, found: 396.1942. **IR (neat)** 2948, 1723, 1625, 1465, 1365, 1281, 1134, 946, 743, 701cm^{-1}. **HPLC conditions**: Chiral column IC, hexane: isopropanol = 99:1, flow rate = 1.0 mL/min, wavelength = 210 nm, tR = 8.7 min for major isomer, tR = 7.3 min for minor isomer.

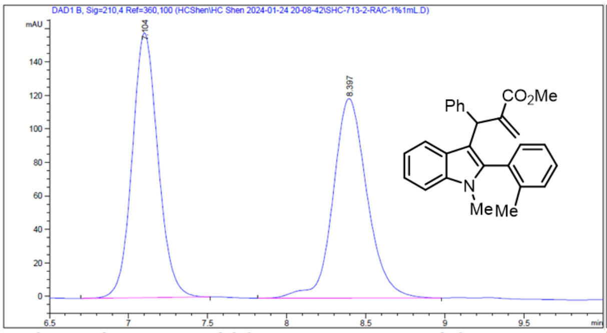

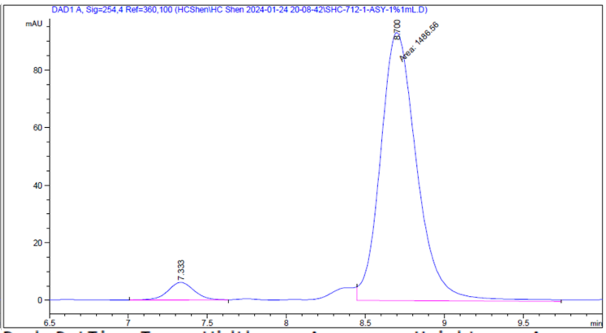

Peak #	RetTime [min]	Type	Width [min]	Area [mAU*s]	Height [mAU]	Area %
1	7.104	BB	0.1744	1796.12805	158.22261	50.1478
2	8.397	BB	0.2288	1785.54150	119.18542	49.8522

Peak #	RetTime [min]	Type	Width [min]	Area [mAU*s]	Height [mAU]	Area %
1	7.333	BB	0.1749	69.26404	6.07880	4.4519
2	8.700	MM	0.2663	1486.56165	93.04391	95.5481

methyl (R)-2-((2-(3-chlorophenyl)-1-methyl-1H-indol-3-yl)(phenyl)methyl)acrylate (5g)

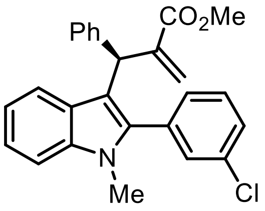

Prepared following **General procedure A**. Purification by flash column chromatography (10% Et_2O in *n*-hexane) gave the title compound (63.5 mg, 77%, 93:7 er, 92:8 rr).

^{1}H NMR (500 MHz, $CDCl_3$) δ 7.51 (d, J = 8.1 Hz, 1H), 7.45 – 7.32 (m, 3H), 7.30 (s, 1H), 7.28 – 7.13 (m, 7H), 7.07-7.04 (m, 1H), 6.44 (t, J = 1.3 Hz, 1H), 5.53 (t, J = 1.6 Hz, 1H), 5.43 (s, 1H), 3.63 (s, 3H), 3.58 (s, 3H) ppm. **^{13}C NMR** (126 MHz, $CDCl_3$) δ 167.6, 143.4, 142.4, 137.5, 137.4, 134.3, 133.8, 130.8, 129.7, 129.0, 128.7, 128.6, 128.3, 127.3, 127.2, 126.3, 121.9, 120.9, 119.7, 113.0, 109.6, 52.0, 44.5, 31.0 ppm. **Specific rotation** $[\alpha]_D^{23}$ = +36 (c = 1.3, CH_2Cl_2). **HRMS** (ESI) m/z calculated for $C_{26}H_{23}ClNO_2$ $[M+H]^+$, 416.1412 found: 416.1394. **IR (neat)** 2948, 1720, 1599, 1465, 1364, 1245, 1134, 998, 737, 701 cm^{-1}. **HPLC conditions**: Chiral column IC, hexane: isopropanol = 99:1, flow rate = 1.0 mL/min, wavelength = 254 nm, tR = 9.4 min for major isomer, tR = 8.3 min for minor isomer.

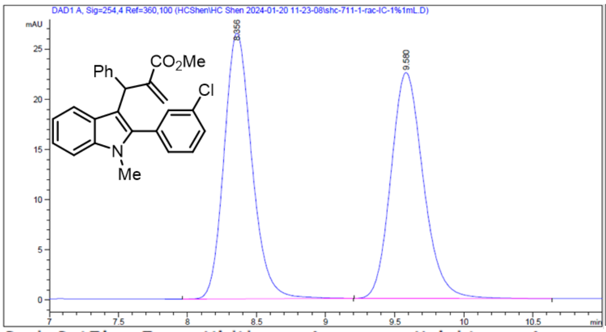

Peak #	RetTime [min]	Type	Width [min]	Area [mAU*s]	Height [mAU]	Area %
1	8.356	BB	0.2148	369.27884	26.46113	50.1802
2	9.580	BB	0.2521	366.62674	22.50101	49.8198

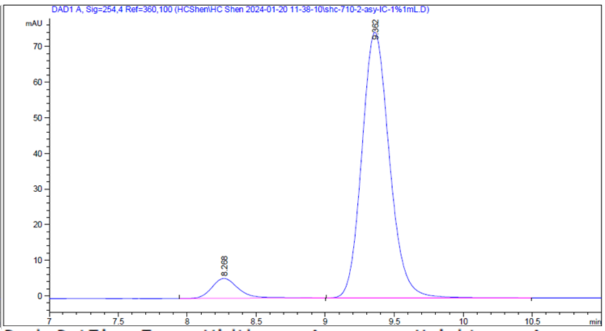

Peak #	RetTime [min]	Type	Width [min]	Area [mAU*s]	Height [mAU]	Area %
1	8.268	BB	0.2060	75.61131	5.58380	6.8087
2	9.362	BB	0.2139	1034.89478	74.58764	93.1913

methyl (*R*)-2-((1-methyl-2-(naphthalen-2-yl)-1H-indol-3-yl)(phenyl)methyl)acrylate (5h)

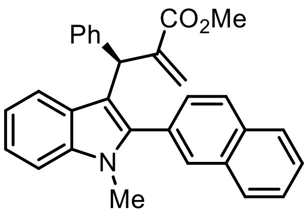

Prepared following **General procedure A**. Purification by flash column chromatography (10% Et_2O in *n*-hexane) gave the title compound (56.2 mg, 65%, 95:5 er, 96:4 rr).

^{1}H NMR (500 MHz, $CDCl_3$) δ 7.96 – 7.93 (m, 2H), 7.88 – 7.78 (m, 2H), 7.60 – 7.54 (m, 3H), 7.48 (d, J = 8.3 Hz, 1H), 7.39 (d, J = 8.2 Hz, 1H), 7.30 – 7.20 (m, 5H), 7.19 – 7.15 (m, 1H), 7.10 – 7.07 (m, 1H), 6.47 (s, 1H), 5.60 (s, 1H), 5.51 (s, 1H), 3.65 (s, 3H), 3.59 (s, 3H) ppm. **^{13}C NMR** (126 MHz, $CDCl_3$) δ 167.7, 143.6, 142.8, 139.0, 137.6, 133.2, 133.1, 130.24, 129.3, 128.8, 128.3, 128.3, 128.2, 128.0, 127.9, 127.4, 127.2, 126.7, 126.5, 126.2, 121.6, 120.8, 119.5, 112.7, 109.5, 51.9, 44.7, 31.1 ppm. **Specific rotation** $[\alpha]_D^{23}$ = +76 (c = 1.4, CH_2Cl_2) **HRMS** (ESI) m/z calculated for $C_{30}H_{26}NO_2$ $[M+H]^+$, 432.1958, found: 432.1937. **IR (neat)** 3025, 2948, 1721, 1627, 1465, 1366, 1244, 1133, 740, 702 cm^{-1}. **HPLC conditions**: Chiral column IC, hexane: isopropanol = 99:1, flow rate = 1.0 mL/min, wavelength = 254 nm, tR = 13.3 min for major isomer, tR = 11.0 min for minor isomer.

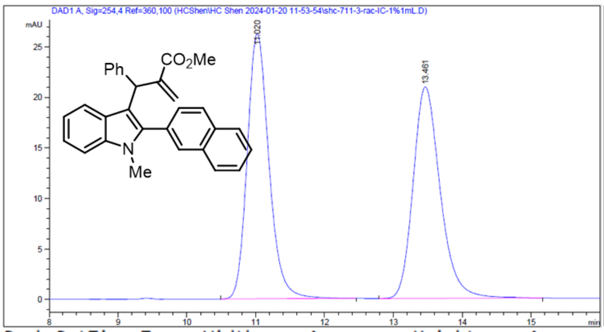

Peak #	RetTime [min]	Type	Width [min]	Area [mAU*s]	Height [mAU]	Area %
1	11.020	BB	0.3376	575.11969	26.25148	50.0570
2	13.461	BB	0.4197	573.80988	20.93847	49.9430

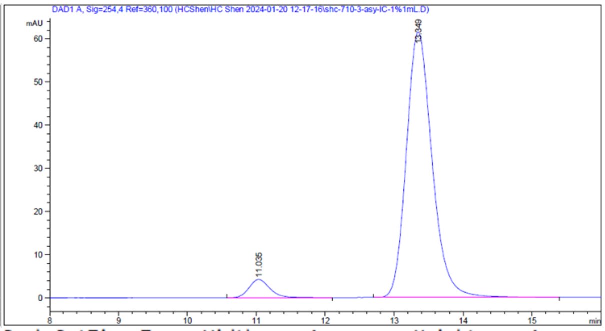

Peak #	RetTime [min]	Type	Width [min]	Area [mAU*s]	Height [mAU]	Area %
1	11.035	BB	0.3219	88.22632	4.22003	5.2305
2	13.349	BB	0.4015	1598.54297	61.45463	94.7695

methyl (*R*)-2-((1-methyl-2-(thiophen-2-yl)-1H-indol-3-yl)(phenyl)methyl)acrylate (5i)

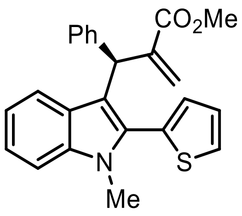

Prepared following **General procedure A**. Purification by flash column chromatography (25% Et_2O in *n*-hexane) gave the title compound (51.4 mg, 66%, 95:5 er, 94:6 rr).

^{1}H NMR (500 MHz, $CDCl_3$) δ 7.49 (dd, *J* = 5.2, 1.2 Hz, 1H), 7.45 (d, *J* = 8.1 Hz, 1H), 7.33 (d, *J* = 8.3 Hz, 1H), 7.27 – 7.11 (m, 7H), 7.07 – 6.97 (m, 2H), 6.44 (s, 1H), 5.57 (s, 1H), 5.52 (s, 1H), 3.66 (s, 3H), 3.64 (s, 3H) ppm. **^{13}C NMR** (126 MHz, $CDCl_3$) δ 167.7, 143.3, 142.6, 137.6, 132.0, 131.2, 130.0, 128.7, 128.3, 127.9, 127.4, 127.3, 127.0, 126.2, 122.1, 120.9, 119.6, 115.1, 109.6, 52.0, 44.7, 30.9 ppm. **Specific rotation** $[\alpha]_D^{23}$ = +58 (c = 1.2, CH_2Cl_2). **HRMS** (ESI) m/z calculated for $C_{24}H_{22}NO_2S$ $[M+H]^+$, 388.1366, found: 388.1349. **IR (neat)** 2970, 2948, 1721, 1627, 1455, 1366, 1244, 1133, 740, 702 cm^{-1}. **HPLC conditions**: Chiral column IC, hexane: isopropanol = 99:1, flow rate = 1.0 mL/min, wavelength = 254 nm, tR = 11.4 min for major isomer, tR = 10.2 min for minor isomer.

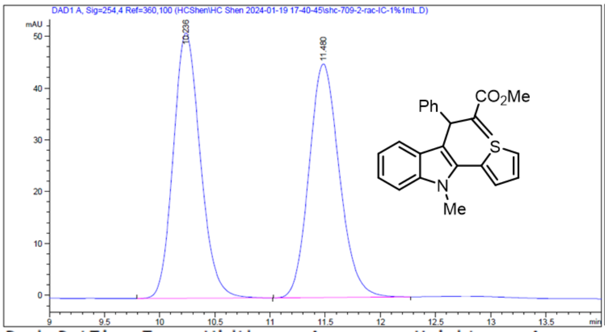

Peak #	RetTime [min]	Type	Width [min]	Area [mAU*s]	Height [mAU]	Area %
1	10.236	BB	0.2626	872.73669	51.25660	50.2386
2	11.480	BB	0.2955	864.44672	45.12495	49.7614

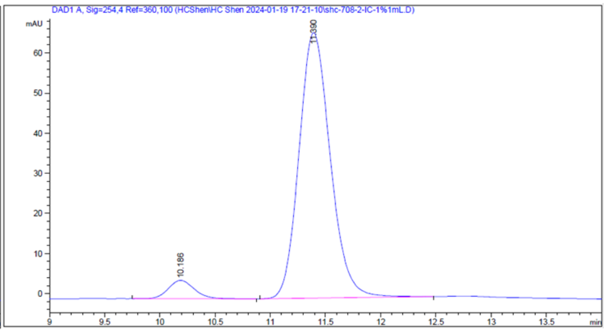

Peak #	RetTime [min]	Type	Width [min]	Area [mAU*s]	Height [mAU]	Area %
1	10.186	BB	0.2525	74.76529	4.57963	5.4327
2	11.390	BB	0.3034	1301.44153	66.17509	94.5673

**tert-butyl (*R*)-3-(2-(methoxycarbonyl)-1-phenylallyl)-1-methyl-1H,1'H-[2,5'-biindole]-1'-

carboxylate (5j)

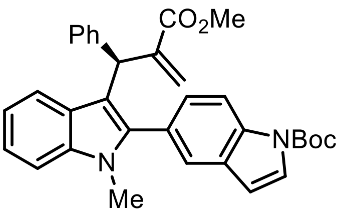

Prepared following **General procedure A**. Purification by flash column chromatography (10% Et_2O in *n*-hexane) gave the title compound (73.0 mg, 70%, 96:4 er, 95:5 rr).

^{1}H NMR (500 MHz, $CDCl_3$) 8.25 (d, *J* = 8.4 Hz, 1H), 7.70 (d, *J* = 3.7 Hz, 1H), 7.55 – 7.45 (m, 2H), 7.35 (d, *J* = 8.0 Hz, 1H), 7.34 – 7.25 (m, 1H), 7.25 – 7.19 (m, 5H), 7.18-7.13 (m, 1H), 7.04 (ddd, *J* = 8.1, 7.0, 1.0 Hz, 1H), 6.62 (d, *J* = 3.6 Hz, 1H), 6.43 (s, 1H), 5.57 (s, 1H), 5.44 (s, 1H), 3.59 (s, 3H), 3.59 (s, 3H), 1.73 (s, 9H) ppm. **^{13}C NMR** (126 MHz, $CDCl_3$) δ 167.8, 149.8, 143.6, 142.9, 139.6, 137.4, 130.8, 128.8, 128.2, 127.3, 127.2, 126.9, 126.7, 126.2, 126.1, 123.4, 121.3, 120.7, 119.3, 115.1, 112.3, 109.4, 107.5, 84.1, 51.9, 44.7, 31.0, 28.3 ppm. **Specific rotation** $[\alpha]_D^{23}$ = +66 (c = 1.6, CH_2Cl_2). **HRMS** (ESI) m/z calculated for $C_{33}H_{33}N_2O_4$ $[M+H]^+$, 521.2435, found: 521.2410. **IR (neat)** 2971, 1736, 1627, 1462, 1365, 1246, 1134, 826, 735, 701 cm^{-1}. **HPLC conditions**: Chiral column IC, hexane: isopropanol = 95:5, flow rate = 1.0 mL/min, wavelength = 210 nm, tR = 13.9 min for major isomer, tR = 8.8 min for minor isomer.

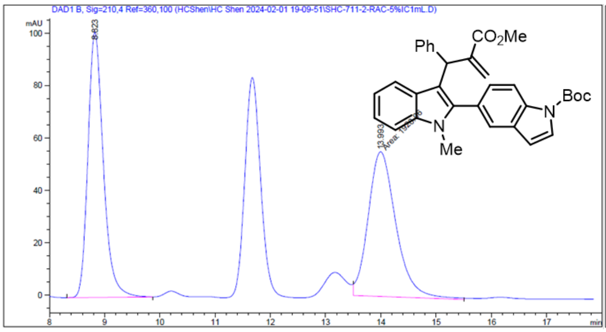

Peak #	RetTime [min]	Type	Width [min]	Area [mAU*s]	Height [mAU]	Area %
1	8.823	BB	0.2977	1958.12903	101.21107	50.4908
2	13.993	MM	0.5799	1920.06348	55.18218	49.5092

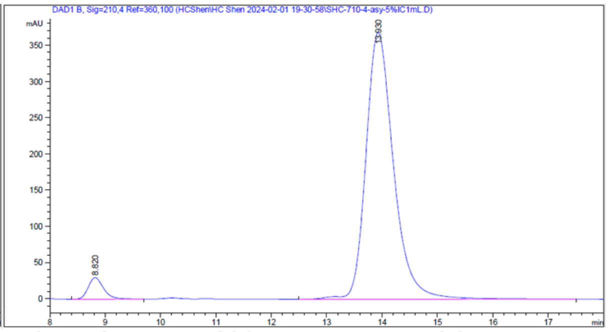

Peak #	RetTime [min]	Type	Width [min]	Area [mAU*s]	Height [mAU]	Area %
1	8.820	BB	0.2934	568.80707	29.70973	4.2676
2	13.930	BB	0.5284	1.27598e4	368.11304	95.7324

tert-butyl (*R*)-4-(3-(2-(methoxycarbonyl)-1-phenylallyl)-1-methyl-1H-indol-2-yl)-3,6-

dihydropyridine-1(2H)-carboxylate (5k)

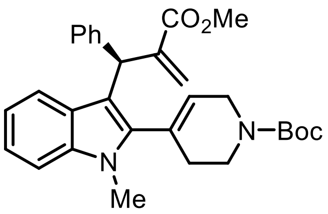

Prepared following **General procedure A**. Purification by flash column chromatography (10% Et_2O in *n*-hexane) gave the title compound (64.0 mg, 66%, 96:4 er, 89:11 rr).

^{1}H NMR (500 MHz, $CDCl_3$) δ 7.42 (d, *J* = 8.0 Hz, 1H), 7.29 – 7.22 (m, 1H), 7.25 – 7.17 (m, 4H), 7.18 – 7.14 (m, 2H), 6.99 (ddd, *J* = 8.0, 7.0, 1.0 Hz, 1H), 6.39 (s, 1H), 5.72 – 5.67 (m, 1H), 5.56 (s, 1H), 5.45 (s, 1H), 4.02 (s, 2H), 3.65 (s, 3H), 3.60 (s, 3H), 3.58 – 3.48 (m, 2H), 2.20 (s, 2H), 1.51 (s, 9H) ppm. **^{13}C NMR** (126 MHz, $CDCl_3$) δ 167.8, 155.1, 143.7, 142.7, 139.6, 137.0, 128.9, 128.3, 127.3, 127.1, 126.3, 121.4, 120.4, 119.3, 111.3, 109.2, 80.0, 52.1, 44.5, 30.6, 30.0, 28.6 ppm. **Specific rotation** $[\alpha]_D^{23}$ = +33 (c = 1.1, CH_2Cl_2). **HRMS** (ESI) m/z calculated for $C_{30}H_{35}N_2O_4$ $[M+H]^+$, 487.2591, found: 487.2573. **IR (neat)** 2970, 1723, 1629, 1365, 1217, 1167, 957, 744, 528 cm^{-1}. **HPLC conditions**: Chiral column IA, hexane: isopropanol = 97:3, flow rate = 1.0 mL/min, wavelength = 210 nm, tR = 12.2 min for major isomer, tR = 14.0 min for minor isomer.

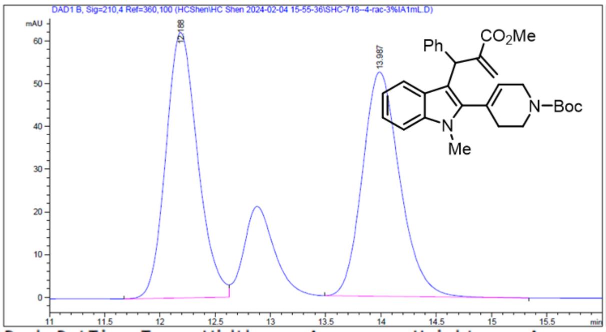

Peak #	RetTime [min]	Type	Width [min]	Area [mAU*s]	Height [mAU]	Area %
1	12.188	BV	0.3070	1239.15344	62.05150	50.1398
2	13.987	BB	0.3603	1232.24170	52.41013	49.8602

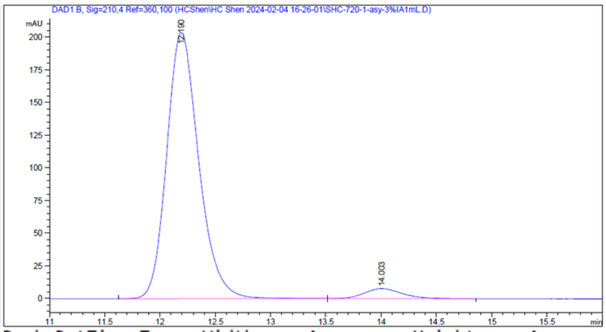

Peak #	RetTime [min]	Type	Width [min]	Area [mAU*s]	Height [mAU]	Area %
1	12.190	BB	0.3064	4089.36621	203.57639	95.7738
2	14.003	BB	0.3647	180.44965	7.60892	4.2262

methyl (*R*)-2-((1-methyl-2-vinyl-1H-indol-3-yl)(phenyl)methyl)acrylate (5l)

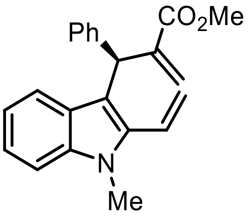

Prepared following **General procedure A**. Purification by flash column chromatography (10% Et_2O in *n*-hexane) gave the title compound (45.4 mg, 69%, 91:9 er, 98:2 rr).

^{1}H NMR (500 MHz, $CDCl_3$) δ 7.35 (d, *J* = 8.1 Hz, 1H), 7.30 – 7.21 (m, 5H), 7.22 – 7.12 (m, 2H), 6.94 (ddd, *J* = 8.0, 6.9, 1.0 Hz, 1H), 6.80 (dd, *J* = 17.8, 11.7 Hz, 1H), 6.43 (t, *J* = 1.3 Hz, 1H), 5.75 (s, 1H), 5.56 (dd, *J* = 11.7, 1.5 Hz, 1H), 5.51 – 5.42 (m, 2H), 3.74 (s, 3H), 3.66 (s, 3H) ppm. **^{13}C NMR** (126 MHz, $CDCl_3$) δ 167.8, 143.4, 142.6, 137.7, 135.8, 128.9, 128.4, 127.4, 127.3, 126.3, 126.1, 121.8, 120.8, 120.3, 119.3, 112.9, 109.3, 52.1, 44.4, 30.9 ppm. **Specific rotation** $[\alpha]_D^{23}$ = -7 (c = 0.9, CH_2Cl_2). **HRMS** (ESI) m/z calculated for $C_{22}H_{22}NO_2$ $[M+H]^+$, 332.1645, found: 332.1631. **IR (neat)** 2970, 1738, 1610, 1366, 1217, 1206, 749, 528 cm^{-1}. **HPLC conditions**: Chiral column IC, hexane: isopropanol = 99:1, flow rate = 1.0 mL/min, wavelength = 254 nm, tR = 10.7 min for major isomer, tR = 8.9 min for minor isomer.

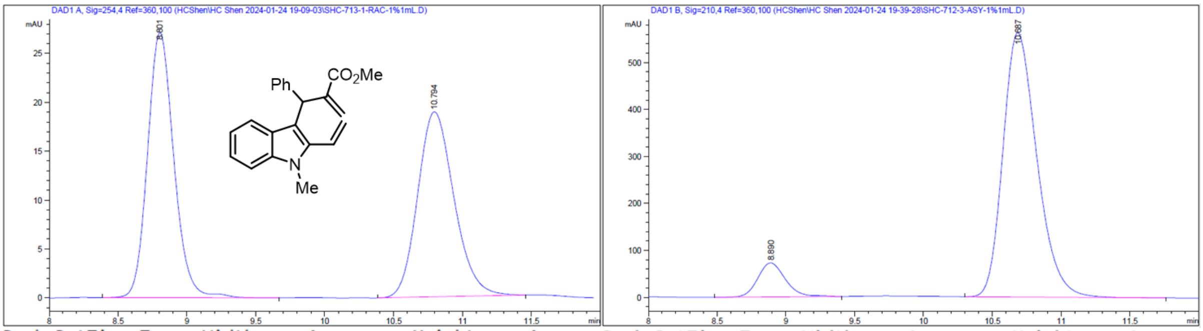

Peak #	RetTime [min]	Type	Width [min]	Area [mAU*s]	Height [mAU]	Area %
1	8.801	BB	0.2029	360.34393	27.14677	50.8849
2	10.794	BB	0.2822	347.81049	18.94602	49.1151

Peak #	RetTime [min]	Type	Width [min]	Area [mAU*s]	Height [mAU]	Area %
1	8.890	BB	0.2153	1016.54242	72.61737	9.5340
2	10.687	BB	0.2661	9645.77051	562.39923	90.4660

methyl (*R*)-2-((2-(6-chlorohexyl)-1-methyl-1H-indol-3-yl)(phenyl)methyl)acrylate (5m)

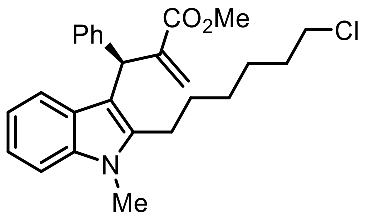

Prepared following **General procedure A**. Purification by flash column chromatography (10% Et_2O in *n*-hexane) gave the title compound (33.8 mg, 40%, 94:6 er, 88:11 rr).

^{1}H NMR (500 MHz, $CDCl_3$) δ 7.36 (d, *J* = 7.8 Hz, 1H), 7.29 – 7.21 (m, 5H), 7.20 – 7.17 (m, 1H), 7.15 – 7.11 (m, 1H), 6.96 (ddd, *J* = 8.0, 7.0, 1.0 Hz, 1H), 6.41 (t, *J* = 1.2 Hz, 1H), 5.63 (s, 1H), 5.49 (t, *J* = 1.5 Hz, 1H), 3.68 (s, 6H), 3.46 (t, *J* = 6.7 Hz, 2H), 2.82 – 2.69 (m, 2H), 1.69 – 1.63 (m, 2H), 1.48 – 1.33 (m, 4H), 1.32 – 1.22 (m, 2H) ppm. **^{13}C NMR** (126 MHz, $CDCl_3$) δ 168.0, 143.5, 142.6, 138.5, 137.0, 128.9, 128.3, 127.5, 127.0, 126.3, 120.5, 119.8, 119.0, 110.4, 108.8, 52.1, 45.1, 44.0, 32.6, 29.8, 29.6, 28.9, 26.7, 24.8 ppm. **Specific rotation** $[\alpha]_D^{23}$ = +24 (c = 0.9, CH_2Cl_2). **HRMS** (ESI) m/z calculated for $C_{26}H_{31}ClNO_2$ $[M+H]^+$, 424.2038, found: 424.2018. **IR (neat)** 2938, 1737, 1627, 1366, 1217, 1134, 742, 703, 527 cm^{-1}. **HPLC conditions**: Chiral column IA, hexane: isopropanol = 99:1, flow rate = 1.0 mL/min, wavelength = 254 nm, tR = 8.1 min for major isomer, tR = 9.0 min for minor isomer.

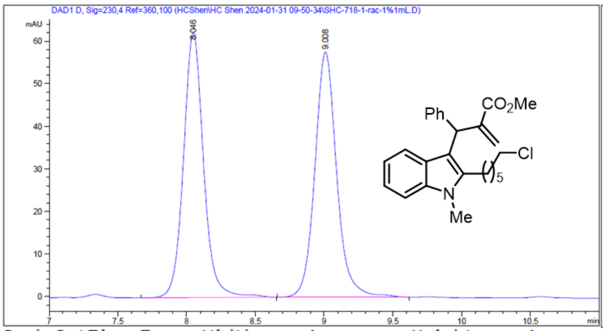

Peak #	RetTime [min]	Type	Width [min]	Area [mAU*s]	Height [mAU]	Area %
1	8.046	BB	0.1546	632.73340	62.26091	49.8222
2	9.008	BB	0.1689	637.24902	57.69070	50.1778

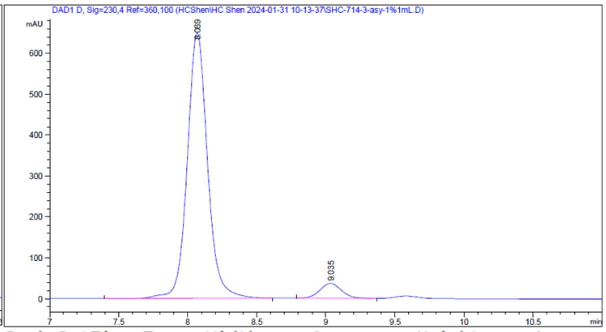

Peak #	RetTime [min]	Type	Width [min]	Area [mAU*s]	Height [mAU]	Area %
1	8.069	BB	0.1550	6629.73682	649.82275	94.4030
2	9.035	BV	0.1685	393.06882	36.26027	5.5970

methyl 2-((*R*)-(2-((*R*)-4,8-dimethylnon-7-en-1-yl)-1-methyl-1H-indol-3-yl)(phenyl)

methyl)acrylate (5n)

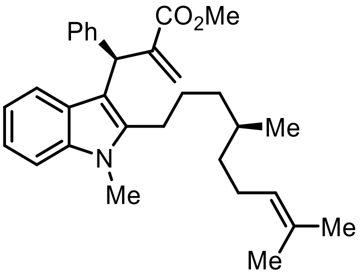

Prepared following **General procedure A**. Purification by flash column chromatography (10% Et_2O in *n*-hexane) gave the title compound (28.9 mg, 32%, 94:6 dr, 94:6 rr).

^{1}H NMR (500 MHz, $CDCl_3$) δ 7.34 (d, *J* = 8.1 Hz, 1H), 7.29 – 7.20 (m, 5H), 7.21 – 7.13 (m, 1H), 7.12 (ddd, *J* = 8.2, 7.0, 1.2 Hz, 1H), 6.95 (ddd, *J* = 8.0, 7.0, 1.0 Hz, 1H), 6.41 (t, *J* = 1.2 Hz, 1H), 5.63 (s, 1H), 5.48 (t, *J* = 1.5 Hz, 1H), 5.09-5.06 (m, 1H), 3.68 (s, 3H), 3.68 (s, 3H), 2.71 (dd, *J* = 8.6, 7.3 Hz, 2H), 2.02 – 1.83 (m, 2H), 1.69 (s, 3H), 1.59 (s, 3H), 1.49 – 1.18 (m, 5H), 1.15 – 1.00 (m, 2H), 0.79 (d, *J* = 6.5 Hz, 3H) ppm. **^{13}C NMR** (126 MHz, $CDCl_3$) δ 168.0, 143.5, 142.7, 138.9, 136.9, 131.2, 128.8, 128.3, 127.6, 127.1, 126.2, 125.0, 120.4, 119.8, 119.0, 110.3, 108.8, 52.1, 44.0, 37.1, 37.1, 32.3, 29.8, 27.3, 25.9, 25.6, 25.2, 19.5, 17.8 ppm. **Specific rotation** $[\alpha]_D^{23}$ = +32 (c = 0.8, CH_2Cl_2). HRMS (ESI) m/z calculated for $C_{31}H_{40}NO_2$ [M+H]+, 458.3054 found: 458.3033. **IR (neat)** 2970, 1738, 1355, 1217, 1183, 901, 747, 528 cm^{-1}. **HPLC conditions**: Chiral column IA, hexane: isopropanol = 99:1, flow rate = 0.5 mL/min, wavelength = 254 nm, tR = 9.8 min for major isomer, tR = 10.4 min for minor isomer.

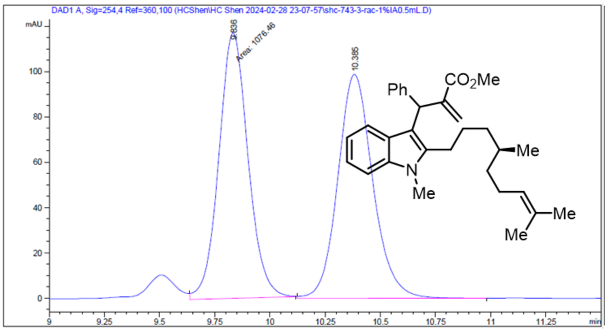

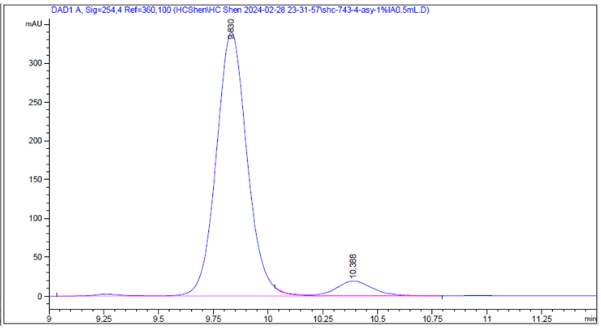

Peak #	RetTime [min]	Type	Width [min]	Area [mAU*s]	Height [mAU]	Area %
1	9.836	MM	0.1531	1076.46350	117.15544	49.7108
2	10.385	VB	0.1685	1088.98926	98.87969	50.2892

Peak #	RetTime [min]	Type	Width [min]	Area [mAU*s]	Height [mAU]	Area %
1	9.830	VV R	0.1497	3333.41870	339.69943	93.7513
2	10.388	VB E	0.1763	222.17856	19.02297	6.2487

methyl (*R*)-2-((4-methoxyphenyl)(1-methyl-2-phenyl-1H-indol-3-yl)methyl)acrylate (5o)

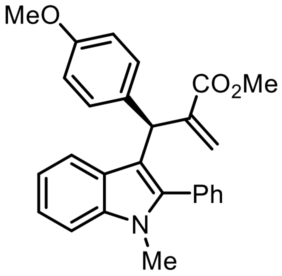

Prepared following **General procedure A**. Purification by flash column chromatography (10% Et_2O in *n*-hexane) gave the title compound (55.3 mg, 67%, 98:2 er, 98:2 rr).

^{1}H NMR (500 MHz, $CDCl_3$) δ 7.51 – 7.40 (m, 4H), 7.37 – 7.29 (m, 3H), 7.21 (ddd, *J* = 8.2, 7.0, 1.2 Hz, 1H), 7.14 – 7.07 (m, 2H), 7.03 (ddd, *J* = 8.1, 7.0, 1.0 Hz, 1H), 6.79 – 6.72 (m, 2H), 6.40 (t, *J* = 1.4 Hz, 1H), 5.54 (t, *J* = 1.6 Hz, 1H), 5.37 (s, 1H), 3.76 (s, 3H), 3.59 (s, 3H), 3.58 (s, 3H) ppm. **^{13}C NMR** (126 MHz, $CDCl_3$) δ 167.8, 157.9, 143.9, 139.0, 137.4, 134.9, 132.0, 130.8, 129.7, 128.4, 128.4, 127.2, 126.9, 121.4, 120.8, 119.4, 113.6, 112.5, 109.5, 55.3, 51.9, 43.8, 31.0 ppm. **Specific rotation** $[\alpha]_D^{23}$ = +68 (c = 1.1, CH_2Cl_2). **HRMS** (ESI) m/z calculated for $C_{27}H_{26}ClNO_3$ $[M+H]^+$, 412.1907 found: 412.1891. **IR (neat)** 3016, 2970, 1738, 1609, 1509, 1365, 1217, 1133, 948, 743, 528 cm^{-1}. **HPLC conditions**: Chiral column IA, hexane: isopropanol = 98:2, flow rate = 0.5 mL/min, wavelength = 230 nm, tR = 13.2 min for major isomer, tR = 9.5 min for minor isomer.

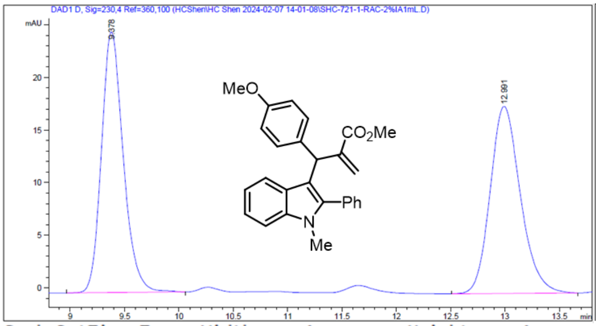

Peak #	RetTime [min]	Type	Width [min]	Area [mAU*s]	Height [mAU]	Area %
1	9.378	BB	0.2129	346.00894	24.77634	50.1379
2	12.991	BB	0.2975	344.10553	17.80319	49.8621

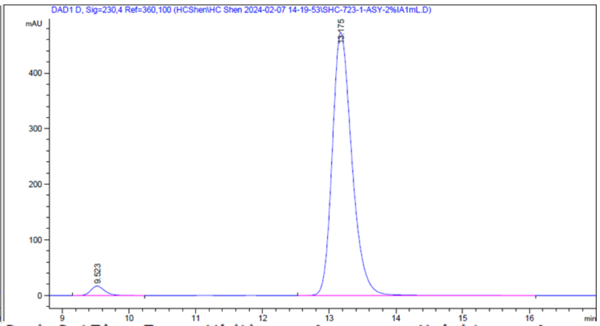

Peak #	RetTime [min]	Type	Width [min]	Area [mAU*s]	Height [mAU]	Area %
1	9.523	BB	0.2152	240.37779	16.97897	2.4263
2	13.175	BB	0.3121	9666.80078	473.59210	97.5737

methyl (*R*)-2-((4-fluorophenyl)(1-methyl-2-phenyl-1H-indol-3-yl)methyl)acrylate (5p)

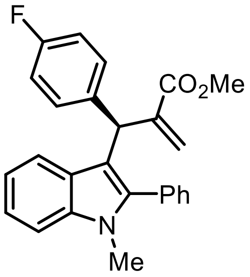

Prepared following **General procedure A**. Purification by flash column chromatography (10% Et_2O in *n*-hexane) gave the title compound (48.3 mg, 61%, 95:5 er, 97:3 rr).

^{1}H NMR (500 MHz, $CDCl_3$) δ 7.50 – 7.40 (m, 4H), 7.35 (d, J = 8.2 Hz, 1H), 7.32 – 7.27 (m, 2H), 7.22 (ddd, J = 8.2, 7.0, 1.1 Hz, 1H), 7.18 – 7.09 (m, 2H), 7.04 (ddd, J = 8.1, 7.0, 1.0 Hz, 1H), 6.93 – 6.85 (m, 2H), 6.43 (t, J = 1.2 Hz, 1H), 5.55 (t, J = 1.5 Hz, 1H), 5.40 (s, 1H), 3.61 (s, 3H), 3.58 (s, 3H) ppm. **^{13}C NMR** (126 MHz, $CDCl_3$) δ 167.6, 161.4 (d, J = 244.0 Hz), 143.4, 139.2, 138.5 (d, J = 3.1 Hz), 137.4, 131.8, 130.7, 130.1 (d, J = 7.8 Hz), 128.5, 128.5, 127.3, 127.1, 121.6, 120.6, 119.5, 115.1 (d, J = 21.3 Hz), 112.1, 109.6, 52.0, 43.9, 31.0 ppm. **Specific rotation** $[\alpha]_D^{23}$ = +36 (c = 1.1, CH_2Cl_2). **HRMS** (ESI) m/z calculated for $C_{26}H_{23}FNO_2$ $[M+H]^+$, 400.1707 found: 400.1690. **IR (neat)** 2950, 1892, 1721, 1605, 1506, 1365, 1219, 1134, 828, 741, 702 cm^{-1}. **HPLC conditions**: Chiral column IA, hexane: isopropanol = 95:5, flow rate = 1 mL/min, wavelength = 254 nm, tR = 6.3 min for major isomer, tR = 5.1 min for minor isomer.

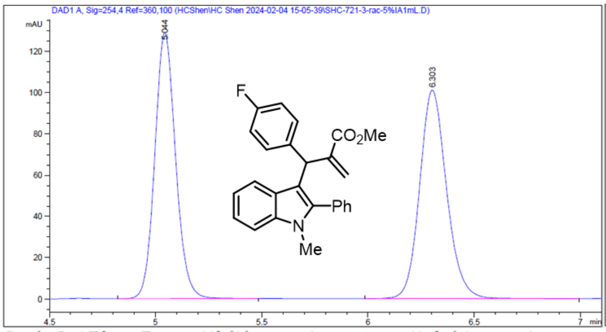

Peak #	RetTime [min]	Type	Width [min]	Area [mAU*s]	Height [mAU]	Area %
1	5.044	BB	0.1032	877.51770	129.18555	49.5864
2	6.303	BB	0.1349	892.15466	101.11275	50.4136

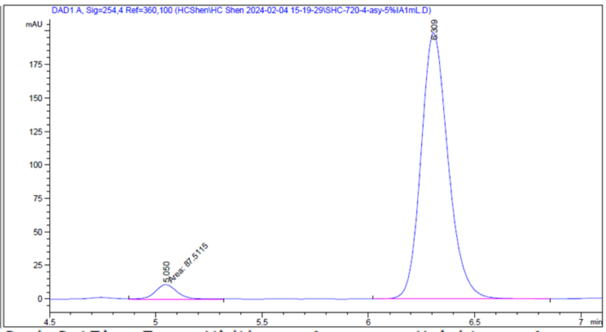

Peak #	RetTime [min]	Type	Width [min]	Area [mAU*s]	Height [mAU]	Area %
1	5.050	MM	0.1297	87.51150	11.24124	4.7409
2	6.309	BB	0.1374	1758.38635	198.49178	95.2591

methyl (*R*)-2-((4-chlorophenyl)(1-methyl-2-phenyl-1H-indol-3-yl)methyl)acrylate (5q)

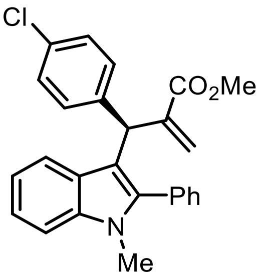

Prepared following **General procedure A**. Purification by flash column chromatography (10% Et_2O in *n*-hexane) gave the title compound (49.5 mg, 60%, 98:2 er, 97:3 rr).

^{1}H NMR (500 MHz, $CDCl_3$) δ 7.50 – 7.40 (m, 4H), 7.35 (d, *J* = 8.3 Hz, 1H), 7.33 – 7.26 (m, 2H), 7.23 (ddd, *J* = 8.2, 7.0, 1.2 Hz, 1H), 7.20 – 7.14 (m, 2H), 7.14 – 7.07 (m, 2H), 7.04 (ddd, *J* = 8.1, 7.0, 1.0 Hz, 1H), 6.44 (t, *J* = 1.2 Hz, 1H), 5.56 (t, *J* = 1.5 Hz, 1H), 5.39 (s, 1H), 3.61 (s, 3H), 3.58 (s, 3H) ppm. **^{13}C NMR** (126 MHz, $CDCl_3$) δ 167.5, 143.1, 141.3, 139.3, 137.4, 131.8, 131.8, 130.7, 130.1, 128.5, 128.5, 128.4, 127.5, 127.0, 121.6, 120.5, 119.6, 111.7, 109.6, 52.0, 44.0, 31.0 ppm. **Specific rotation** $[\alpha]_D^{23}$ = +83 (c = 2.4, CH_2Cl_2). **HRMS** (ESI) m/z calculated for $C_{26}H_{23}ClNO_2$ $[M+H]^+$, 416.1412 found: 416.1393. **IR (neat)** 2970, 1720, 1627, 1466, 1364, 1243, 1133, 1014, 953, 820, 739, 701 cm^{-1}. **HPLC conditions**: Chiral column IA, hexane: isopropanol = 97:3, flow rate = 1 mL/min, wavelength = 254 nm, tR = 7.1 min for major isomer, tR = 5.4 min for minor isomer.

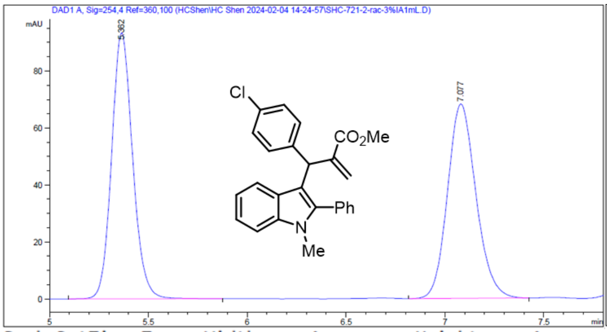

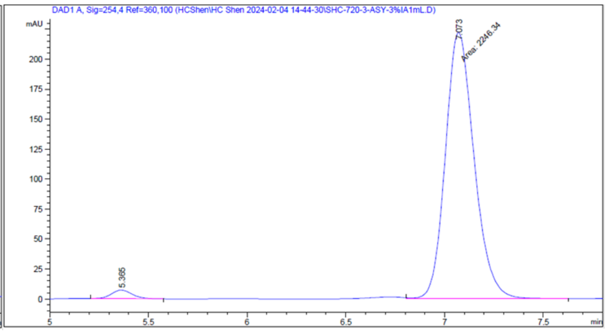

Peak #	RetTime [min]	Type	Width [min]	Area [mAU*s]	Height [mAU]	Area %
1	5.362	BB	0.1141	689.50879	93.46565	50.2065
2	7.077	BB	0.1549	683.83740	68.24067	49.7935

Peak #	RetTime [min]	Type	Width [min]	Area [mAU*s]	Height [mAU]	Area %
1	5.365	BB	0.1106	50.19108	7.09165	2.1855
2	7.073	MM	0.1683	2246.34473	222.42667	97.8145

methyl (*R*)-4-(2-(methoxycarbonyl)-1-(1-methyl-2-phenyl-1H-indol-3-yl)allyl)benzoate

(5r)

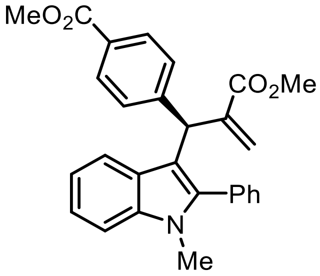

Prepared following **General procedure A**. Purification by flash column chromatography (10% Et_2O in *n*-hexane) gave the title compound (77.2 mg, 88%, 98:2 er, 97:3 rr).

^{1}H NMR (500 MHz, $CDCl_3$) δ 7.91 – 7.85 (m, 2H), 7.49 – 7.42 (m, 3H), 7.43-7.39 (m, 1H), 7.35 (d, *J* = 8.3 Hz, 1H), 7.33 – 7.27 (m, 2H), 7.25 (d, *J* = 8.2 Hz, 2H), 7.22 (ddd, *J* = 8.2, 7.0, 1.1 Hz, 1H), 7.02 (ddd, *J* = 8.1, 7.0, 1.0 Hz, 1H), 6.47 (t, *J* = 1.3 Hz, 1H), 5.57 (t, *J* = 1.5 Hz, 1H), 5.46 (s, 1H), 3.88 (s, 3H), 3.61 (s, 3H), 3.58 (s, 3H) ppm. **^{13}C NMR** (126 MHz, $CDCl_3$) δ 167.4, 167.2, 148.3, 142.7, 139.3, 137.4, 131.7, 130.7, 129.6, 128.7, 128.6, 128.5, 128.1, 127.7, 127.0, 121.6, 120.5, 119.6, 111.5, 109.6, 52.1, 52.0, 44.6, 31.0 ppm. **Specific rotation** $[\alpha]_D^{23}$ = +71 (c = 1.1, CH_2Cl_2). **HRMS** (ESI) m/z calculated for $C_{28}H_{26}NO_4$ $[M+H]^+$, 440.1856 found: 440.1837. **IR (neat)** 3016, 2970, 1738, 1435, 1366, 1217, 900, 750, 527 cm^{-1}. **HPLC conditions**: Chiral column IA, hexane: isopropanol = 95:5, flow rate = 1 mL/min, wavelength = 254 nm, tR = 19.6 min for major isomer, tR = 9.0 min for minor isomer.

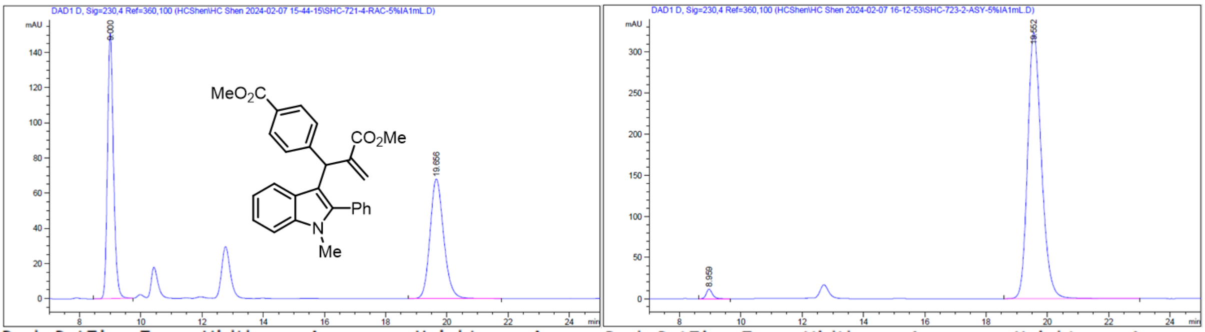

Peak #	RetTime [min]	Type	Width [min]	Area [mAU*s]	Height [mAU]	Area %
1	9.000	BB	0.2085	2049.14063	150.83849	49.2535
2	19.656	BB	0.4758	2111.25513	68.08897	50.7465

Peak #	RetTime [min]	Type	Width [min]	Area [mAU*s]	Height [mAU]	Area %
1	8.959	BB	0.2084	156.13440	11.50207	1.5197
2	19.552	BB	0.4788	1.01178e4	323.58710	98.4803

**methyl (*R*)-4-(1-(2-(4-bromophenyl)-1-methyl-1H-indol-3-yl)-2-(methoxycarbonyl)allyl)

benzoate (5s)

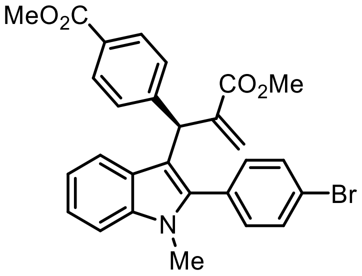

Prepared following **General procedure A**. Purification by flash column chromatography (10% Et_2O in *n*-hexane) gave the title compound (90.5 mg, 88%, 99:1 er, 92:8 rr).

^{1}H NMR (400 MHz, $CDCl_3$) δ 7.87 (d, J = 8.3 Hz, 2H), 7.58 (d, J = 8.7 Hz, 2H), 7.41 (d, J = 8.0, 1H), 7.34 (d, J = 8.4, 1H), 7.26 – 7.17 (m, 3H), 7.15 (d, J = 7.8 Hz, 2H), 7.02 (ddd, J = 8.1, 7.0, 1.0 Hz, 1H), 6.45 (s, 1H), 5.52 (t, J = 1.4 Hz, 1H), 5.40 (s, 1H), 3.88 (s, 3H), 3.61 (s, 3H), 3.56 (s, 3H) ppm. **^{13}C NMR** (151 MHz, $CDCl_3$) δ 167.3, 167.2, 148.0, 142.7, 138.0, 137.5, 132.3, 131.8, 130.7, 129.7, 128.7, 128.3, 127.7, 126.9, 123.1, 122.0, 120.6, 119.8, 111.9, 109.7, 52.1, 44.6, 31.0 ppm. **Specific rotation** $[\alpha]_D^{23}$ = +74 (c = 1.7, CH_2Cl_2). **HRMS** (ESI) m/z calculated for $C_{28}H_{24}BrNO_2$ $[M+H]^+$, 518.0961 found: 518.0967. **IR (neat)** 2970, 1720, 1609, 1435, 1279, 1217, 1106, 743 cm^{-1}. **HPLC conditions**: Chiral column IA, hexane: isopropanol = 95:5, flow rate = 1 mL/min, wavelength = 210 nm, tR = 11.1 min for major isomer, tR = 9.4 min for minor isomer.

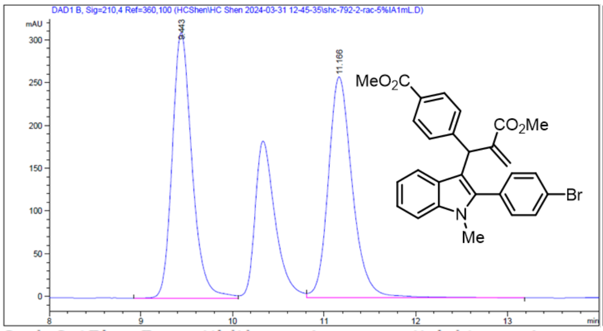

Peak #	RetTime [min]	Type	Width [min]	Area [mAU*s]	Height [mAU]	Area %
1	9.443	BV	0.2290	4650.12842	310.00323	49.5120
2	11.166	VB	0.2782	4741.79688	258.23544	50.4880

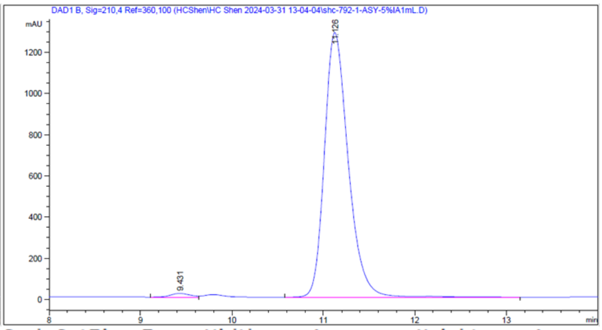

Peak #	RetTime [min]	Type	Width [min]	Area [mAU*s]	Height [mAU]	Area %
1	9.431	BV	0.2188	254.14424	17.98616	1.0836
2	11.126	BV R	0.2761	2.32001e4	1283.37366	98.9164

methyl (*R*)-2-((3-bromophenyl)(1-methyl-2-phenyl-1H-indol-3-yl)methyl)acrylate (5t)

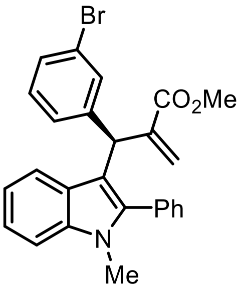

Prepared following **General procedure A**. Purification by flash column chromatography (10% Et_2O in *n*-hexane) gave the title compound (84.4 mg, 92%, 84:16 er, 91:9 rr).

^{1}H NMR (500 MHz, $CDCl_3$) δ 7.51 – 7.41 (m, 4H), 7.36 (d, *J* = 8.2 Hz, 1H), 7.33 – 7.25 (m, 4H), 7.24 (ddd, *J* = 8.2, 7.0, 1.1 Hz, 1H), 7.14 (d, *J* = 8.0 Hz, 1H), 7.11 – 7.02 (m, 2H), 6.47 (s, 1H), 5.59 (t, *J* = 1.5 Hz, 1H), 5.42 (s, 1H), 3.62 (s, 3H), 3.58 (s, 3H) ppm. **^{13}C NMR** (126 MHz, $CDCl_3$) δ 167.4, 145.2, 142.7, 139.3, 137.4, 131.7, 131.7, 130.7, 129.8, 129.3, 128.6, 128.5, 127.8, 127.3, 127.0, 122.4, 121.6, 120.4, 119.6, 111.6, 109.6, 52.0, 44.2, 31.0 ppm. **Specific rotation** $[\alpha]_D^{23}$ = +52 (c = 1.7, CH_2Cl_2). **HRMS** (ESI) m/z calculated for $C_{26}H_{23}BrNO_2$ $[M+H]^+$, 460.0907 found: 460.0894. **IR (neat)** 2970, 1721, 1566, 1467, 1364, 1134, 738, 701 cm^{-1}. **HPLC conditions**: Chiral column IA, hexane: isopropanol = 98:1, flow rate = 1 mL/min, wavelength = 254 nm, tR = 6.4 min for major isomer, tR =6.0 min for minor isomer.

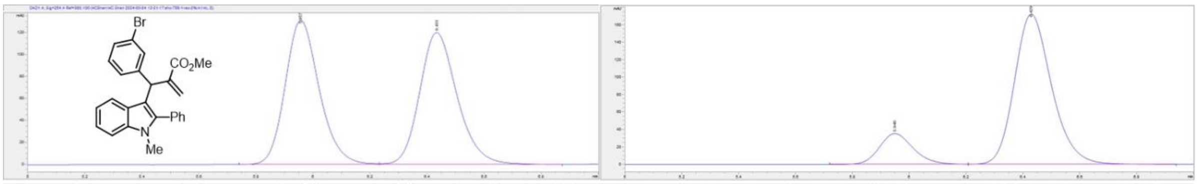

#	Time	Area	Height	Width	Area%	Symmetry
1	5.957	1092.1	131.4	0.1269	49.847	0.805
2	6.433	1098.8	120.3	0.1405	50.153	0.785

#	Time	Area	Height	Width	Area%	Symmetry
1	5.949	293.8	35.4	0.1269	15.766	0.81
2	6.429	1569.9	172.5	0.1402	84.234	0.783

methyl (*R*)-2-(furan-2-yl(1-methyl-2-phenyl-1H-indol-3-yl)methyl)acrylate (5u)

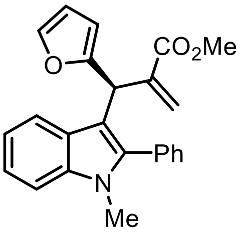

Prepared following **General procedure A**. Purification by flash column chromatography (10%

Et$_2$O in *n*-hexane) gave the title compound (62.0 mg, 84%, 95:5 er, 95:5 rr).

^{1}H NMR (500 MHz, CDCl$_3$) δ 7.55 – 7.43 (m, 4H), 7.43 – 7.37 (m, 2H), 7.36 (d, *J* = 8.2 Hz, 1H), 7.31 (dd, *J* = 1.8, 0.9 Hz, 1H), 7.24 (ddd, *J* = 8.2, 7.0, 1.1 Hz, 1H), 7.07 (ddd, *J* = 8.1, 7.0, 1.0 Hz, 1H), 6.38 (t, *J* = 1.2 Hz, 1H), 6.27 (dd, *J* = 3.2, 1.8 Hz, 1H), 6.05 (dt, *J* = 3.2, 0.9 Hz, 1H), 5.62 (t, *J* = 1.4 Hz, 1H), 5.44 (d, *J* = 1.0 Hz, 1H), 3.62 (s, 3H), 3.60 (s, 3H) ppm. **^{13}C NMR** (126 MHz, CDCl$_3$) δ 167.2, 155.6, 141.5, 141.0, 139.1, 137.4, 131.6, 130.8, 128.5, 128.5, 126.9, 126.7, 121.6, 120.6, 119.5, 110.2, 110.0, 109.6, 107.5, 51.9, 38.8, 31.0 ppm. **Specific rotation** $[\alpha]_D^{23}$ = +13 (c = 1.3, CH$_2$Cl$_2$). **HRMS** (ESI) m/z calculated for $C_{24}H_{22}NO_3$ [M+H]$^+$, 372.1594 found: 372.1576. **IR (neat)** 2970, 1721, 1628, 1467, 1365, 1217, 736, 701 cm^{-1}. **HPLC conditions**: Chiral column IA, hexane: isopropanol = 98:2, flow rate = 1 mL/min, wavelength = 254 nm, tR = 7.9 min for major isomer, tR = 7.3 min for minor isomer.

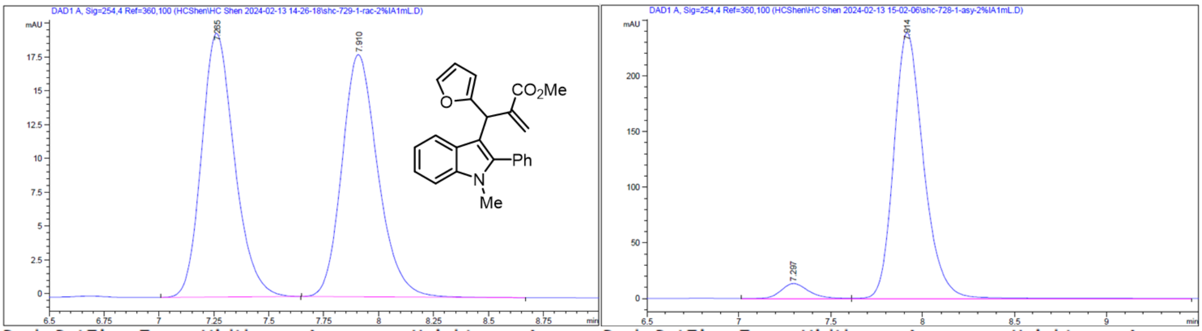

Peak #	RetTime [min]	Type	Width [min]	Area [mAU*s]	Height [mAU]	Area %
1	7.265	BB	0.1594	203.02901	19.50860	49.9125
2	7.910	BB	0.1747	203.74091	17.90863	50.0875

Peak #	RetTime [min]	Type	Width [min]	Area [mAU*s]	Height [mAU]	Area %
1	7.297	BV	0.1555	137.16711	13.39534	4.8687
2	7.914	VB	0.1709	2680.17114	238.90988	95.1313

methyl (R)-2-((1-methyl-2-phenyl-1H-indol-3-yl)(thiophen-2-yl)methyl)acrylate (5v)

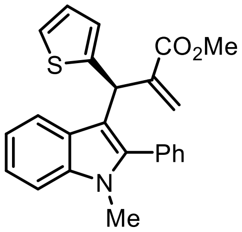

Prepared following **General procedure A**. Purification by flash column chromatography (10% Et$_2$O in *n*-hexane) gave the title compound (53.5 mg, 69%, 95:5 er, 97:3 rr).

^{1}H NMR (500 MHz, $CDCl_3$) δ 7.54 (d, *J* = 8.1 Hz, 1H), 7.52 – 7.41 (m, 3H), 7.40 – 7.33 (m, 3H), 7.23 (ddd, *J* = 8.2, 7.0, 1.2 Hz, 1H), 7.13 (dd, *J* = 5.1, 1.2 Hz, 1H), 7.06 (ddd, *J* = 8.0, 7.0, 1.0 Hz, 1H), 6.89 (dd, *J* = 5.1, 3.5 Hz, 1H), 6.82 (dt, *J* = 3.5, 1.2 Hz, 1H), 6.42 (t, *J* = 1.2 Hz, 1H), 5.76 (t, *J* = 1.4 Hz, 1H), 5.60 (d, *J* = 1.4 Hz, 1H), 3.60 (s, 3H), 3.60 (s, 3H) ppm. **^{13}C NMR** (126 MHz, $CDCl_3$) δ 167.3, 146.8, 143.1, 139.0, 137.4, 131.7, 130.8, 128.5, 128.5, 126.7, 126.5, 125.7, 124.1, 121.6, 120.8, 119.5, 112.1, 109.6, 52.0, 39.8, 31.0 ppm. **Specific rotation** $[\alpha]_D^{23}$ = -13 (c = 1.1, CH_2Cl_2). **HRMS** (ESI) m/z calculated for $C_{24}H_{22}NO_2S$ $[M+H]^+$, 388.1366 found: 388.1349. **IR (neat)** 2948, 1720, 1627, 1466, 1258, 1136, 824, 739, 700 cm^{-1}. **HPLC conditions**: Chiral column IA, hexane: isopropanol = 98:2, flow rate = 1 mL/min, wavelength = 254 nm, tR = 8.9 min for major isomer, tR = 6.8 min for minor isomer.

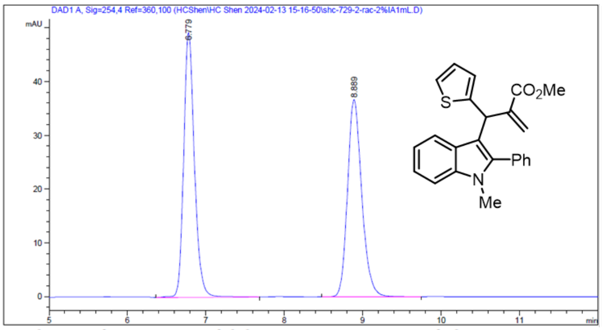

Peak #	RetTime [min]	Type	Width [min]	Area [mAU*s]	Height [mAU]	Area %
1	6.779	BB	0.1452	468.89099	49.21002	50.2526
2	8.889	BB	0.1936	464.17673	36.69421	49.7474

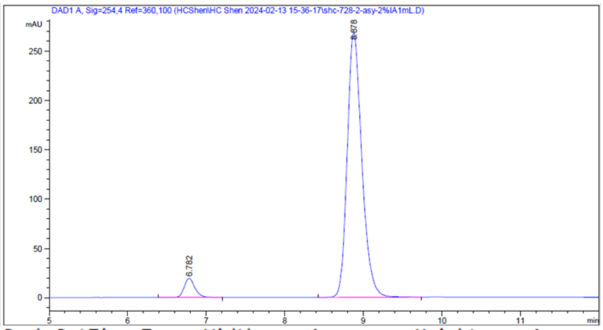

Peak #	RetTime [min]	Type	Width [min]	Area [mAU*s]	Height [mAU]	Area %
1	6.782	BB	0.1447	184.03244	19.38829	5.0409
2	8.878	BB	0.1989	3466.74731	268.05771	94.9591

methyl 2-((5a*S*, 12*R*)-4',4',5,5',5'-pentamethyl-5a-phenyl-5,5a,12,12a-tetrahydro-6l4,7l4-spiro[pyrido[1',2':1,6][1,2]azaborinino[3,4-b]indole-6,2'-[1,3,2]dioxaborolan]-12-yl)acrylate (5w)

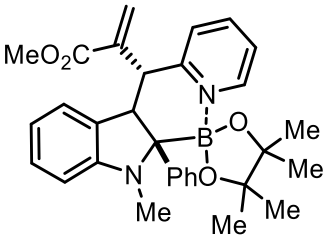

Prepared following **General procedure C**. Purification by flash column chromatography (20% ethyl acetate in *n*-hexane) gave the title compound (58.0 mg branched + 69.2 mg linear, > 99%, 92:8 er of branched, >95:5 dr of branched, 46:54 rr).

^{1}H NMR (500 MHz, $CDCl_3$) δ 9.17-9.16 (m, 1H), 7.64 (td, J = 7.8, 1.8 Hz, 1H), 7.55 – 7.49 (m, 2H), 7.32 – 7.26 (m, 1H), 7.22-7.19 (m, 3H), 7.11 – 7.04 (m, 1H), 6.90 – 6.81 (m, 2H), 6.65 (d, J = 7.2 Hz, 1H), 6.17 (t, J = 7.3 Hz, 1H), 6.08 (d, J = 6.0 Hz, 1H), 5.92 (d, J = 7.8 Hz, 1H), 5.72 (s, 1H), 3.80 (s, 3H), 3.76 (d, J = 6.0 Hz, 1H), 2.91 (s, 3H), 1.20 (s, 6H), 0.66 (s, 6H) ppm. **^{13}C NMR** (126 MHz, $CDCl_3$) δ 168.0, 159.1, 153.2, 150.0, 143.0, 139.2, 135.5, 131.4, 128.3, 127.3, 127.2, 126.9, 124.8, 122.8, 121.6, 112.9, 102.4, 80.1, 55.8, 52.5, 44.7, 32.4, 26.7, 25.8 ppm. **Specific rotation** $[\alpha]_D^{23}$ = +54 (c = 1.2, CH_2Cl_2). **HRMS** (ESI) m/z calculated for $C_{31}H_{35}BN_2O_4$ $[M+H]^+$, 511.2763 found: 511.2738. **IR (neat)** 2987, 1717, 1605, 1497, 1281, 1141, 1013, 754 cm^{-1}. **HPLC conditions**: Chiral column IC, hexane: isopropanol = 95:5, flow rate = 1 mL/min, wavelength = 230 nm, tR = 6.8 min for major isomer, tR = 5.6 min for minor isomer.

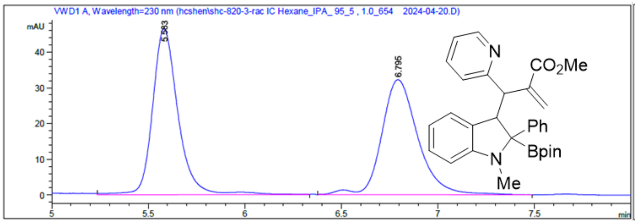

Peak #	RetTime [min]	Type	Width [min]	Area [mAU*s]	Height [mAU]	Area %
1	5.583	VV R	0.1406	426.59067	46.24540	50.4881
2	6.795	VB R	0.1960	418.34235	32.07048	49.5119

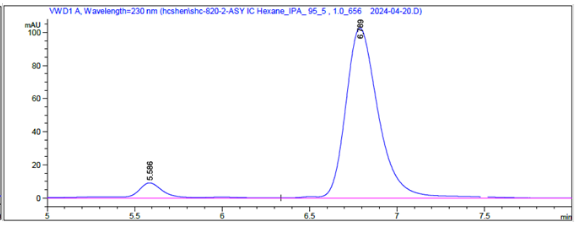

Peak #	RetTime [min]	Type	Width [min]	Area [mAU*s]	Height [mAU]	Area %
1	5.586	VV R	0.1674	121.59101	9.29202	8.2490
2	6.789	VB	0.2031	1352.42200	102.72244	91.7510

tert-butyl (*R*)-2-((1-methyl-2-phenyl-1H-indol-3-yl)(phenyl)methyl)acrylate (5x)

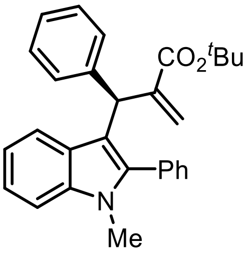

Prepared following **General procedure A**. Purification by flash column chromatography (10% Et_2O in *n*-hexane) gave the title compound (35.7 mg, 42%, 91:9 er, 99:1 rr).

^{1}H NMR (500 MHz, $CDCl_3$) δ 7.49 – 7.38 (m, 4H), 7.35 – 7.29 (m, 3H), 7.23 – 7.14 (m, 5H), 7.14 – 7.07 (m, 1H), 7.00 (ddd, *J* = 8.1, 7.0, 1.0 Hz, 1H), 6.30 (t, *J* = 1.5 Hz, 1H), 5.42 (t, *J* = 1.7 Hz, 1H), 5.35 (s, 1H), 3.58 (s, 3H), 1.24 (s, 9H) ppm. **^{13}C NMR** (126 MHz, $CDCl_3$) δ 166.8,

145.2, 143.2, 138.7, 137.4, 132.0, 130.8, 128.6, 128.4, 128.3, 128.1, 127.3, 126.0, 125.9, 121.5, 120.9, 119.4, 113.3, 109.4, 80.5, 44.8, 31.0, 27.9 ppm. **Specific rotation** $[\alpha]_D^{23}$ = +93 (c = 0.8, CH_2Cl_2). **HRMS** (ESI) m/z calculated for $C_{29}H_{30}NO_2$ $[M+H]^+$, 424.2271 found: 424.2256. **IR (neat)** 2971, 1714, 1629, 1467, 1166, 1217, 1136, 740, 701 cm^{-1}. **HPLC conditions**: Chiral column IA, hexane: isopropanol = 99:1, flow rate = 1 mL/min, wavelength = 230 nm, tR = 5.0 min for major isomer, tR = 5.4 min for minor isomer.

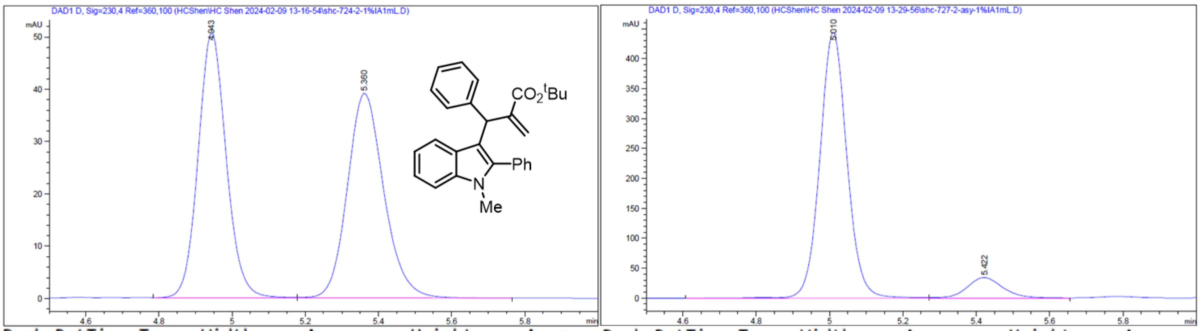

Peak #	RetTime [min]	Type	Width [min]	Area [mAU*s]	Height [mAU]	Area %
1	4.943	BV	0.0821	269.69647	50.65084	49.9408
2	5.360	VB	0.1066	270.33606	39.14142	50.0592

Peak #	RetTime [min]	Type	Width [min]	Area [mAU*s]	Height [mAU]	Area %
1	5.010	VV R	0.0794	2269.30859	445.36392	90.8880
2	5.422	VB	0.1019	227.51115	34.07576	9.1120

tert-butyl (*R*)-2-((4-chlorophenyl)(1-methyl-2-phenyl-1H-indol-3-yl)methyl)acrylate (5y)

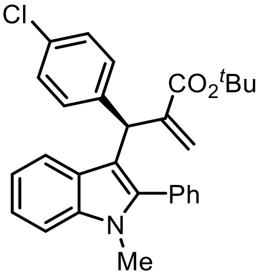

Prepared following **General procedure A**. Purification by flash column chromatography (10% Et_2O in *n*-hexane) gave the title compound (59.1 mg, 65%, 95:5 er, 99:1 rr).

^{1}H NMR (500 MHz, $CDCl_3$) δ 7.48 – 7.37 (m, 4H), 7.33 (d, *J* = 8.1 Hz, 1H), 7.31 – 7.26 (m, 2H), 7.21 (ddd, *J* = 8.2, 7.0, 1.1 Hz, 1H), 7.17 – 7.11 (m, 2H), 7.12 – 7.05 (m, 2H), 7.03 (ddd, *J* = 8.1, 7.0, 1.0 Hz, 1H), 6.32 (t, *J* = 1.4 Hz, 1H), 5.45 (t, *J* = 1.7 Hz, 1H), 5.34 (s, 1H), 3.58 (s, 3H), 1.27 (s, 9H) ppm. **^{13}C NMR** (126 MHz, $CDCl_3$) δ 166.5, 144.7, 141.8, 138.8, 137.4, 131.8, 131.6, 130.7, 129.9, 128.4, 128.2, 127.0, 126.3, 121.6, 120.6, 119.5, 112.8, 109.5, 80.7, 44.2, 31.0, 27.9 ppm. **Specific rotation** $[\alpha]_D^{23}$ = +127 (c = 1.2, CH_2Cl_2). **HRMS** (ESI) m/z

calculated for $C_{29}H_{28}ClNO_2$ $[M+H]^+$, 458.1881 found: 458.1880. **IR (neat)** 2970, 1739, 1629, 1466, 1366, 1217, 1187, 740, 701, 527 cm^{-1}. **HPLC conditions**: Chiral column IC, hexane: isopropanol = 99:1, flow rate = 1 mL/min, wavelength = 254 nm, tR = 5.8 min for major isomer, tR = 6.5 min for minor isomer.

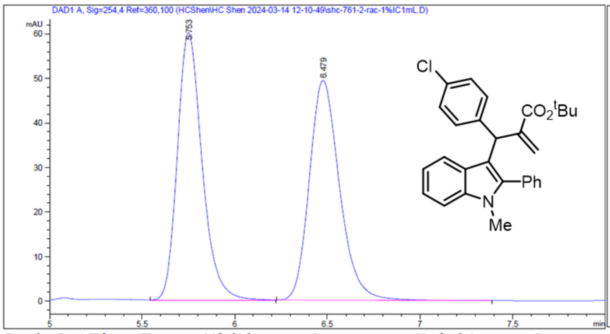

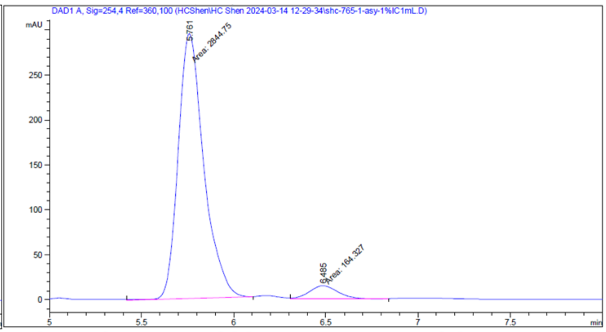

Peak #	RetTime [min]	Type	Width [min]	Area [mAU*s]	Height [mAU]	Area %
1	5.753	BB	0.1420	556.01776	60.04440	49.8959
2	6.479	BB	0.1741	558.33862	49.32523	50.1041

Peak #	RetTime [min]	Type	Width [min]	Area [mAU*s]	Height [mAU]	Area %
1	5.761	MM	0.1609	2844.74927	294.74820	94.5390
2	6.485	MM	0.1858	164.32675	14.73804	5.4610

(*R*)-2-((1-methyl-2-phenyl-1H-indol-3-yl)(phenyl)methyl)acrylonitrile (5z)

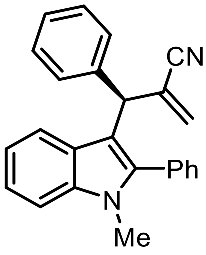

Prepared following **General procedure A**. Purification by flash column chromatography (10% Et_2O in *n*-hexane) gave the title compound (31.0 mg, 44%, 86:14 er, 96:4 rr).

^{1}H NMR (500 MHz, $CDCl_3$) δ 7.53 – 7.43 (m, 4H), 7.41 – 7.28 (m, 3H), 7.31 – 7.20 (m, 6H), 7.08 (ddd, *J* = 8.1, 7.0, 1.0 Hz, 1H), 6.08 (d, *J* = 1.6 Hz, 1H), 5.64 (d, *J* = 1.8 Hz, 1H), 5.04 (t, *J* = 1.8 Hz, 1H), 3.60 (s, 3H) ppm. **^{13}C NMR** (126 MHz, $CDCl_3$) δ 139.9, 139.5, 137.5, 131.7, 131.3, 130.8, 128.9, 128.8, 128.7, 128.6, 127.1, 126.5, 126.4, 122.0, 120.6, 119.9, 119.2, 109.8, 47.4, 31.0 ppm. **Specific rotation** $[\alpha]_D^{23}$ = -4 (c = 1.0, CH_2Cl_2). **HRMS** (ESI) m/z calculated for $C_{25}H_{21}N_2$ $[M+H]^+$, 349.1699 found: 349.1684. **IR (neat)** 2920, 2218, 1738, 1602, 1467, 1365, 1217, 740, 700 cm^{-1}. **HPLC conditions**: Chiral column IA, hexane: isopropanol = 98:2,

flow rate = 1 mL/min, wavelength = 254 nm, tR = 8.1 min for major isomer, tR = 12.7 min for minor isomer.

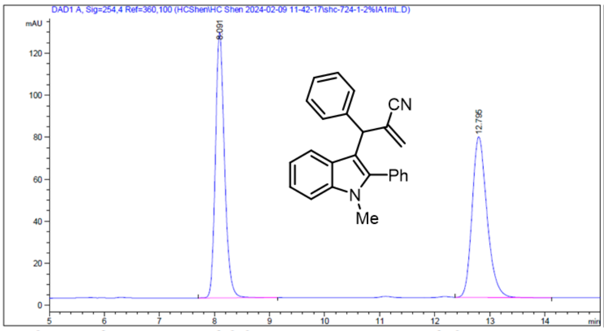

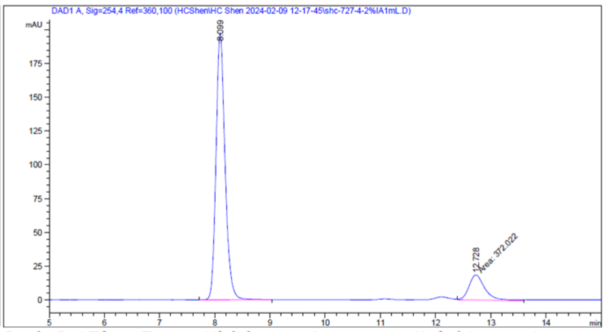

Peak #	RetTime [min]	Type	Width [min]	Area [mAU*s]	Height [mAU]	Area %
1	8.091	BB	0.1757	1447.66504	126.31271	50.2027
2	12.795	BB	0.2870	1435.97290	76.49577	49.7973

Peak #	RetTime [min]	Type	Width [min]	Area [mAU*s]	Height [mAU]	Area %
1	8.099	BB	0.1727	2243.36792	197.28024	85.7757
2	12.728	MM	0.3337	372.02194	18.58257	14.2243

methyl (*R*)-2-((4-chlorophenyl)(5-methoxy-1-methyl-2-phenyl-1H-indol-3-yl)methyl) acrylate (5aa)

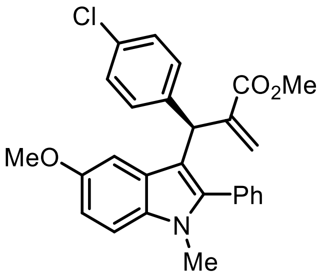

Prepared following **General procedure B**. Purification by flash column chromatography (10% Et_2O in *n*-hexane) gave the title compound (68.3 mg, 77%, 97:3 er, 86:14 rr).

^{1}H NMR (500 MHz, $CDCl_3$) δ 7.49 – 7.39 (m, 3H), 7.33 – 7.25 (m, 2H), 7.23 (d, *J* = 8.8 Hz, 1H), 7.21 – 7.14 (m, 2H), 7.14 – 7.08 (m, 2H), 6.88 (dd, *J* = 8.8, 2.5 Hz, 1H), 6.85 (d, *J* = 2.4 Hz, 1H), 6.45 (t, J = 1.2 Hz, 1H), 5.57 (t, J = 1.5 Hz, 1H), 5.37 (s, 1H), 3.74 (s, 3H), 3.62 (s, 3H), 3.55 (s, 3H) ppm. **^{13}C NMR** (126 MHz, $CDCl_3$) δ 167.5, 153.8, 143.0, 141.3, 139.8, 132.9, 131.8, 131.8, 130.6, 130.0, 128.5, 128.3, 127.4, 127.3, 111.3, 111.3, 110.2, 102.7, 55.9, 52.0, 44.0, 31.1 ppm. **Specific rotation** $[\alpha]_D^{23}$ = +56 (c = 1.0, CH_2Cl_2). **HRMS** (ESI) m/z calculated for $C_{27}H_{25}ClNO_3$ $[M+H]^+$, 446.1517 found: 446.1498. **IR (neat)** 2949, 1721, 1620, 1481, 1229, 1135, 700, 510cm^{-1}. **HPLC conditions**: Chiral column IA, hexane: isopropanol = 95:5, flow

rate = 1 mL/min, wavelength = 254 nm, tR = 9.5 min for major isomer, tR = 6.1 min for minor isomer.

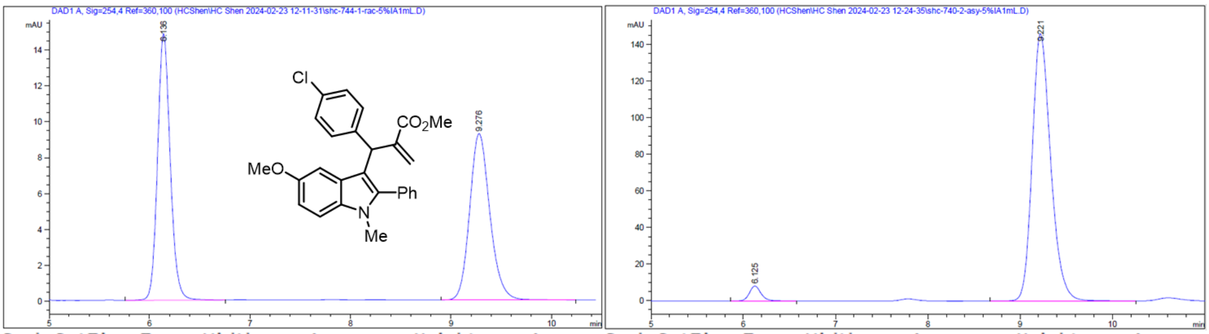

Peak #	RetTime [min]	Type	Width [min]	Area [mAU*s]	Height [mAU]	Area %
1	6.136	BB	0.1341	129.77675	14.83305	49.9828
2	9.276	BB	0.2154	129.86584	9.26975	50.0172

Peak #	RetTime [min]	Type	Width [min]	Area [mAU*s]	Height [mAU]	Area %
1	6.125	BB	0.1323	69.53336	8.08683	3.2960
2	9.221	BB	0.2130	2040.08923	145.97807	96.7040

methyl (*R*)-2-((4-chlorophenyl)(5-fluoro-1-methyl-2-phenyl-1H-indol-3-yl)methyl) acrylate (5bb)

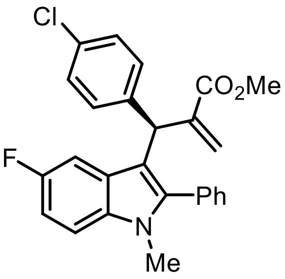

Prepared following **General procedure B**. Purification by flash column chromatography (10% Et_2O in *n*-hexane) gave the title compound (60.3 mg, 70%, 98:2 er, 94:6 rr).

^{1}H NMR (500 MHz, $CDCl_3$) δ 7.50 – 7.39 (m, 3H), 7.33 – 7.20 (m, 3H), 7.21 – 7.15 (m, 2H), 7.13 – 7.05 (m, 3H), 6.96 (td, *J* = 9.0, 2.5 Hz, 1H), 6.44 (t, *J* = 1.2 Hz, 1H), 5.51 (t, *J* = 1.4 Hz, 1H), 5.34 (s, 1H), 3.61 (s, 3H), 3.56 (s, 3H) ppm. **^{13}C NMR** (126 MHz, $CDCl_3$) δ 167.4, 157.4 (d, *J* = 234.1 Hz), 142.8, 141.0, 140.90, 134.1, 132.0, 131.4, 130.6, 123.0, 128.8, 128.6, 128.5, 127.5, 127.1 (d, *J* = 9.9 Hz), 111.7 (d, *J* = 4.8 Hz), 110.2 (d, *J* = 9.6 Hz), 109.9 (d, *J* = 26.2 Hz), 105.3 (d, *J* = 24.1 Hz), 52.0, 43.9, 31.2 ppm. **Specific rotation** $[\alpha]_D^{23}$ = +69 (c = 1.3, CH_2Cl_2). **HRMS** (ESI) m/z calculated for $C_{26}H_{22}ClFNO_2$ $[M+H]^+$, 434.1318 found: 434.1303. **IR (neat)** 2970. 1738, 1624, 1479, 1366, 1229, 915, 527 cm^{-1}. **HPLC conditions**: Chiral column IA,

hexane: isopropanol = 95:5, flow rate = 1 mL/min, wavelength = 254 nm, tR = 6.5 min for major isomer, tR = 5.5 min for minor isomer.

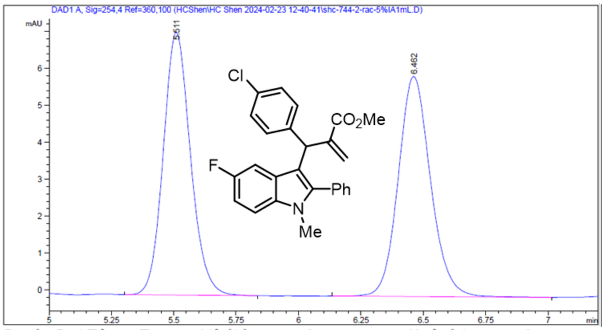

Peak #	RetTime [min]	Type	Width [min]	Area [mAU*s]	Height [mAU]	Area %
1	5.511	BB	0.1166	54.11318	7.12837	50.1922
2	6.462	BB	0.1390	53.69878	5.96811	49.8078

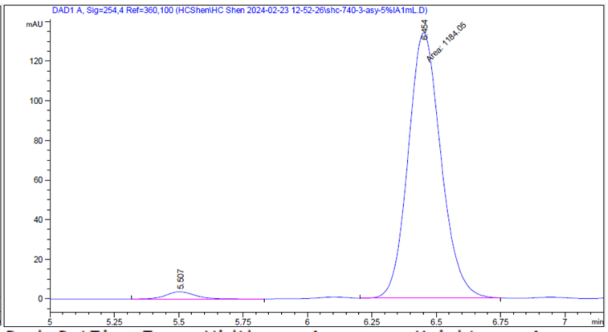

Peak #	RetTime [min]	Type	Width [min]	Area [mAU*s]	Height [mAU]	Area %
1	5.507	BB	0.1165	27.14546	3.57750	2.2412
2	6.454	MM	0.1472	1184.05273	134.05763	97.7588

methyl (*R*)-2-((1-benzyl-2-phenyl-1H-indol-3-yl)(4-chlorophenyl)methyl)acrylate (5cc)

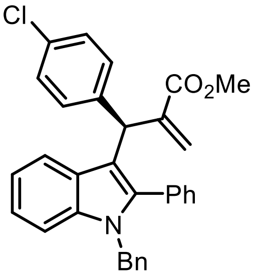

Prepared following **General procedure B**. Purification by flash column chromatography (10% Et_2O in *n*-hexane) gave the title compound (75.8 mg, 77%, 94:6 er, >95:5 rr).

^{1}H NMR (500 MHz, $CDCl_3$) δ 7.47 (d, J = 8.1 Hz, 1H), 7.42 – 7.31 (m, 3H), 7.26 – 7.15 (m, 8H), 7.16 – 7.10 (m, 3H), 7.04 (ddd, J = 8.1, 7.0, 1.1 Hz, 1H), 6.92 – 6.84 (m, 2H), 6.46 (s, 1H), 5.57 (s, 1H), 5.40 (s, 2H), 5.20 (s, 2H), 3.62 (s, 3H) ppm. **^{13}C NMR** (126 MHz, $CDCl_3$) δ 167.5, 143.1, 141.2, 139.4, 138.2, 137.0, 131.9, 131.6, 130.7, 130.1, 128.7, 128.7, 128.5, 128.4, 127.5, 127.4, 127.2, 126.1, 121.9, 120.6, 119.9, 112.4, 110.6, 52.0, 47.7, 44.1 ppm. **Specific rotation** $[\alpha]_D^{23}$ = +57 (c = 0.9, CH_2Cl_2). **HRMS** (ESI) m/z calculated for $C_{32}H_{27}ClNO_2$ $[M+H]^+$, 492.1725 found: 492.1737. **IR (neat)** 2950, 1721, 1627, 1461, 1342, 1248, 1137, 820, 741 cm^{-1}. **HPLC conditions**: Chiral column IA, hexane: isopropanol = 98:2, flow rate = 1 mL/min, wavelength = 254 nm, tR = 9.3 min for major isomer, tR = 7.6 min for minor isomer.

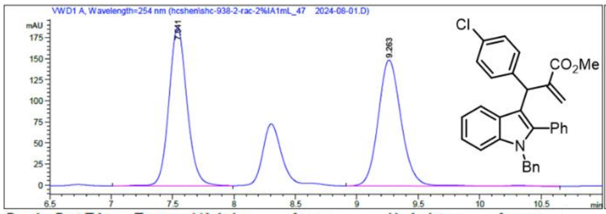

Peak #	RetTime [min]	Type	Width [min]	Area [mAU*s]	Height [mAU]	Area %
1	7.541	BV	0.1612	1932.77991	187.18166	49.7525
2	9.263	BV R	0.1972	1952.00989	149.12210	50.2475

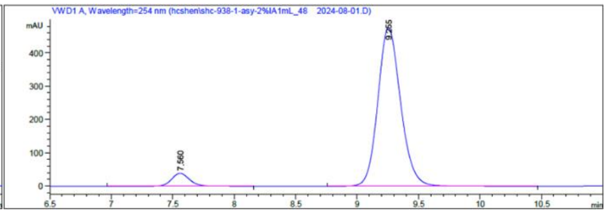

Peak #	RetTime [min]	Type	Width [min]	Area [mAU*s]	Height [mAU]	Area %
1	7.560	BB	0.1611	400.46536	38.82108	6.1357
2	9.255	BB	0.1970	6126.32178	475.04922	93.8643

methyl (*R*)-2-((4-chlorophenyl)(1-(4-methoxybenzyl)-2-phenyl-1H-indol-3-yl)methyl)acrylate (5dd)

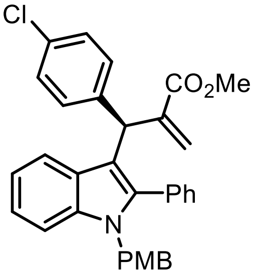

Prepared following **General procedure B**. Purification by flash column chromatography (10% Et_2O in *n*-hexane) gave the title compound (75.4 mg, 72%, 94:6 er, >95:5 rr).

^{1}H NMR (500 MHz, $CDCl_3$) δ 7.45 (d, J = 8.1 Hz, 1H), 7.42 – 7.31 (m, 3H), 7.25 – 7.14 (m, 4H), 7.16 – 7.08 (m, 3H), 7.02 (ddd, J = 8.2, 7.1, 1.2 Hz, 1H), 6.83 – 6.78 (m, 2H), 6.78 – 6.72 (m, 2H), 6.44 (s, 1H), 5.55 (s, 1H), 5.38 (s, 1H), 5.13 (s, 2H), 3.75 (s, 3H), 3.61 (s, 3H) ppm. **^{13}C NMR** (126 MHz, $CDCl_3$) δ 167.5, 158.8, 143.1, 141.2, 139.4, 137.0, 131.9, 131.6, 130.7, 130.3, 130.1, 128.6, 128.5, 128.4, 127.5, 127.4, 127.3, 121.8, 120.6, 119.8, 114.1, 112.4, 110.6, 55.4, 52.0, 47.1, 44.1 ppm. **Specific rotation** $[\alpha]_D^{23}$ = +67 (c = 0.7, CH_2Cl_2). **HRMS** (ESI) m/z calculated for $C_{33}H_{29}ClNO_3$ $[M+H]^+$, 522.1830 found: 522.1814. **IR (neat)** 2952, 1721, 1513, 1462, 1342, 1247, 1136, 1033, 820 cm^{-1}. **HPLC conditions**: Chiral column IA, hexane: isopropanol = 98:2, flow rate = 1 mL/min, wavelength = 254 nm, tR = 14.3 min for major isomer, tR = 10.6 min for minor isomer.

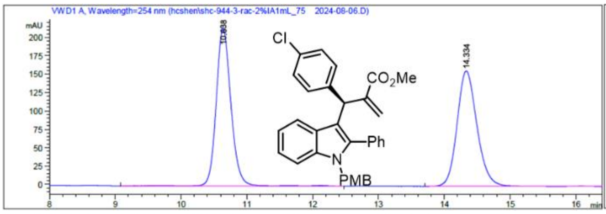

Peak #	RetTime [min]	Type	Width [min]	Area [mAU*s]	Height [mAU]	Area %
1	10.638	VV R	0.2378	3349.30176	214.87013	50.1532
2	14.334	BV R	0.3227	3328.84595	156.82201	49.8468

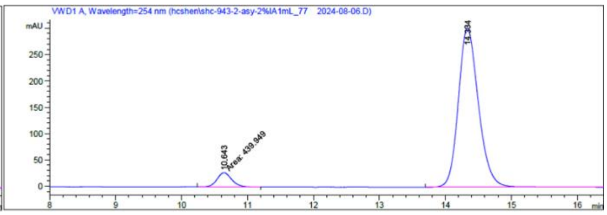

Peak #	RetTime [min]	Type	Width [min]	Area [mAU*s]	Height [mAU]	Area %
1	10.643	MM	0.2671	439.94919	27.45102	6.3993
2	14.334	BV R	0.3244	6434.95801	301.92188	93.6007

methyl (*R*,*E*)-3-(1-methyl-2-phenyl-1H-indol-3-yl)-2-methylene-5-phenylpent-4-enoate (5ee)

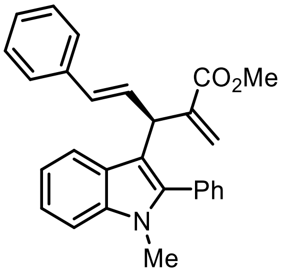

Prepared following **General procedure B**. Purification by flash column chromatography (20% DCM in toluene) gave the title compound (29.1 mg, 36%, 68:32 er, 42:58 rr).

^{1}H NMR (500 MHz, $CDCl_3$) δ 7.65 (d, J = 8.0 Hz, 1H), 7.52 – 7.39 (m, 5H), 7.35 (d, J = 8.1 Hz, 1H), 7.31 – 7.20 (m, 5H), 7.21 – 7.14 (m, 1H), 7.09-7.06 (m, 1H), 6.55 (dd, J = 15.9, 6.6 Hz, 1H), 6.34-6.30 (m, 2H), 5.79 (s, 1H), 4.89 (dd, J = 6.6, 1.4 Hz, 1H), 3.60 (s, 3H), 3.59 (s, 3H) ppm. **^{13}C NMR** (126 MHz, $CDCl_3$) δ 167.6, 142.8, 139.1, 137.7 137.5 132.0, 130.9 130.8, 128.5, 128.5 128.5 127.2, 127.0, 126.4, 125.6, 121.5, 120.7, 119.4, 110.8, 109.6, 51.9, 41.9, 31.0 ppm. **Specific rotation** $[\alpha]_D^{23}$ = +27 (c = 0.7, CH_2Cl_2). **HRMS** (ESI) m/z calculated for $C_{28}H_{25}NO_2$ $[M+H]^+$, 408.1958 found: 408.1955. **IR (neat)** 2970, 1738, 1435, 1217, 900, 750, 527 cm^{-1}. **HPLC conditions**: Chiral column IA, hexane: isopropanol = 98:2, flow rate = 1 mL/min, wavelength = 254 nm, tR = 7.9 min for major isomer, tR = 12.4 min for minor isomer.

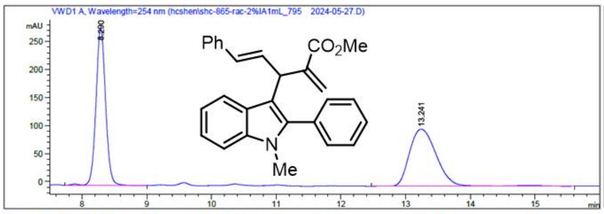

Peak #	RetTime [min]	Type	Width [min]	Area [mAU*s]	Height [mAU]	Area %
1	8.290	VB R	0.1604	2892.19507	280.23953	49.3394
2	13.241	VV R	0.4603	2969.64453	101.91763	50.6606

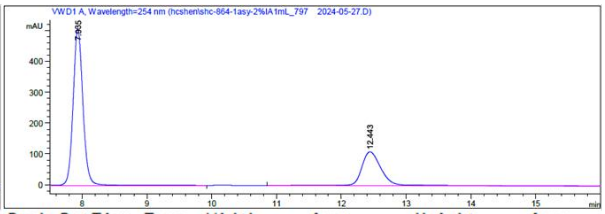

Peak #	RetTime [min]	Type	Width [min]	Area [mAU*s]	Height [mAU]	Area %
1	7.935	VV R	0.1594	5426.60791	508.62485	67.9814
2	12.443	VV R	0.3195	2555.88232	109.60107	32.0186

Linear Product 5ee': **^{1}H NMR** (500 MHz, $CDCl_3$) δ 7.66 (dt, J = 7.9, 1H), 7.48 – 7.39 (m, 4H), 7.38-7.33 (m, 1H), 7.33 – 7.24 (m, 4H), 7.28 – 7.18 (m, 4H), 7.10 (dd, J = 8.1, 6.9, 1H), 6.86 (dd, J = 15.4, 11.4 Hz, 1H), 6.72 (d, J = 15.4 Hz, 1H), 3.98 (s, 2H), 3.63 (s, 3H), 3.53 (s, 3H) ppm. **^{13}C NMR** (126 MHz, $CDCl_3$) δ 168.8, 139.5, 139.0, 138.5, 137.1, 136.7, 132.2, 131.1, 130.9, 128.7, 128.7, 128.5, 128.3, 127.7, 127.4, 124.1, 121.7, 119.8, 119.5, 110.7, 109.4, 51.8, 30.9, 23.2 ppm.

methyl (*R*)-3-(1-methyl-2-phenyl-1H-indol-3-yl)-2-methylene-5-phenylpentanoate (5ff)

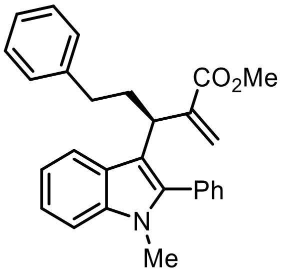

Prepared following **General procedure B**. Purification by flash column chromatography (20% DCM in toluene) gave the title compound (19.2 mg, 24%, 79:21 er, 22:78 rr).

^{1}H NMR (500 MHz, CDCl3) δ 7.70 (d, J = 8.0 Hz, 1H), 7.49 – 7.39 (m, 3H), 7.36-7.35 (m, 2H), 7.27 – 7.21 (m, 1H), 7.18-7.14 (m, 2H), 7.15 – 7.06 (m, 2H), 6.96 (d, J = 7.4 Hz, 2H), 6.21 (s, 0H), 5.68 (s, 1H), 4.05 (dd, J = 9.7, 5.0 Hz, 1H), 3.61 (s, 3H), 3.56 (s, 3H), 2.47 – 2.23 (m, 3H), 2.24 – 2.14 (m, 1H) ppm. **^{13}C NMR** (126 MHz, CDCl3) δ 168.1, 143.9, 142.6, 139.3, 137.6, 132.2, 131.0, 128.5, 128.4, 128.4, 128.3, 126.8, 125.7, 124.2, 121.5, 120.6, 119.3, 112.0, 109.6, 51.8, 38.4, 35.4, 34.6, 31.0 ppm. **Specific rotation** $[\alpha]_D^{23}$ = -5 (c = 0.4, CH_2Cl_2). **HRMS** (ESI) m/z calculated for C28H27NO2 $[M+H]^+$, 410.2115 found: 410.2112. **IR** (neat) 2945, 1720, 1631, 1467, 1273, 1137, 742, 702cm-1. **HPLC conditions**: Chiral column IA, hexane:

isopropanol = 99:1, flow rate = 1 mL/min, wavelength = 254 nm, tR = 9.1 min for major isomer, tR = 7.2 min for minor isomer.

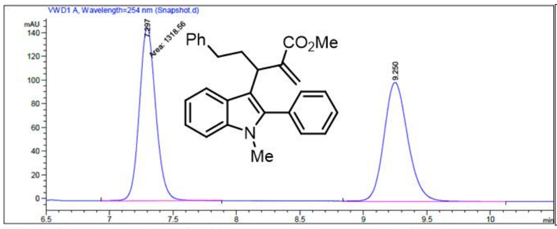

Peak #	RetTime [min]	Type	Width [min]	Area [mAU*s]	Height [mAU]	Area %
1	7.297	MM	0.1496	1318.56140	146.85603	49.9062
2	9.250	VB	0.2063	1323.51660	100.37457	50.0938

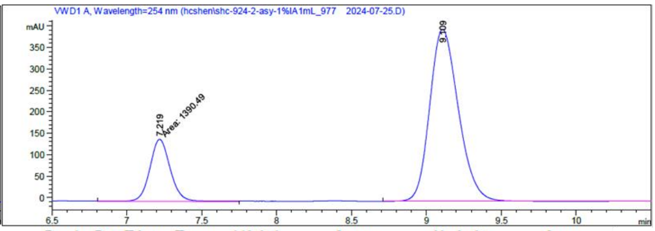

Peak #	RetTime [min]	Type	Width [min]	Area [mAU*s]	Height [mAU]	Area %
1	7.219	MM	0.1592	1390.48523	145.58369	20.6096
2	9.109	BB	0.2086	5356.31104	400.07648	79.3904

Linear Product 5ff': **^{1}H NMR** (500 MHz, CDCl3) δ 7.58 (d, J = 7.8 Hz, 1H), 7.47-7.39 (m, 3H), 7.37 – 7.29 (m, 3H), 7.25-7.22 (m, 3H), 7.22 – 7.15 (m, 1H), 7.12-7.09 (m, 1H), 6.96 (d, J = 7.0 Hz, 1H), 6.71 (t, J = 7.4 Hz, 1H), 3.76 (s, 2H), 3.62 (s, 3H), 3.53 (s, 2H), 2.56 – 2.46 (m, 2H), 2.25 (q, J = 7.7 Hz, 2H) ppm. **^{13}C NMR** (126 MHz, CDCl3) δ 168.8, 142.2, 141.3, 138.3, 137.1, 132.3, 132.0, 131.1, 128.4, 128.4, 128.2, 127.6, 126.1, 121.6, 119.7, 119.3, 110.4, 109.34, 51.8, 34.7, 30.9, 30.5, 22.7 ppm.

methyl 2-((*S*)-(4-chlorophenyl)((2*R*,3*R*)-1-(4-methoxybenzyl)-2-phenyl-2-(4,4,5,5-tetramethyl-1,3,2-dioxaborolan-2-yl)indolin-3-yl)methyl)acrylate (6)

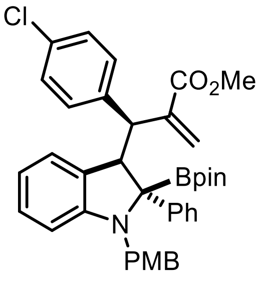

Prepared following **General procedure B**, without oxidation. Purification by flash column chromatography (10% EtOAc in *n*-hexane) gave the title compound (95.6 mg, 74%, >95:5 rr, >95:5 dr).

^{1}H NMR (400 MHz, CD_2Cl_2) δ 7.30 (d, J = 7.7 Hz, 1H), 7.25-7.22 (m, 2H), 7.18-7.13 (m, 3H), 7.01 – 6.87 (m, 5H), 6.79 (d, J = 8.7 Hz, 2H), 6.41 – 6.31 (m, 2H), 6.11 (dd, J = 14.7, 7.6 Hz,

2H), 5.62 (s, 1H), 5.00 (d, J = 8.4 Hz, 1H), 4.44 (d, J = 16.5 Hz, 1H), 4.28 (d, J = 16.6 Hz, 1H), 3.92 (d, J = 8.4 Hz, 1H), 3.80 (s, 3H), 3.64 (s, 3H), 1.12 (s, 6H), 1.09 (s, 3H). ppm. **^{13}C NMR** (126 MHz, CDCl3) δ 167.0, 158.3, 152.3, 145.7, 143.2, 139.5, 132.4, 131.8, 131.7, 128.2, 128.2, 128.1, 127.9, 127.5, 126.4, 126.1, 126.1, 116.5, 113.5, 105.9, 84.2, 57.6, 55.4, 51.9, 51.0, 48.4, 25.1, 24.8 ppm. **HRMS** (ESI) m/z calculated for $C_{39}H_{42}BClNO_5$ $[M+H]^+$, 650.2839, found: 650.2817. **IR (neat)** 2980, 1721, 1600, 1510, 1485, 1244, 1110, 852, 732 cm^{-1}.

methyl 2-((*S*)-((2*S*,3*S*)-1-methyl-2-phenylindolin-3-yl)(phenyl)methyl)acrylate (7)

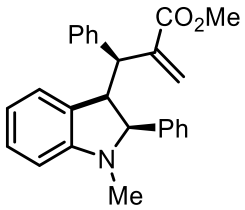

Prepared following **General procedure D**. Purification by flash column chromatography (10% Et_2O in *n*-hexane) gave the title compound (40.8 mg, 63%, 95:5 er, > 95:5 dr, >95:5 rr).

^{1}H NMR (500 MHz, CD_2Cl_2) δ 7.32 – 7.20 (m, 6H), 7.20 – 7.12 (m, 4H), 7.06 – 7.00 (m, 1H), 6.40 – 6.35 (m, 1H), 6.34 (s, 1H), 6.30 (td, *J* = 7.4, 1.0 Hz, 1H), 5.96 (d, *J* = 7.3 Hz, 1H), 5.67 (s, 1H), 4.27 (d, *J* = 4.8 Hz, 1H), 4.11 (d, *J* = 10.7 Hz, 1H), 3.94 (dd, *J* = 10.7, 4.8 Hz, 1H), 3.57 (s, 3H), 2.56 (s, 3H) ppm. **^{13}C NMR** (126 MHz, CD_2Cl_2) δ 167.2, 152.8, 142.5, 142.5, 141.4, 129.9, 129.9, 128.8, 128.5, 128.5, 128.0, 127.7, 127.2, 126.3, 125.9, 116.5, 105.6, 75.1, 53.0, 52.4, 52.1, 32.6 ppm. **Specific rotation** $[\alpha]_D^{23}$ = +157 (c = 1.0, CH_2Cl_2). **HRMS** (ESI) m/z calculated for $C_{26}H_{25}NO_2$ $[M+H]^+$, 384.1958 found: 384.1940. **IR (neat)** 3027, 2948, 1721, 1602, 1487, 1373, 1216, 1138, 748, 700 cm^{-1}. **HPLC conditions**: Chiral column IA, hexane: isopropanol = 97:3, flow rate = 1 mL/min, wavelength = 254 nm, tR = 6.5 min for major isomer, tR = 4.7 min for minor isomer.

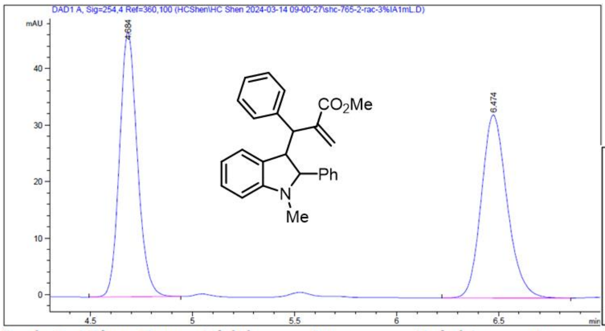

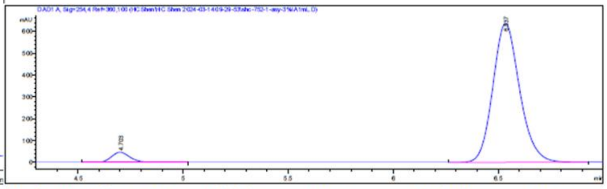

Peak #	RetTime [min]	Type	Width [min]	Area [mAU*s]	Height [mAU]	Area %
1	4.684	BB	0.0949	284.80066	46.88788	49.9153
2	6.474	BB	0.1348	285.76746	32.43019	50.0847

Peak #	RetTime [min]	Type	Width [min]	Area [mAU*s]	Height [mAU]	Area %
1	4.703	BB	0.0940	289.28418	46.88819	4.8778
2	6.537	BB	0.1374	5641.29053	636.49084	95.1222

methyl (*R*)-2-((4-chlorophenyl)(2-phenyl-1H-indol-3-yl)methyl)acrylate (8)

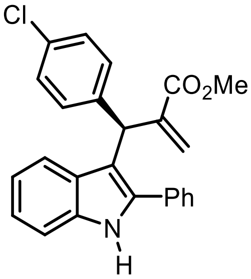

Prepared following **Procedure G**. Purification by flash column chromatography (10% EtOAc in *n*-hexane) gave the title compound (38.7 mg, 84%, 93.4:6.6 er).

^{1}H NMR (500 MHz, CD_2Cl_2) δ 8.17 (s, 1H), 7.49 – 7.32 (m, 7H), 7.18 (qd, J = 8.4, 6.1 Hz, 5H), 7.00 (ddd, J = 8.0, 7.0, 1.0 Hz, 1H), 6.49 (s, 1H), 5.67 (s, 1H), 5.61 (s, 1H), 3.61 (s, 3H) ppm. **^{13}C NMR** (126 MHz, CDCl3) δ 167.6, 142.8, 141.0, 136.2, 132.8, 132.1, 130.1, 128.9, 128.6, 128.5, 128.3, 128.2, 127.8, 122.2, 120.9, 120.0, 111.7, 111.1, 52.1, 43.6 ppm. **Specific rotation** $[\alpha]_D^{23}$ = +85 (c = 0.8, CH_2Cl_2). **HRMS** (ESI) m/z calculated for $C_{25}H_{21}ClNO_2$ $[M+H]^+$, 402.1255 found: 402.1263. **IR (neat)** 3375, 2950, 1711, 1628, 1489, 1253, 1140, 1014, 743 cm^{-1}. **HPLC conditions**: Chiral column IA, hexane: isopropanol = 90:10, flow rate = 1 mL/min, wavelength = 254 nm, tR = 29.8 min for major isomer, tR = 23.1 min for minor isomer.

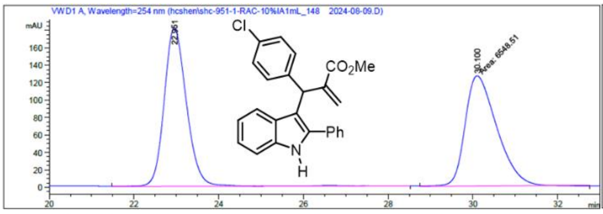

Peak #	RetTime [min]	Type	Width [min]	Area [mAU*s]	Height [mAU]	Area %
1	22.951	VV R	0.5584	6665.93994	181.51311	50.4443
2	30.100	MM	0.8647	6548.51465	126.21425	49.5557

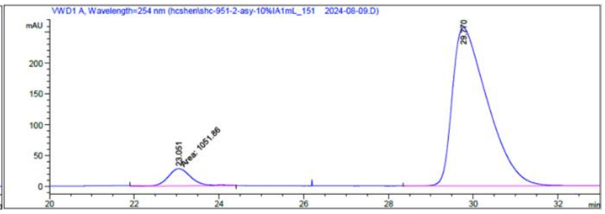

Peak #	RetTime [min]	Type	Width [min]	Area [mAU*s]	Height [mAU]	Area %
1	23.051	MM	0.6265	1051.86316	27.98441	6.6004
2	29.770	BV	0.8686	1.48845e4	256.74548	93.3996

methyl 2-((1-methyl-1H-pyrrol-2-yl)(phenyl)methyl)acrylate (12)

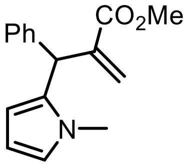

Prepared following **General procedure B**. Purification by flash column chromatography (10% Et_2O in *n*-hexane) gave the title compound (20.5 mg, 40%, 60:40 rr).

^{1}H NMR (400 MHz, $CDCl_3$) δ 7.34 – 7.25 (m, 2H), 7.27 – 7.18 (m, 1H), 7.18 – 7.10 (m, 2H), 6.59 (dd, J = 2.7, 1.8 Hz, 1H), 6.37 (t, J = 1.0 Hz, 1H), 6.02 (dd, J = 3.6, 2.7 Hz, 1H), 5.68 (ddd, J = 3.6, 1.8, 0.8 Hz, 1H), 5.32 (s, 1H), 5.20 (t, J = 1.3 Hz, 1H), 3.71 (s, 3H), 3.42 (s, 3H) ppm. **^{13}C NMR** (126 MHz, CD_2Cl_2) 167.4, 142.6, 140.5, 132.7, 129.0, 128.6, 127.1, 127.0, 122.5, 108.9, 106.6, 52.2, 44.7, 33.8 ppm **HRMS** (ESI) m/z calculated for $C_{16}H_{17}NO_2$ $[M+H]^+$, 256.1332 found: 256.1328. **IR (neat)** 2970, 1726, 1628, 1436, 1366, 1229, 1134, 705 cm^{-1}.

2.6. Crystallography

X-ray diffraction experiments on **5r** and **5s** were carried out at 100(2) K on a Bruker D8 Venture diffractometer using Cu-K_α (λ = 1.54178 Å) (**5r**) or Mo-K_α radiation (λ = 0.71073 Å) (**5s**). Intensities were integrated in SAINT[5] and absorption corrections based on equivalent reflections were applied using SADABS.[6] The structures were solved using ShelXT[7] and refined by full matrix least squares against F^2 in ShelXL[8, 9] using Olex2[10]. All of the non-hydrogen atoms were refined anisotropically. While all of the hydrogen atoms were located geometrically and refined using a riding model. In **5s** there was a partially occupied water the occupancy of which was refined freely and came out at ~8%. *The absolute structure of **5r** (Flack 0.07(8)) was not able to be fully determined by crystallographic methods.* Crystal structure and refinement data are given in Table 1. Crystallographic data for compounds **5r** and **5s** have been deposited with the Cambridge Crystallographic Data Centre as supplementary publication CCDC 2359404-2359405. Copies of the data can be obtained free of charge on application to CCDC, 12 Union Road, Cambridge CB2 1EZ, UK [fax(+44) 1223 336033, e-mail: deposit@ccdc.cam.ac.uk].

Table S8 Crystal data and structure refinement for **5r** and **5s**.

Identification code	**5r**	**5s**
Empirical formula	$C_{28}H_{25}NO_4$	$C_{28}H_{24.16}BrNO_{4.08}$
Formula weight	439.49	519.92
Temperature/K	100(2)	100(2)
Crystal system	orthorhombic	monoclinic
Space group	$P2_12_12_1$	$P2_1$
a/Å	9.65730(10)	7.9025(2)
b/Å	9.87800(10)	20.6123(6)

c/Å	23.6851(3)	8.1821(2)
α/°	90	90
β/°	90	116.5580(10)
γ/°	90	90
Volume/Å^3	2259.44(4)	1192.14(6)
Z	4	2
ρ_{calc}g/cm^3	1.292	1.448
μ/mm^{-1}	0.694	1.760
F(000)	928.0	534.0
Crystal size/mm^3	0.336 × 0.24 × 0.14	0.38 × 0.36 × 0.186
Radiation	CuKα (λ = 1.54178)	MoKα (λ = 0.71073)
2θ range for data collection/°	7.464 to 136.402	3.952 to 55.946
Index ranges	$-11 \le h \le 11$, $-11 \le k \le 11$, $-28 \le l \le 28$	$-10 \le h \le 10$, $-27 \le k \le 27$, $-10 \le l \le 10$
Reflections collected	38511	40093
Independent reflections	4123 [R_{int} = 0.0456, R_{sigma} = 0.0271]	5694 [R_{int} = 0.0495, R_{sigma} = 0.0361]
Data/restraints/parameters	4123/0/301	5694/1/320
Goodness-of-fit on F^2	1.075	1.039
Final R indexes [I>=2σ (I)]	R_1 = 0.0319, wR_2 = 0.0860	R_1 = 0.0321, wR_2 = 0.0750
Final R indexes [all data]	R_1 = 0.0321, wR_2 = 0.0862	R_1 = 0.0349, wR_2 = 0.0765
Largest diff. peak/hole / e Å^{-3}	0.18/-0.24	0.25/-0.26
Flack parameter	0.07(8)	-0.011(7)

3. REFERENCES

[1] A. F. Burchat, J. M. Chong, N. Nielsen, Titration of alkyllithiums with a simple reagent to a blue endpoint. *J. Organomet. Chem.* **1997**, *542*, 281−283.

[2] G. Zhu, J. Yang, G. Bao, M. Zhang, J. Li, Y. Li, W. Sun, L. Hong, R. Wang, Catalyst-controlled switch of regioselectivity in the asymmetric allylic alkylation of oxazolones with MBHCs. *Chem. Commun.* **2016**, *52*, 7882--7885.

[3] V. K. Aggarwal, I. Emme, S. Y. Fulford, Correlation between pK(a) and reactivity of quinuclidine-based catalysts in the Baylis-Hillman reaction: discovery of quinuclidine as optimum catalyst leading to substantial enhancement of scope. *J. Org. Chem.* **2003**, *68*, 692-700.

[4] S. Panda, J. M. Ready. Tandem allylation/1,2-boronate rearrangement for the asymmetric synthesis of indolines with adjacent quaternary stereocenters. *J. Am. Chem. Soc.* **2018**, *140*, 13242–13252.

[5] Bruker, SAINT+ v8.38A Integration Engine, Data Reduction Software, Bruker Analytical X-ray Instruments Inc., Madison, WI, USA **2015**.

[6] Bruker, SADABS 2014/5, Bruker AXS area detector scaling and absorption correction, Bruker Analytical X-ray Instruments Inc., Madison, Wisconsin, USA **2014/5**.

[7] G. M. Sheldrick, A short history of SHELX. *Acta Crystallogr.* **2008**, *64*, 112–122.

[8] G. M. Sheldrick, Crystal structure refinement with SHELXL. *Acta Crystallogr. C Struct. Chem.* **2015**, *71*, 3–8.

[9] O. V. Dolomanov, L. J. Bourhis, R. J. Gildea, J. A. K. Howard, H. Puschmann, OLEX2: a complete structure solution, refinement and analysis program. *J. Appl. Crystallogr.* **2009**, *42*, 339–341.

4. NMR SPECTRA

Compound **5a**

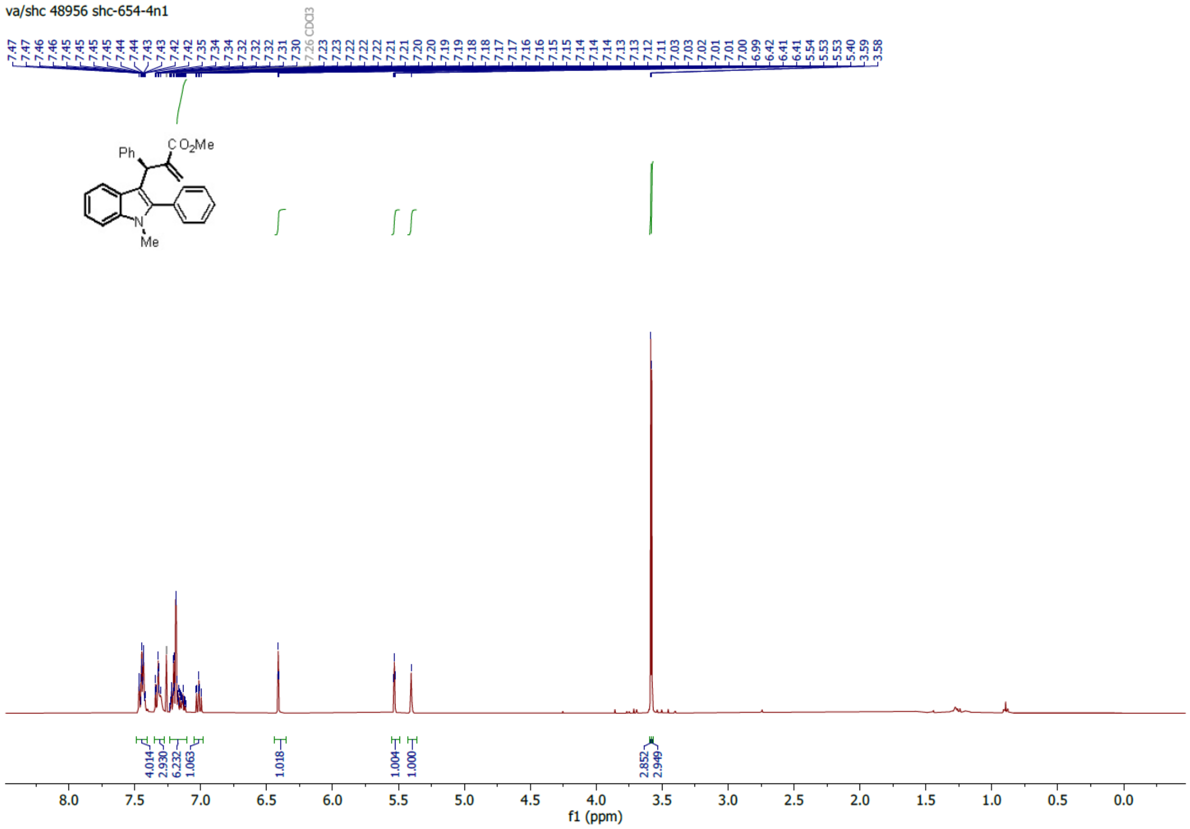

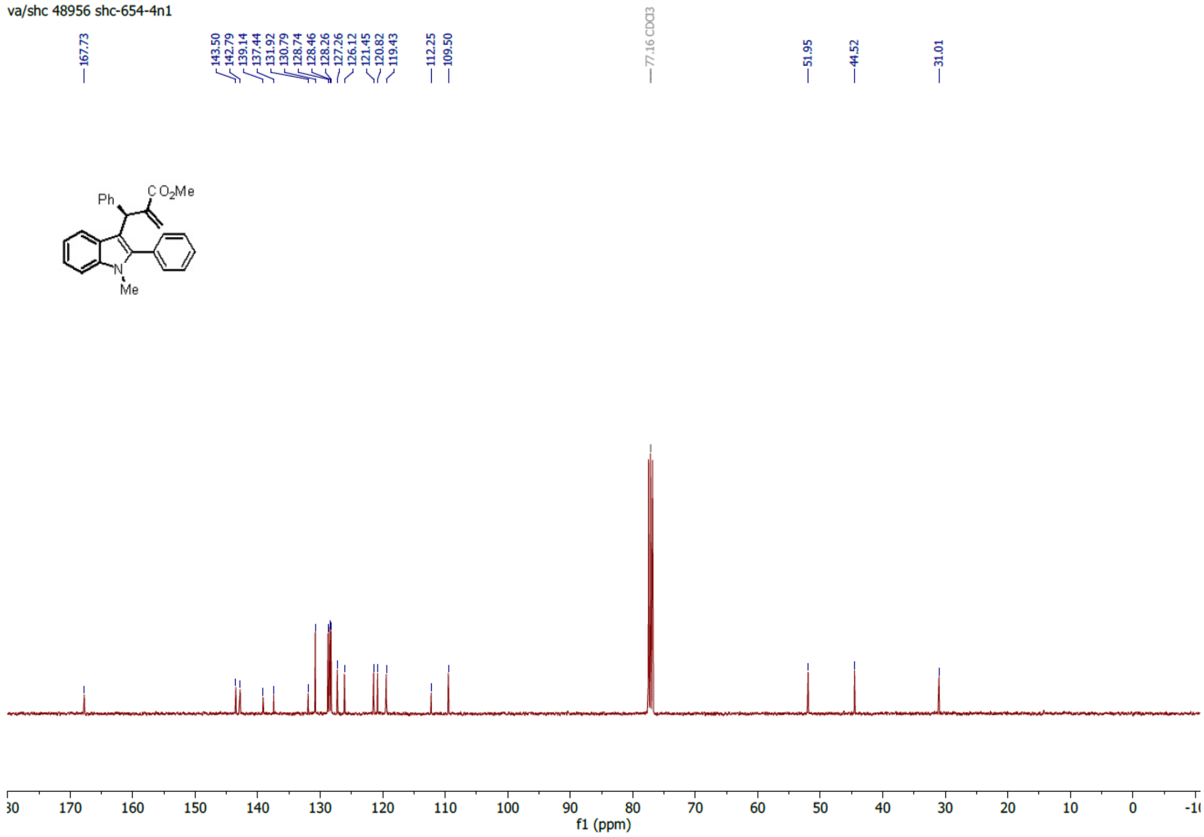

Compound **4**

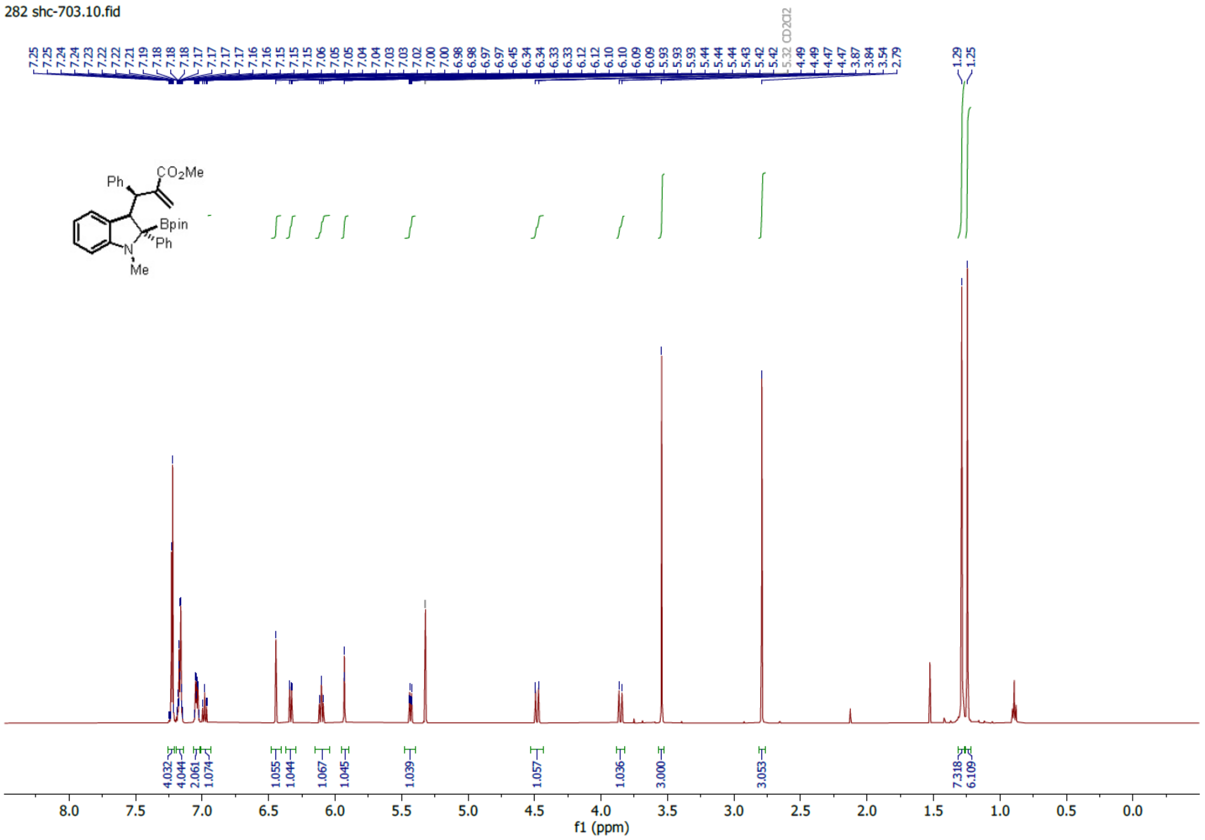

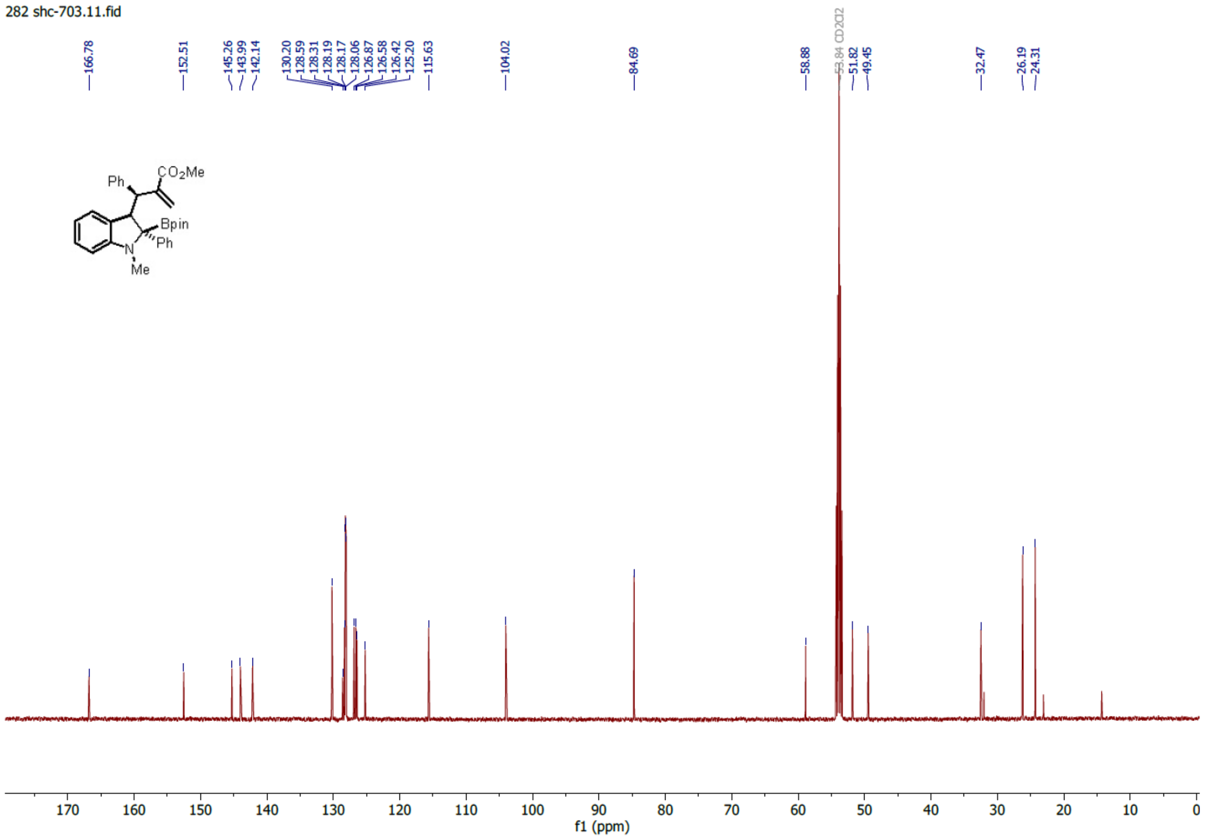

Compound **4** HSQC

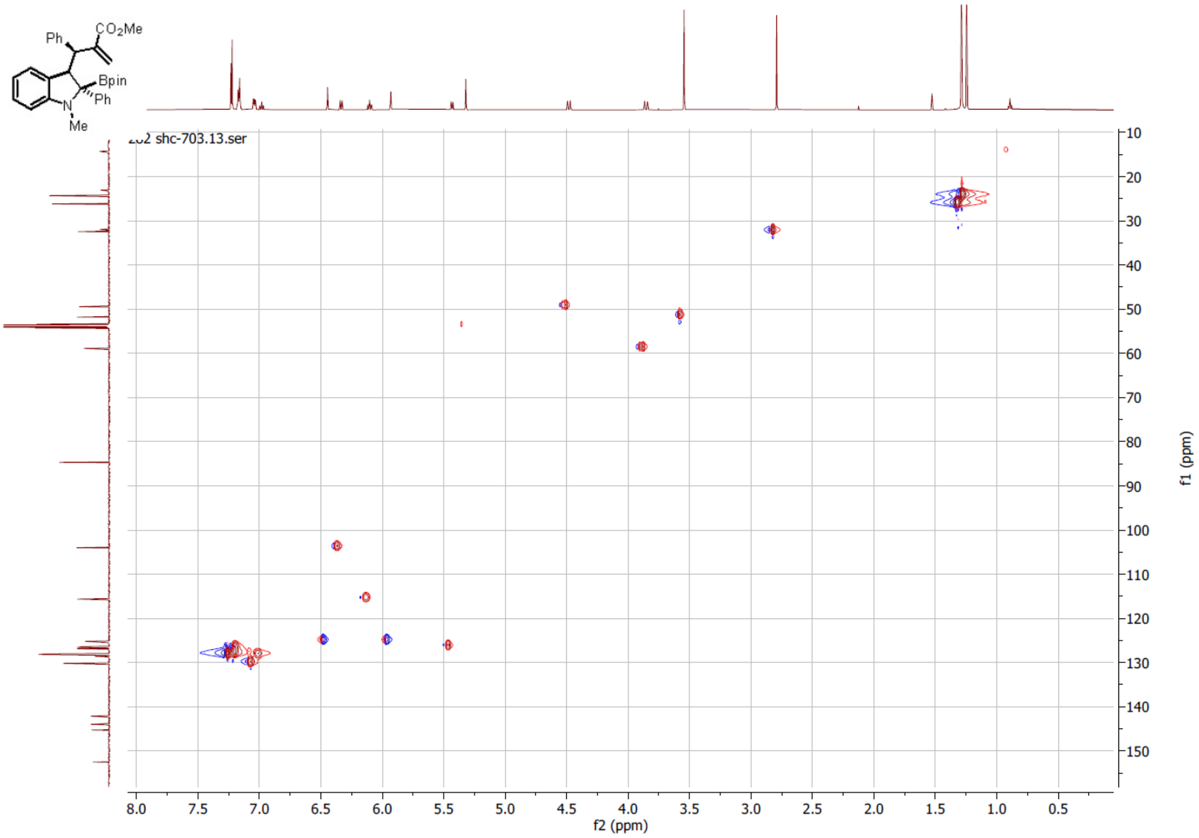
Ph
CO2Me
Bpin
Ph
N
Me
shc-703.13.ser
f2 (ppm)
f1 (ppm)

Compound **5’**

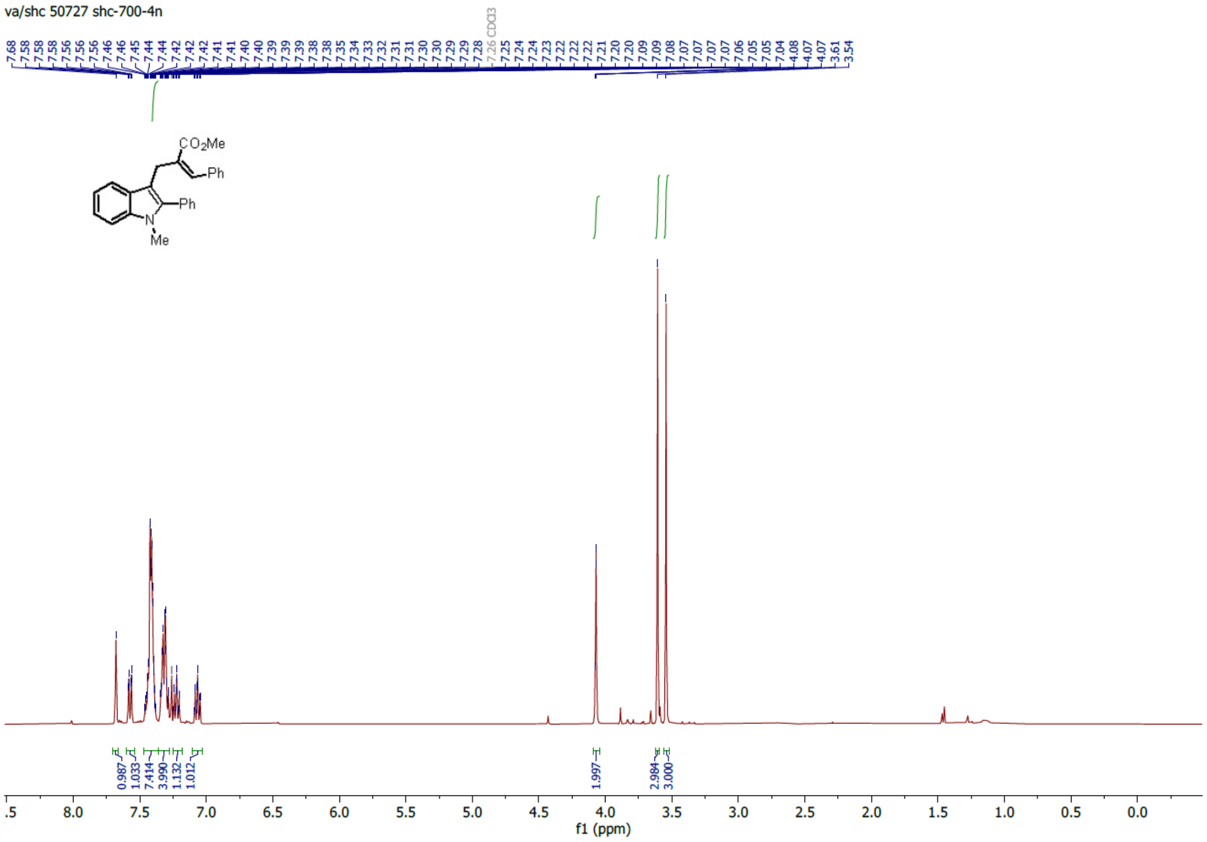

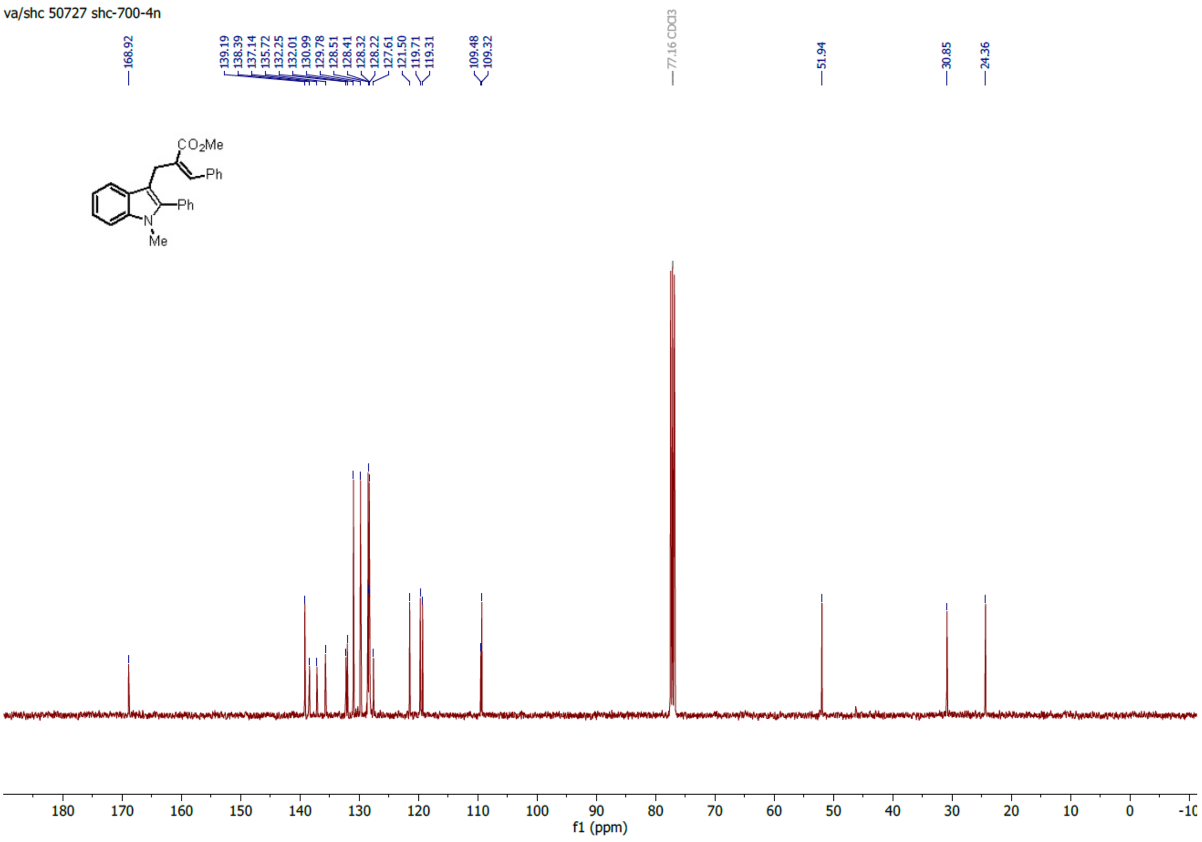

Compound **5b**

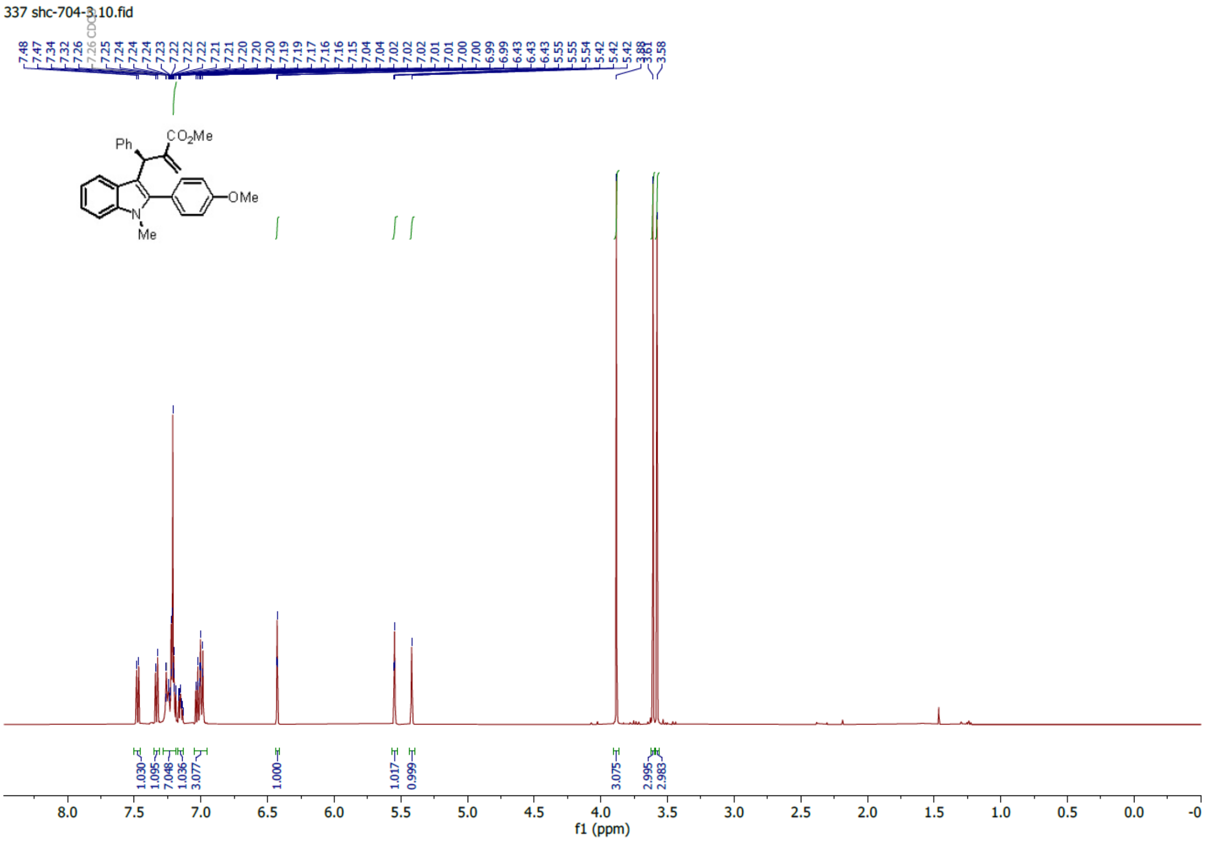

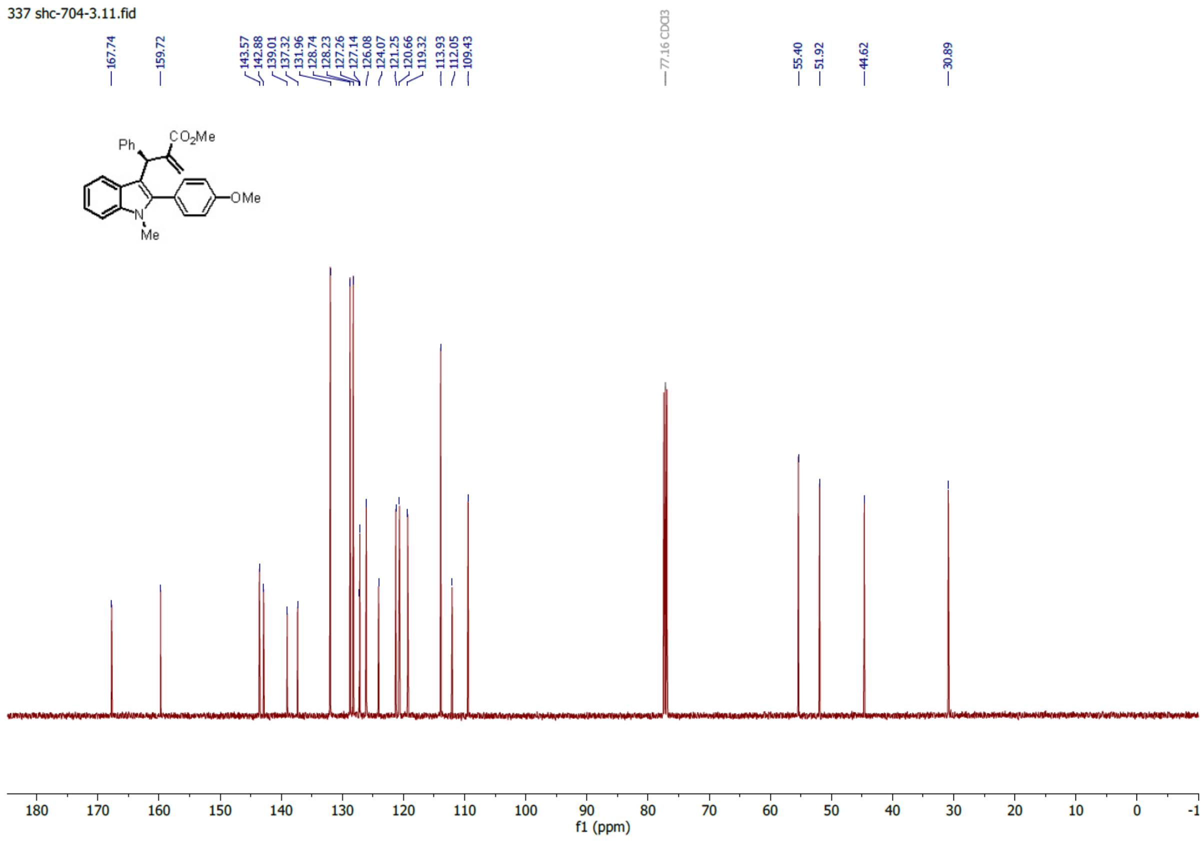

Compound **5c**

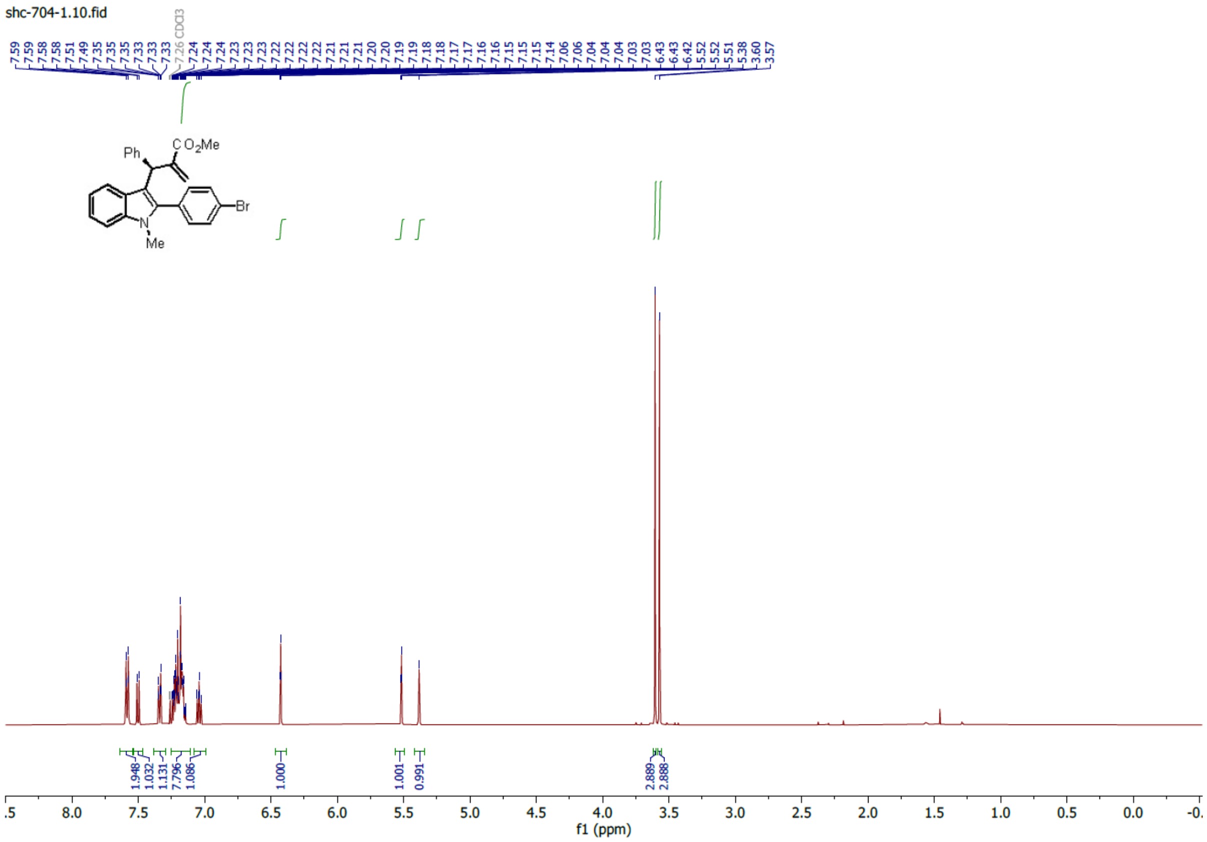
shc-704-1.10.fid
7.59
7.59
7.58
7.58
7.51
7.49
7.35
7.35
7.35
7.33
7.33
7.33
7.26 CDCl3
7.24
7.24
7.24
7.23
7.23
7.23
7.22
7.22
7.22
7.22
7.21
7.21
7.21
7.20
7.20
7.19
7.19
7.18
7.18
7.17
7.17
7.16
7.16
7.15
7.15
7.15
7.14
7.06
7.06
7.04
7.04
7.03
7.03
6.43
6.43
6.42
5.52
5.52
5.51
5.38
3.60
3.57
Ph
CO2Me
Br
N
Me
1.948
1.032
1.131
7.796
1.086
1.000
1.001
0.991
2.889
2.888
f1 (ppm)

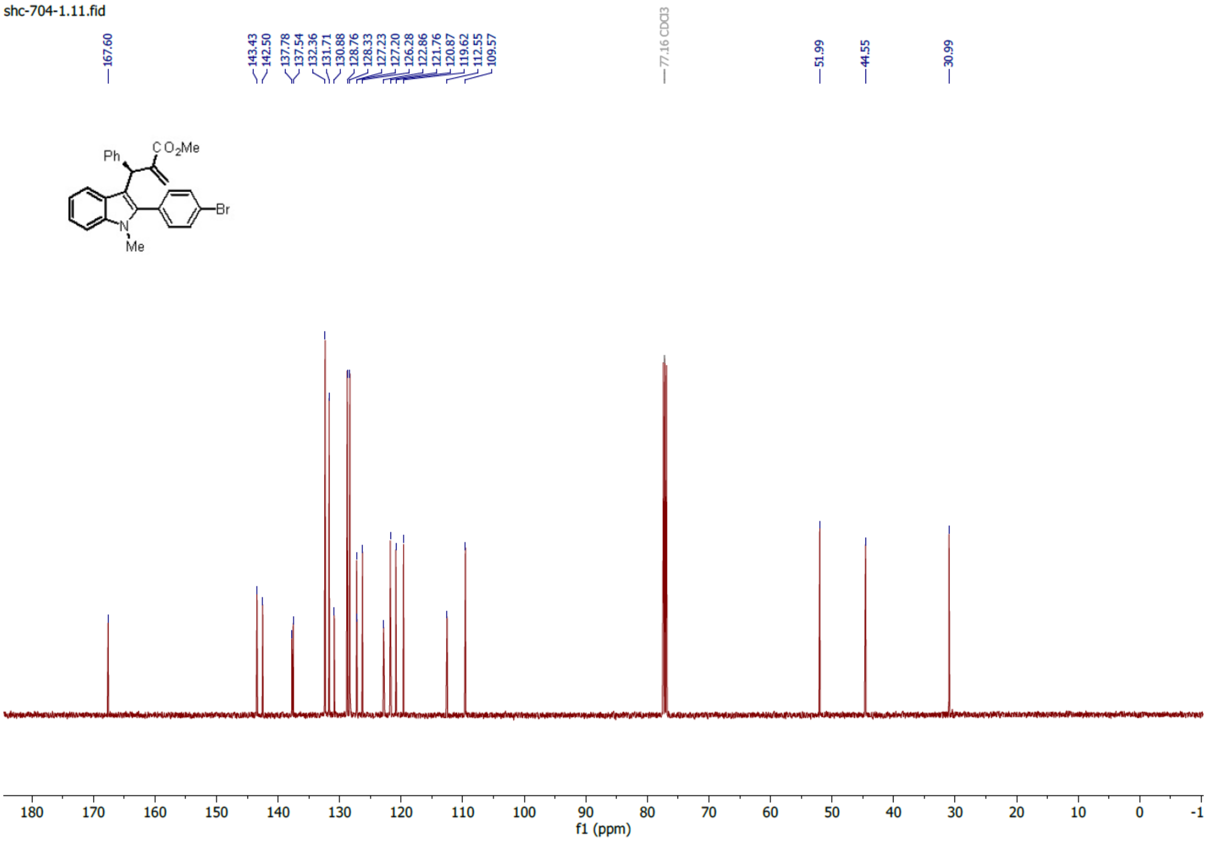
shc-704-1.11.fid
167.60
143.43
142.50
137.78
137.54
132.36
131.71
130.88
128.76
128.33
127.23
127.20
126.28
122.86
121.76
120.87
119.62
112.55
109.57
77.16 CDCl3
51.99
44.55
30.99
Ph
CO2Me
Br
N
Me
f1 (ppm)

Compound **5d**

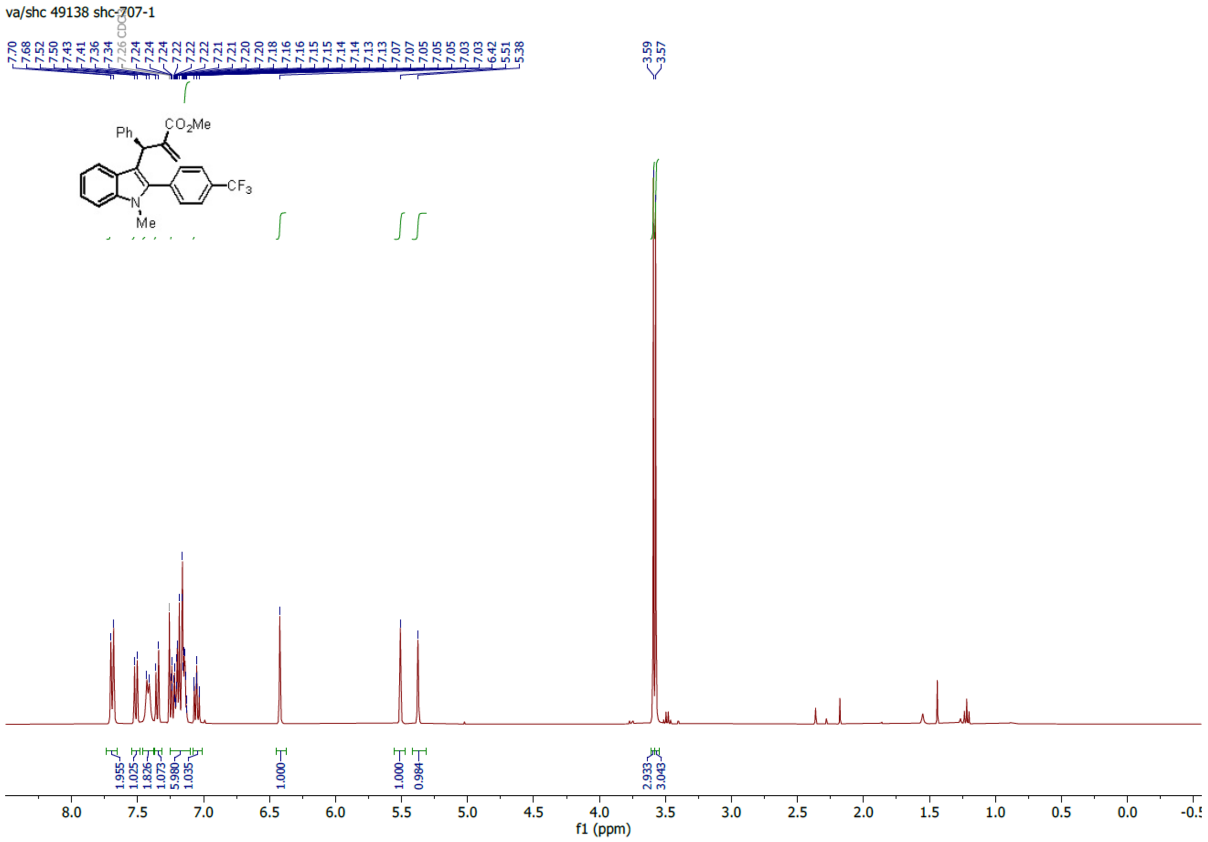

va/shc 49138 shc-707-1
7.70 7.68 7.52 7.50 7.43 7.41 7.36 7.34 7.26 CDCl3 7.24 7.24 7.24 7.22 7.22 7.22 7.21 7.21 7.20 7.20 7.18 7.16 7.16 7.15 7.15 7.14 7.14 7.13 7.13 7.07 7.07 7.05 7.05 7.05 7.03 7.03 6.42 5.51 5.38
3.59 3.57
Ph
CO2Me
CF3
N
Me
1.955 1.025 1.826 1.073 5.980 1.035 1.000 1.000 0.984 2.933 3.043
f1 (ppm)

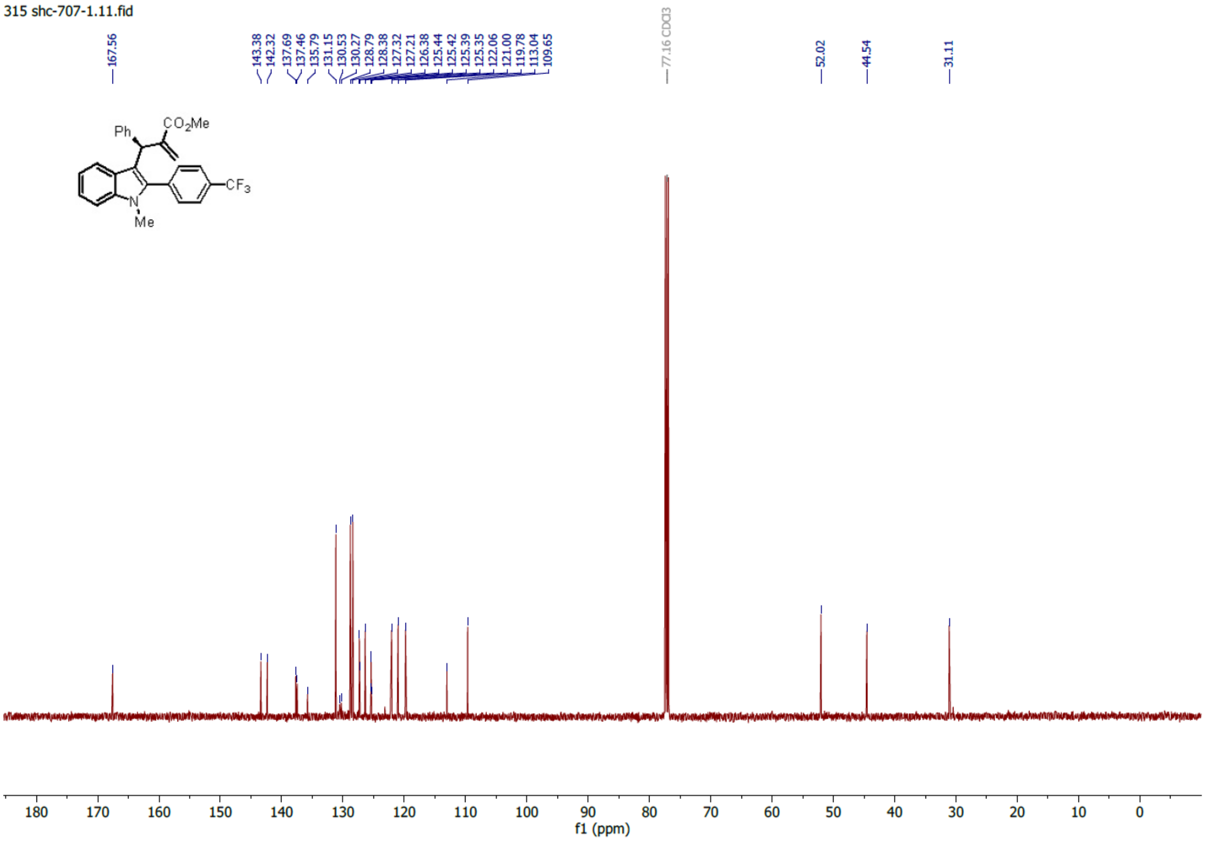

315 shc-707-1.11.fid
167.56
143.38 142.32 137.69 137.46 135.79 131.15 130.53 130.27 128.79 128.38 127.32 127.21 126.38 125.44 125.42 125.39 125.35 122.06 121.00 119.78 113.04 109.65
77.16 CDCl3
52.02
44.54
31.11
Ph
CO2Me
CF3
N
Me
f1 (ppm)

Compound **5e**

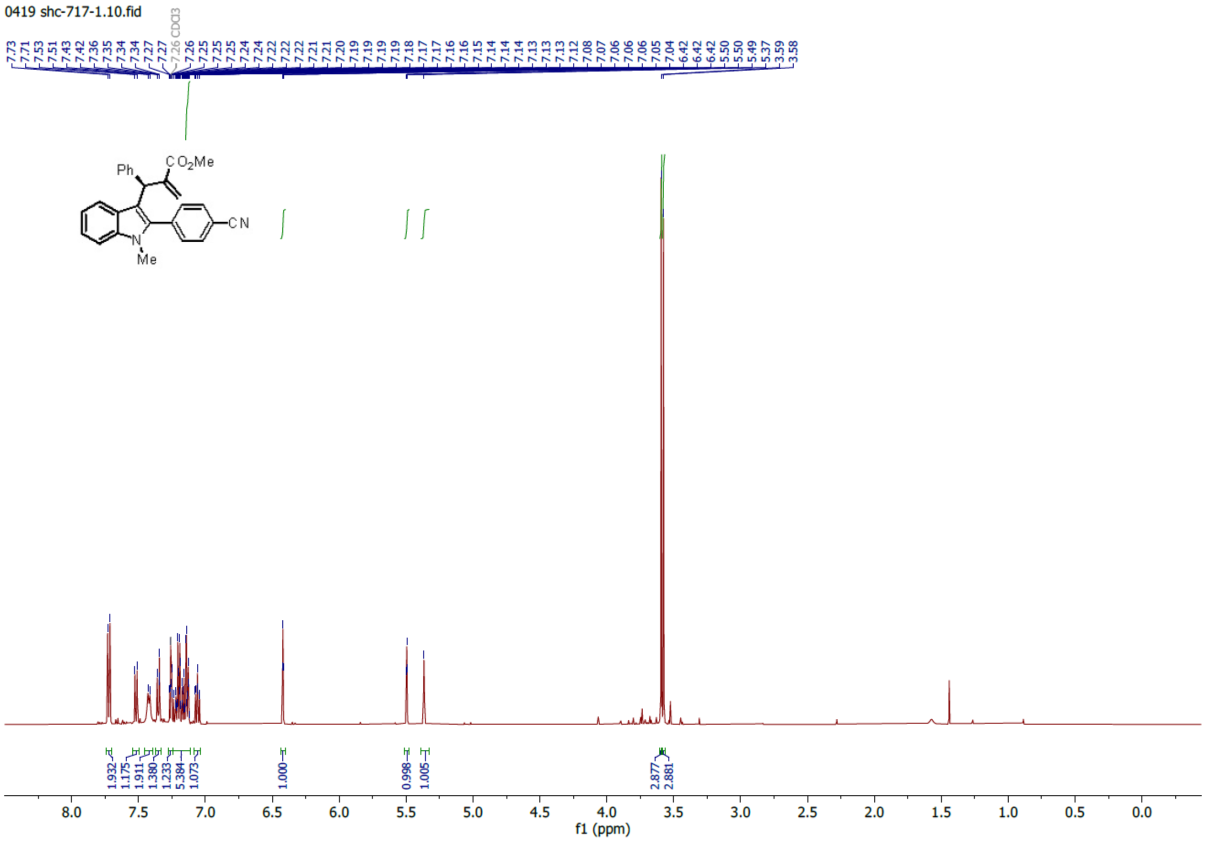
0419 shc-717-1.10.fid
CO2Me
Ph
CN
N
Me
f1 (ppm)

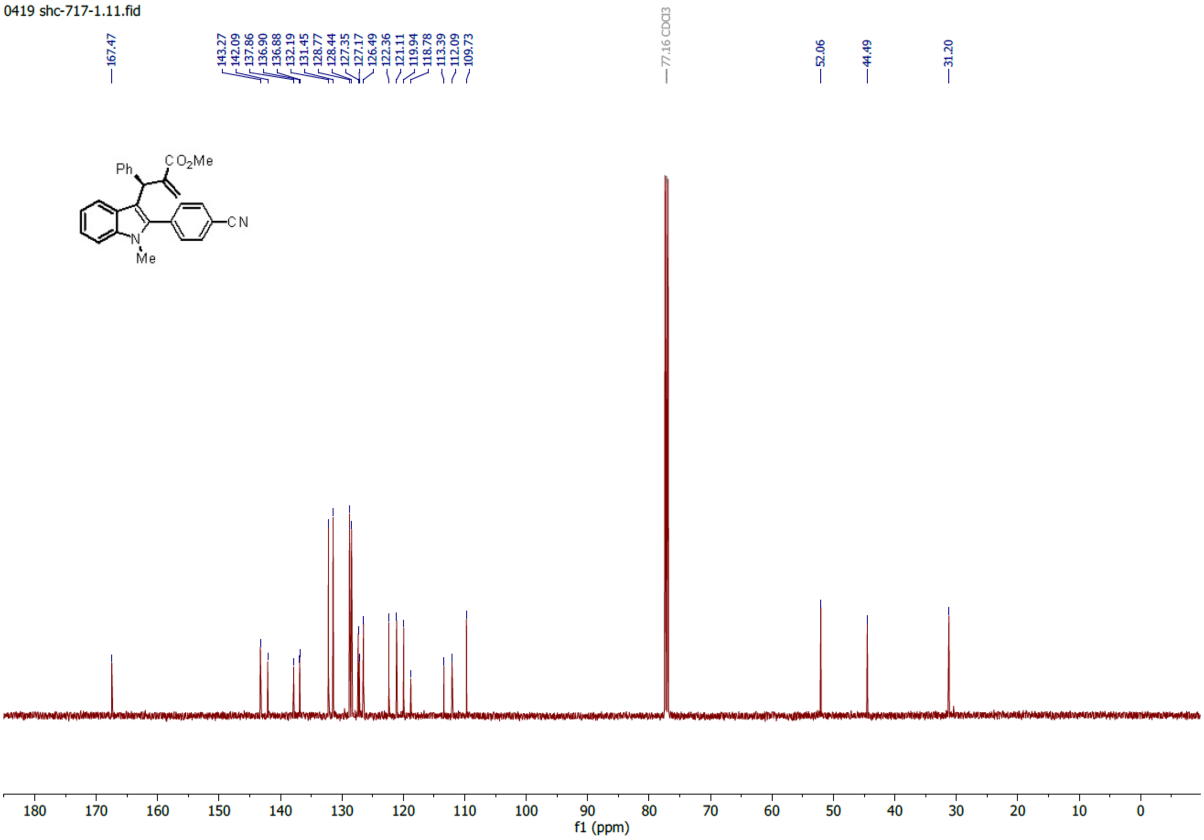
0419 shc-717-1.11.fid
167.47
77.16 CDCl3
52.06
44.49
31.20
CO2Me
Ph
CN
N
Me
f1 (ppm)

Compound **5f**

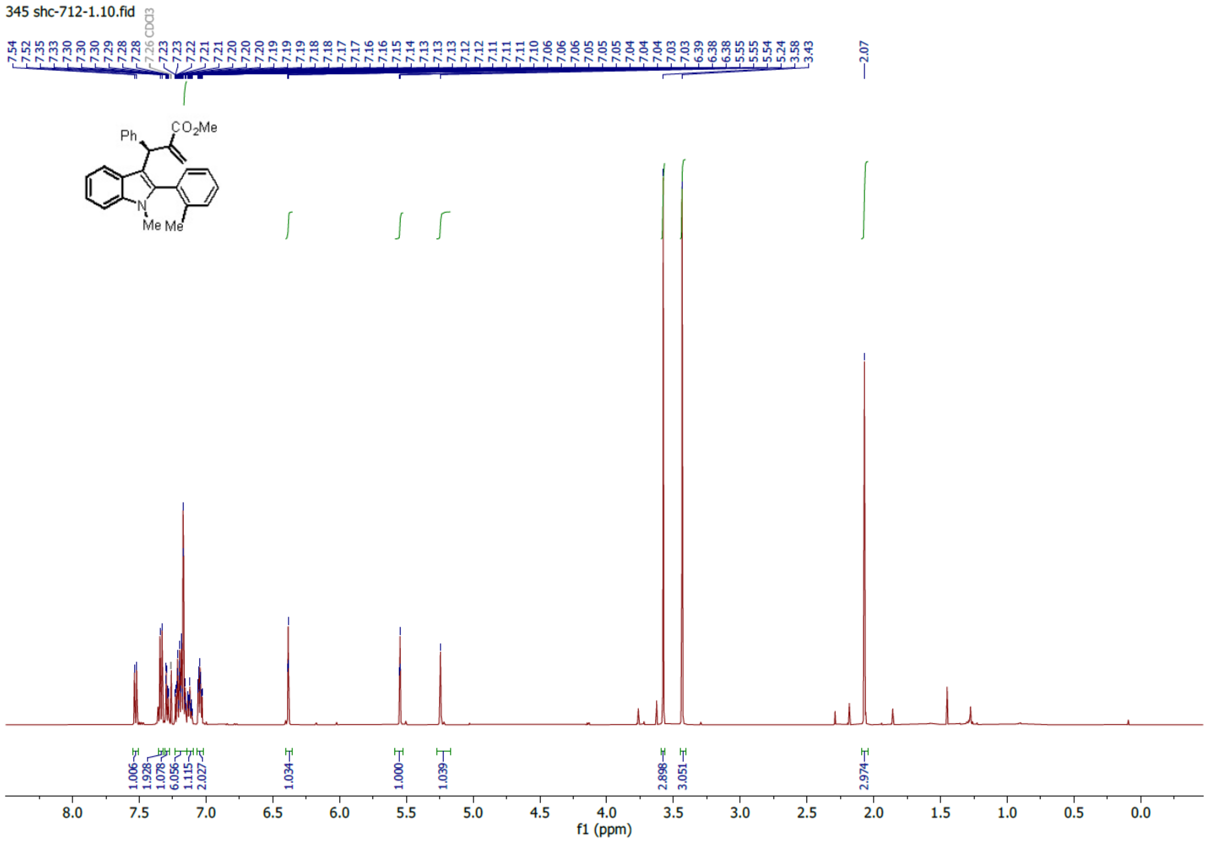

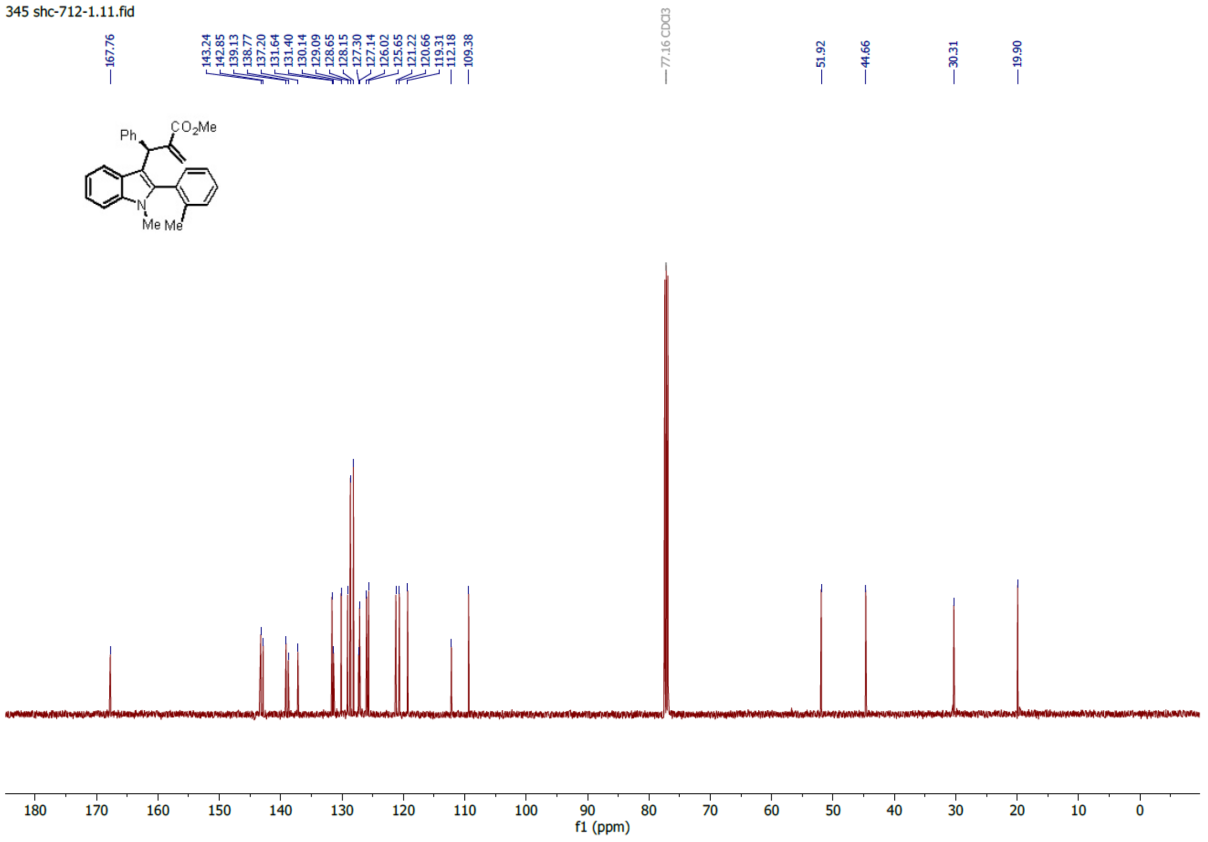

Compound **5g**

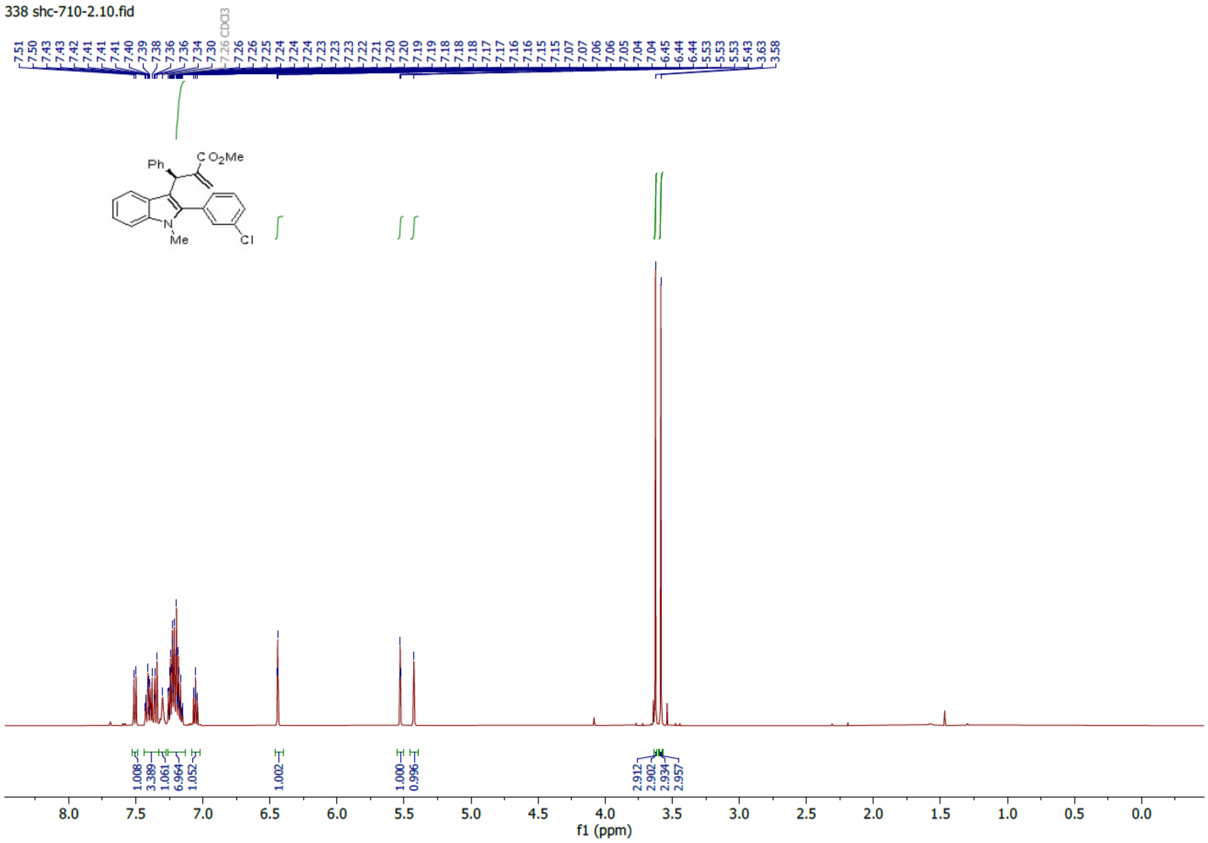
338 shc-710-2.10.fid
7.51
7.50
7.43
7.43
7.42
7.41
7.41
7.41
7.40
7.39
7.38
7.36
7.36
7.34
7.30
7.26 CDCl3
7.26
7.26
7.25
7.24
7.24
7.24
7.23
7.23
7.23
7.22
7.21
7.20
7.20
7.19
7.19
7.18
7.18
7.18
7.17
7.17
7.16
7.16
7.15
7.15
7.07
7.07
7.06
7.06
7.05
7.04
7.04
6.45
6.44
6.44
5.53
5.53
5.53
5.43
3.63
3.58
Ph
CO2Me
N
Me
Cl
1.008
3.389
1.061
6.964
1.052
1.002
1.000
0.996
2.912
2.902
2.934
2.957
8.0
7.5
7.0
6.5
6.0
5.5
5.0
4.5
4.0
3.5
3.0
2.5
2.0
1.5
1.0
0.5
0.0
f1 (ppm)

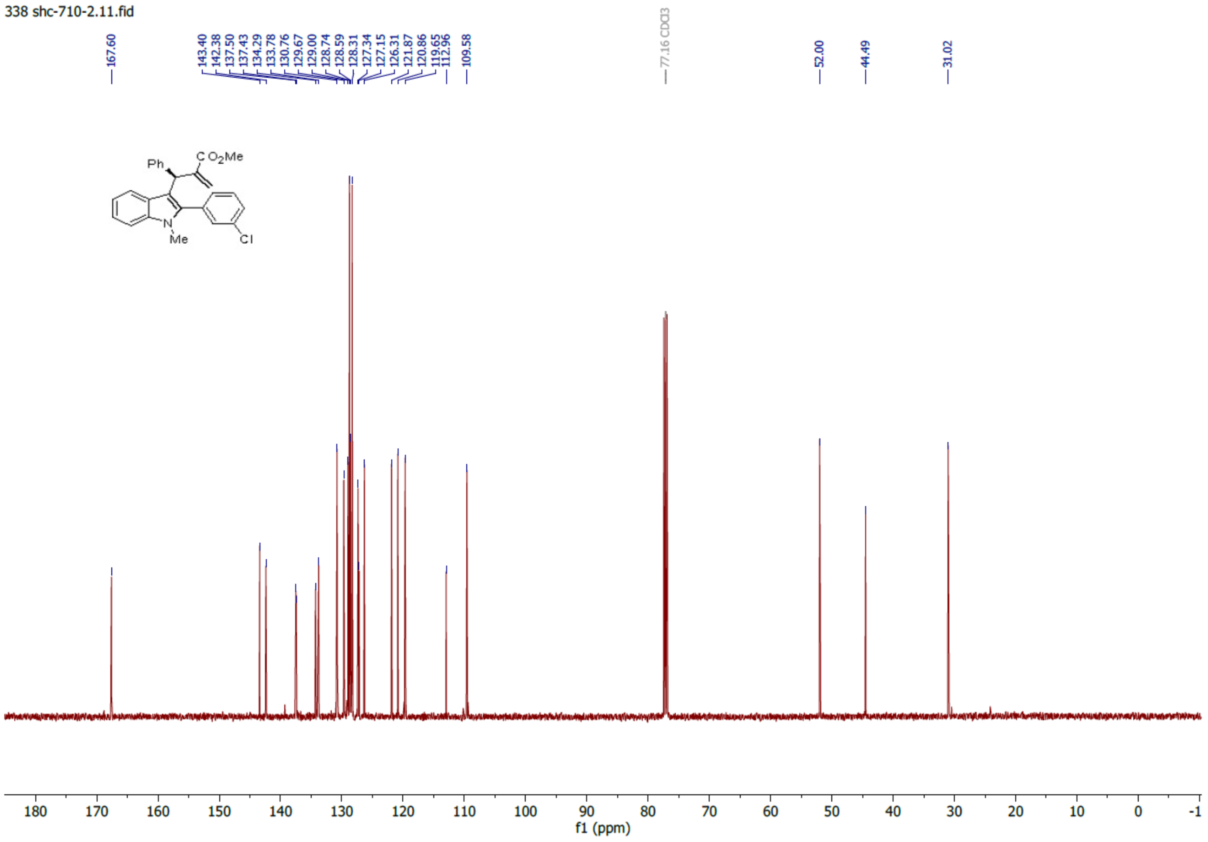
338 shc-710-2.11.fid
167.60
143.40
142.38
137.50
137.43
134.29
133.78
130.76
129.67
129.00
128.74
128.59
128.31
127.34
127.15
126.31
121.87
120.86
119.65
112.96
109.58
77.16 CDCl3
52.00
44.49
31.02
Ph
CO2Me
N
Me
Cl
180
170
160
150
140
130
120
110
100
90
80
70
60
50
40
30
20
10
0
-1
f1 (ppm)

Compound **5h**

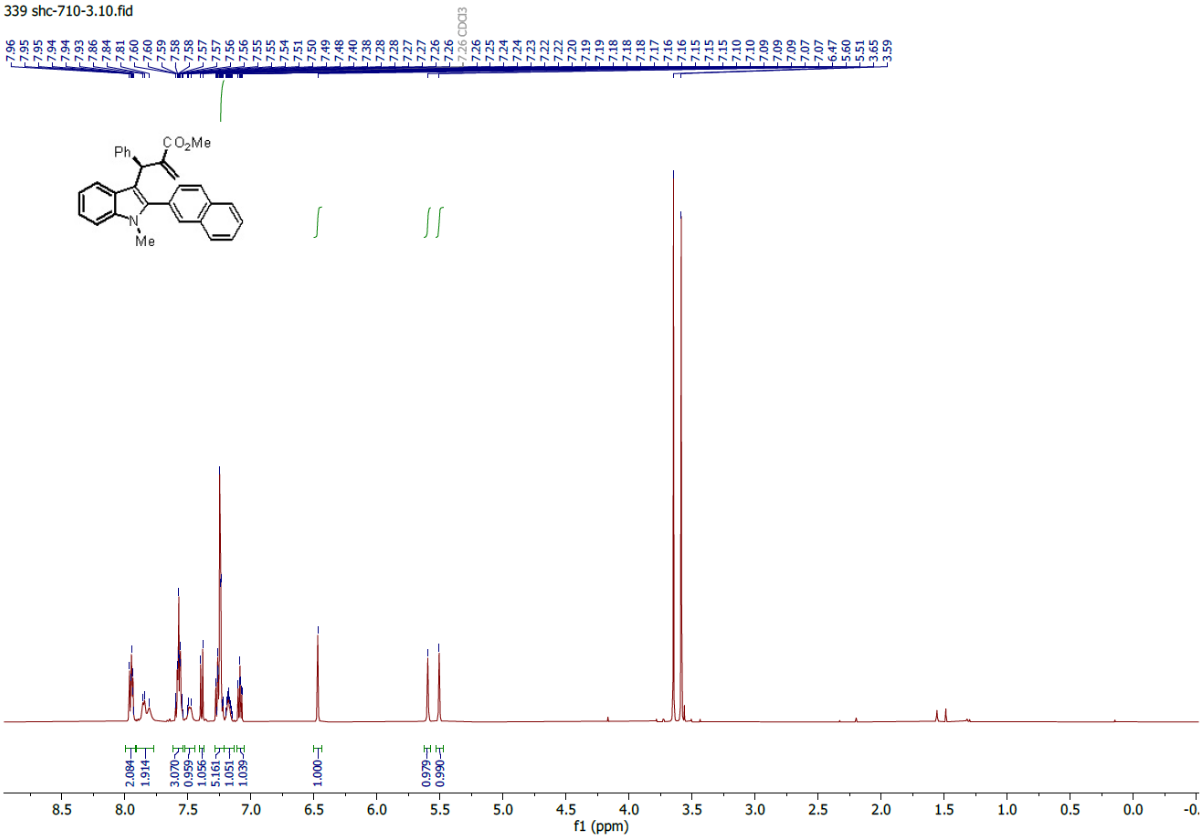
339 shc-710-3.10.fid
Ph
CO2Me
N
Me
f1 (ppm)

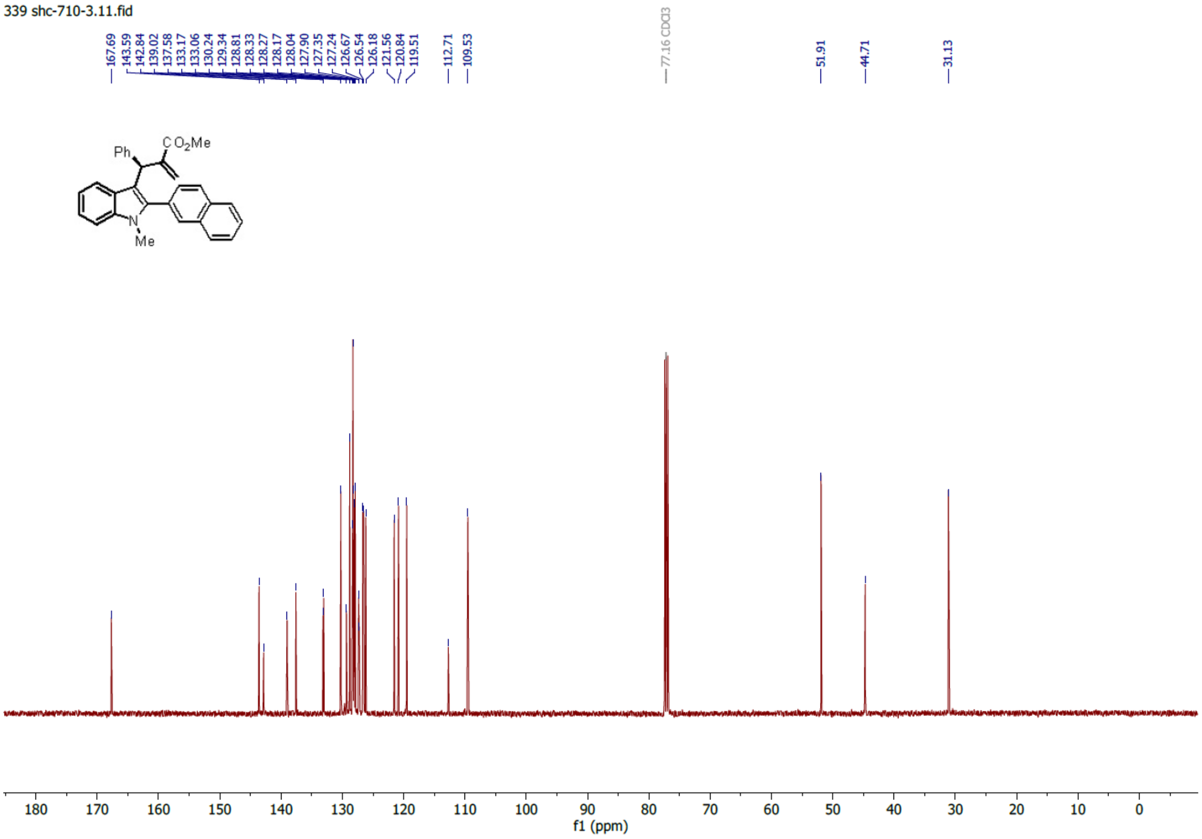
339 shc-710-3.11.fid
167.69
143.59
142.84
139.02
137.58
133.17
133.06
130.24
129.34
128.81
128.33
128.27
128.17
128.04
127.90
127.35
127.24
126.67
126.54
126.18
121.56
120.84
119.51
112.71
109.53
77.16 CDCl3
51.91
44.71
31.13
Ph
CO2Me
N
Me
f1 (ppm)

Compound **5i**

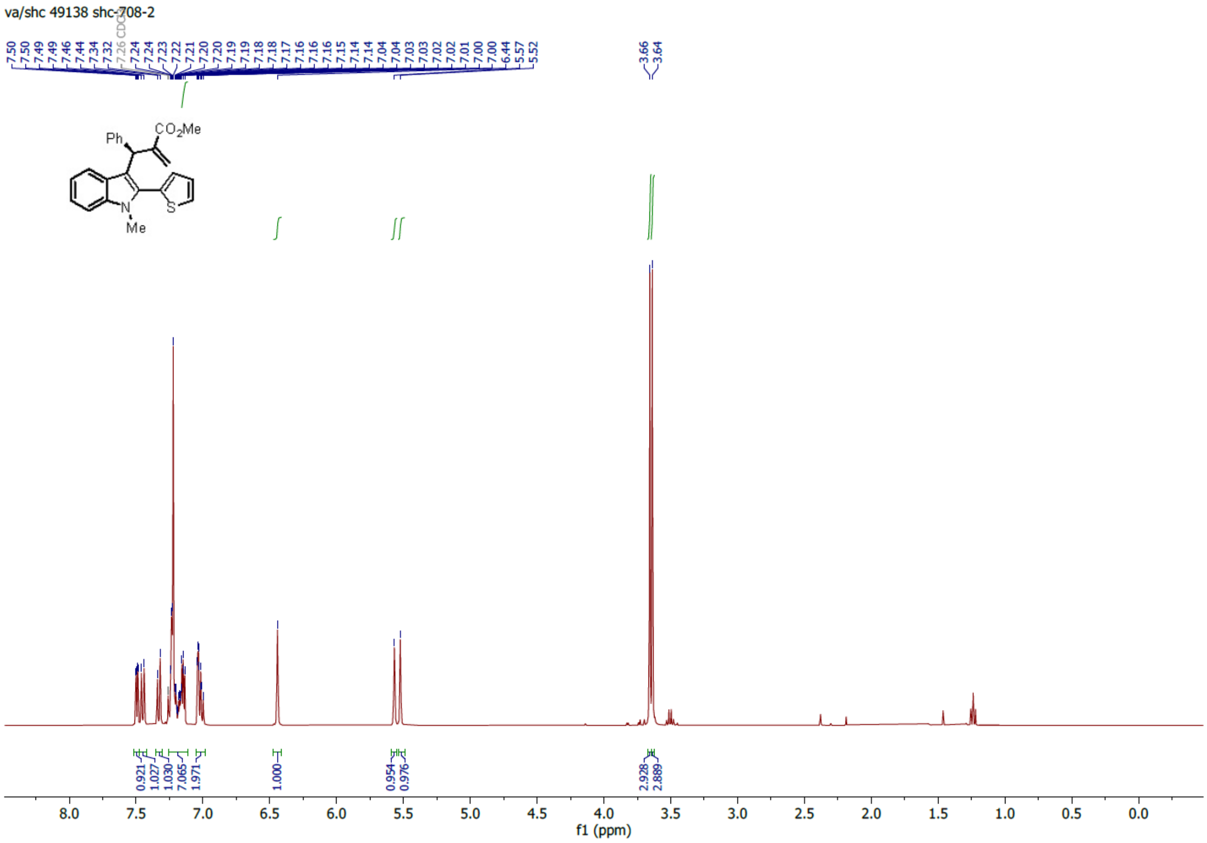

va/shc 49138 shc-708-2
7.50 7.50 7.49 7.49 7.46 7.44 7.34 7.32 7.26 CDCl3 7.24 7.24 7.23 7.22 7.21 7.20 7.20 7.19 7.19 7.18 7.18 7.17 7.16 7.16 7.15 7.14 7.14 7.04 7.04 7.03 7.03 7.02 7.02 7.01 7.00 7.00 6.44 5.57 5.52
3.66 3.64
Ph
CO2Me
N
Me
S
0.921 1.027 1.030 7.065 1.971 1.000 0.954 0.976 2.928 2.889
8.0 7.5 7.0 6.5 6.0 5.5 5.0 4.5 4.0 3.5 3.0 2.5 2.0 1.5 1.0 0.5 0.0
f1 (ppm)

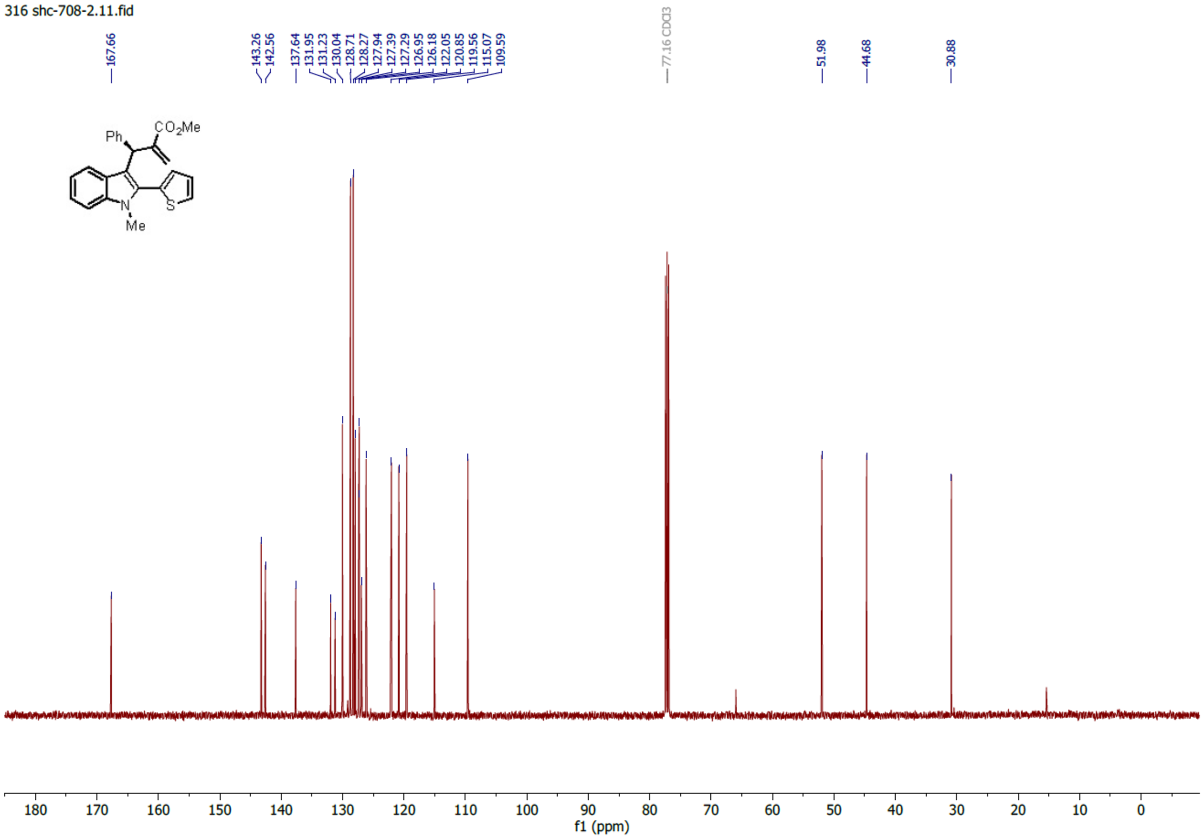

316 shc-708-2.11.fid
167.66
143.26 142.56 137.64 131.95 131.23 130.04 128.71 128.27 127.94 127.39 127.29 126.95 126.18 122.05 120.85 119.56 115.07 109.59
77.16 CDCl3
51.98
44.68
30.88
Ph
CO2Me
N
Me
S
180 170 160 150 140 130 120 110 100 90 80 70 60 50 40 30 20 10 0
f1 (ppm)

Compound **5j**

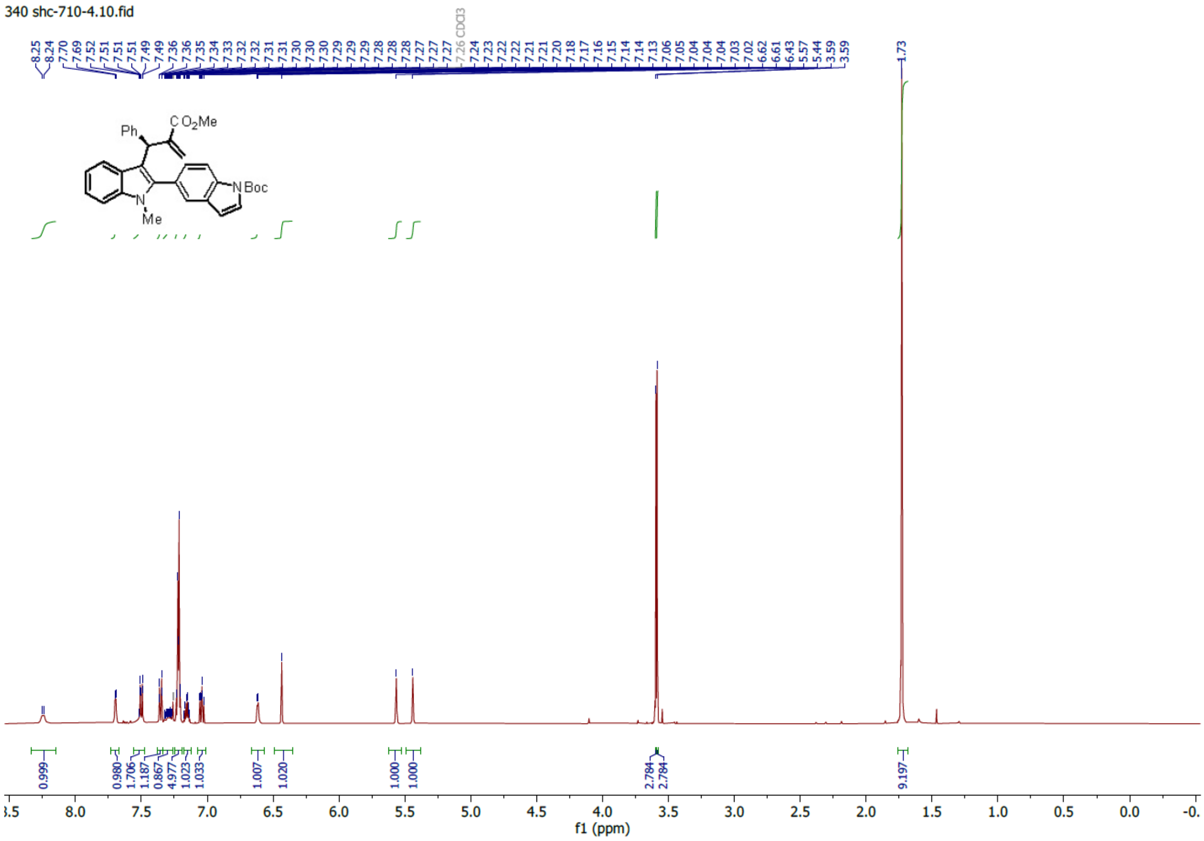

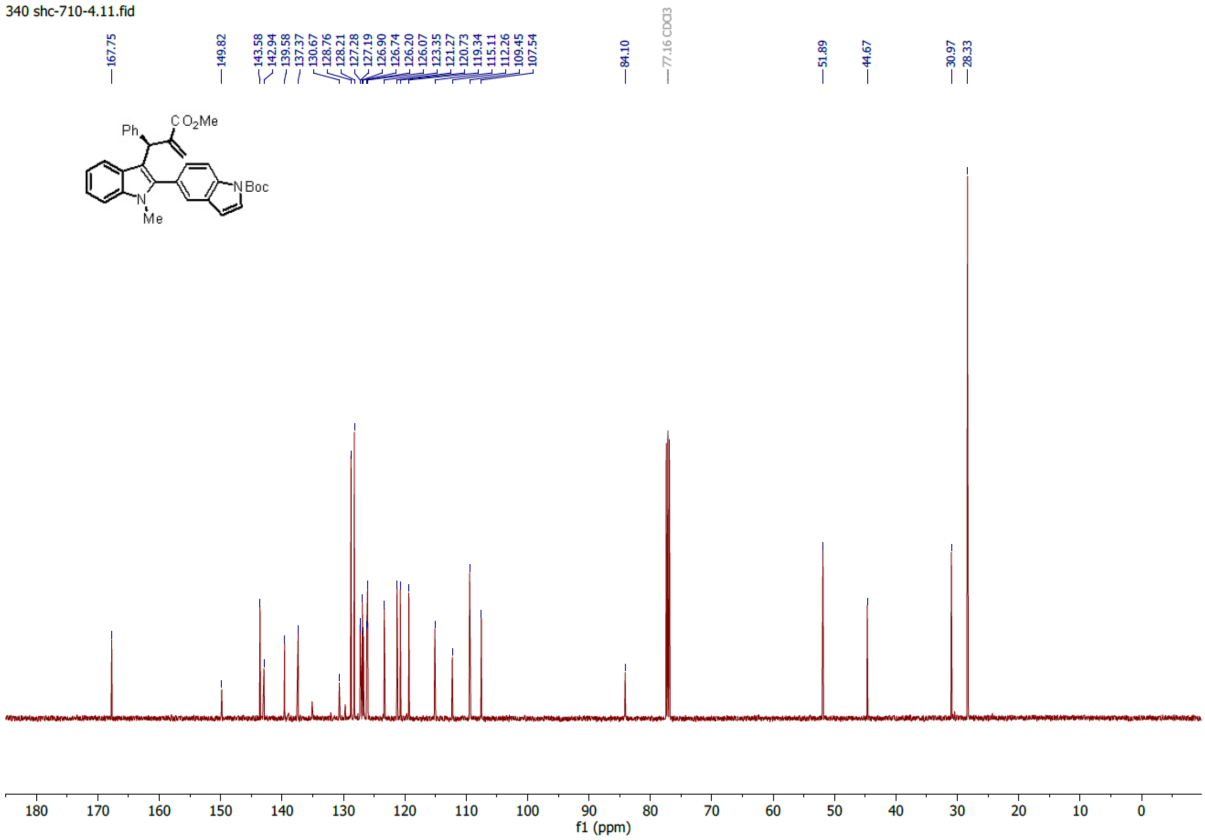

Compound **5k**

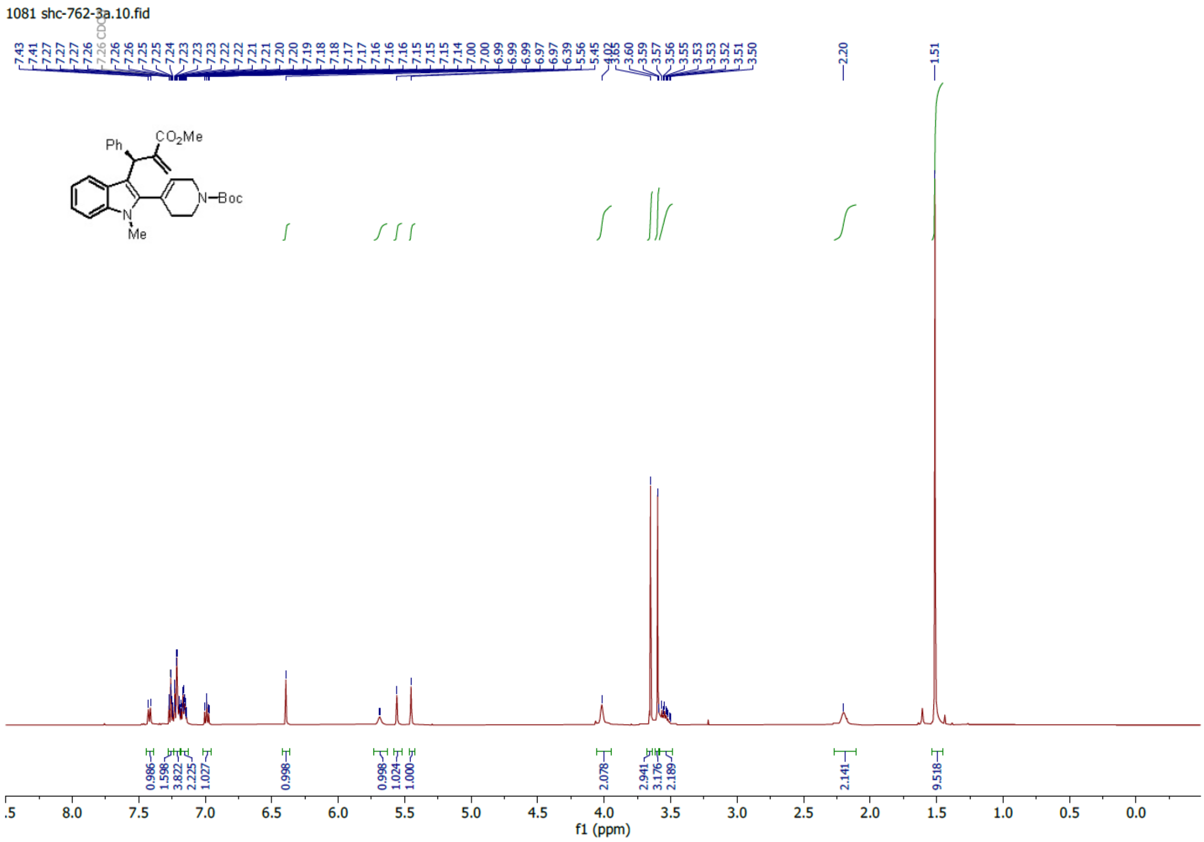

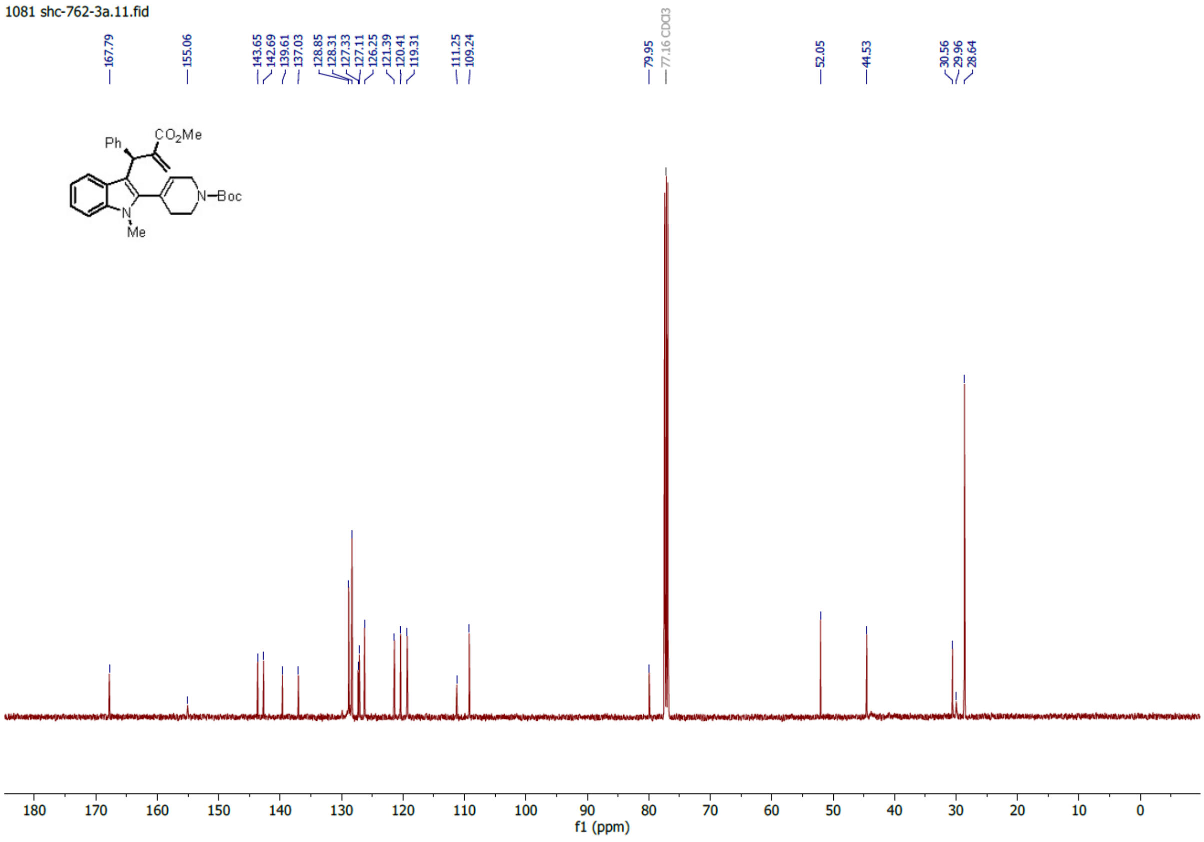

Compound **5l**

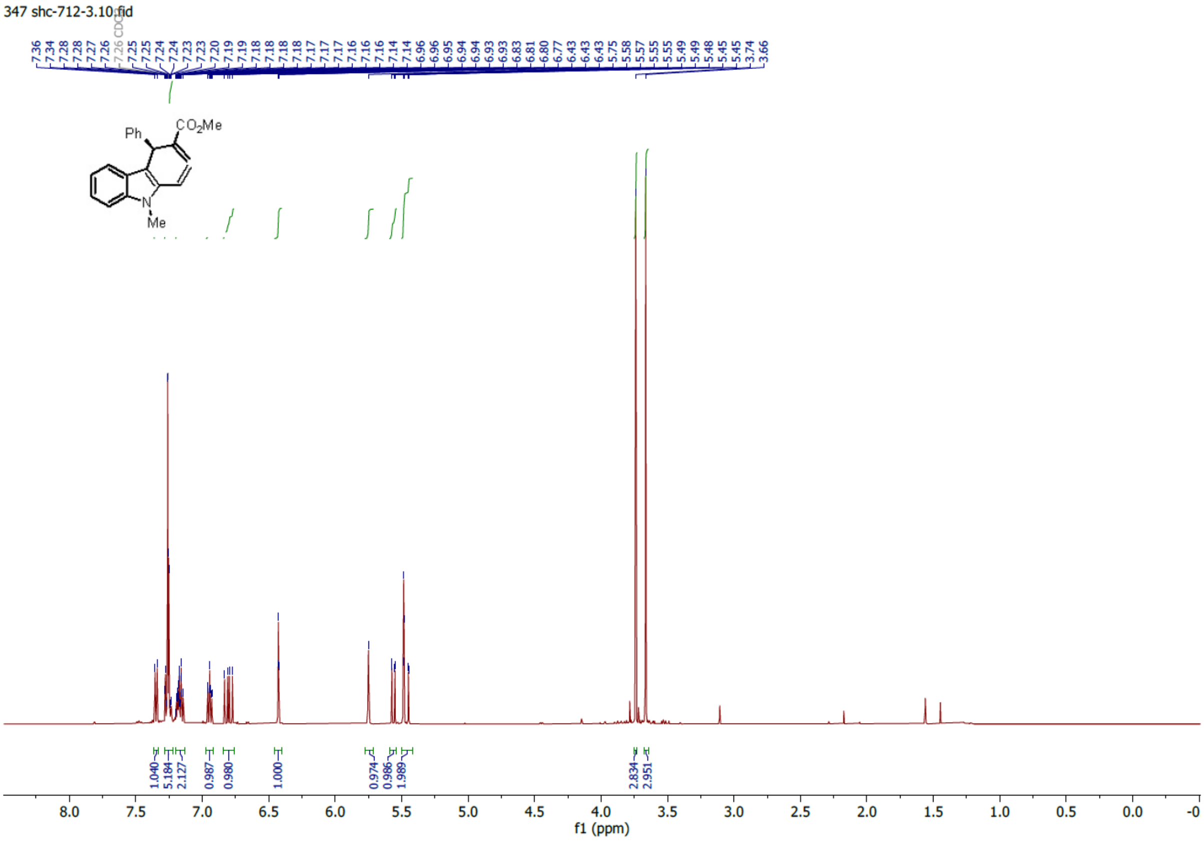

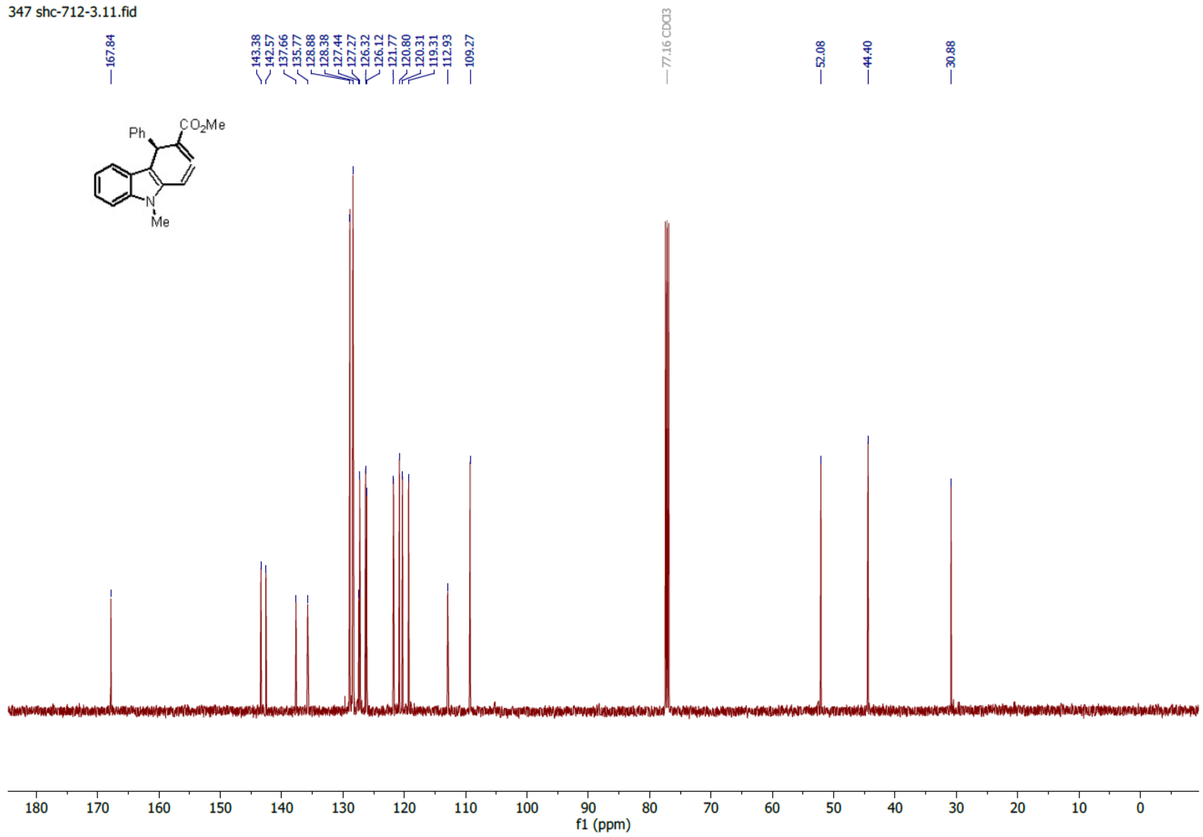

Compound **5m**

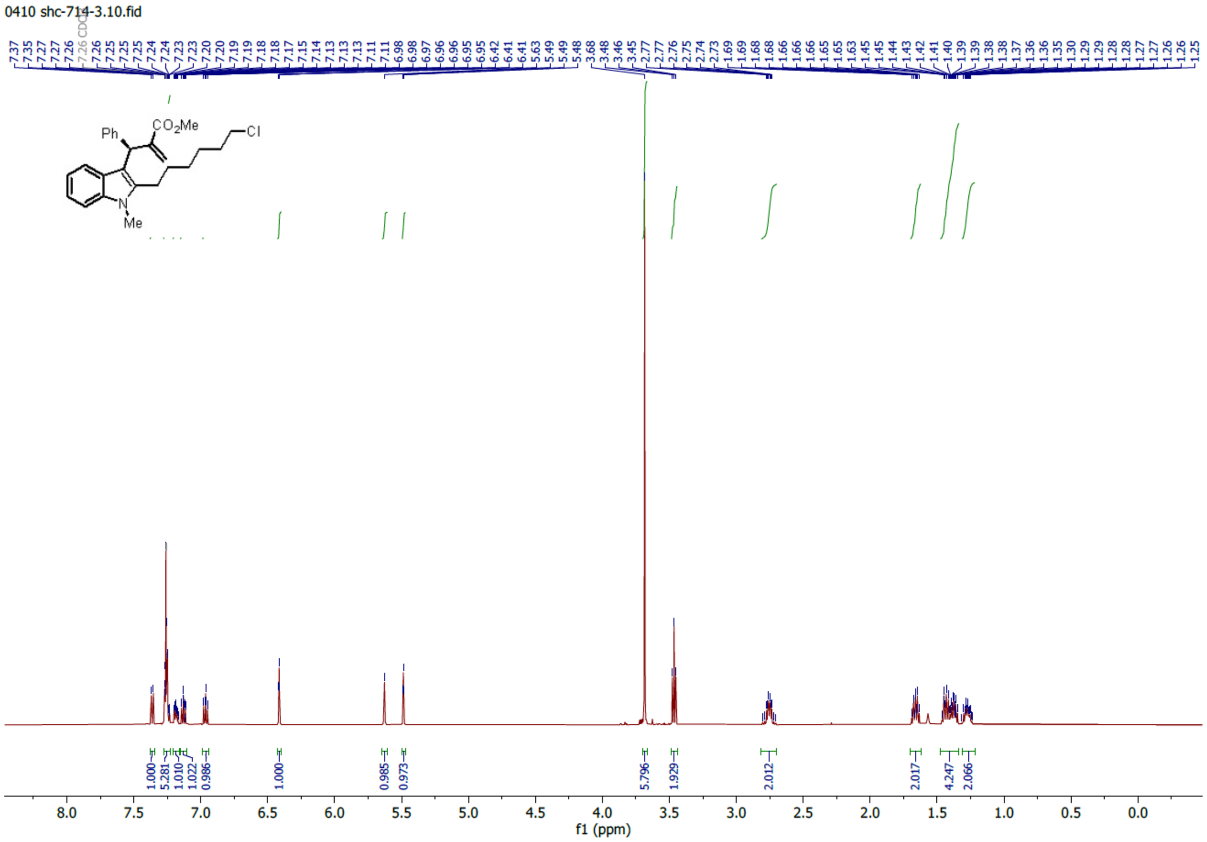

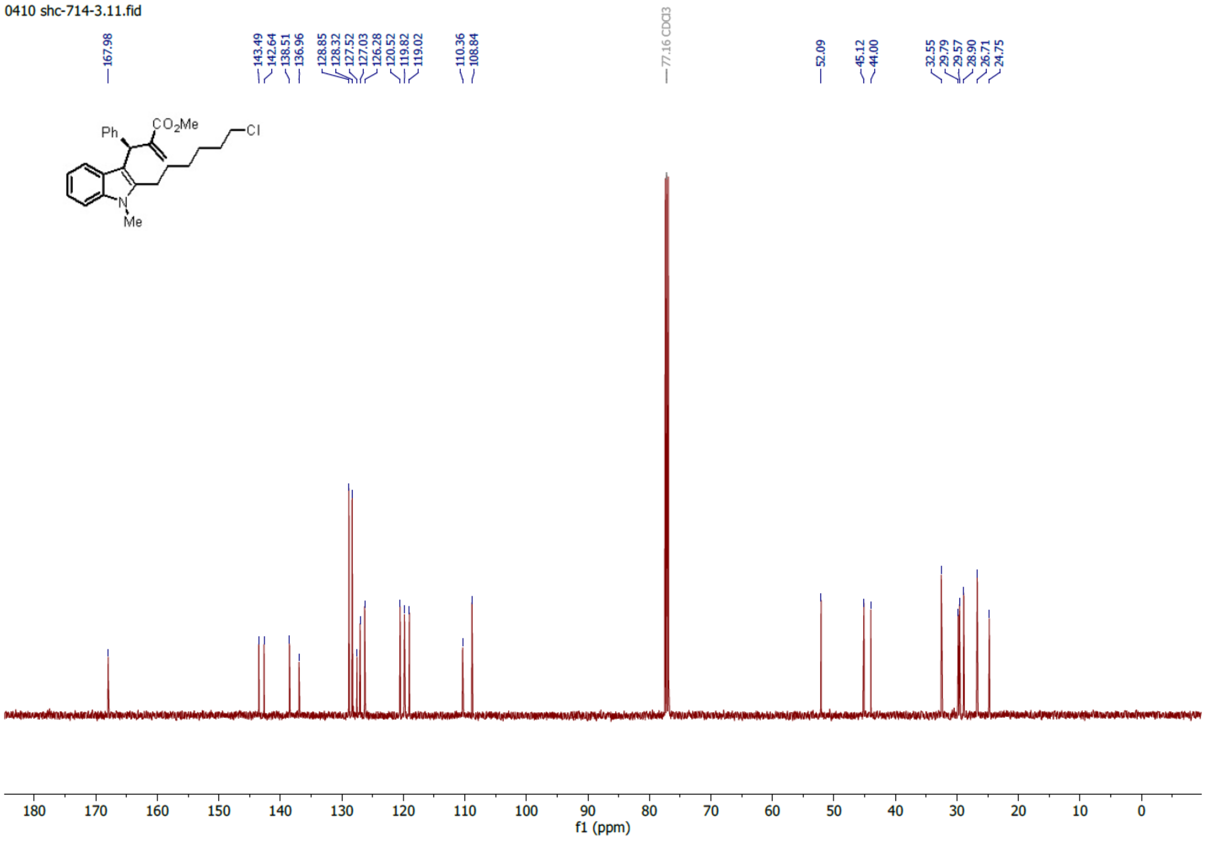

Compound **5n**

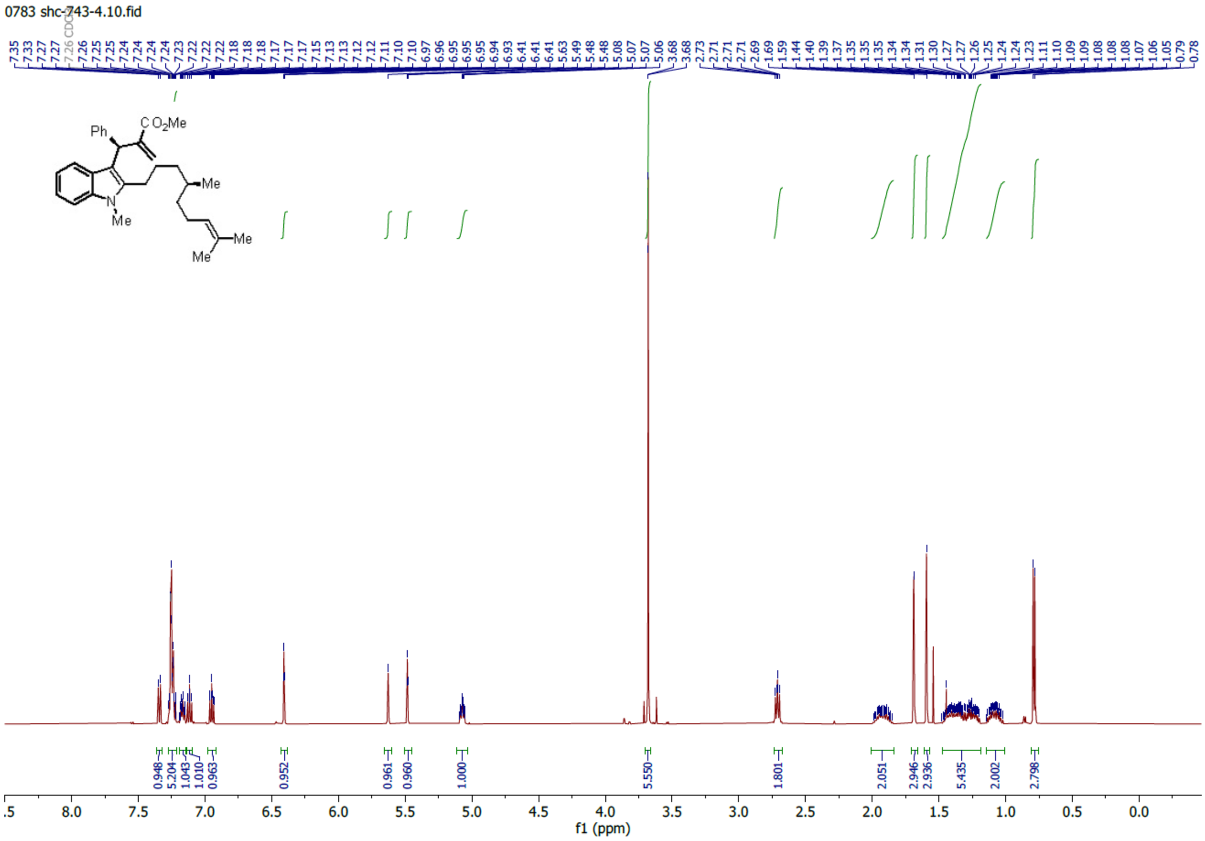

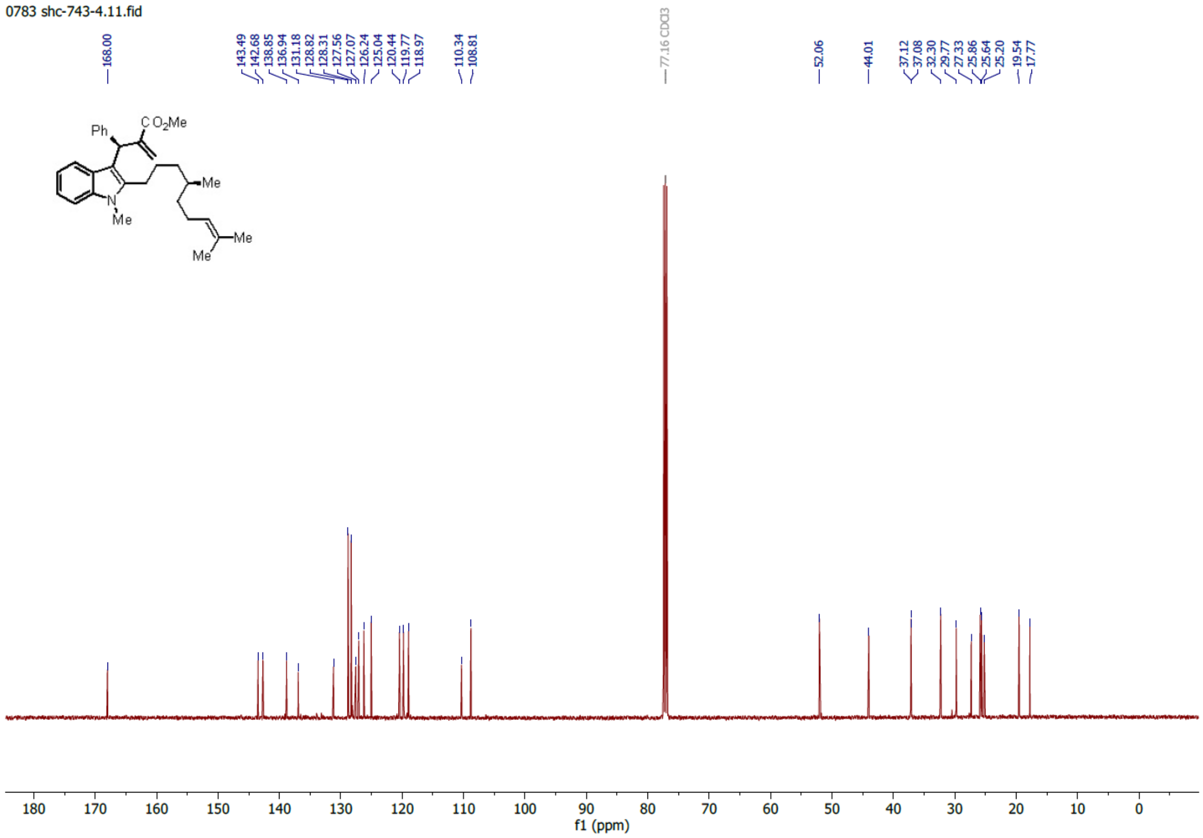

Compound **5o**

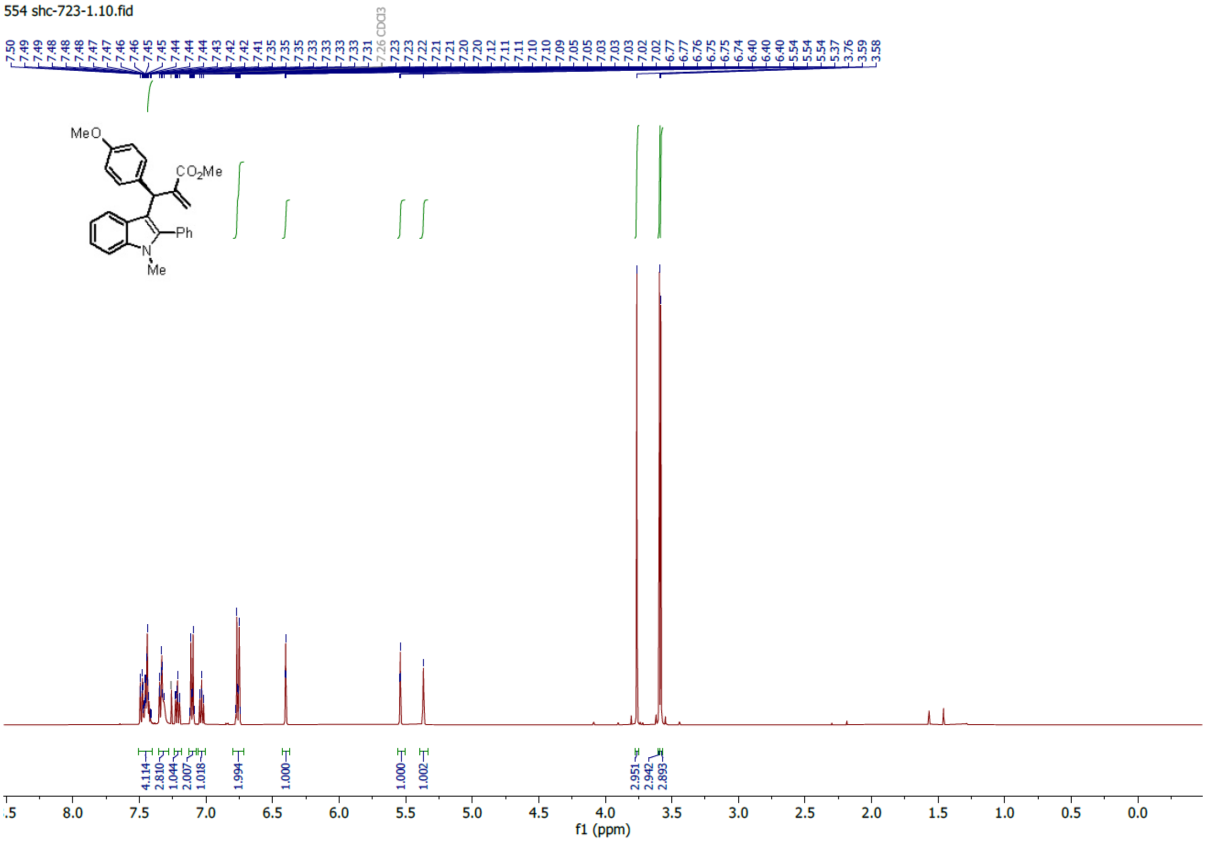
554 shc-723-1.10.fid
MeO
CO2Me
Ph
N
Me
f1 (ppm)

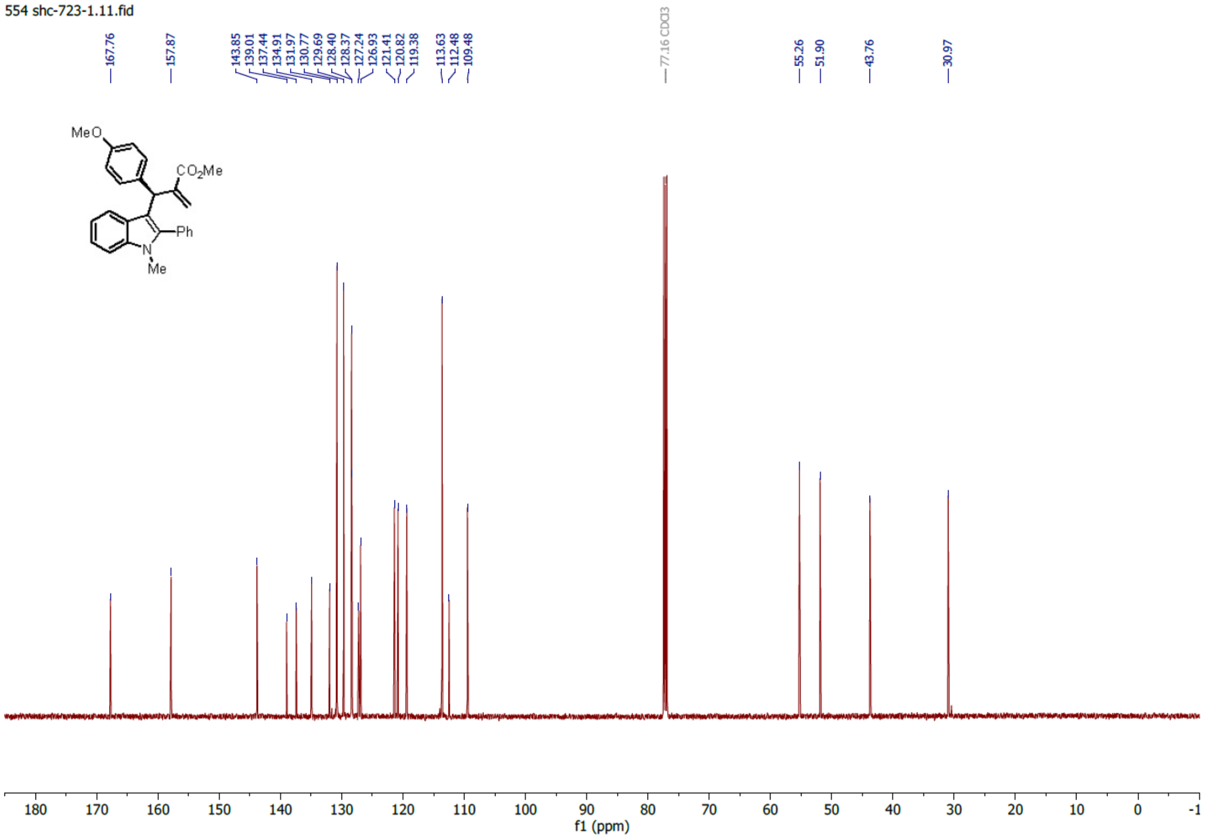
554 shc-723-1.11.fid
167.76
157.87
143.85
139.01
137.44
134.91
131.97
130.77
129.69
128.40
128.37
127.24
126.93
121.41
120.82
119.38
113.63
112.48
109.48
77.16 CDCl3
55.26
51.90
43.76
30.97
MeO
CO2Me
Ph
N
Me
f1 (ppm)

Compound **5p**

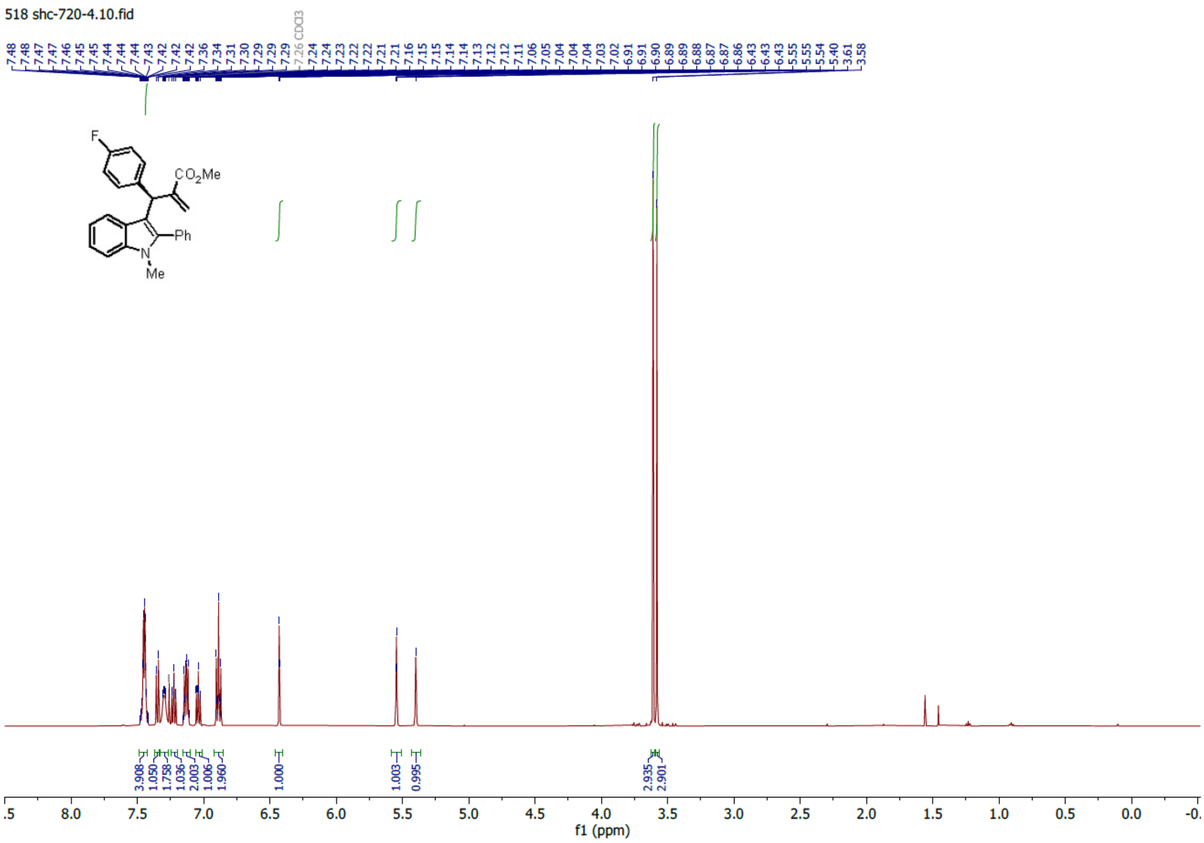
518 shc-720-4.10.fid
7.48 7.48 7.47 7.47 7.46 7.45 7.45 7.44 7.44 7.43 7.42 7.42 7.42 7.36 7.34 7.31 7.30 7.29 7.29 7.29 7.26 CDCl3 7.24 7.24 7.23 7.23 7.22 7.22 7.21 7.21 7.16 7.15 7.15 7.14 7.14 7.13 7.12 7.12 7.11 7.06 7.05 7.04 7.04 7.03 7.02 6.91 6.91 6.90 6.89 6.89 6.88 6.87 6.87 6.86 6.43 6.43 6.43 5.55 5.55 5.54 5.40 3.61 3.58
F
CO2Me
Ph
N
Me
3.908 1.050 1.758 1.036 2.003 1.006 1.960 1.000 1.003 0.995 2.935 2.901
f1 (ppm)

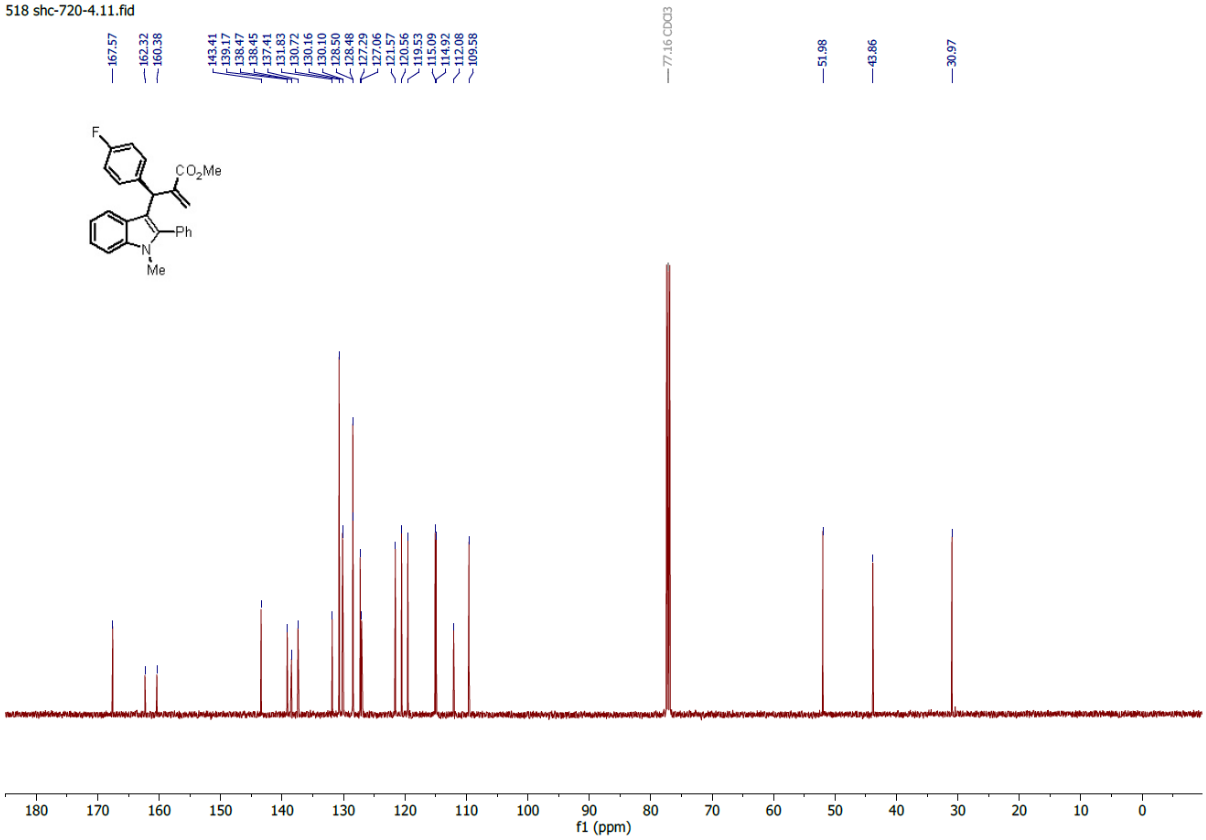
518 shc-720-4.11.fid
167.57 162.32 160.38 143.41 139.17 138.47 138.45 137.41 131.83 130.72 130.16 130.10 128.50 128.48 127.29 127.06 121.57 120.56 119.53 115.09 114.92 112.08 109.58 77.16 CDCl3 51.98 43.86 30.97
F
CO2Me
Ph
N
Me
f1 (ppm)

Compound **5q**

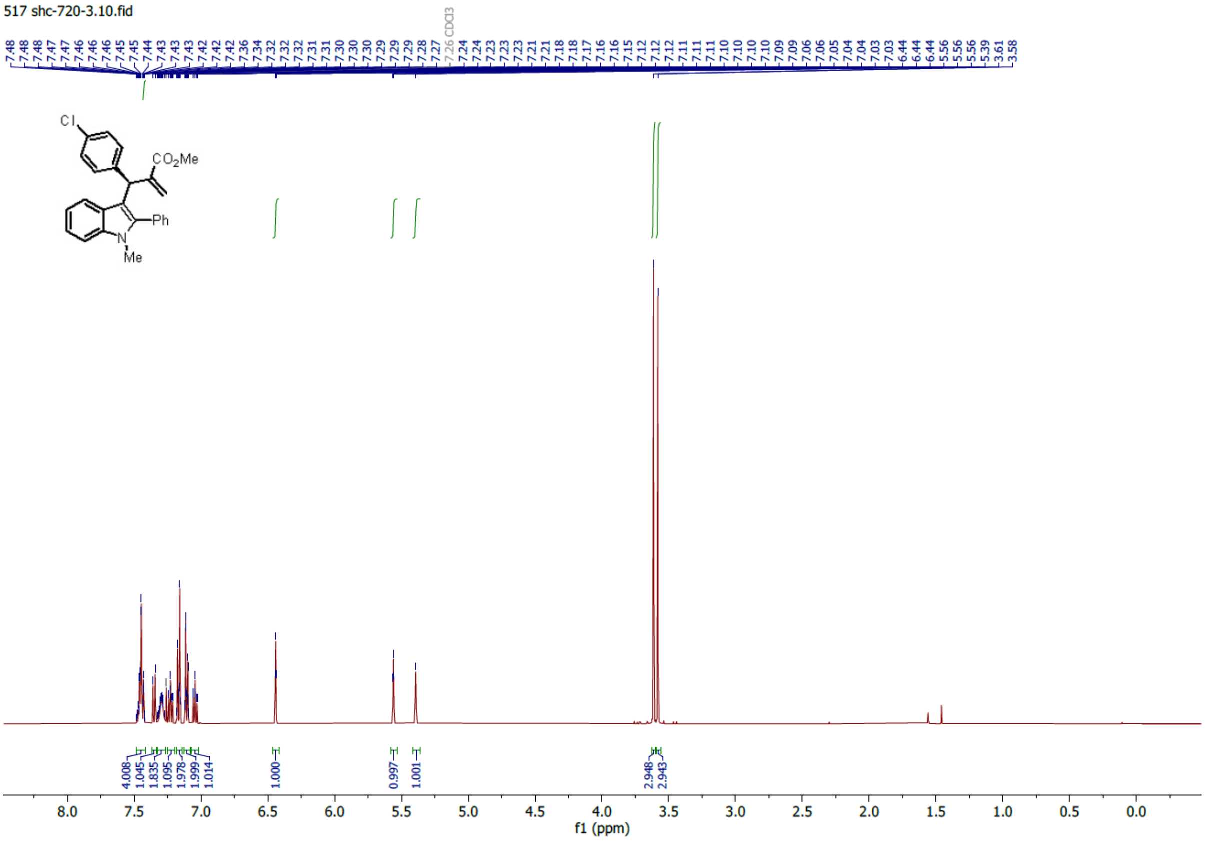

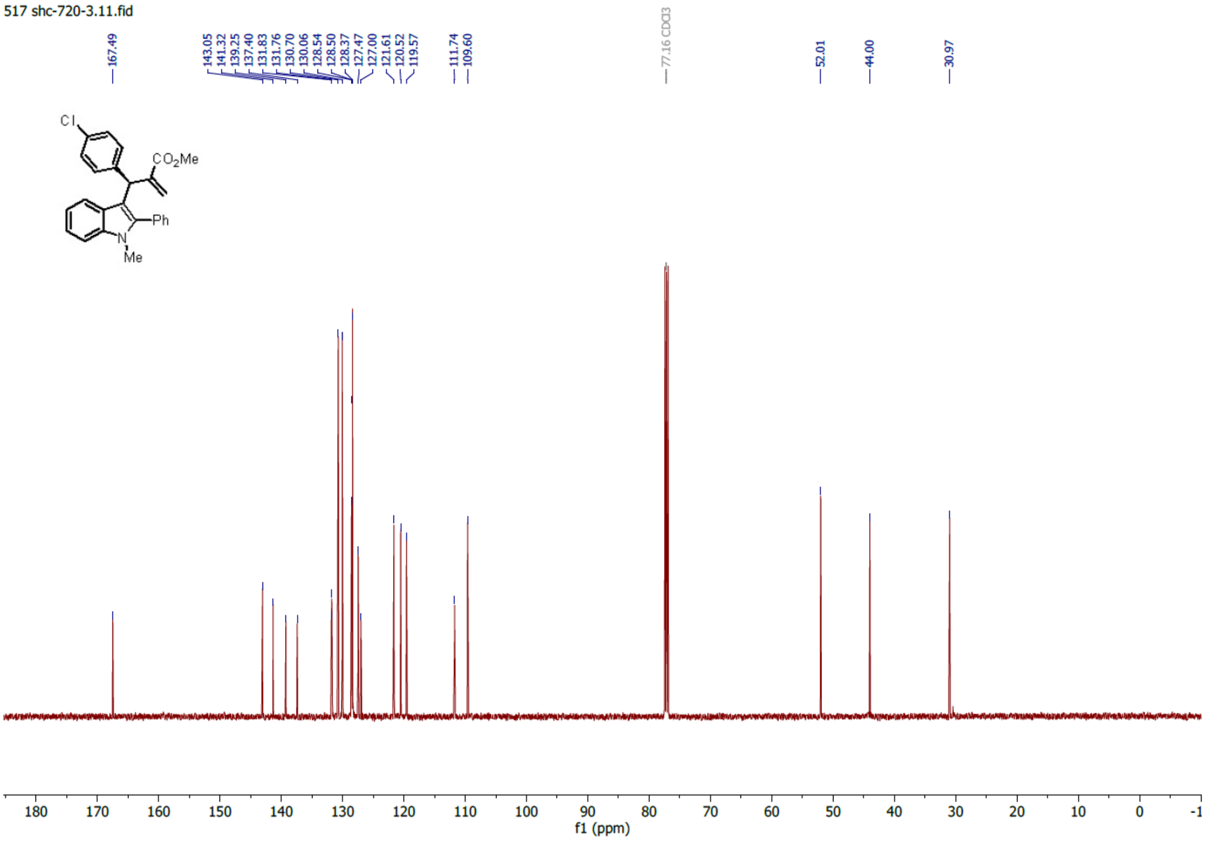

Compound **5r**

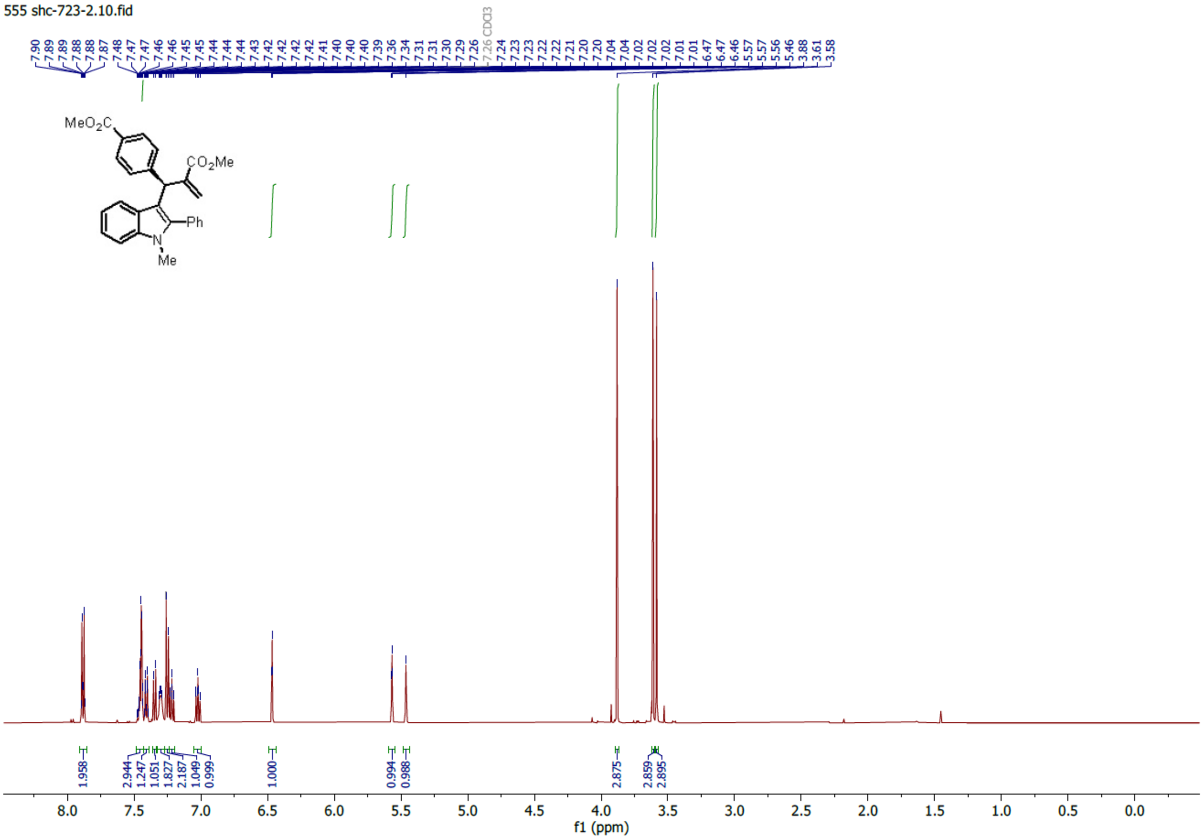

555 shc-723-2.10.fid
MeO2C
CO2Me
Ph
N
Me
f1 (ppm)

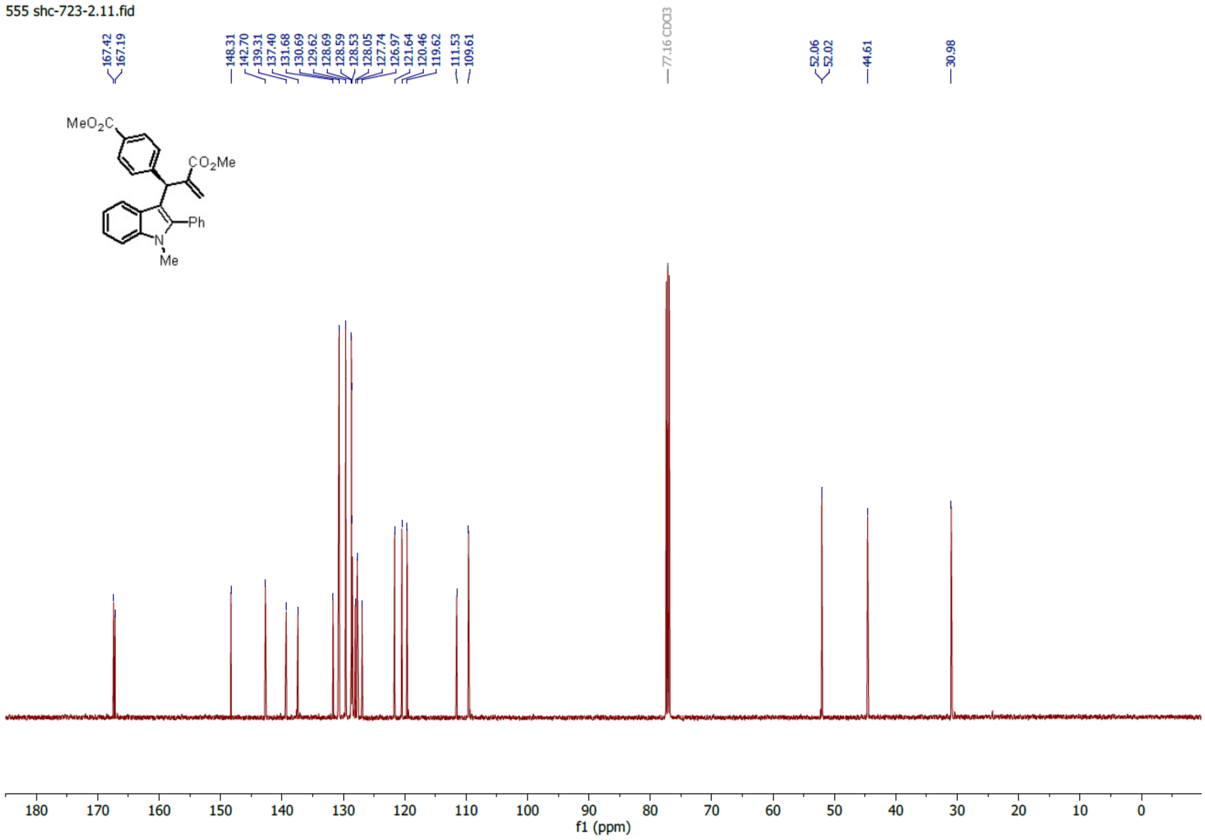

555 shc-723-2.11.fid
MeO2C
CO2Me
Ph
N
Me
f1 (ppm)

Compound **5s**

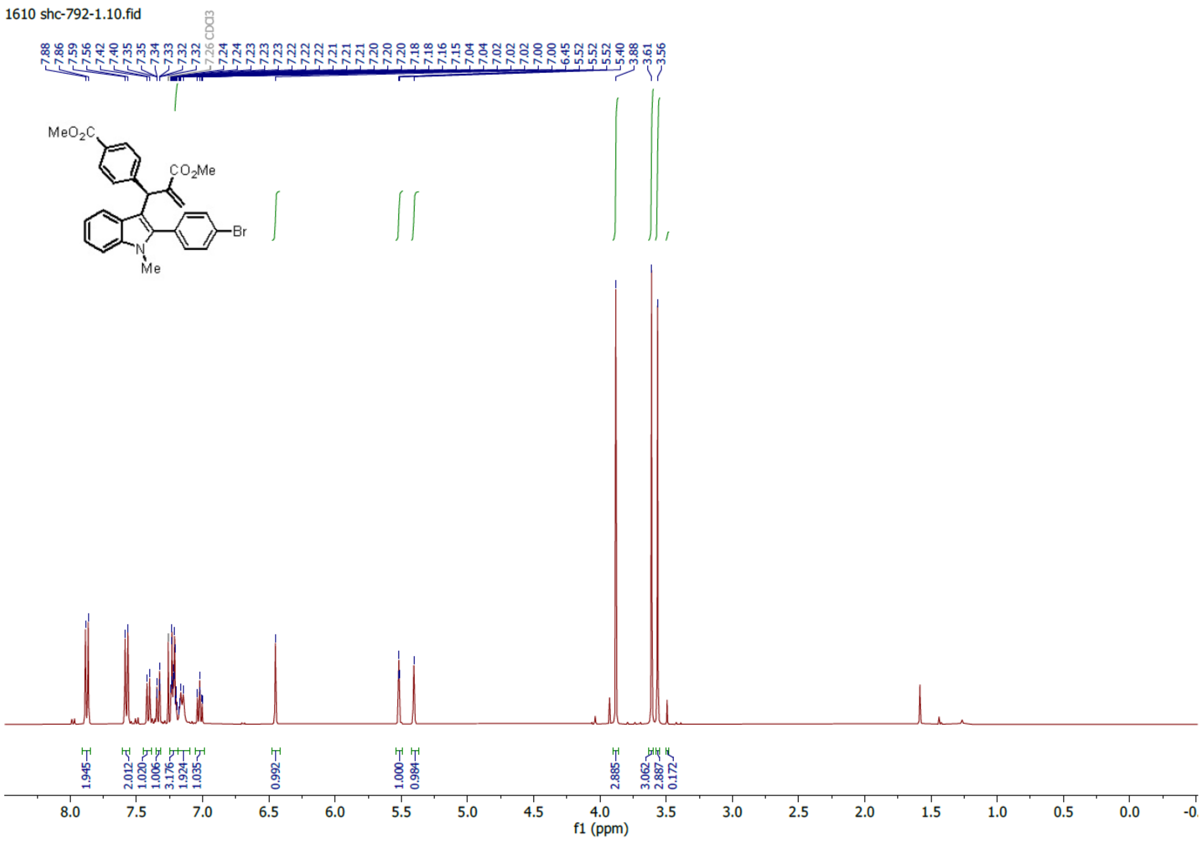
1610 shc-792-1.10.fid
7.88 7.86 7.59 7.56 7.42 7.40 7.35 7.35 7.34 7.33 7.32 7.32 7.26 CDCl3 7.24 7.24 7.23 7.23 7.23 7.22 7.22 7.22 7.21 7.21 7.21 7.20 7.20 7.20 7.18 7.18 7.16 7.15 7.04 7.04 7.02 7.02 7.02 7.00 7.00 6.45 5.52 5.52 5.52 5.40 3.88 3.61 3.56
MeO2C
CO2Me
Br
N
Me
1.945 2.012 1.020 1.006 3.176 1.924 1.035 0.992 1.000 0.984 2.885 3.062 2.887 0.172
f1 (ppm)

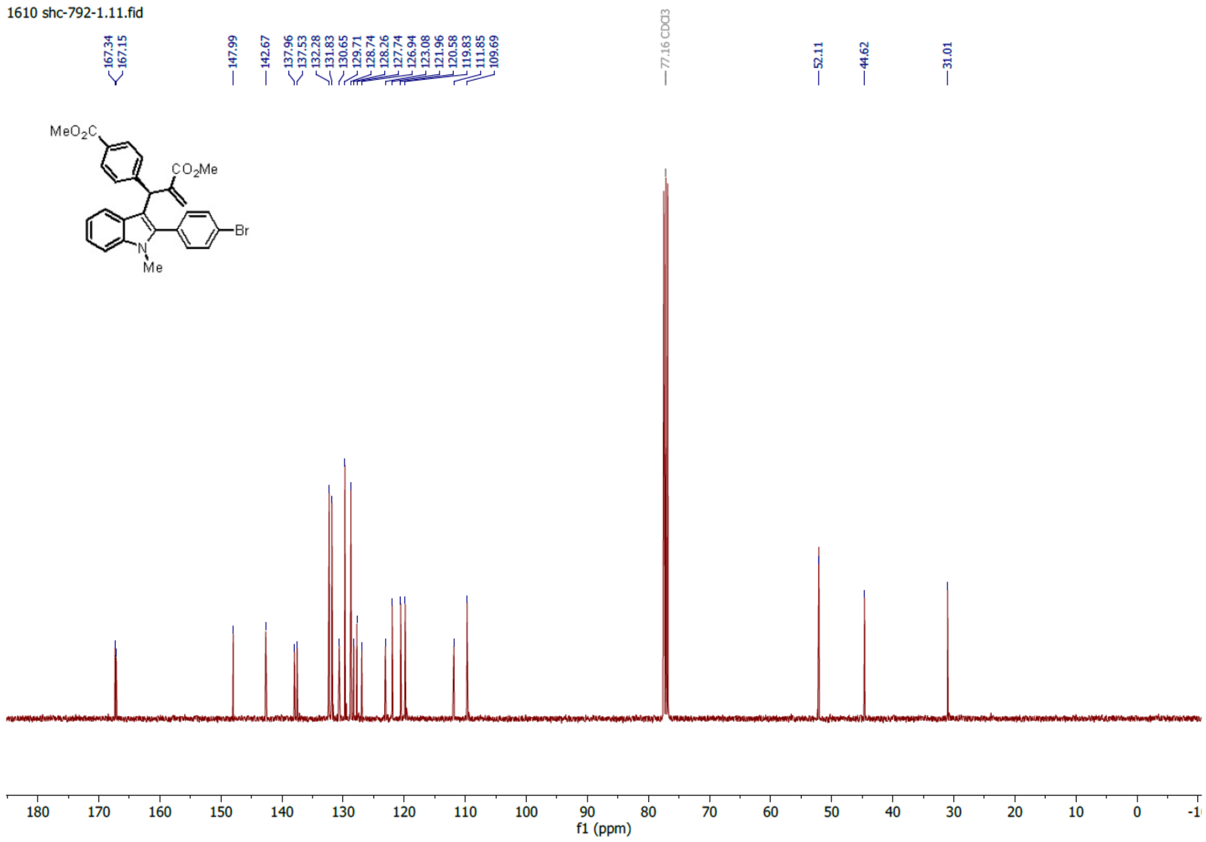
1610 shc-792-1.11.fid
167.34 167.15 147.99 142.67 137.96 137.53 132.28 131.83 130.65 129.71 128.74 128.26 127.74 126.94 123.08 121.96 120.58 119.83 111.85 109.69 77.16 CDCl3 52.11 44.62 31.01
MeO2C
CO2Me
Br
N
Me
f1 (ppm)

Compound **5t**

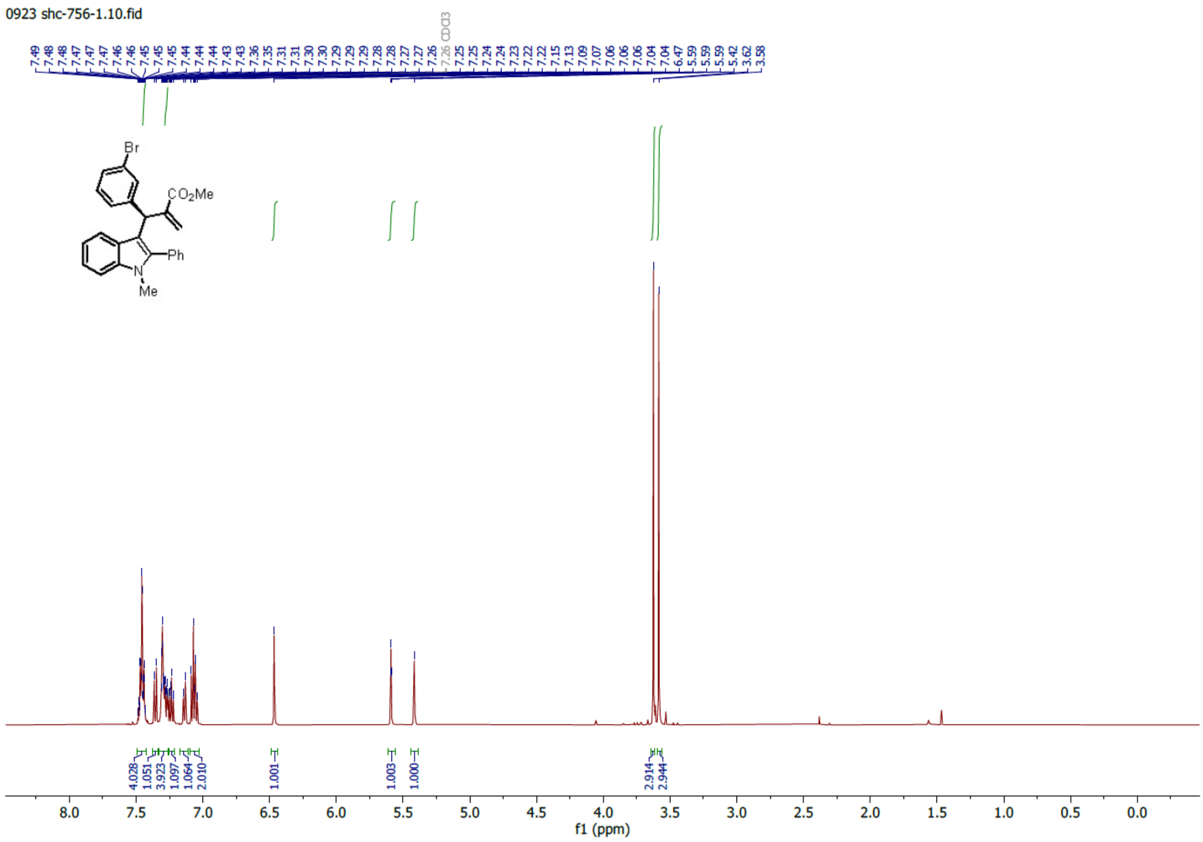

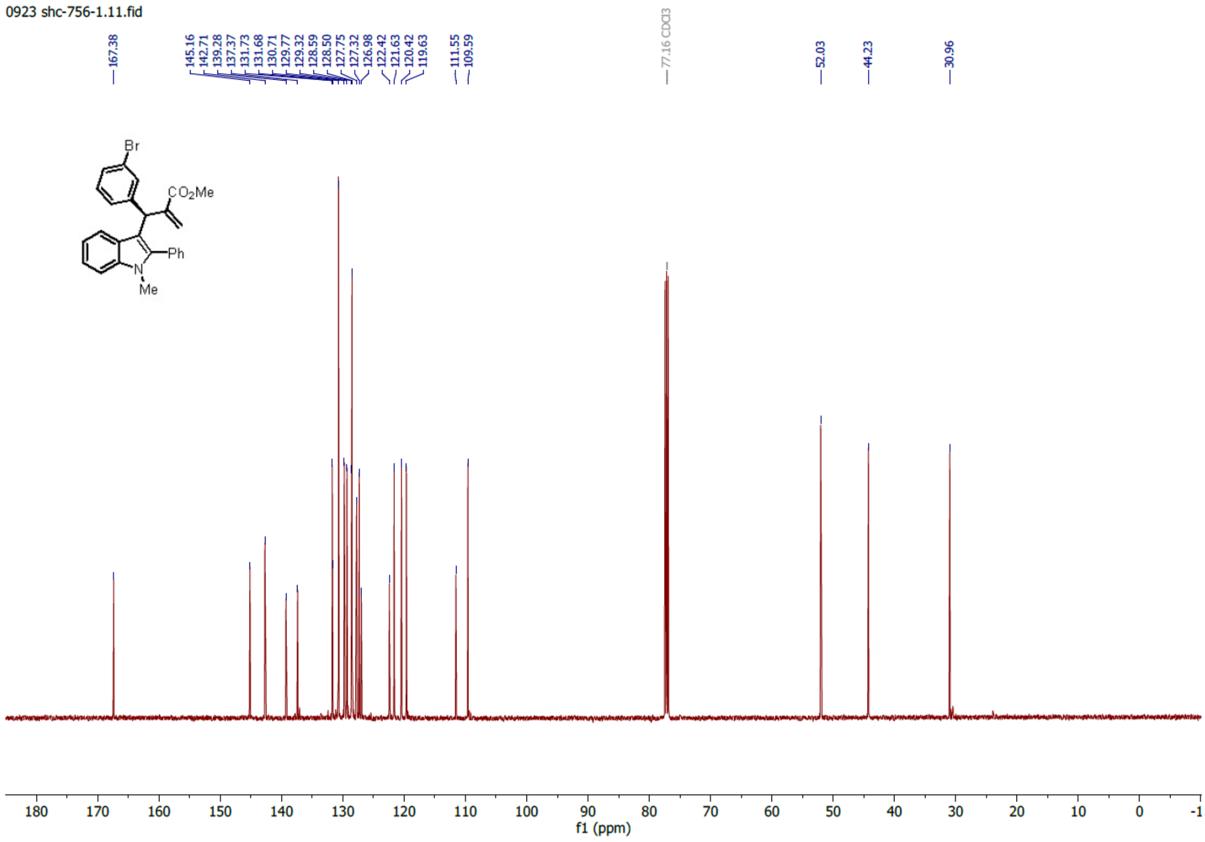

Compound **5u**

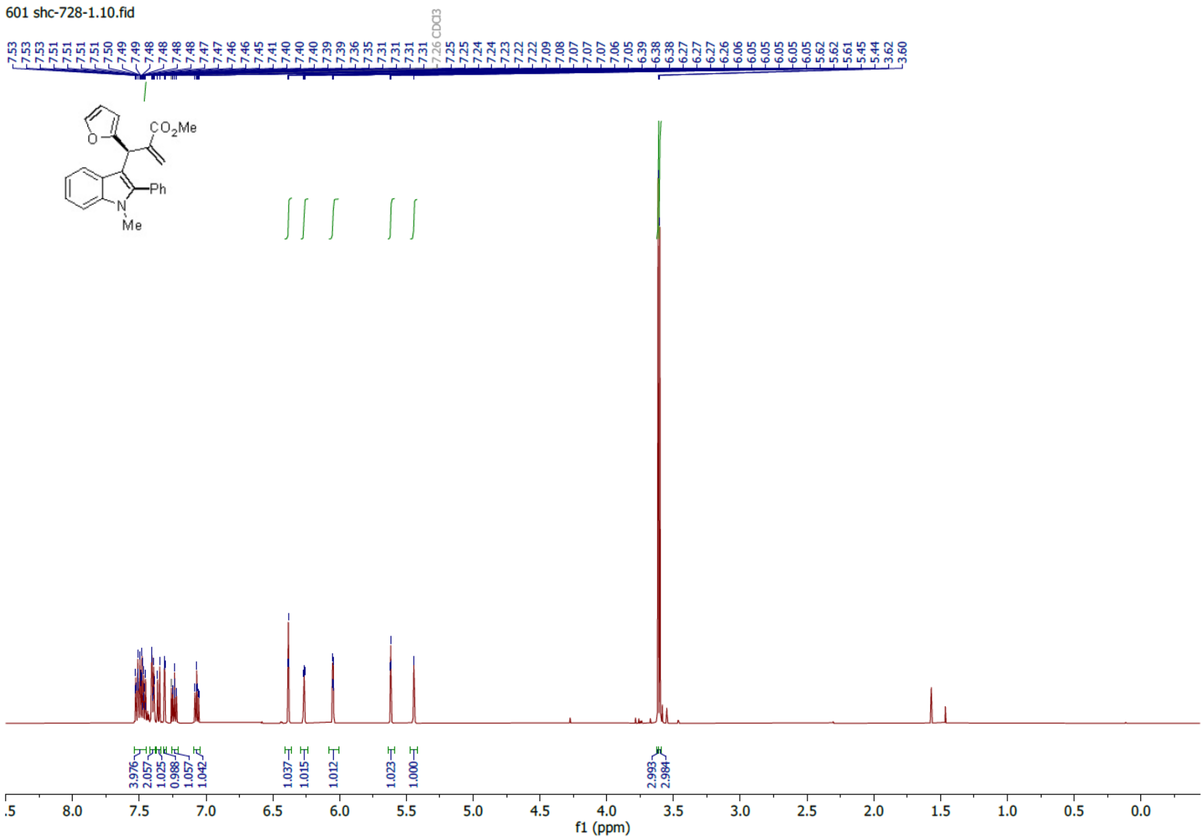

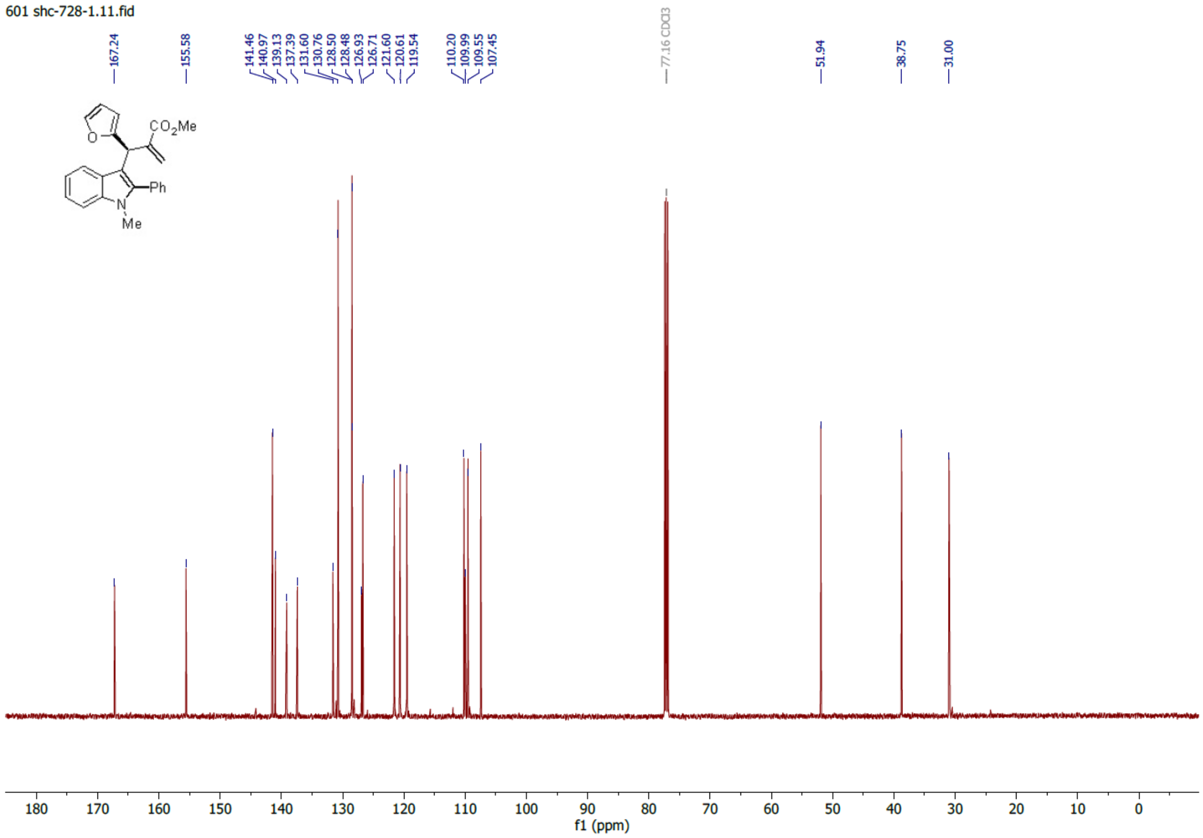

Compound **5v**

602 shc-728-2.10.fid

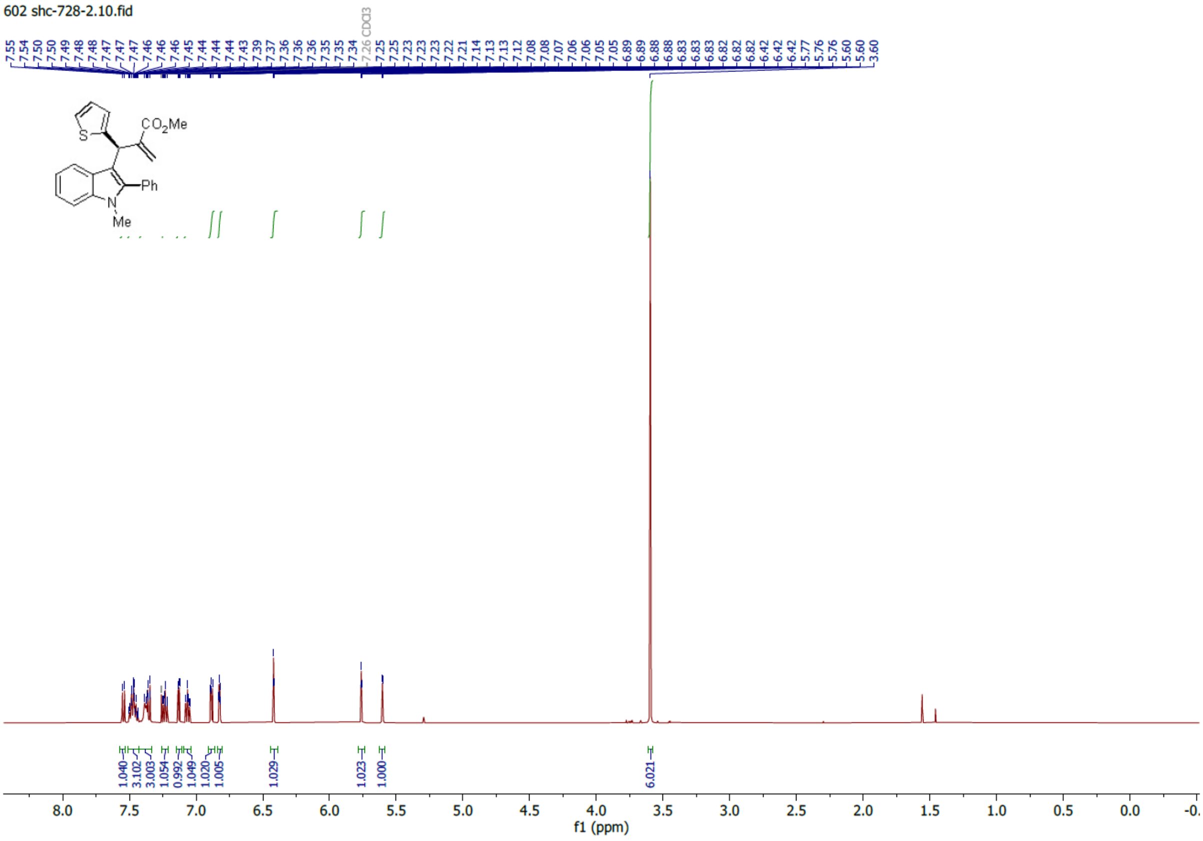

602 shc-728-2.11.fid

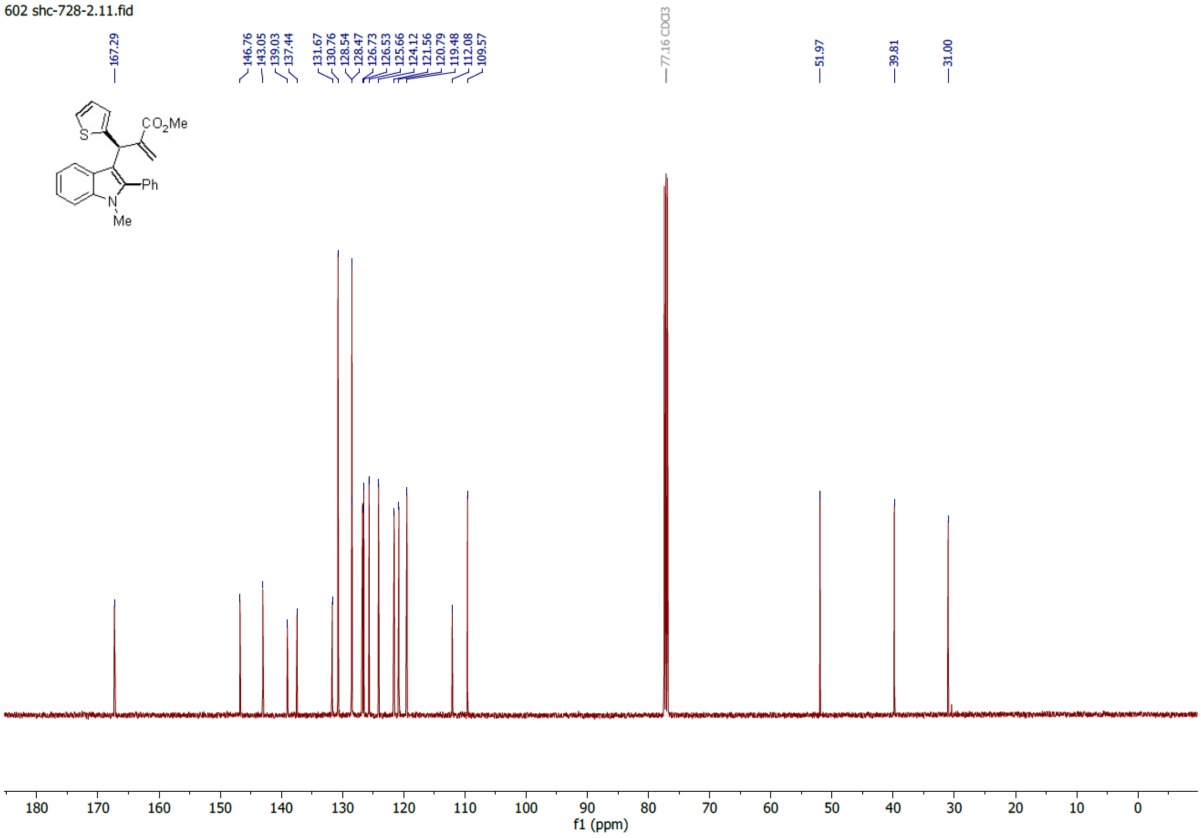

Compound **5w**

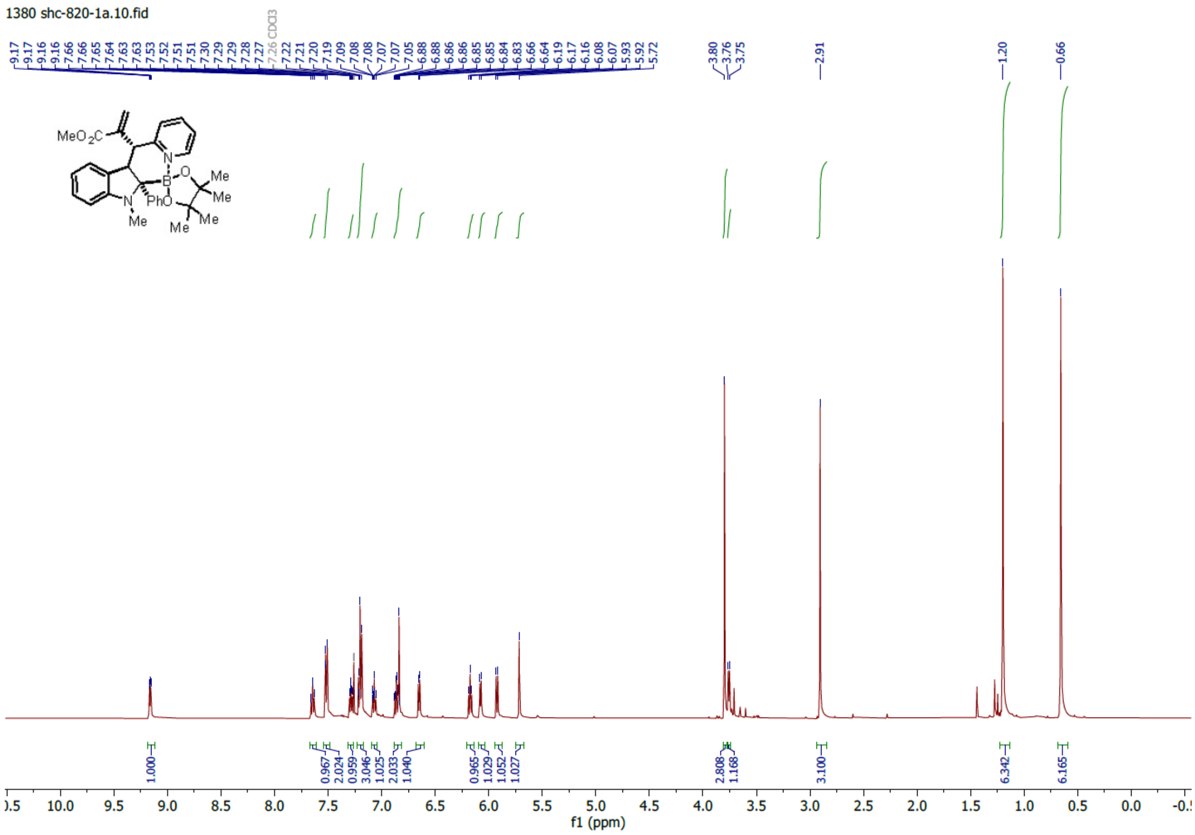

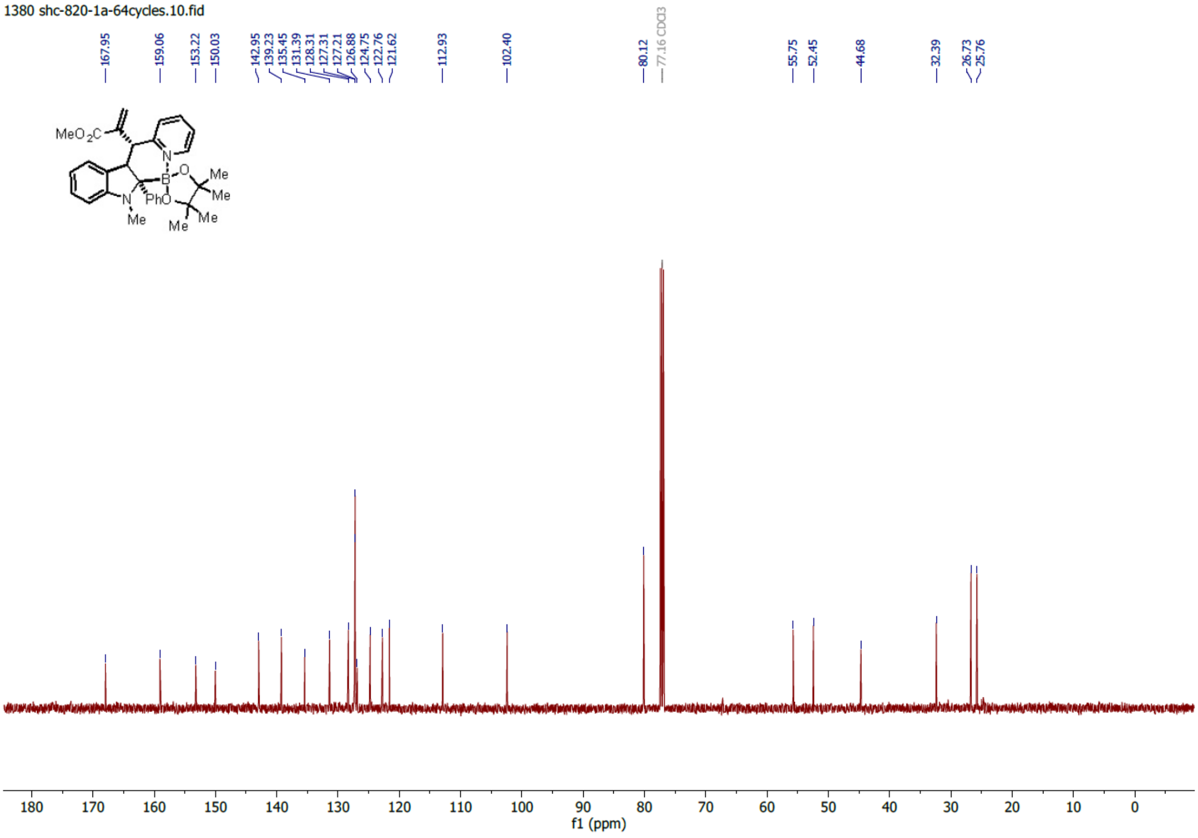

Compound **5x**

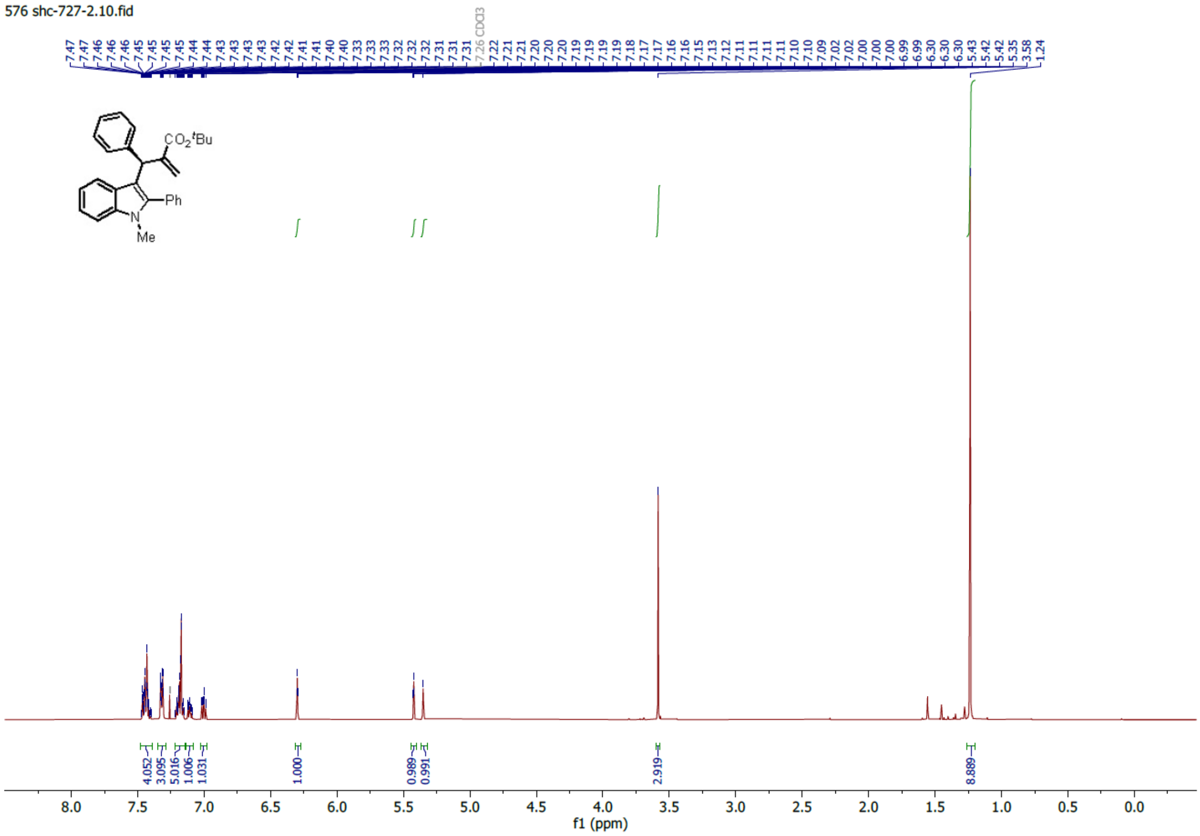

576 shc-727-2.10.fid
7.47 7.47 7.46 7.46 7.45 7.45 7.45 7.45 7.44 7.44 7.43 7.43 7.43 7.43 7.42 7.42 7.41 7.41 7.40 7.40 7.33 7.33 7.33 7.32 7.32 7.32 7.31 7.31 7.26 CDCl3 7.22 7.21 7.21 7.20 7.20 7.20 7.19 7.19 7.19 7.18 7.17 7.17 7.16 7.16 7.15 7.13 7.12 7.11 7.11 7.11 7.11 7.10 7.09 7.02 7.02 7.00 7.00 7.00 6.99 6.99 6.30 6.30 6.30 5.43 5.42 5.42 5.35 3.58 1.24
CO2tBu
Ph
N
Me
4.052 3.095 5.016 1.006 1.031 1.000 0.989 0.991 2.919 8.889
8.0 7.5 7.0 6.5 6.0 5.5 5.0 4.5 4.0 3.5 3.0 2.5 2.0 1.5 1.0 0.5 0.0
f1 (ppm)

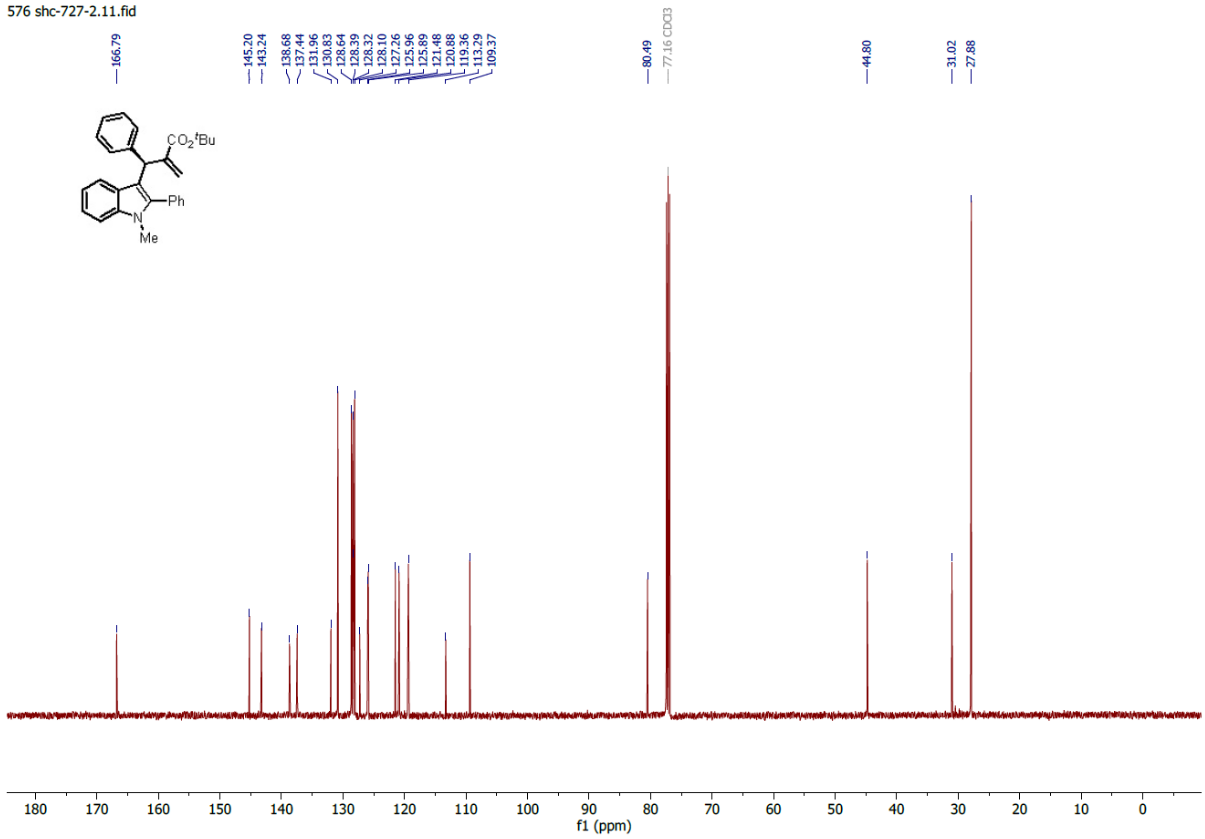

576 shc-727-2.11.fid
166.79 145.20 143.24 138.68 137.44 131.96 130.83 128.64 128.39 128.32 128.10 127.26 125.96 125.89 121.48 120.88 119.36 113.29 109.37 80.49 77.16 CDCl3 44.80 31.02 27.88
CO2tBu
Ph
N
Me
180 170 160 150 140 130 120 110 100 90 80 70 60 50 40 30 20 10 0
f1 (ppm)

Compound **5y**

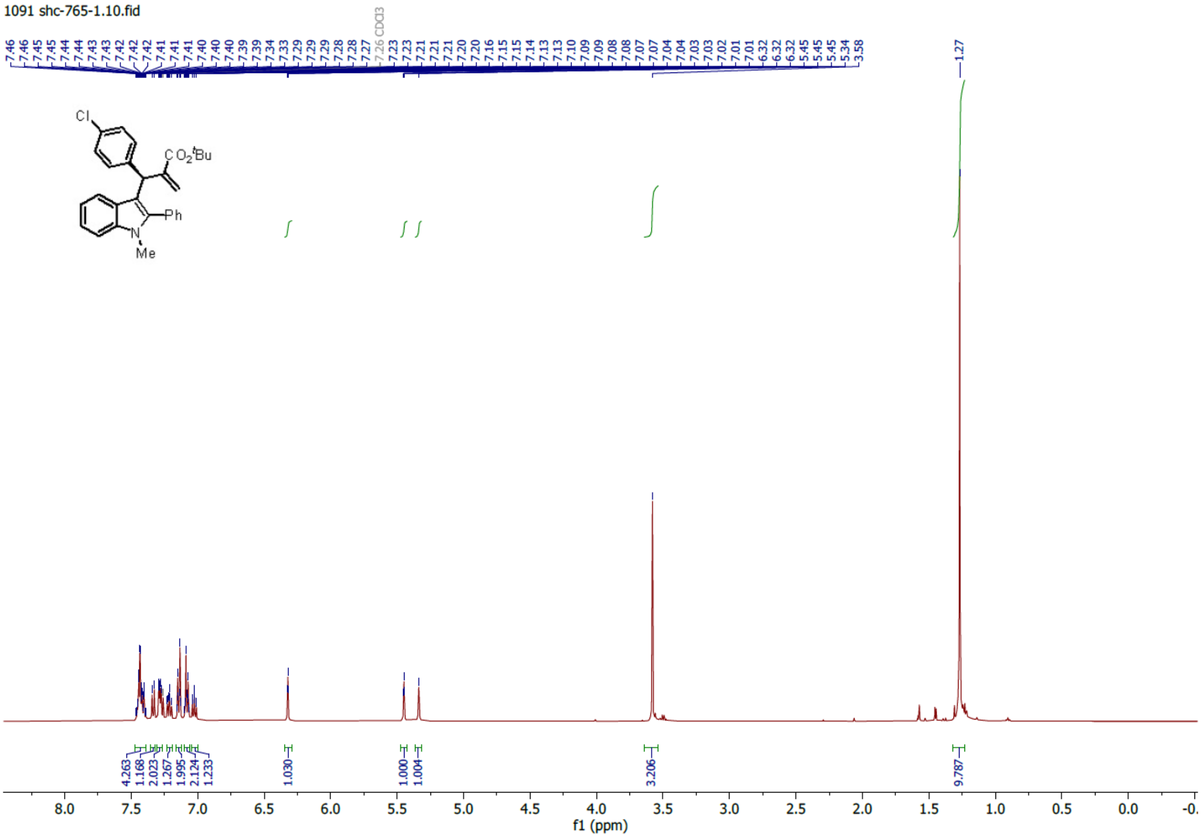

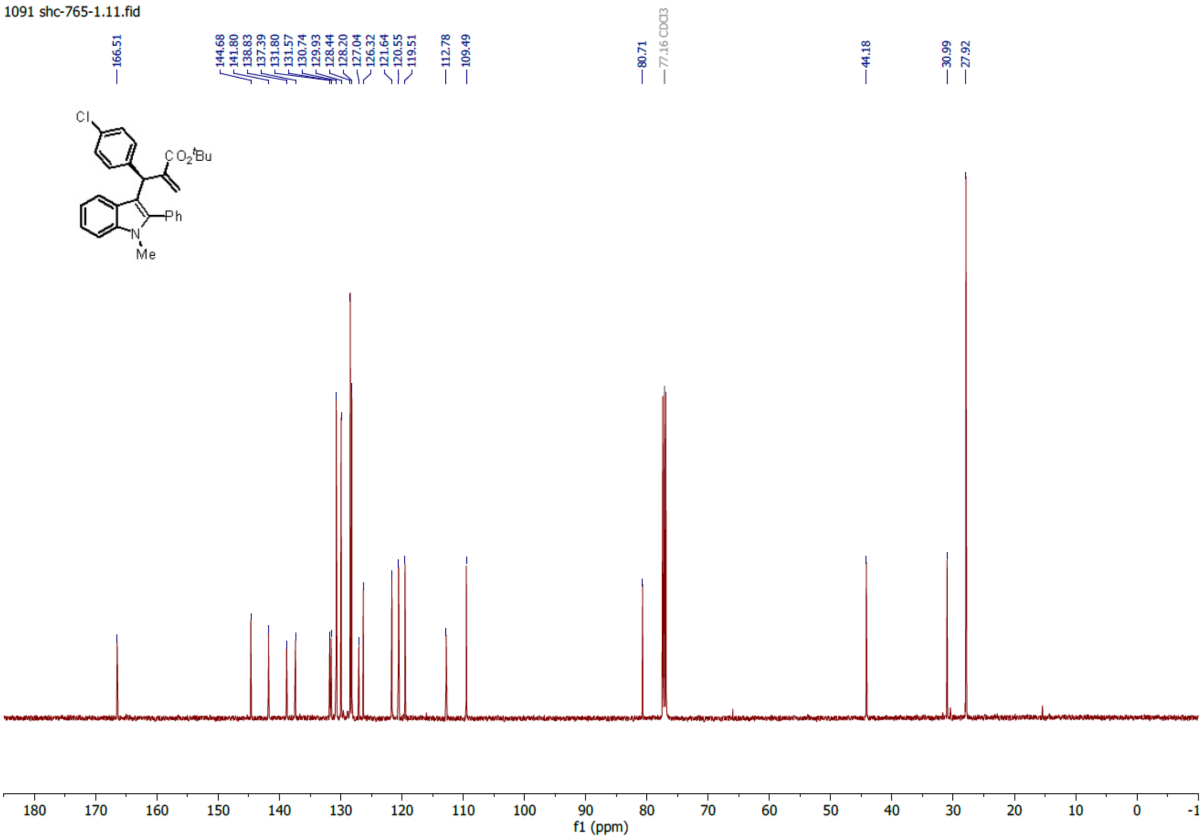

Compound **5z**

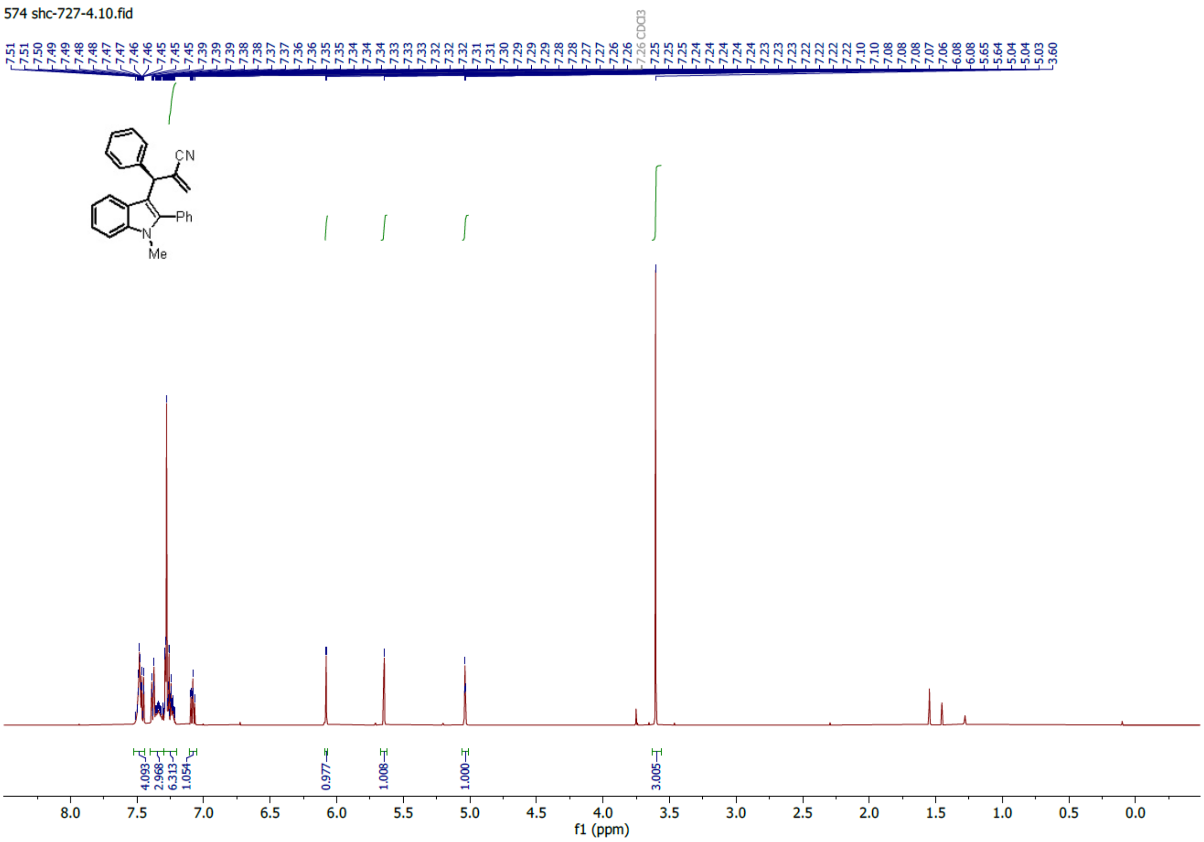
574 shc-727-4.10.fid
7.51 7.51 7.50 7.49 7.49 7.48 7.48 7.47 7.47 7.46 7.46 7.45 7.45 7.45 7.39 7.39 7.39 7.38 7.38 7.37 7.37 7.36 7.36 7.35 7.35 7.34 7.34 7.33 7.33 7.33 7.32 7.32 7.31 7.31 7.30 7.29 7.29 7.28 7.27 7.27 7.26 7.26 CDCl3 7.25 7.25 7.25 7.24 7.24 7.24 7.24 7.23 7.23 7.23 7.22 7.22 7.22 7.10 7.10 7.08 7.08 7.07 7.06 6.08 5.65 5.64 5.04 5.04 5.03 3.60
CN
Ph
N
Me
4.093
2.968
6.313
1.054
0.977
1.008
1.000
3.005
f1 (ppm)

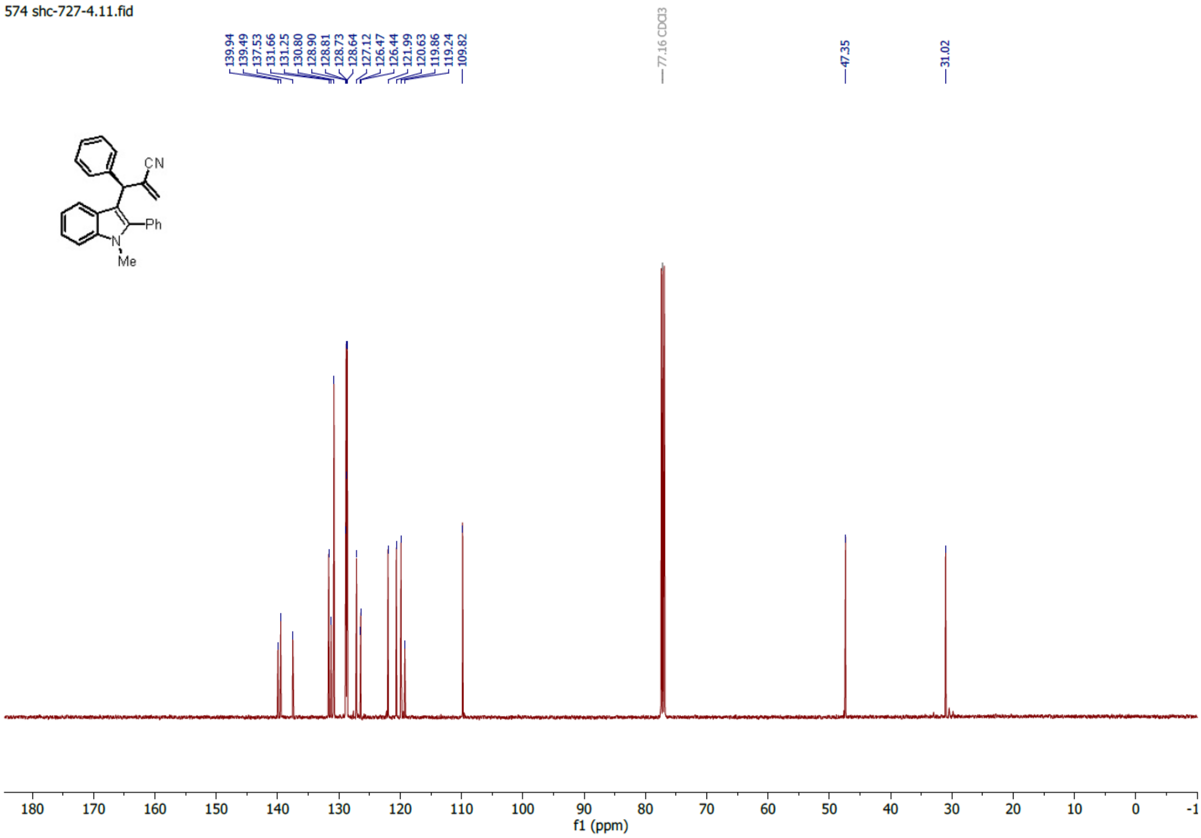
574 shc-727-4.11.fid
139.94 139.49 137.53 131.66 131.25 130.80 128.90 128.81 128.73 128.64 127.12 126.47 126.44 121.99 120.63 119.86 119.24 109.82
77.16 CDCl3
47.35
31.02
CN
Ph
N
Me
f1 (ppm)

Compound **5aa**

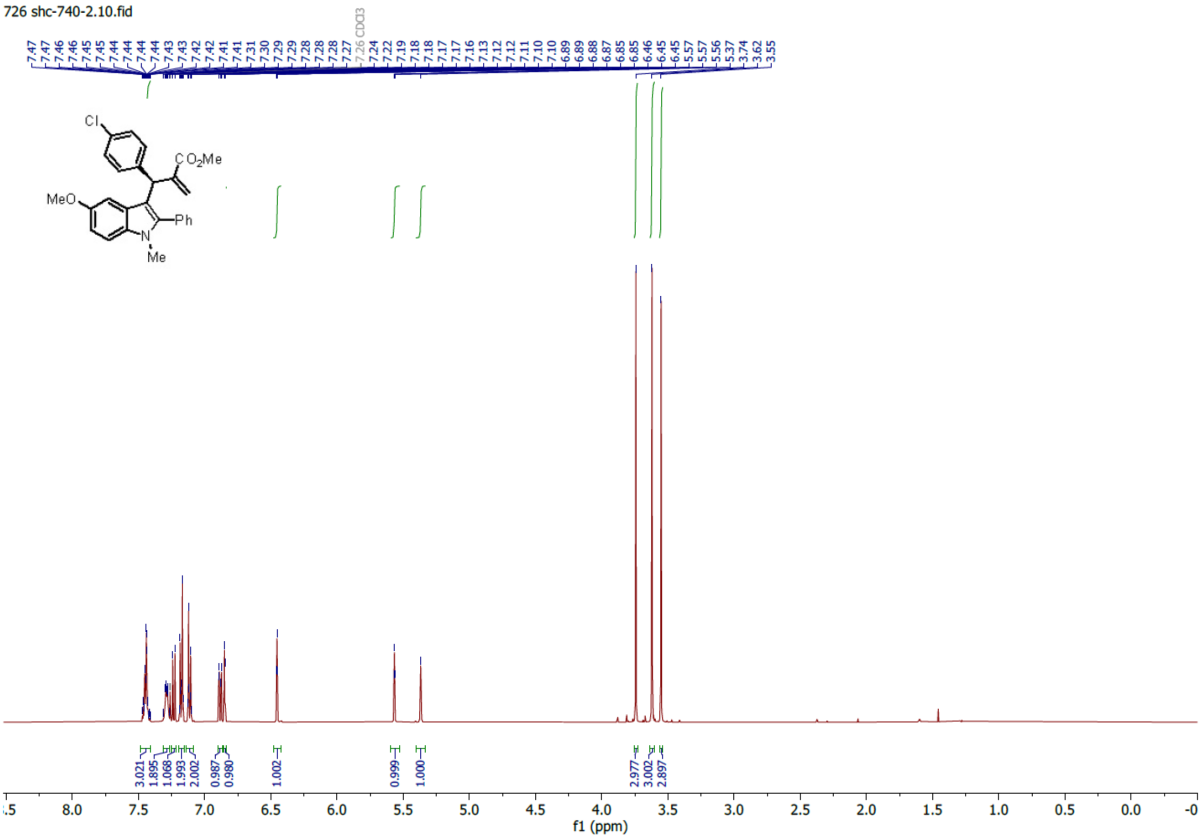

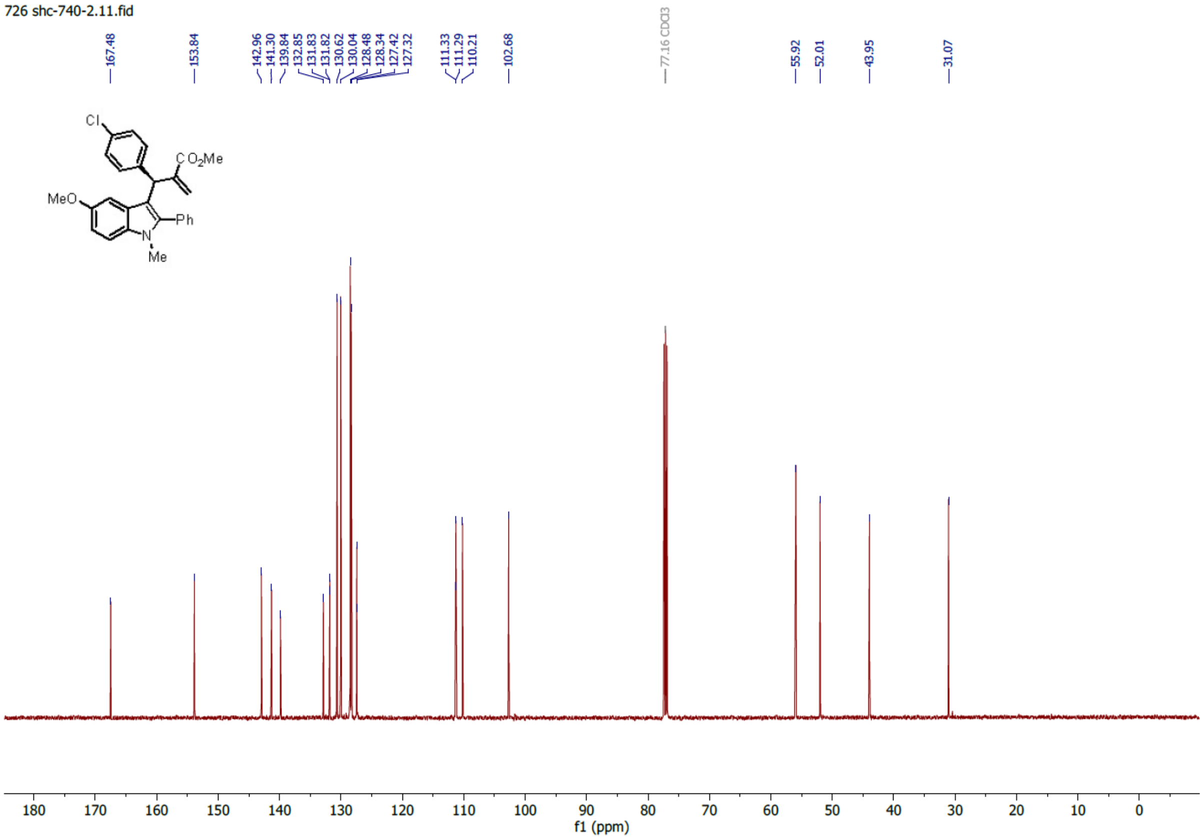

Compound **5bb**

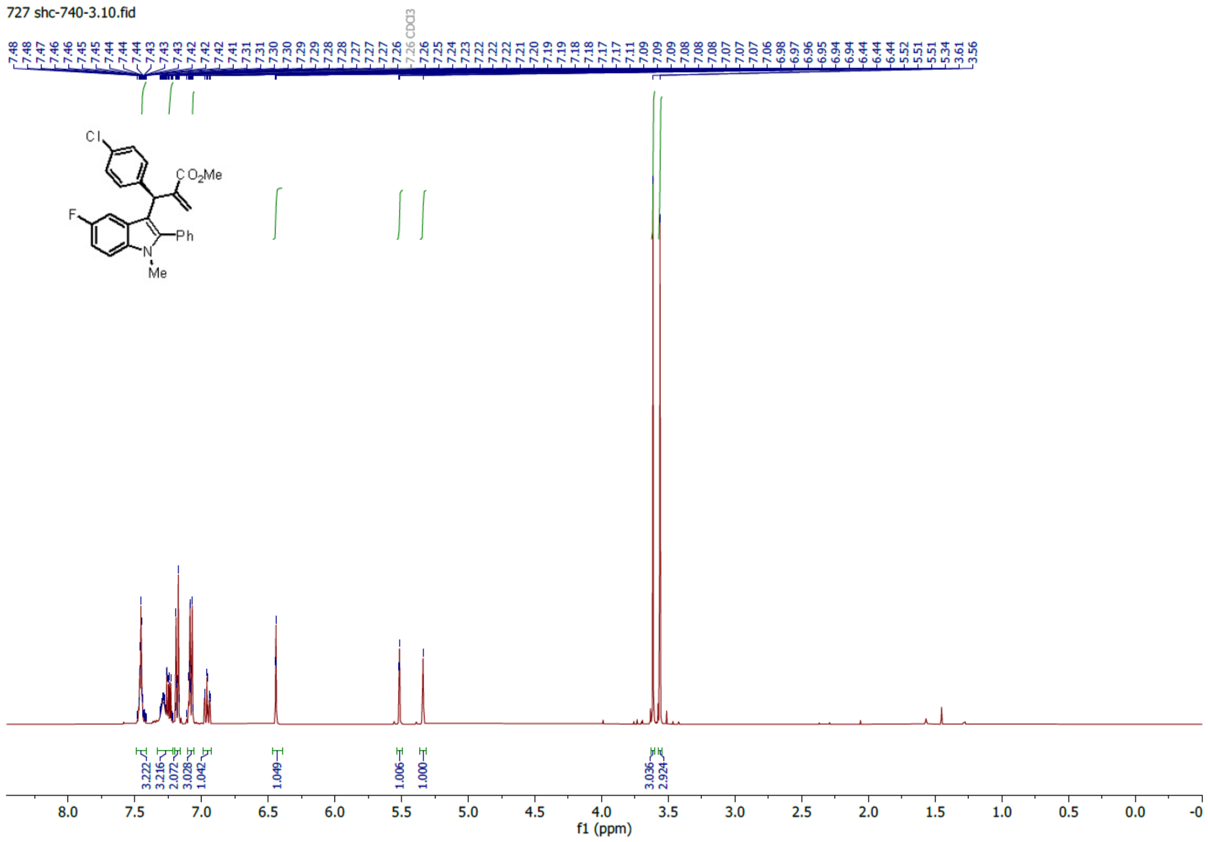

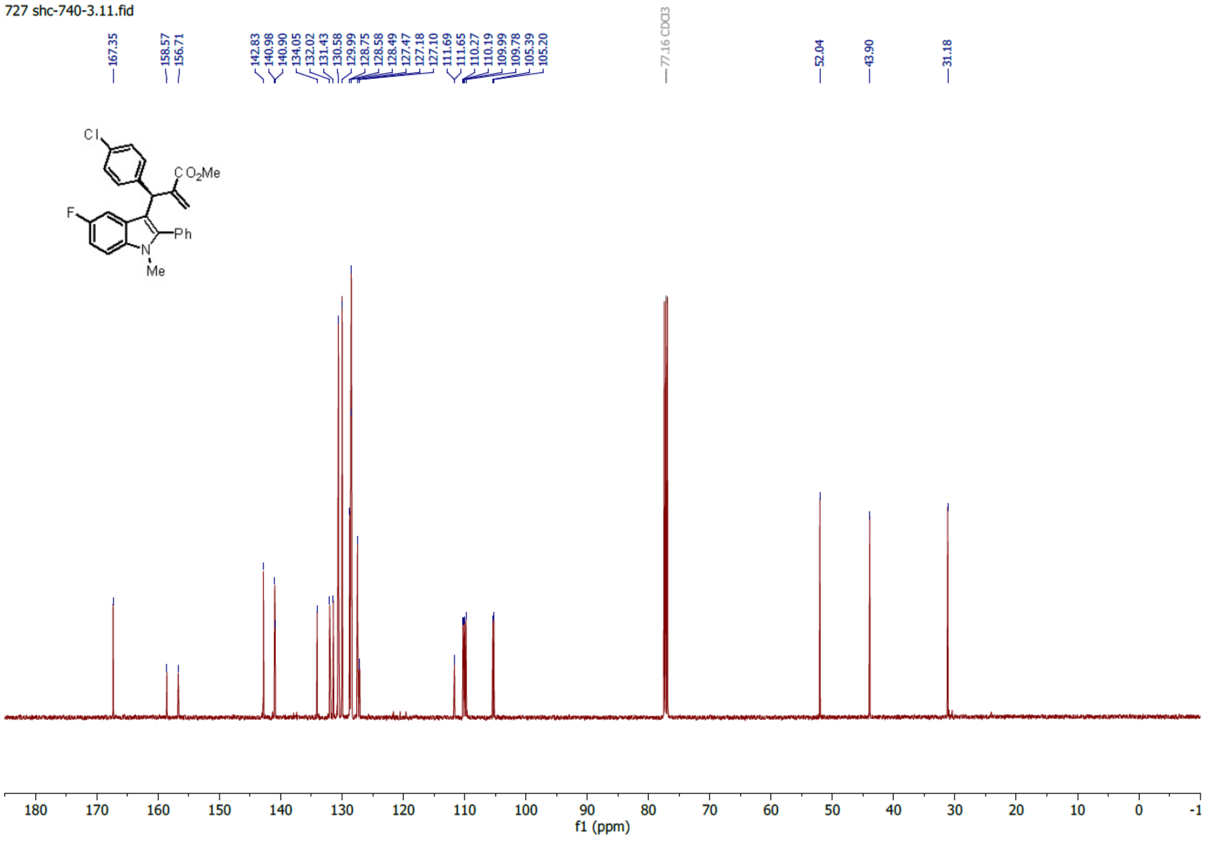

Compound **5cc**

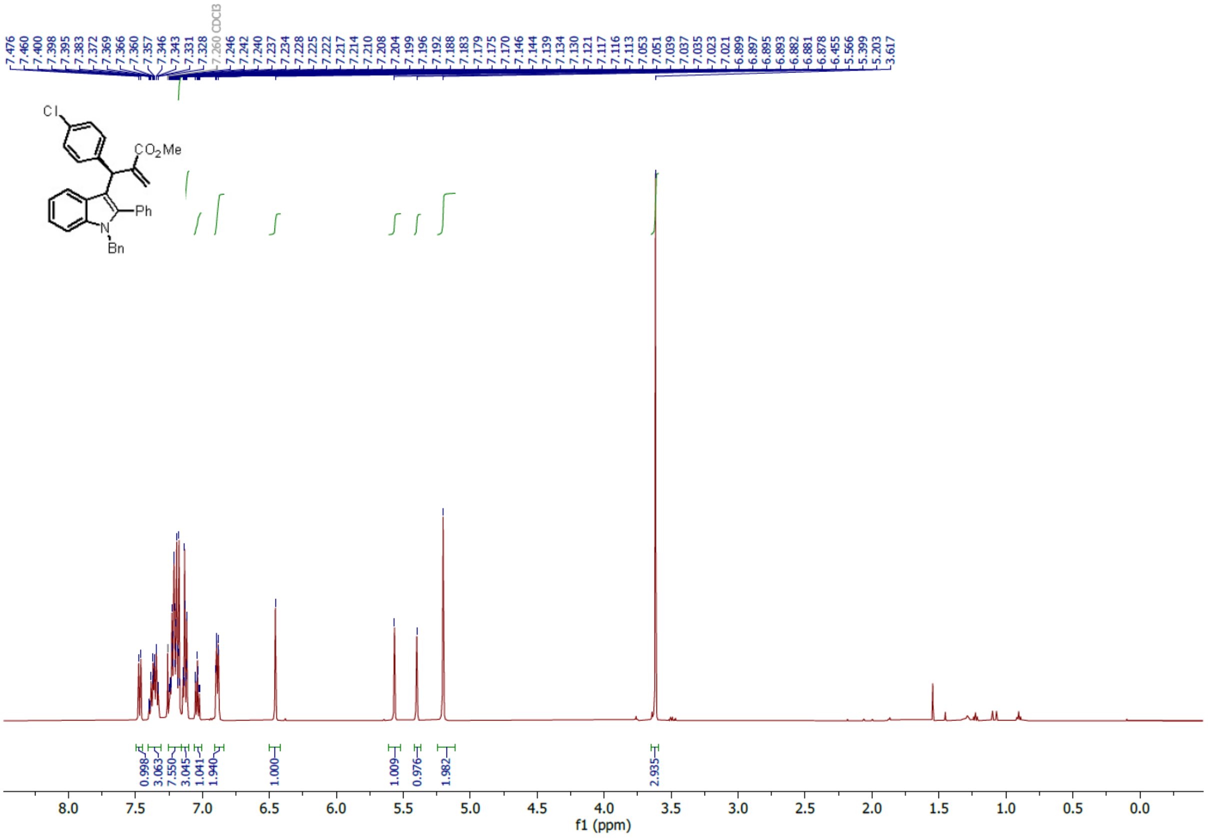

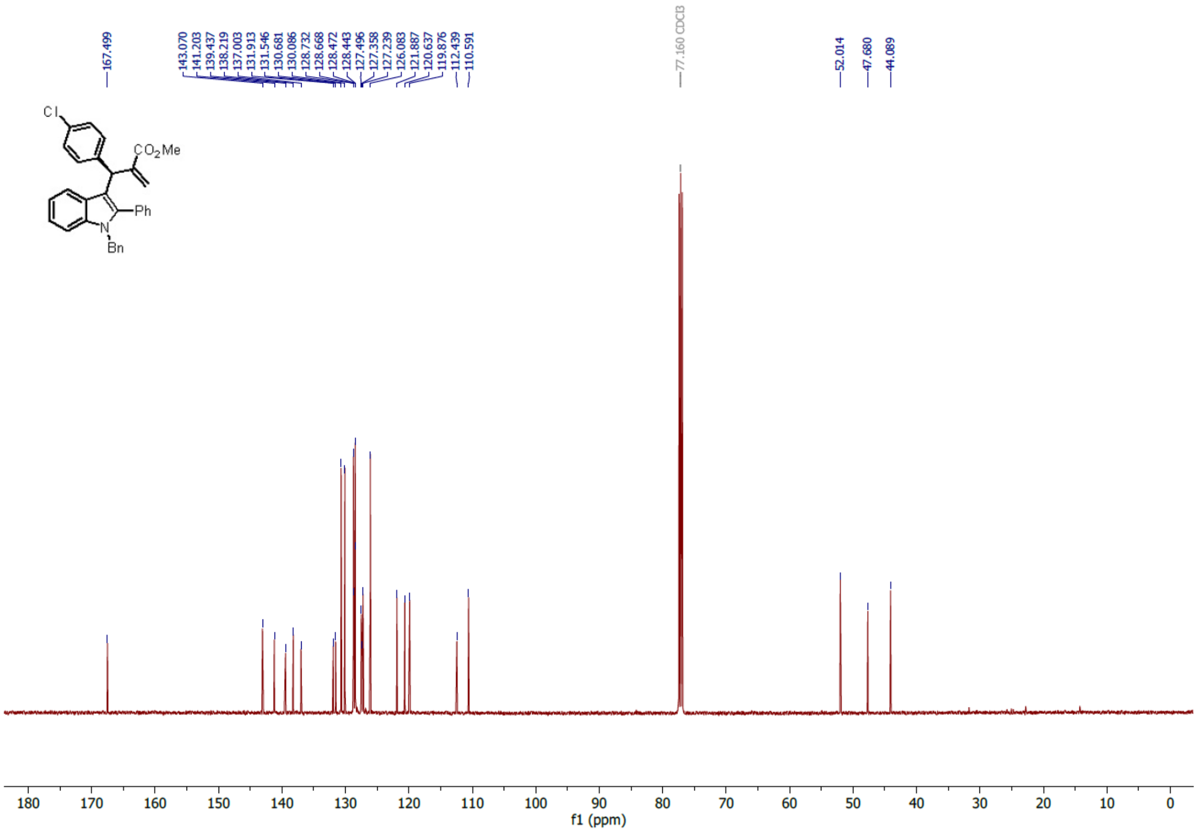

Compound **5dd**

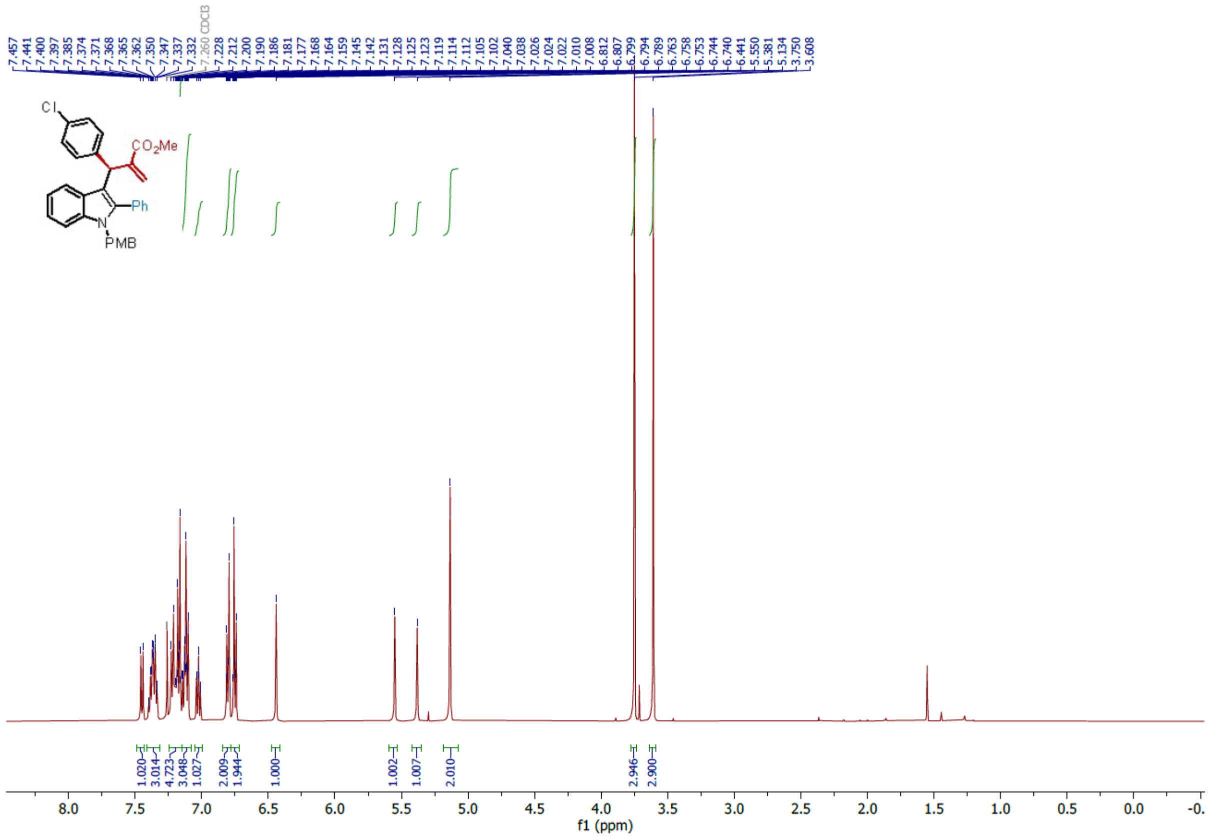

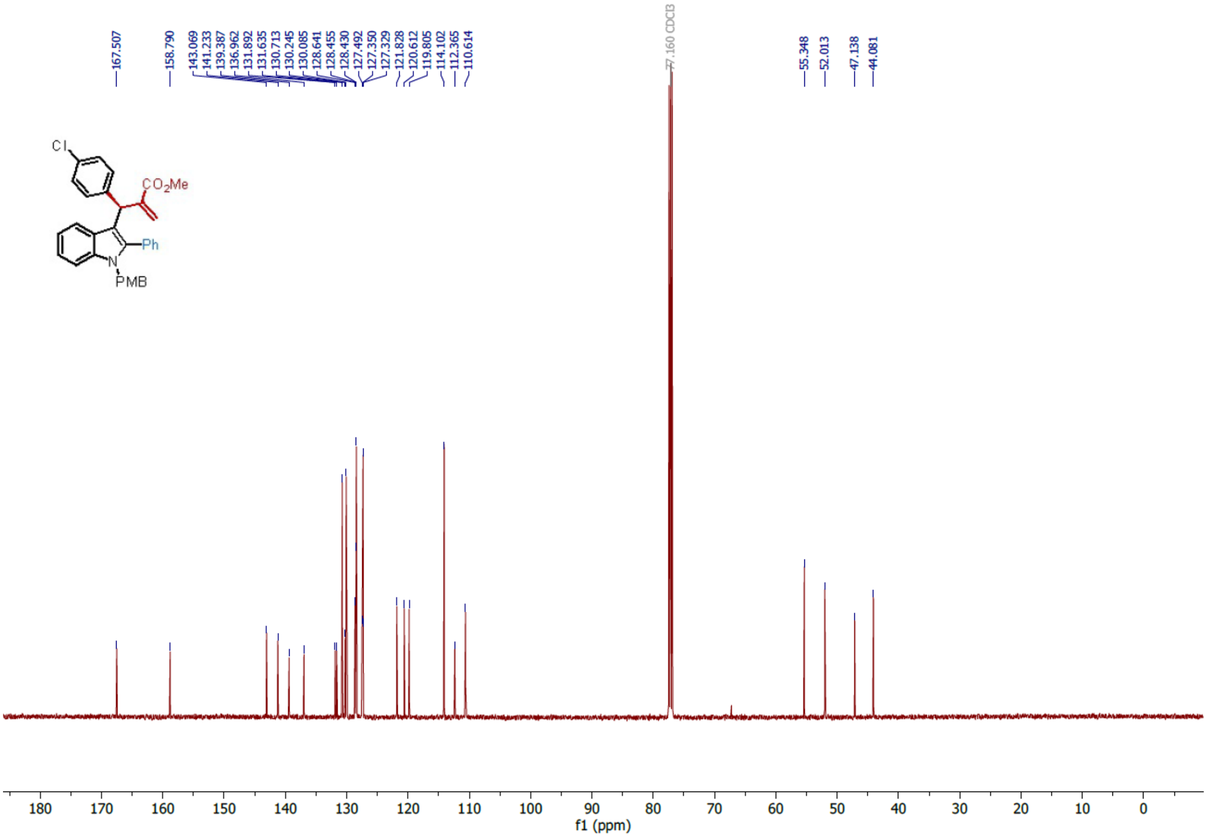

Compound **5ee**

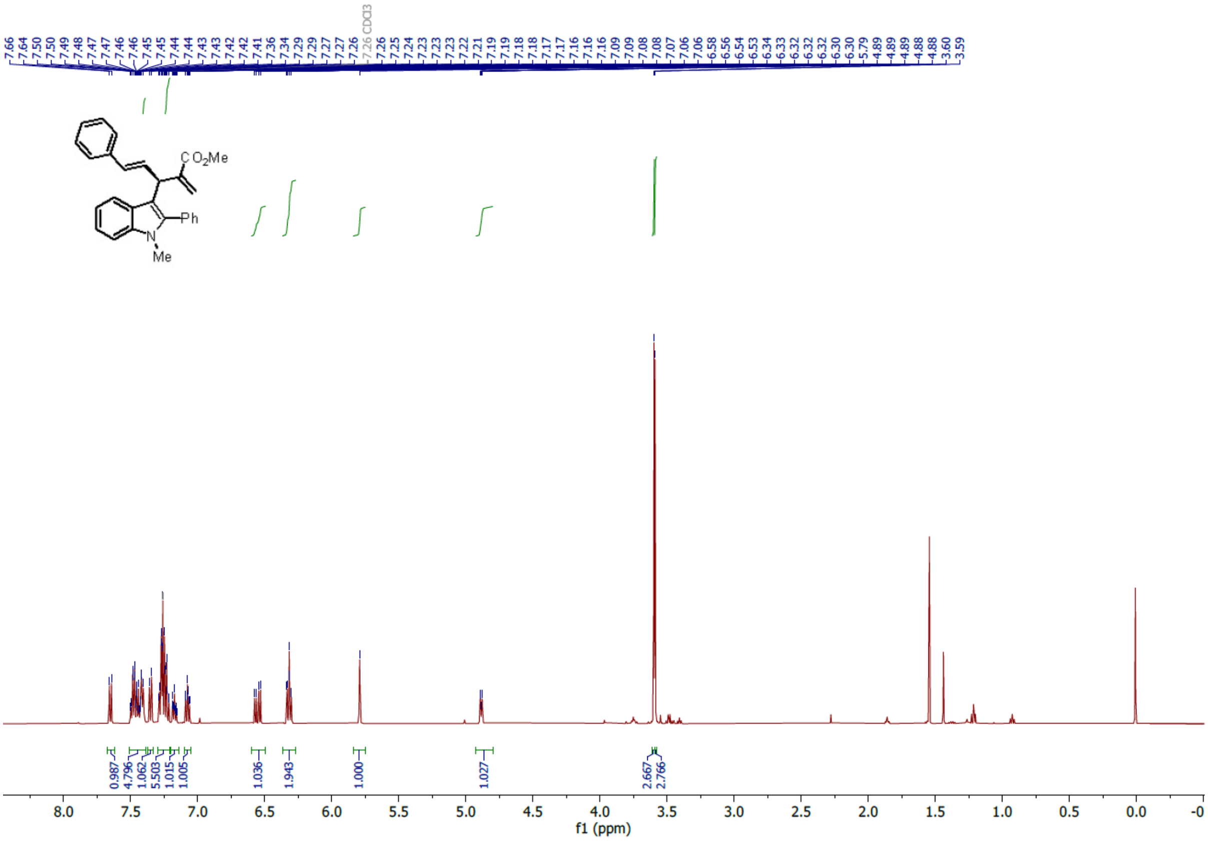

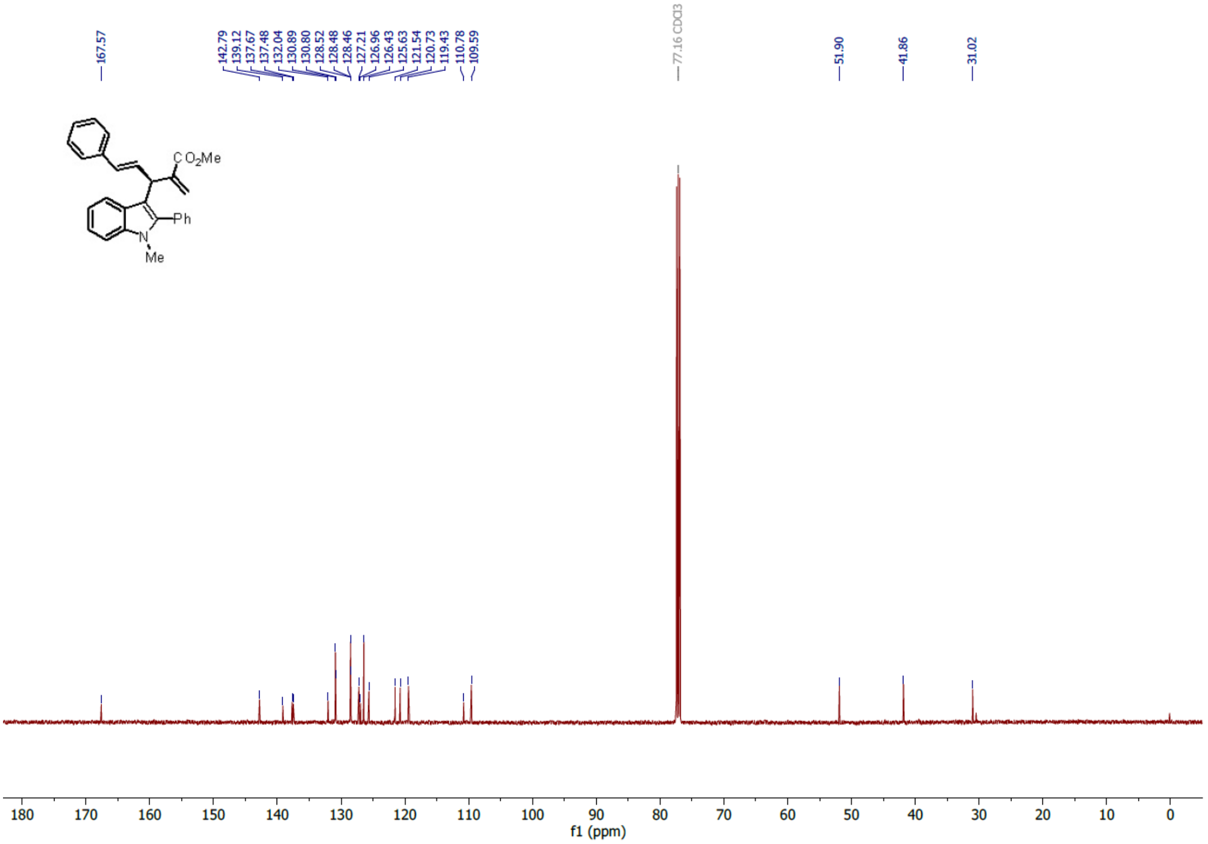

Compound **5ee'**

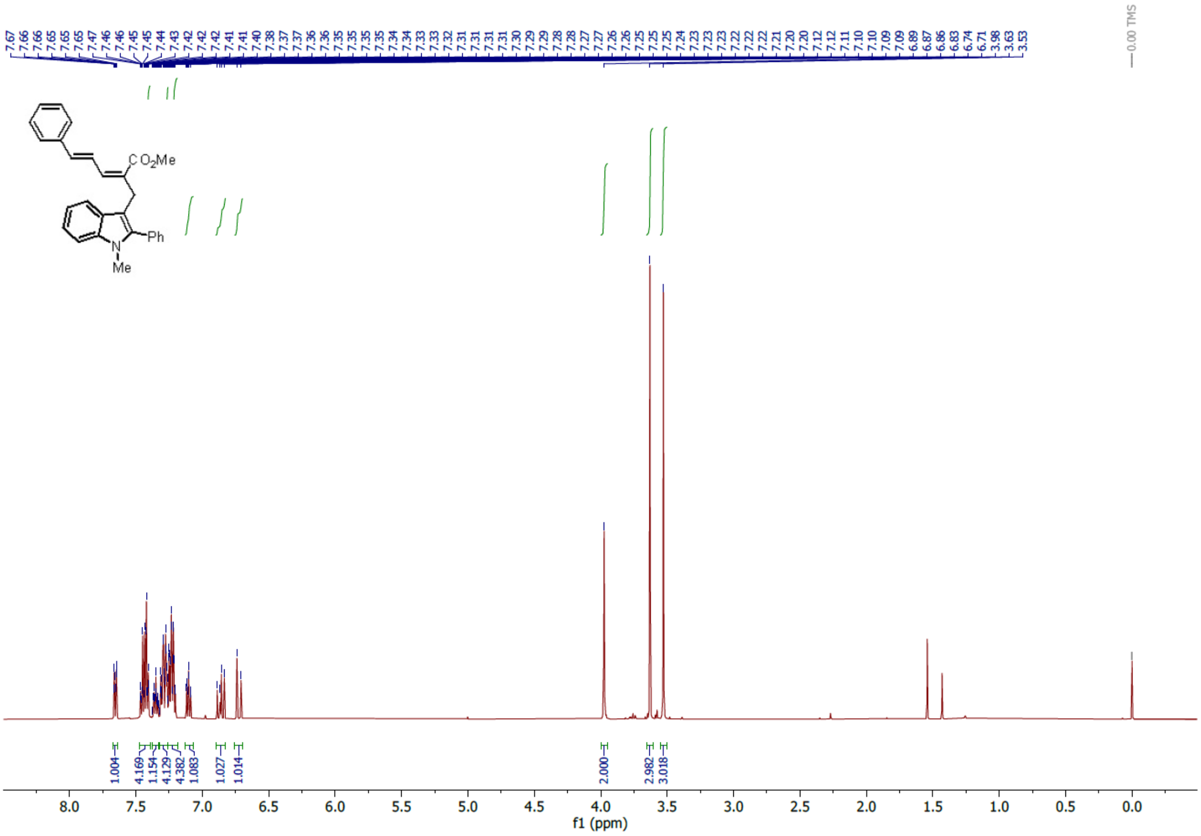

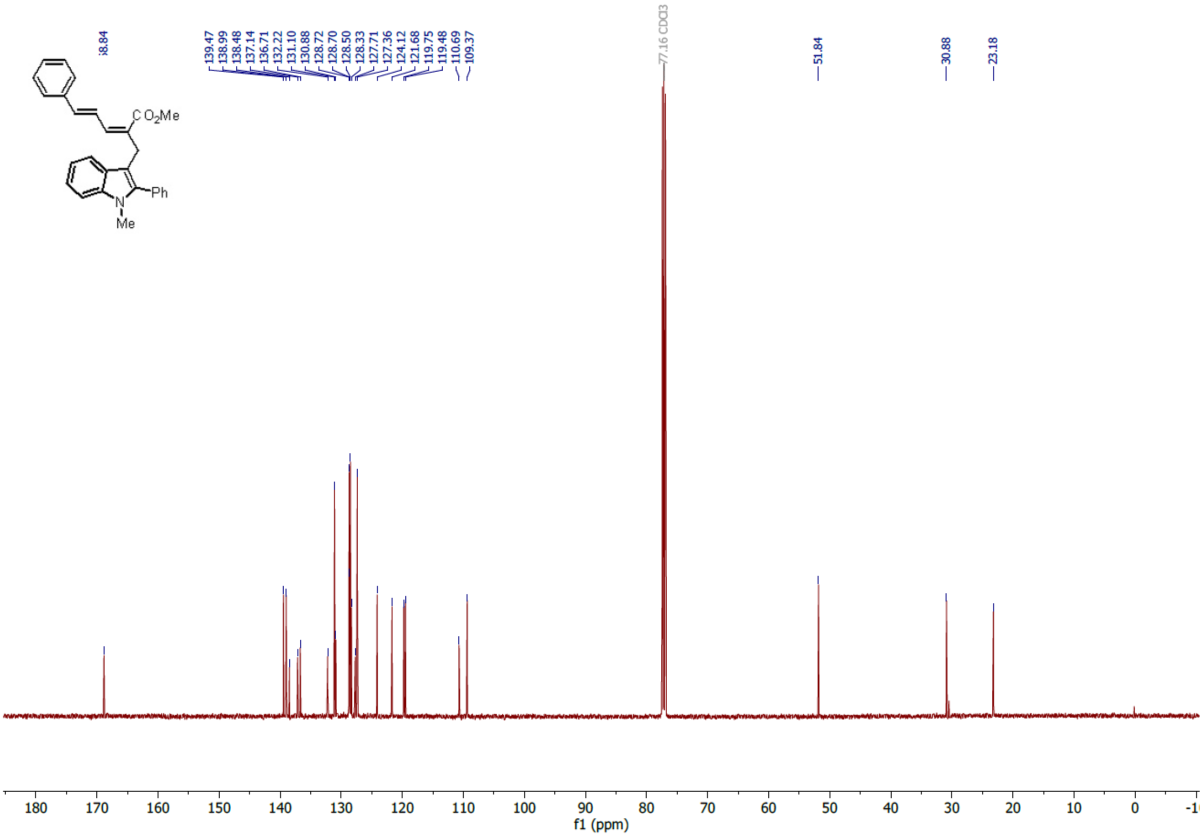

Compound **5ff**

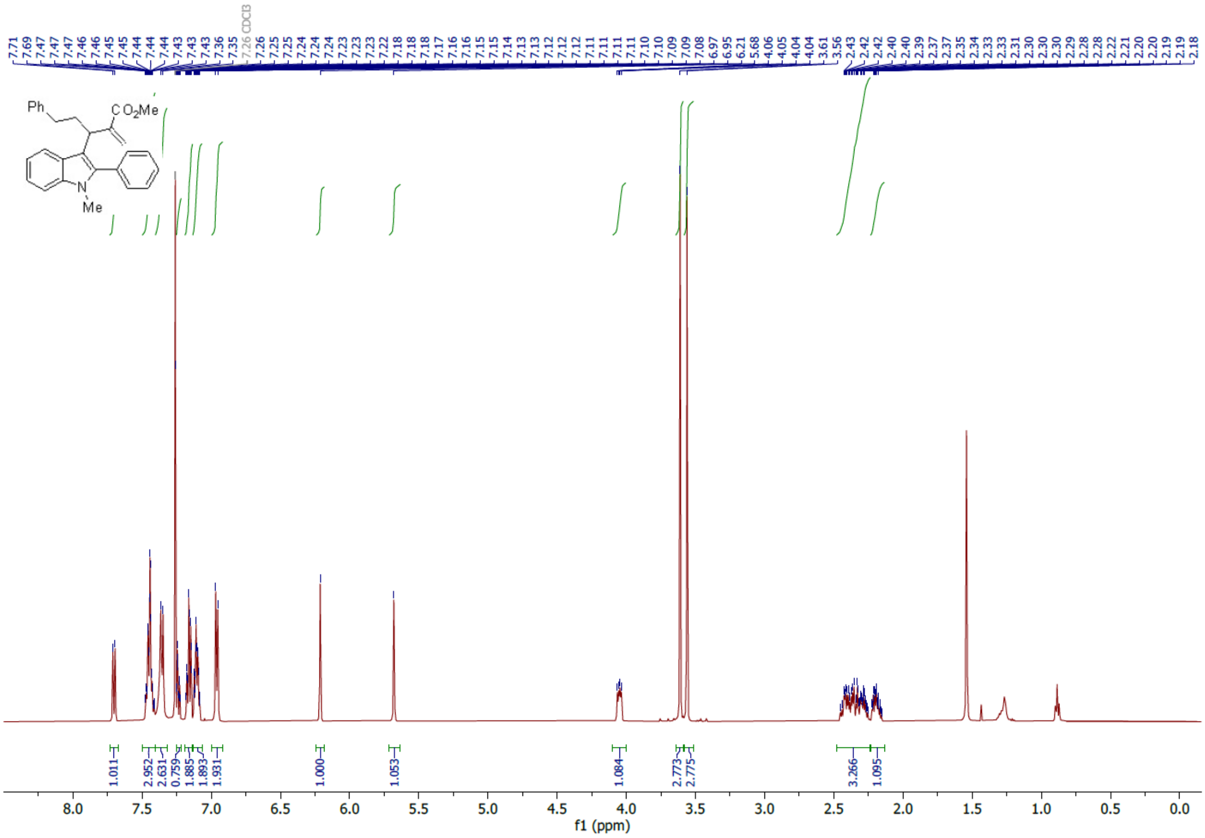

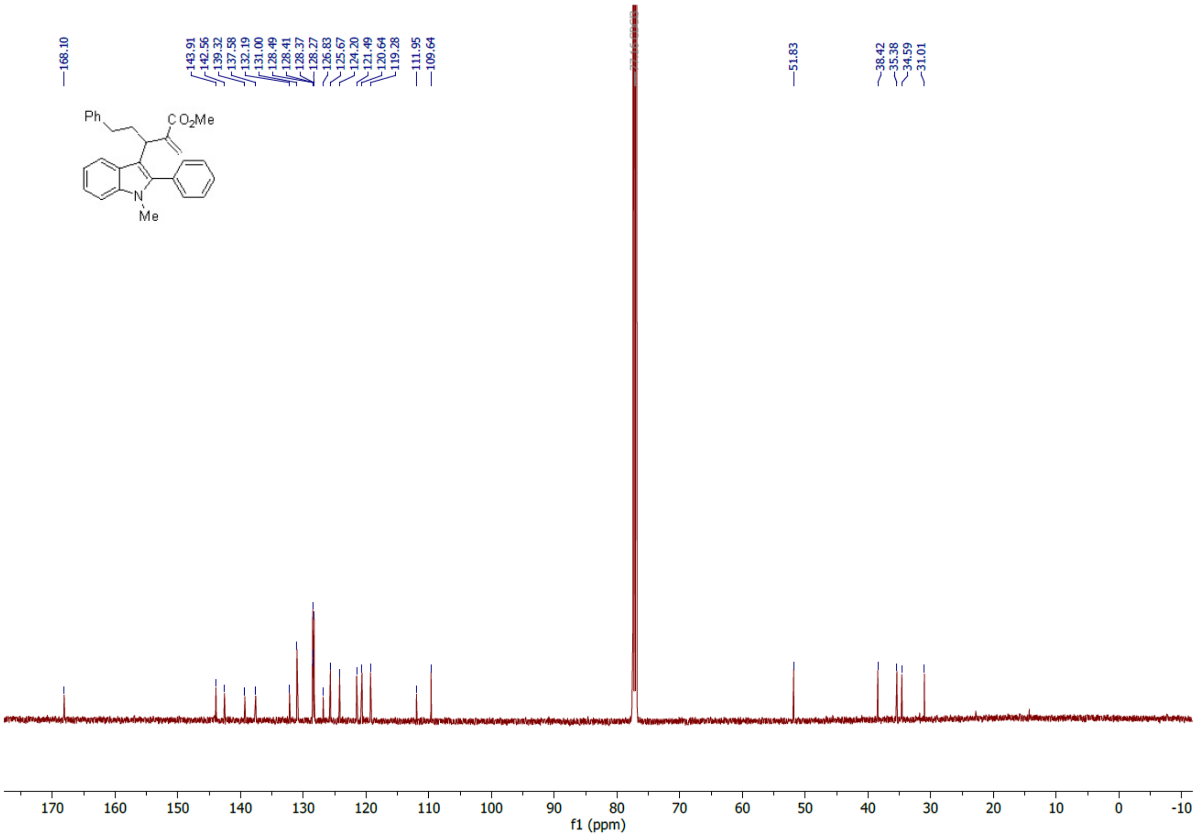

Compound **5ff'**

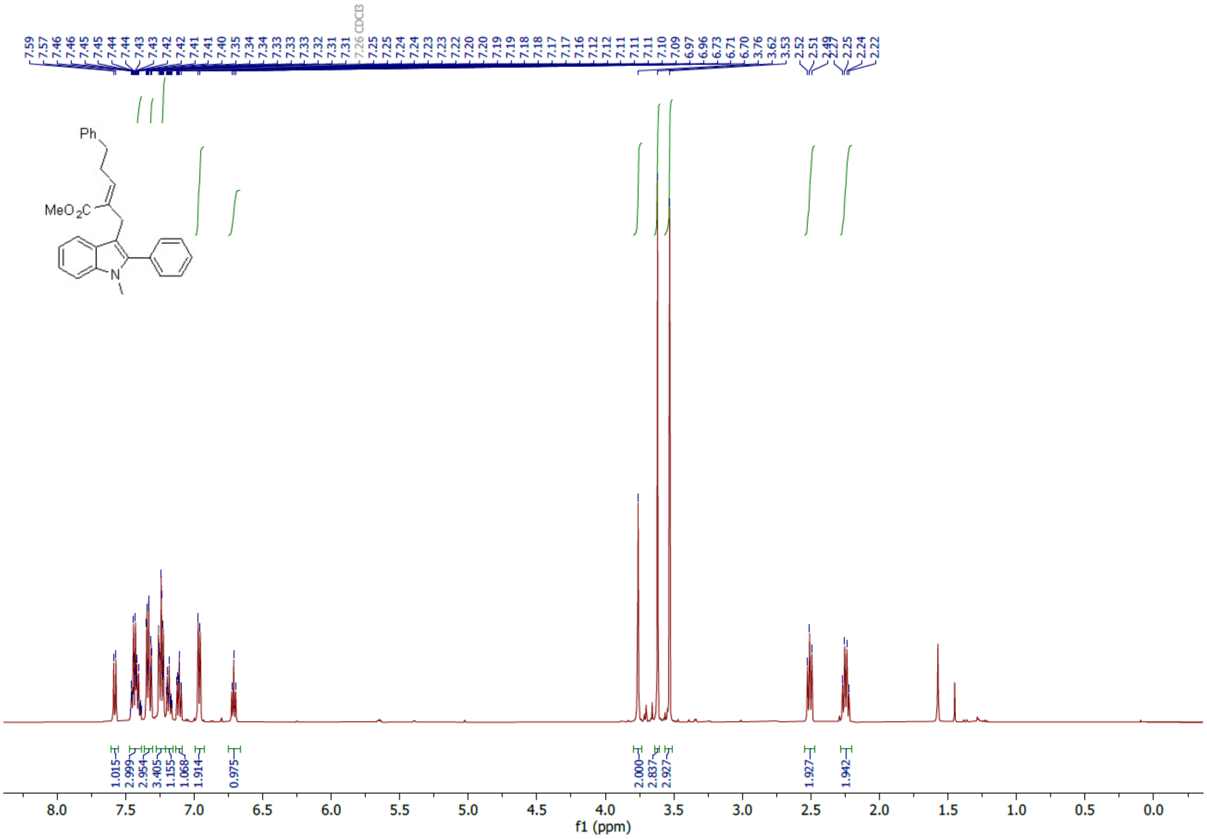

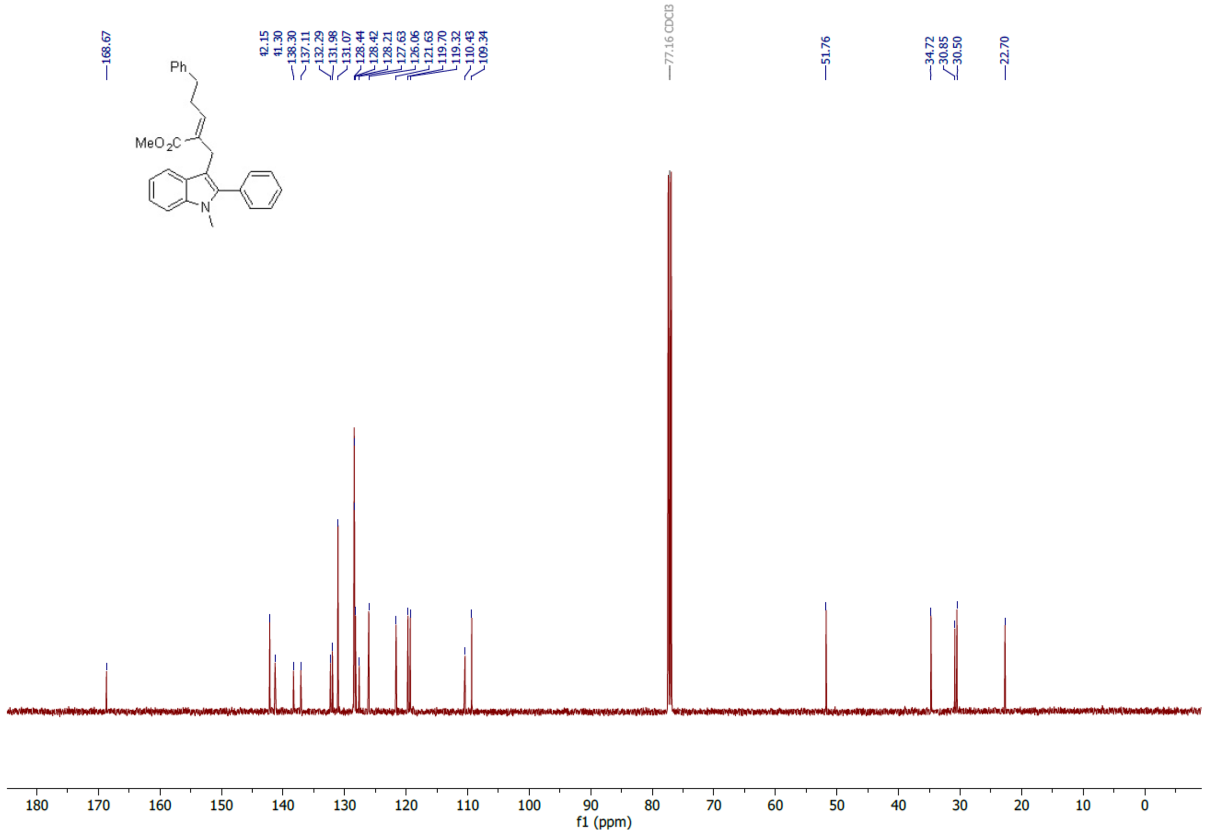

Compound **6**

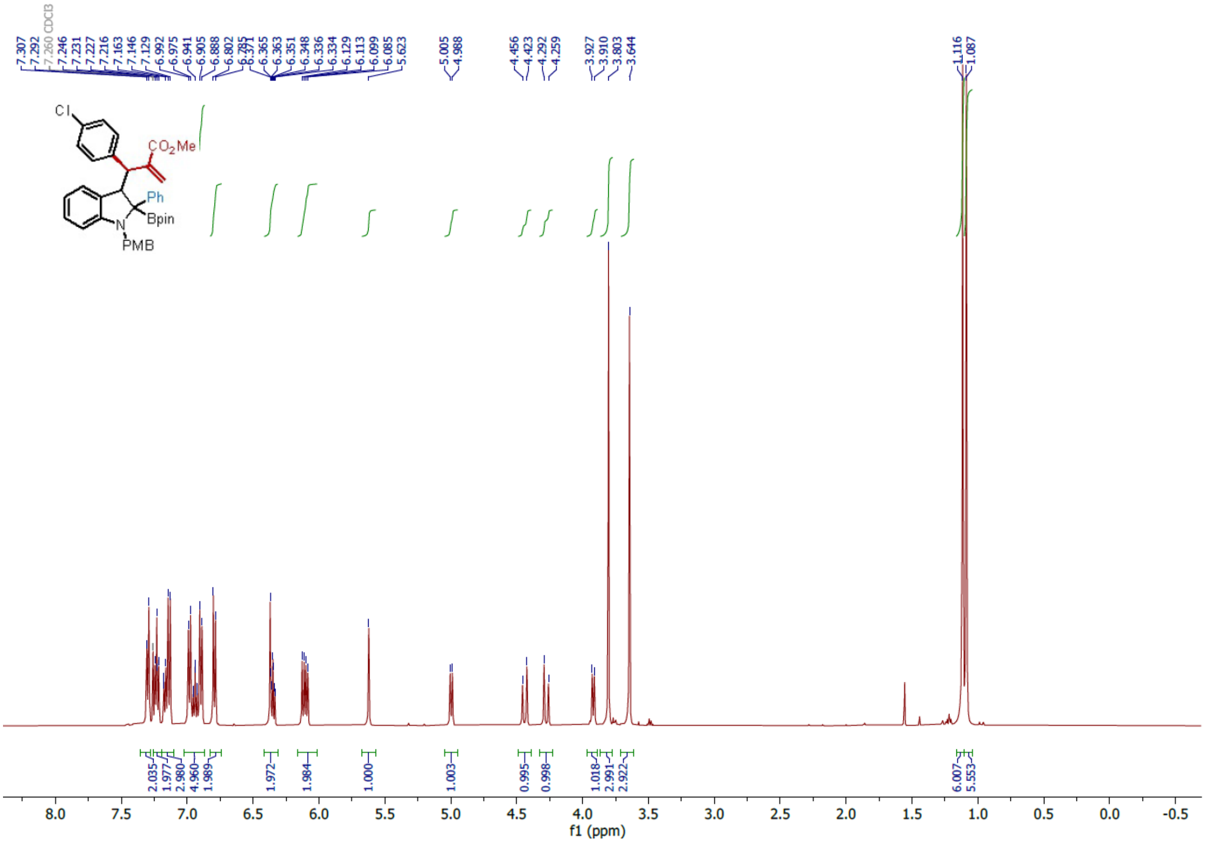

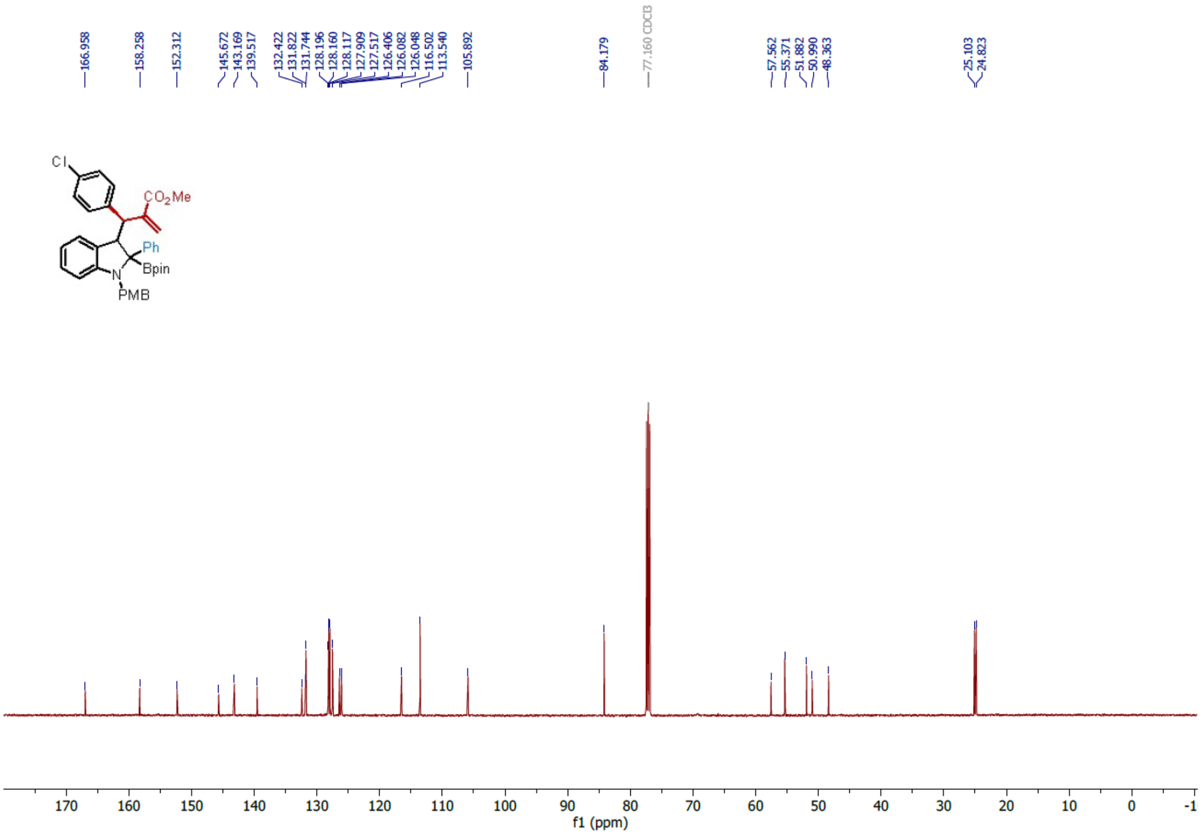

Compound **7**

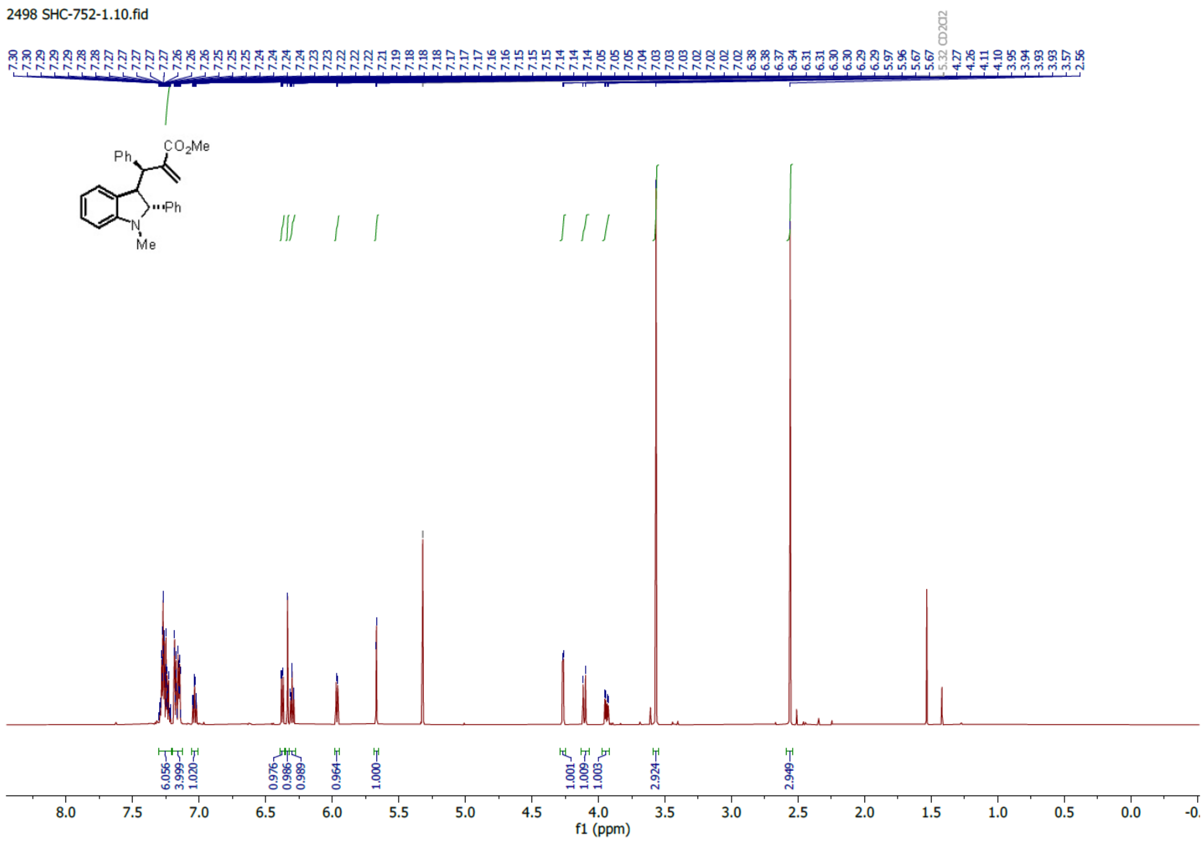
2498 SHC-752-1.10.fid
Ph
CO2Me
Ph
N
Me
f1 (ppm)

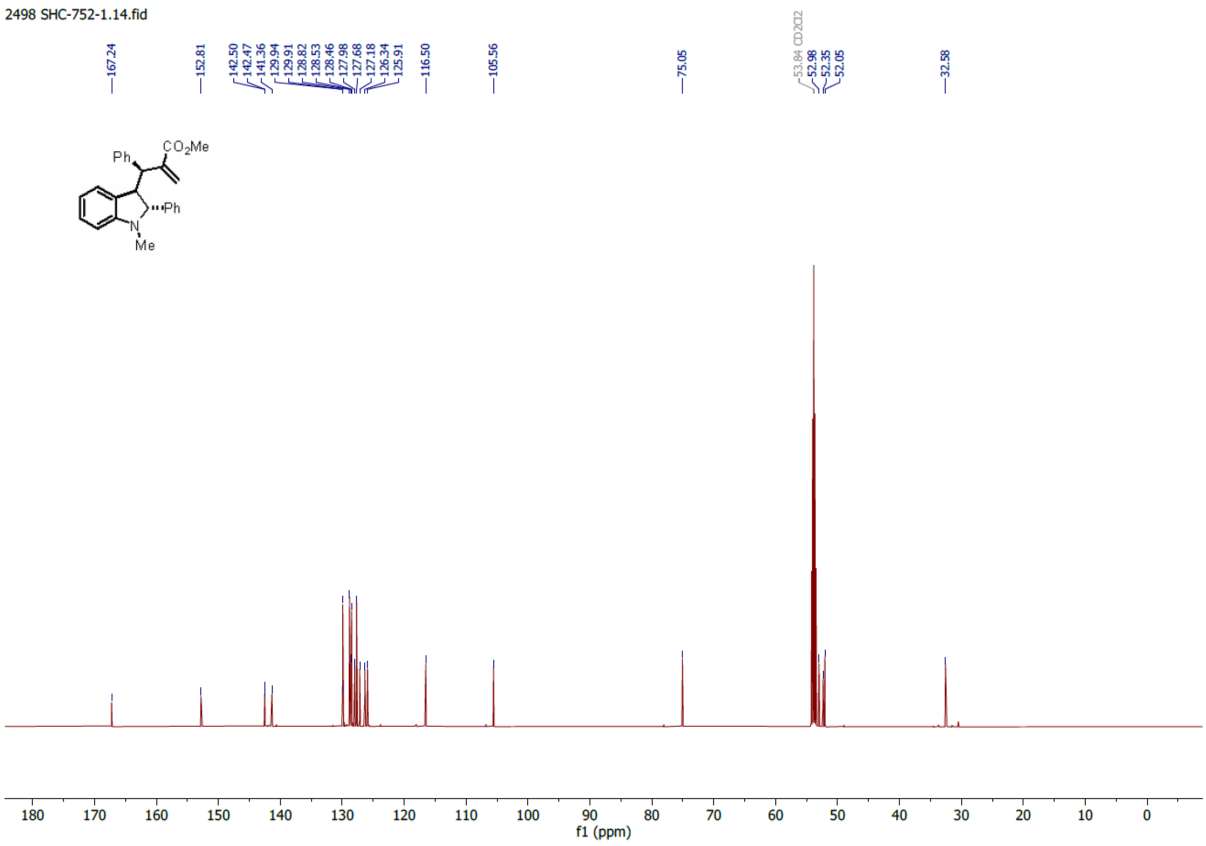
2498 SHC-752-1.14.fid
167.24
152.81
142.50
142.47
141.36
129.94
129.91
128.82
128.53
128.46
127.98
127.68
127.18
126.34
125.91
116.50
105.56
75.05
53.84 CD2Cl2
52.98
52.35
52.05
32.58
Ph
CO2Me
Ph
N
Me
f1 (ppm)

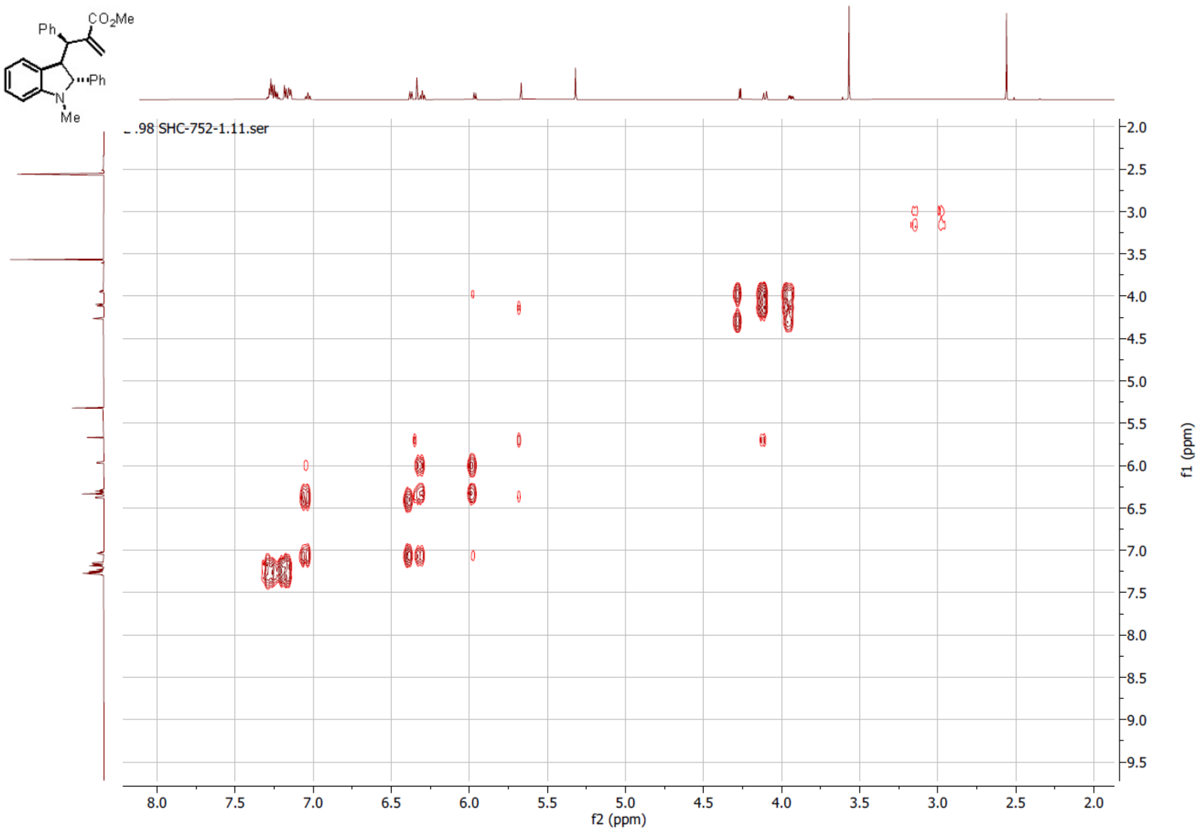

Ph
CO_2Me
Ph
N
Me
SHC-752-1.11.ser
f2 (ppm)
f1 (ppm)

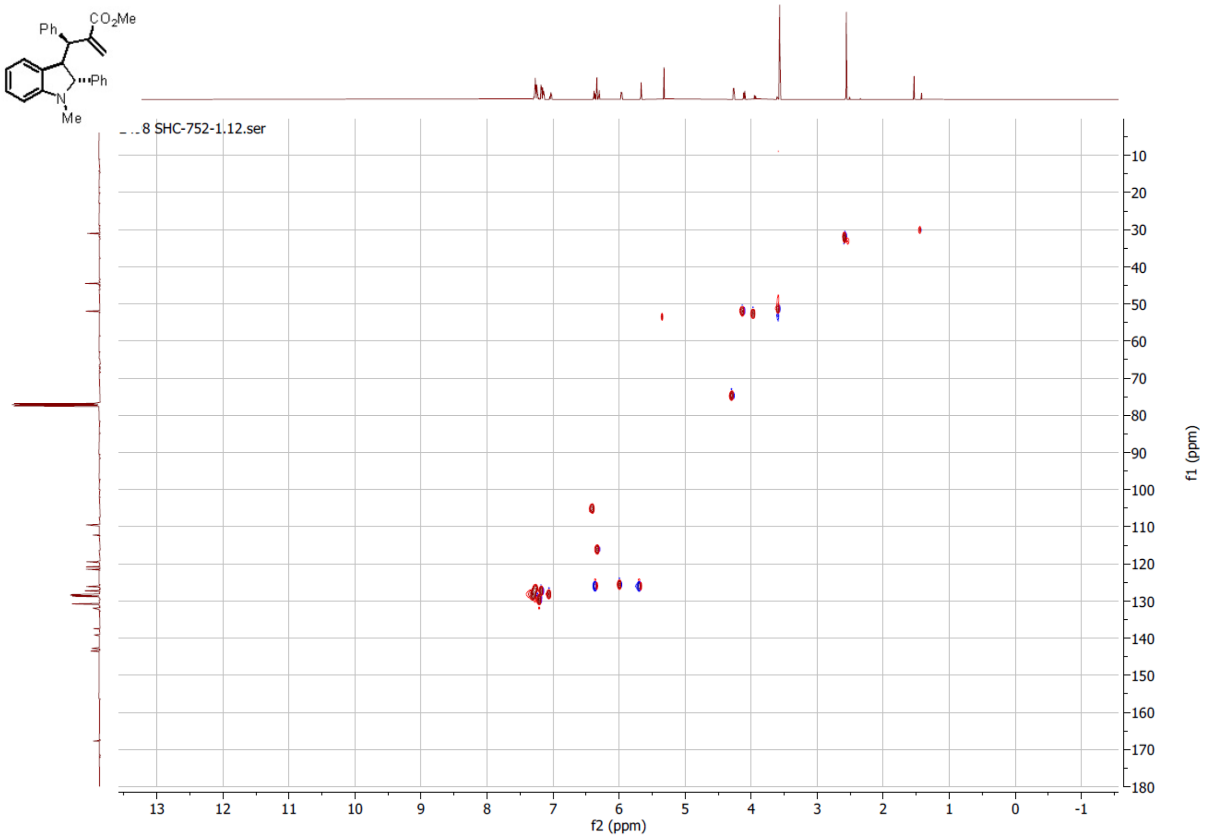

Ph
CO_2Me
Ph
N
Me
SHC-752-1.12.ser
f2 (ppm)
f1 (ppm)

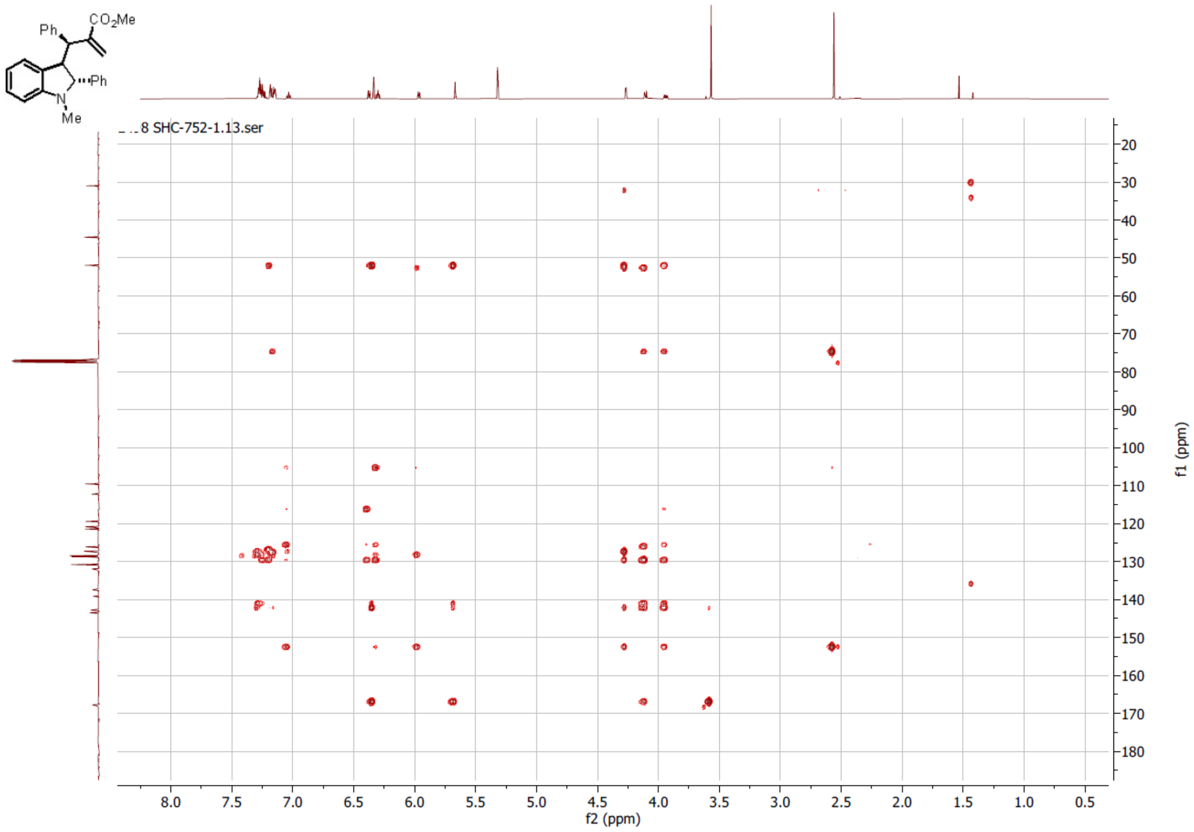

CO2Me
Ph
Ph
N
Me
SHC-752-1.13.ser
f2 (ppm)
f1 (ppm)

Compound **8**

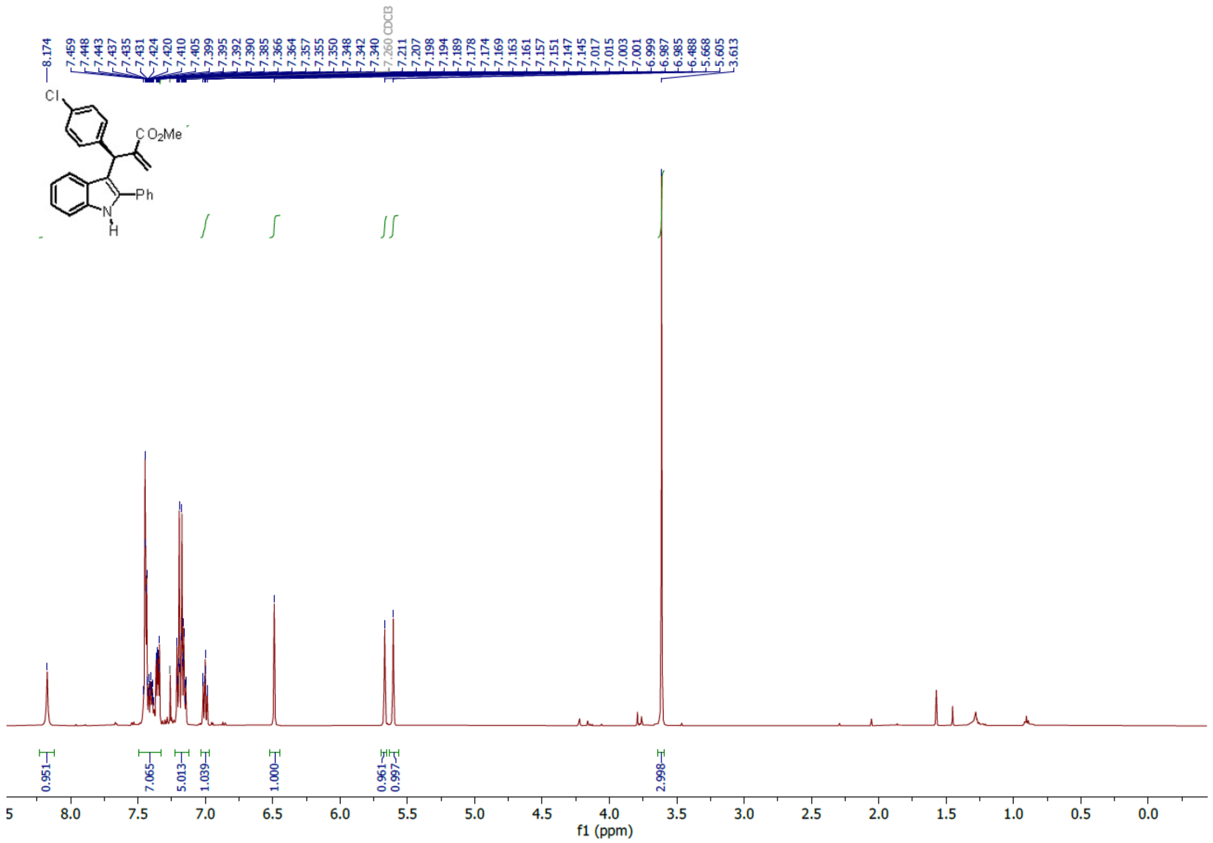

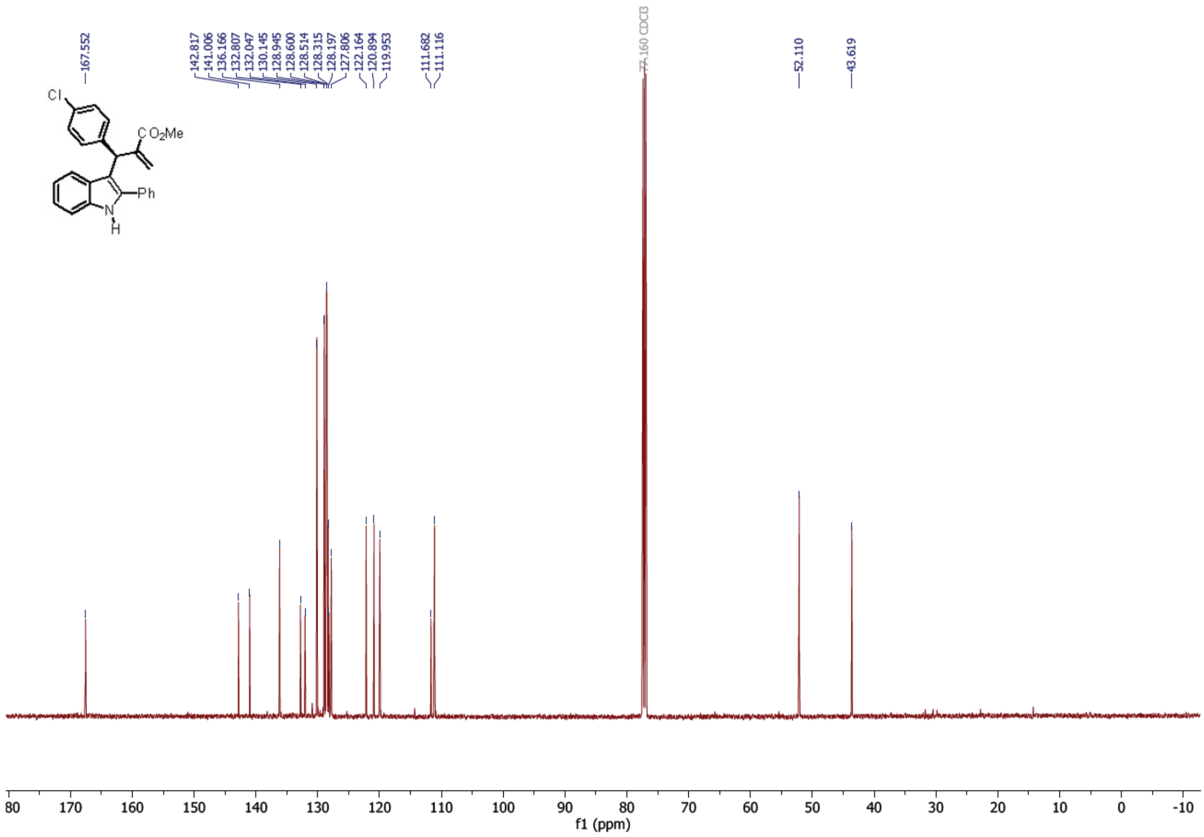

Compound **12**

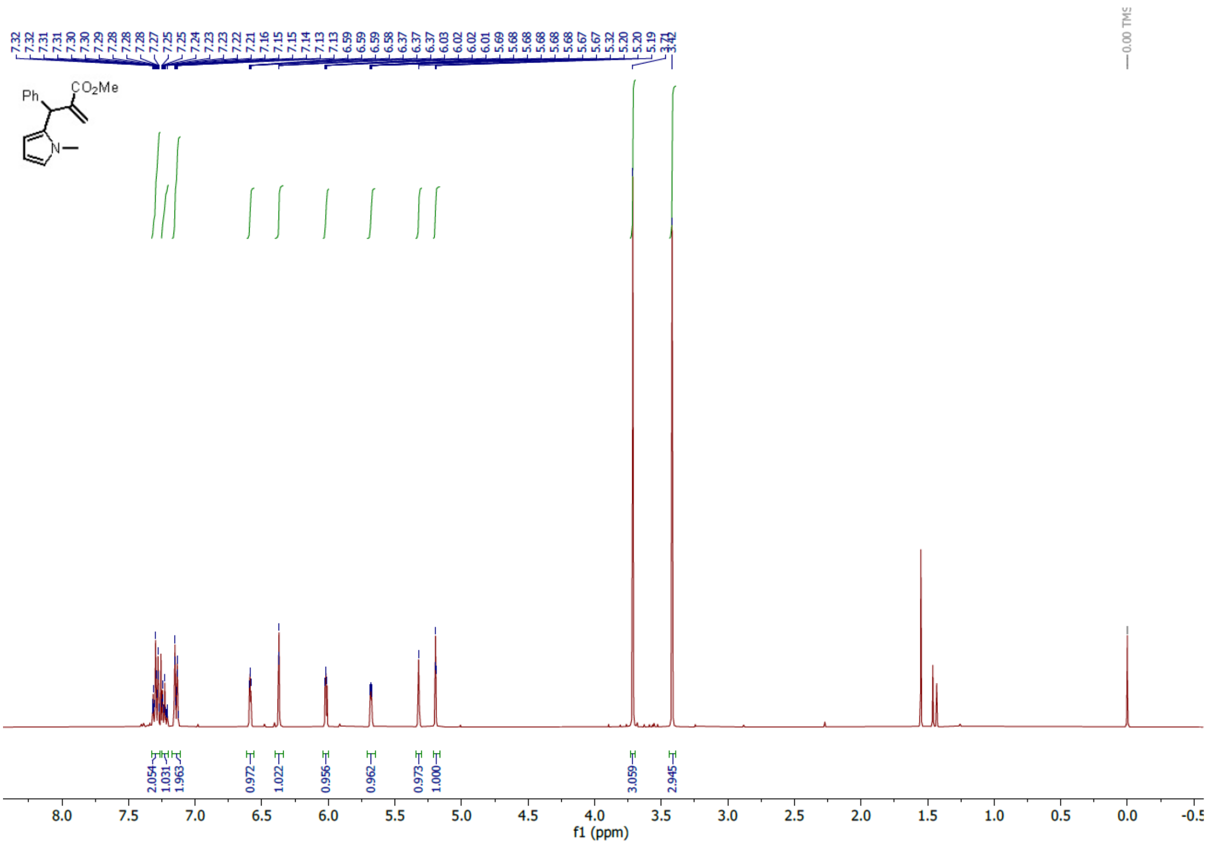

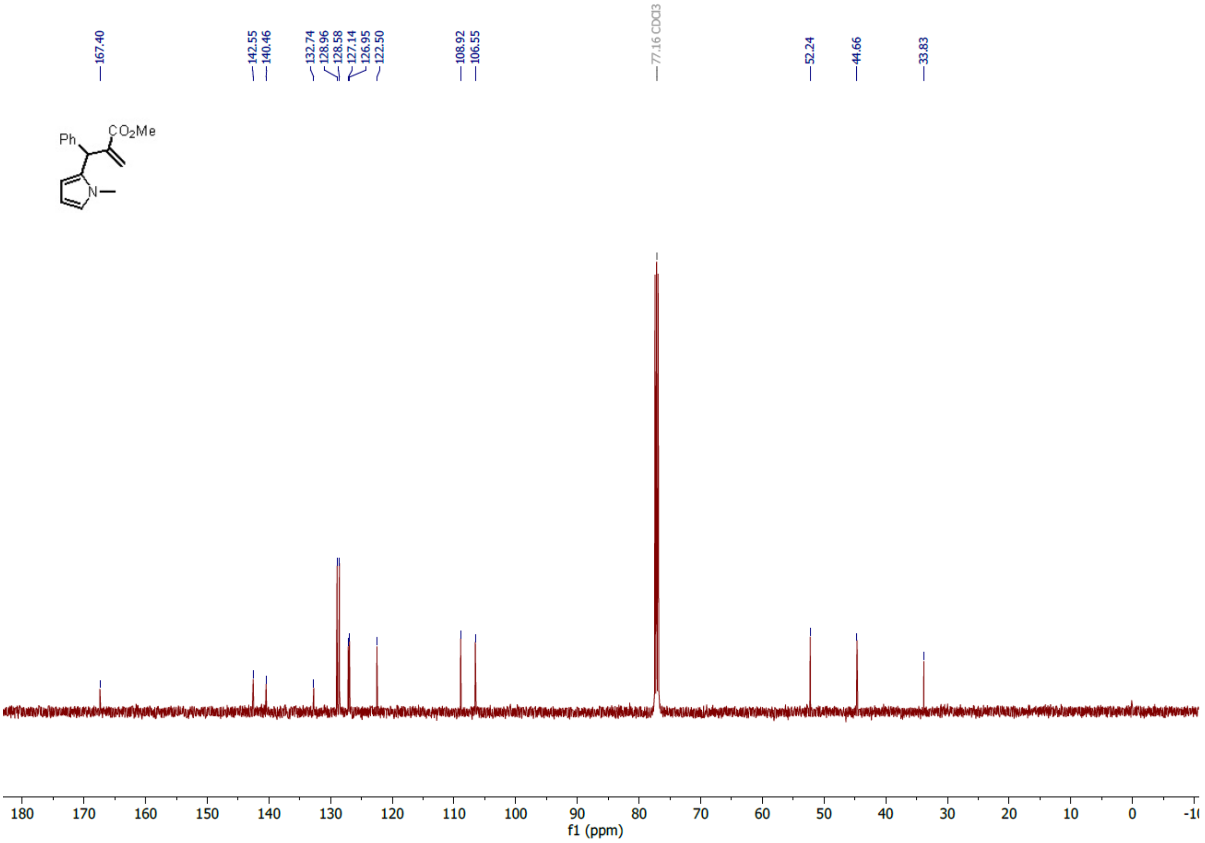

Supplement: Supplementary file 1 — ja4c11113_si_001.pdf [file ja4c11113_si_001.pdf]
